# *roX*1 and *roX*2 lncRNAs promote heterochromatinization in intestinal stem cells and impair longevity

**DOI:** 10.1038/s44319-026-00791-8

**Published:** 2026-05-09

**Authors:** Kai-Le Li, Chen Wang, Yong-Hao Li, Bo Peng, Li Zheng, Qinyun Che, Xin-Yi Lu, Donglin Li, Yu-Tong Li, Hui-Min Wei, Jian-Feng Chang, Wen-Fei Wang, Mengmeng Liu, Xiangnan Li, Fangfang Jin, Kai Liu, Yabin Li, Li-Wei Liu, Mingyang Li, Jian-Quan Ni, Ting Xie, Fang-Lin Sun

**Affiliations:** 1 School of Life Sciences and Technology, Tongji University, Shanghai, China; 2 Tsingtao Institute for Advanced Research, Tongji University, Tsingtao, China; 3 School of Basic Medical Sciences, Tsinghua University, Beijing, China; 4 State Key Laboratory of Molecular Oncology, Beijing, China; 5 Department of Biochemistry and Molecular Biology, Tulane University School of Medicine, Louisiana Cancer Research Center, New Orleans, LA USA; 6 Department of Thoracic Surgery and Lung Transplantation, First Affiliated Hospital of Zhengzhou University, Zhengzhou, China; 7 Hangzhou Zhongnanfeier Biopharmaceutical Co., Ltd., Hangzhou, China; 8 Senior Department of Gastroenterology, The First Medical Center of Chinese PLA General Hospital, Beijing, China; 9 SXMU-Tsinghua Collaborative Innovation Center for Frontier Medicine, Shanxi Medical University, Taiyuan, China; 10 Division of Life Science, The Hong Kong University of Science and Technology, Hong Kong, China

**Keywords:** Chromatin, Transcription & Genomics, RNA Biology, Stem Cells & Regenerative Medicine

## Abstract

Maintenance of the genome and epigenome stability is vital for animal longevity. Long noncoding RNAs, *roX*1 and *roX*2, are known to be important in the male X chromosome dosage complex in *Drosophila* males. However, their functions in *Drosophila* females have never been explored. This study demonstrates a role of *roX* RNAs in promoting heterochromatin formation in intestinal stem cells (ISCs) of *Drosophila* females under pathogen infection or aging. Increased heterochromatin formation in ISCs and progenitor enteroblasts (EBs) is associated with decreased active epigenetic modifications and global gene repression. Elevation of *roX* RNAs in ISCs promotes heterochromatinization and represses gene expression by recruiting heterochromatin proteins such as HP1a and Su(var)3-9. Overexpression of *roX* RNAs promotes ISCs hyperplasia, while their inactivation mitigates ISCs dysplasia and extends lifespan. Moreover, *Xist* RNA, the functional analog of *roX* RNAs, also promotes heterochromatin formation and ISCs hyperplasia in *Drosophila*, and significantly increases in aged people. Therefore, our findings reveal a role of *roX* RNAs in promoting heterochromatin expansion and controlling animal longevity.

## Synopsis

The long noncoding RNAs *roX*1 and *roX*2 promote heterochromatin formation in intestinal stem cells of female flies upon aging or infection, and *roX* RNAs also shorten lifespan.

- Upon aging or infection, *roX* RNAs are induced in intestinal stem cells of female flies, where they promote heterochromatin formation and global gene repression.
- *roX* RNAs drive heterochromatinization by recruiting heterochromatin proteins.
- *roX* RNAs promote ISC hyperplasia, and their depletion in ISCs or EBs extends lifespan.

## Abstract (web summary)

The long noncoding RNAs *roX*1 and *roX*2 promote heterochromatin formation in intestinal stem cells of female flies upon aging or infection, and *roX* RNAs also shorten lifespan.

## Graphical abstract

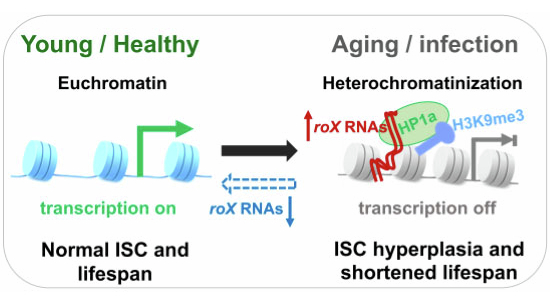

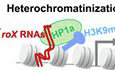

## Introduction

In eukaryotic cells, chromatin is a complex of DNA, proteins, and RNAs, which packages DNA into condensed structures and regulates gene transcription spatially. Heterochromatin, a distinct cytological superstructure in chromatin, plays critical roles in epigenetic gene silencing, cell lineage specification, maintenance of cell identity, genome integrity, and cellular reprogramming (Allshire and Madhani, 2018; Janssen et al, 2018; Grewal, 2023; Nicetto and Zaret, 2019). The multistep assembly of heterochromatin requires a cascade of regulators and pathways, including heterochromatin protein 1 (HP1), histone methyltransferases (HMTases) targeting H3 at lysine 9 (H3K9me), as well as the machinery of small interfering RNAs (siRNA) and PIWI-interacting RNAs (piRNAs) (Allshire and Madhani, 2018; Johnson and Straight, 2017; Montavon et al, 2021). By switching gene transcriptional/post-transcriptional programs, eukaryotic cells have evolved sophisticated mechanisms to sense and adapt to various intrinsic and extrinsic challenges, such as pathogen infection and aging. However, the extent of heterochromatin involvement in these responses, and the specific factors driving alterations in heterochromatin, remain largely unknown.

LncRNAs play important roles in diverse biological processes, including transcriptional regulation by modulating local chromatin architecture, post-transcriptional regulation by producing small regulatory RNAs (such as siRNAs, piRNAs, or miRNAs), and protein complex assembly by interacting with various ribonucleoproteins (Ozata et al, 2019; Marsano et al, 2019). In the *Drosophila* male, lncRNAs *roX*1 and *roX*2 are associated with the Male-Specific Lethal (MSL) complex to stimulate gene transcription on the X chromosome (Lucchesi and Kuroda, 2015; Valsecchi et al, 2021). However, whether *roX* RNAs play a role in the *Drosophila* female remains unknown.

Previous studies have established a critical role of chromatin regulation in determining the fate of stem cells, including self-renewal, proliferation, and subsequent differentiation. Tissue-resident stem cells, or adult stem cells (ASCs), play a fundamental role in maintaining tissue and organ homeostasis in the post-reproductive lifespan (Chandel et al, 2016; Schultz and Sinclair, 2016). Dysregulated gene transcription in ASCs under stress and subsequent decline in tissue/organ regenerative capacity often result in the development of aging-associated organ defects and compromise lifespan (Oh et al, 2014; Ermolaeva et al, 2018; Wu et al, 2024). Accumulated alterations in chromatin architecture, genome instability, and transcriptional outputs are implicated as major causes of impaired ASC function (Booth and Brunet, 2016; Keyes and Fuchs, 2018; Cagan et al, 2022) and reduced lifespan (Ermolaeva et al, 2018). However, the key driver triggers the turnover of the epigenomic landscape and the associated mechanism leading to aberrant gene transcription in stem cells remains poorly understood.

*Drosophila melanogaster* has served as a prime model organism in studying both heterochromatin architecture and ASCs. Its sophisticated genetic tools enable precise spatial and temporal modulation of gene activity in specific ASCs, such as the intestinal stem cells (ISCs) in the *Drosophila* midgut (Micchelli and Perrimon, 2006; Ohlstein and Spradling, 2006; Jiang and Edgar, 2012). ISCs respond to intrinsic or extrinsic cues by either self-renewing or differentiating into different cell types, accompanied by changes in their transcriptome. This dynamics process serves as an excellent system for elucidating both the interplay between the changes in heterochromatin landscape and stem cell fate decisions, as well as the underlying molecular mechanisms, in *vivo*.

In this study, we have found that heterochromatin formation or heterochromatinization is elevated in the ISCs of aged and pathogen-infected female flies. Through single-cell sequencing, *roX* RNAs are identified as key drivers of the heterochromatinization process. Furthermore, the increased expression of *roX* RNAs contributes to the hyperplasia of ISCs under stress conditions, thereby influencing lifespan. Interestingly, our results also reveal a parallel function of mammalian counterpart *Xist* RNA in driving heterochromatin formation. Together, this study has revealed the role of *roX* RNAs in modulating heterochromatin dynamics in ISCs under stress conditions and aging.

## Results

### Pathogenic bacteria and aging induce ISC hyperplasia by increasing heterochromatin

**Figure 1:**
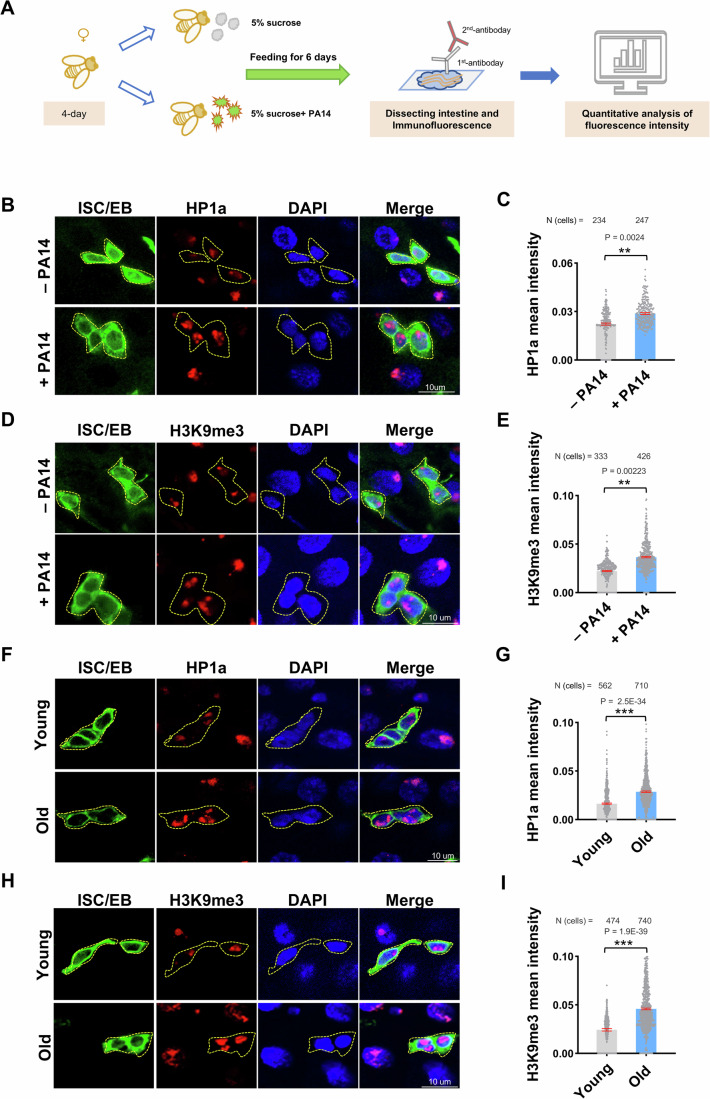
The expansion of heterochromatin in pathogen-stressed and aged ISCs/EBs. (**A**) Schematic diagram of the PA14 infection experiment. 4 DAE female *Drosophila* were fed 5% sucrose with or without PA14 for 6 days. The guts were then dissected, and IF was performed. (**B**) The change in HP1a expression (red) in ISCs/EBs (green, dotted circle) after PA14 infection for 6 days; the genotypes are *esg-Gal4, UAS-GFP*. Upper panel, midgut from the control without infection; lower panel, midgut from the line after PA14 infection. (**C**) The bar graph indicates the quantification of HP1a intensity from the control and infected midguts. The cell numbers indicated on the graph represent the total count of ISCs/EBs analyzed per condition, combined from *N* = 3 independent biological replicates. (**D**) Antibody staining shows the intensity of H3K9me3 (red) in ISCs/EBs (green, dotted circle) from uninfected and infected flies of the same genotype and under the same conditions as used in (**B**). (**E**) Quantification of H3K9me3 intensity in each ISC/EB with or without infection. The cell numbers indicated on the graph represent the total count of ISCs/EBs analyzed per condition, combined from *N* = 3 independent biological replicates. (**F**) The expression of HP1a (red) in ISCs/EBs (green, dotted circle) between young *Drosophila* (7 DAE) and old *Drosophila* (67 DAE, aged) midguts. Upper panel, midgut from young as a control (genotype: *esg-Gal4, UAS-GFP*), 7 DAE; lower panel, midgut from aged, same genotype as young, 67 DAE. (**G**) The bar graph indicates the quantification of HP1a intensity in the young and old midguts. The cell numbers indicated on the graph represent the total count of ISCs/EBs analyzed per condition, combined from *N* = 3 independent biological replicates. (**H**) The intensity of H3K9me3 (red) in ISCs/EBs (green, dotted circle) from young and old midguts, detected by antibody. The genotype and condition are the same as those used in (**F**). (**I**) The bar graph indicates quantification of H3K9me3 intensity from young and old midguts. The cell numbers indicated on the graph represent the total count of ISCs/EBs analyzed per condition, combined from *N* ≥ 3 independent biological replicates. The center values are the averages, and the error bars indicate the s.e.m. *P* values were obtained by two-tailed unpaired Student’s *t* test. n.s., not significant, *P* ≥ 0.05, *P < 0.05, **P < 0.01, ***P < 0.0001. All images were captured from adult female posterior midguts. Scale bar, 10 μm. Source data are available online for this figure.

**Figure EV1:**
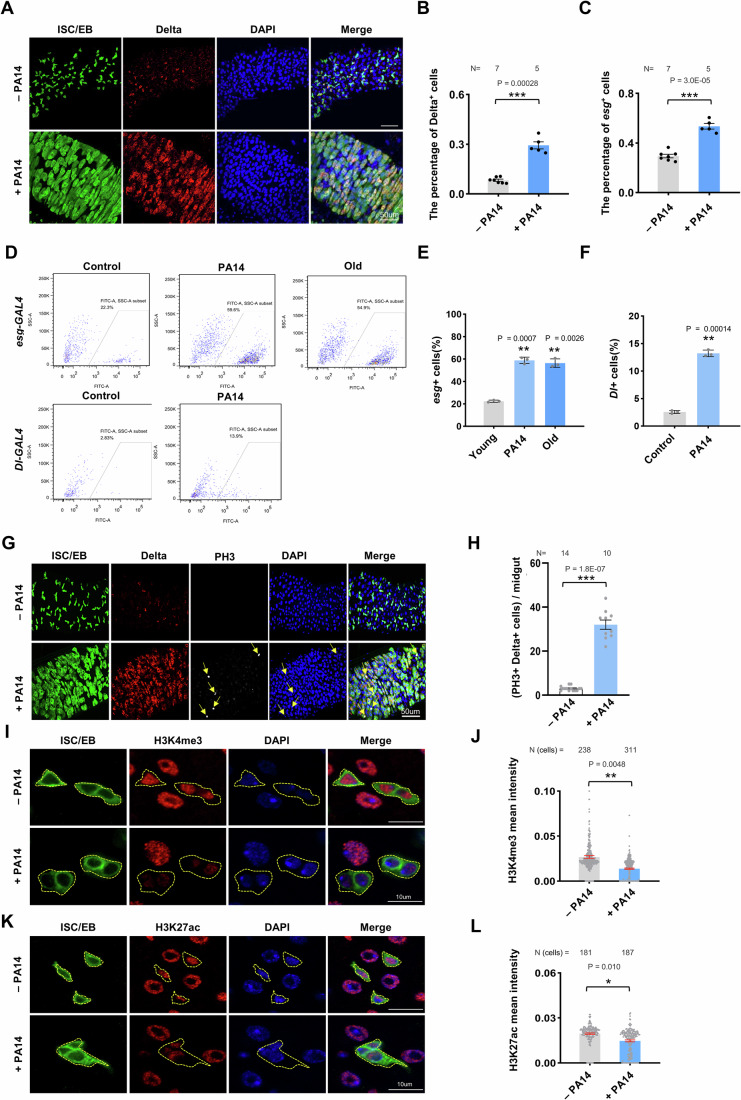
Pathogen stress promotes ISC hyperplasia with a reduction of euchromatinization. (**A**) IF showing the ISCs/EBs (GFP, green) and ISCs (indicated by Delta antibody, red) in control and infected (fed with PA14) *Drosophila*. (**B**) Statistical results of the ISCs in (**A**). Data are from three independent biological replicates. The number of fields of view analyzed is indicated in the figure. (**C**) Statistical results of ISCs/EBs in (**A**). Data are from three independent biological replicates. The number of fields of view analyzed is indicated in the figure. (**D**) Gating strategies for identifying esg⁺ cells (ISCs/EBs) and Delta⁺ ISCs from the midguts of *esg-Gal4, UAS-GFP* or *Dl-Gal4* driven *UAS-GFP* female flies under aging and infection conditions.” (**E**) Quantification of the percentage of esg⁺ cells (ISCs/EBs) shows a significant increase in both aged and PA14-infected conditions compared to controls. *N* = 3. (**F**) The percentage of Delta⁺ cells (ISCs) was quantified under infected conditions. *N* = 3. (**G**) PH3 staining of *esg-GAL4* female flies under PA14-infected and uninfected conditions. Arrows indicate PH3⁺Delta⁺ cells. (**H**) The number of PH3⁺Delta⁺ cells per midgut was quantified. (*N* = 3, the number of midguts is labeled on the graph). (**I**) Antibody staining shows the intensity of H3K4me3 (red) in ISCs/EBs (green, dotted circle) of control and PA14-infected *Drosophila*. Upper panel, midgut from uninfected *Drosophila* as a control (genotype: *esg-Gal4, UAS-GFP*); lower panel, midgut from PA14-infected *Drosophila*, same genotype as a control. (**J**) The bar graph shows quantification of H3K4me3 intensity from the control and infected gut. *N* = 3. The number of cells counted is labeled at the top of the bar graph, and the *P* value is included. (**K**) IF shows the intensity of H3K27ac (red) in ISCs/EBs (green, dotted circle) of control and PA14-infected *Drosophila*. Upper panel, midgut from uninfected *Drosophila* as a control (genotype: *esg-Gal*4*, UAS-GFP*); lower panel, midgut from PA14-infected *Drosophila*, same genotype as a control. (**L**)The bar graph indicates quantification of H3K27ac intensity from the control and infected gut. *N* = 3. The number of cells is indicated on the graph., and the *P* value is included. The center values are the averages, and the error bars indicate the s.e.m. *P* values were obtained by two-tailed unpaired Student’s *t* test. n.s., not significant, *P* ≥ 0.05, *P < 0.05, **P < 0.01, ***P < 0.0001. All images were captured from adult female posterior midguts. Scale bar, 10 μm.

Exposure to bacterial pathogens or gut microbial dysbiosis in gastrointestinal systems can pose critical challenges to the health and survival of multicellular organisms (Yu et al, 2017; Wong and Yu, 2023; Ferguson and Foley, 2022). Bacterial pathogens, commensal bacteria, or damage in the *Drosophila* intestine have been shown to cause ISC hyperproliferation (Clark et al, 2015; Gervais and Bardin, 2017; Amcheslavsky et al, 2009; Tian et al, 2015, 2022), a phenomenon reminiscent of what occurs in aged *Drosophila*. To investigate if pathogenic bacteria infection alters the heterochromatin in ISCs, we fed 4-day-old female flies in which the *esg-Gal4* driver directs GFP expression specifically to ISCs and EBs, with human pathogen *Pseudomonas aeruginosa* (PA14) known to infect *Drosophila* (Rahme et al, 1995) in 5% sucrose for 6 days, which were compared to the control flies fed with only 5% sucrose and subsequently dissected their intestines for cytological analysis (Fig. 1A). As shown in Fig. EV1A–C, the number of ISCs, identified by specific anti-Delta antibody staining, which is widely used to mark ISCs, significantly increases after infection. To quantify Delta⁺ and esg⁺ cells, we performed FACS analysis using *Delta-Gal4* and *esg-Gal4* lines in combination with UAS-GFP. Consistent with the immunofluorescence data, FACS quantification demonstrated a significant elevation of Delta⁺ and esg+ cells under infection conditions (Fig. EV1D–F). Furthermore, quantification of mitotic ISCs using the mitosis marker PH3 revealed a concurrent increase in the number of mitotic ISCs (Fig. EV1G,H), suggesting that PA14 infection can efficiently trigger ISC hyperplasia. Next, we assessed the changes in repressive heterochromatin by examining markers such as HP1a and H3K9me3. Interestingly, in contrast to the uninfected control, the expression of both H3K9me3 and HP1a was significantly increased in pathogen-infected ISCs and progenitor enteroblasts (ISCs/EBs) labeled by GFP, supporting the acquisition of heterochromatin in ISCs/EBs following pathogen infection (Fig. 1B–E). In contrast, the expression of active chromatin markers, such as H3K4me3 and H3K27ac, was decreased (Fig. EV1I–L). These observations suggest that exposure to pathogenic bacteria leads to significantly enhanced heterochromatinization in ISCs/EBs.

**Figure EV2:**
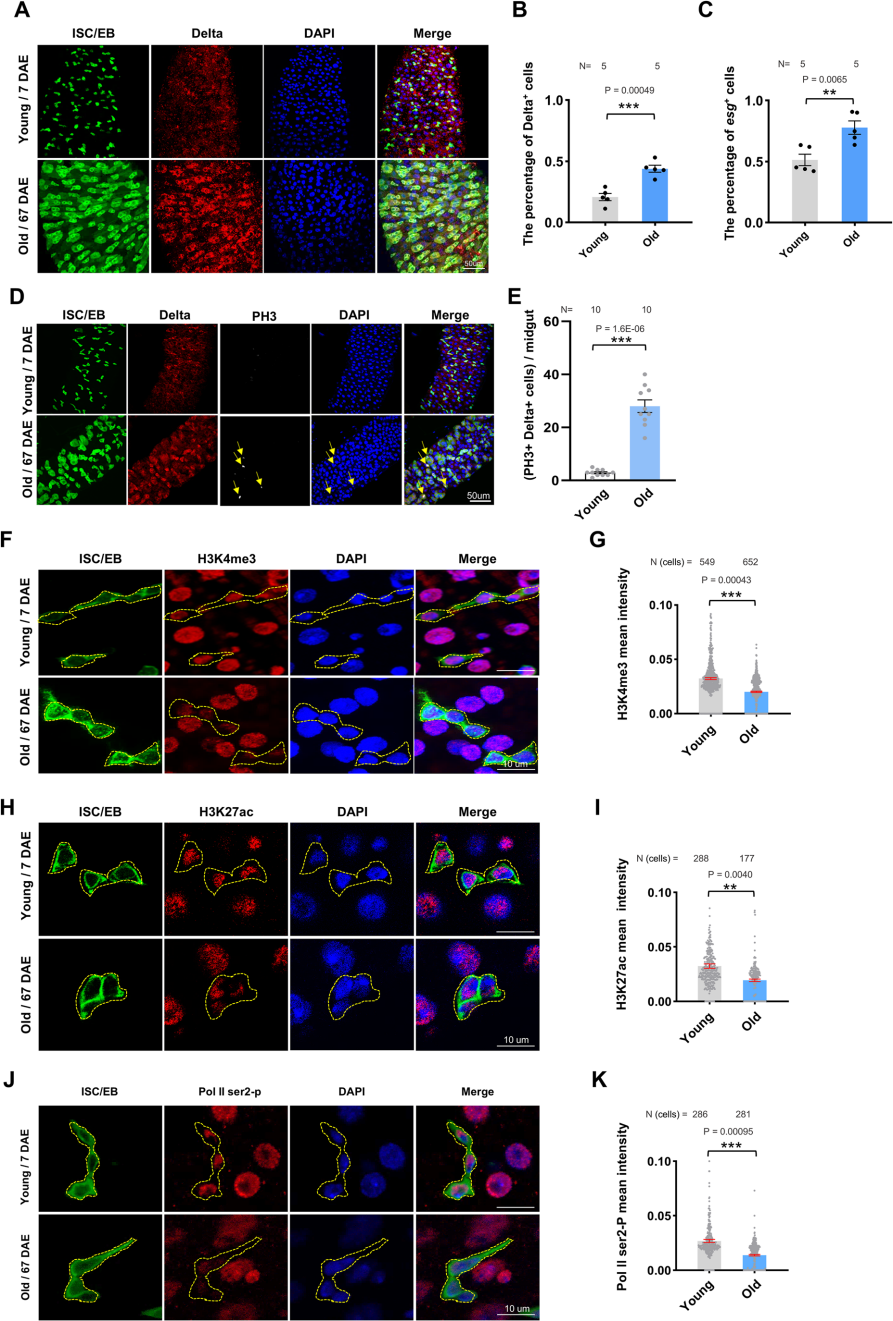
ISC hyperplasia with a decrease of euchromatinization in aging. (**A**) IF shows the ISCs/EBs (GFP, green) and ISCs alone (Delta antibody recognizing ISC, red) in Drosophila (*esg-Gal4*, *UAS-GFP*) at 7 DAE and 67 DAE. (**B**) Statistical analysis of the ISCs in (**A**). Data are from three independent biological replicates. The number of fields of view analyzed is indicated in the figure. (**C**) Statistical analysis of ISCs/EBs in (**A**). Data are from three independent biological replicates. The number of fields of view analyzed is indicated in the figure. (**D**) PH3 staining of *esg-GAL4* female flies from young and aged groups. Arrows indicate PH3⁺Delta⁺ cells. (**E**) The number of PH3⁺Delta⁺ cells per midgut was quantified. *N* = 3. The number of counted midguts is marked at the top. (**F**) Antibody staining shows the intensity of H3K4me3 (red) in ISCs/EBs (green, dotted circle) in young and old posterior midguts. Upper panel, midgut from young Drosophila as a control (genotype: *esg-Gal4, UAS-GFP*), 7 DAE; lower panel, midgut from aged Drosophila, same genotype as young, 67 DAE. (**G**) The bar graph shows the quantification of H3K4me3 intensity from young and old midguts. *N* = 3. The number of cells is indicated on the graph, and the *P* value is included. (**H**) Antibody staining showing the intensity of H3K27ac (red) in ISCs/EBs (green, dotted circle) from young and old midguts, the same genotype and the same condition as used in (**F**). (**I**) The bar graph indicates the quantification of H3K27ac intensity from young and old midguts. *N *= 3. The number of cells is indicated on the graph, and *P* value is included. (**J**) IF showing the intensity of Pol II Ser2-p (red) in ISCs/EBs (green, dotted circle) from young and old midguts with the same genotype and the same conditions as used in (**F**). (**K**) The bar graph shows the quantification of Pol II Ser2-p intensity in young and old midguts. *N* = 3. The number of cells is indicated on the graph, and *P* values are included. The center values are the averages, and the error bars indicate the s.e.m. *P* values were obtained by two-tailed unpaired Student’s *t* test. n.s., not significant, *P* ≥ 0.05, *P < 0.05, **P < 0.01, ***P < 0.0001. All images were captured from adult female posterior midguts. Scale bar, 10 μm.

Next, we examined the landscape of heterochromatin in ISCs during aging. The proliferation and differentiation of aged ISCs are deregulated in response to physiological perturbations of the gut epithelia, resulting in ISC dysplasia (Amcheslavsky et al, 2009; Tian et al, 2015, 2022). Given that naturally aged adult flies typically have an average lifespan of approximately 70 DAE (Ziehm et al, 2013), we designated adult flies at 7 DAE as young flies and those at 67 DAE as naturally aged flies. Compared with young flies, the number of ISCs/EBs marked by esg-GFP, as well as ISCs themselves recognized by the anti-Delta antibody, significantly increased in aged female flies (Fig. EV2A–C), consistent with previous studies (Jasper, 2020). FACS-based quantification further confirmed a significant increase in the esg⁺ population under aging conditions (Fig. EV1D,E). In addition, we observed an elevated number of mitotic ISCs (Fig. EV2D,E), further confirming the age-associated hyperproliferation. The expression of HP1a and H3K9me3 in ISCs/EBs from aged flies significantly increases compared with young flies (Fig. 1F–I), whereas the expression of both H3K4me3 and H3K27ac, active chromatin markers, significantly decreased (Fig. EV2F–I). To further examine any changes in the transcriptional activity, we analyzed the expression levels of phosphorylated RNA polymerase II at Ser2 (Ser2-p), a known marker of transcriptional elongation (Jonkers and Lis, 2015). In contrast to the young ISCs/EBs, the expression of Ser2-p in aged ISCs/EBs significantly diminishes (Fig. EV2J,K). Taken together, these observations indicate that heterochromatinization occurs in the ISCs/EBs of the bacterial pathogen-infected and aged females.

Since Esg⁺ cells include both ISCs and EBs, we accordingly stratified our analysis into three different cell types: Esg⁺Delta⁺ (ISCs), Esg⁺Delta^−^ (EBs), and Esg^−^Delta^−^ (other cells). Consistent with the findings from Fig. 1, aging or pathogen infection significantly intensified the signals of HP1a and H3K9me3 in both ISCs and EBs. Moreover, the other cells (Esg^−^Delta^−^) also exhibited elevated heterochromatin levels (Appendix Fig. S1A–J). To validate the chromatin alterations observed by immunofluorescence, we performed western blot to detect the protein level of HP1a and H3K9me3 in response to infection. Compared to controls, both gut samples and FACS-sorted ISCs/EBs showed elevated expression of these heterochromatin markers (Appendix Fig. S2A,B), further confirming that the heterochromatinization is related to aging and pathogen infection in the intestine. In male flies, the same effects on heterochromatin were observed based on our analysis (Appendix Fig. S3A–J). Altogether, the above data collectively demonstrate that both infection and aging induce heterochromatin enhancement in the *Drosophila* intestine.

### Global gene silencing couples with *roX* RNA activation in hyperplastic ISCs

**Figure 2:**
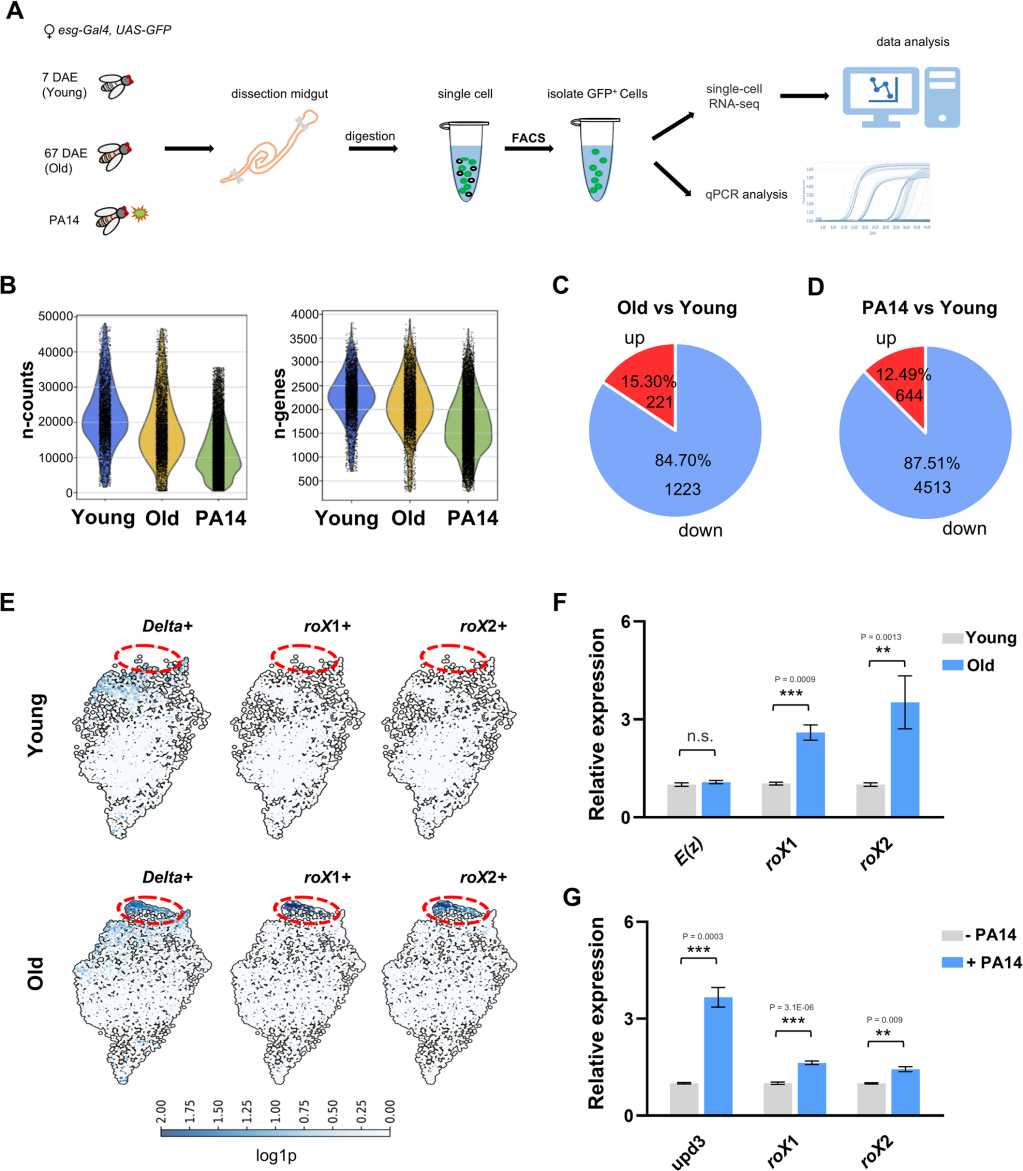
The scRNA-seq atlas showed a decrease in global transcription, while the expression of *roX* RNAs was elevated. (**A**) The scRNA-seq procedure used to analyze aging and PA14-treated ISCs/EBs. (**B**) UMI and gene counts of scRNA-seq from control, old and PA14 treatment. (**C**) The pie chart shows the percentage of up- and downregulated genes in old flies' ISCs compared with young flies. The total number of differentially expressed genes is indicated in the figure. (**D**) The pie chart shows the percentage of up- and downregulated genes in PA14 treatment flies ISCs compared with young flies. The total number of differentially expressed genes is indicated in the figure. (**E**) UMAP embeddings of the *Delta* transcription level. Following the increase in *Delta* transcription in the aged ISCs/EBs, *roX*1 or *roX*2 was preferentially present in *Delta*-highly enriched cells. The scale indicates the log1p-transformed normalized expression value of the indicated gene. The red circles highlight the regions in the UMAP where cells form a distinct cluster with detectable Delta expression. (**F**) Real-time PCR shows the elevation of *roX*1 or *roX*2 in ISCs/EBs during aging; *E(z)* serves as a negative control. GFP-marked ISCs/EBs were isolated by FACS and used for this assay. Real-time PCR was normalized against *rp*49, yielding results then normalized to the first control (*esg-Gal4, UAS-GFP*). Average results from three independent experiments are shown (*N* = 3). Error bars indicate the s.e.m., and *P* values are shown on the bar graph. (**G**) Real-time PCR shows the upregulation of *roX* RNAs in ISCs/EBs after PA14 infection. *Upd*3 is a positive control. As in (**F**), real-time PCR was normalized against *rp49* and then normalized to the control without PA14 infection. The results are averaged from three independent experiments (*N* = 3). Error bars indicate the s.e.m., and *P* values are shown on the bar graph. *P* values were obtained by two-tailed unpaired Student’s *t* test. n.s., not significant, *P* ≥ 0.05, *P < 0.05, **P < 0.01, ***P < 0.0001. Source data are available online for this figure.

To evaluate whether any transcriptional changes are related to the hyperplasia of ISCs/EBs in the context of aging or exposure to pathogenic bacteria, GFP-marked ISCs/EBs were isolated by fluorescence-activated cell sorting (FACS) from young female, aged female, and female *Drosophila* midguts fed with PA14, followed by single-cell RNA sequencing (scRNA-seq), focusing on Delta-expressing ISCs (Fig. 2A). The median unique molecular identifier (UMI) counts and genes from ISCs, compared with controls, decrease significantly in both aged and PA14-treated flies (Fig. 2B), indicating global repression in transcription under aging/pathogen-stressed conditions. Consistently, the majority of detected genes are downregulated, with 85% downregulation of genes in aged ISCs and 88% of genes in ISCs after PA14 treatment (Fig. 2B–D). Furthermore, we performed Gene Ontology (GO) term enrichment analysis to investigate whether similar biological pathways were affected under aging and PA14 infection. The downregulated genes in both conditions were enriched for terms such as “ATP synthesis” and “ribosome biogenesis”, indicating a coordinated decline in metabolic and translational capacity with aging or infection (Appendix Fig. S4A). Notably, the upregulated genes in both conditions were strongly enriched for “DNA-binding transcription repressor activity” (Appendix Fig. S4B), consistent with our observation of heterochromatin expansion and the global reduction in transcriptional activity in aged or infected ISCs/EBs.

Interestingly, among the few increased RNA transcripts, two lncRNAs, *roX*1 and *roX*2, were upregulated in ISCs/EBs, predominantly in Delta-positive ISCs (Fig. 2E). Quantification revealed that upon PA14 infection or aging, increased *roX* expression was detected in ~15–20% of Delta⁺ ISCs (Appendix Fig. S5A,B). To validate the increase in *roX* RNA transcripts, we performed quantitative RT-PCR (qRT-PCR) using total RNA extracted from the FACS-isolated ISCs/EBs from young, aged, and PA14-treated young female adult midguts. As for the aged flies, the expression of *roX*1 and *roX*2 was significantly upregulated by ~twofold and threefold, respectively. Upon PA14 infection, the expression of *upd3*, which is a positive marker for infection, was robustly induced by approximately threefold, suggesting the infection was successful. Then we detected both *roX*1 and *roX*2, their expression in ISCs/EBs showed a significant increase of ~1.5-fold (Fig. 2F,G). These results confirmed the elevation of *roX* RNAs in female ISCs/EBs under stressed conditions. In addition, we found that the expression levels of *roX* RNAs increased with the duration of PA14 treatment in females. In contrast, PA14 infection did not significantly induce *roX* RNA expression in males (Appendix Fig. S6). This finding suggests that the upregulation of *roX* RNAs in response to stress is preferential in females.

### *roX* RNAs promote the formation of heterochromatin

**Figure 3:**
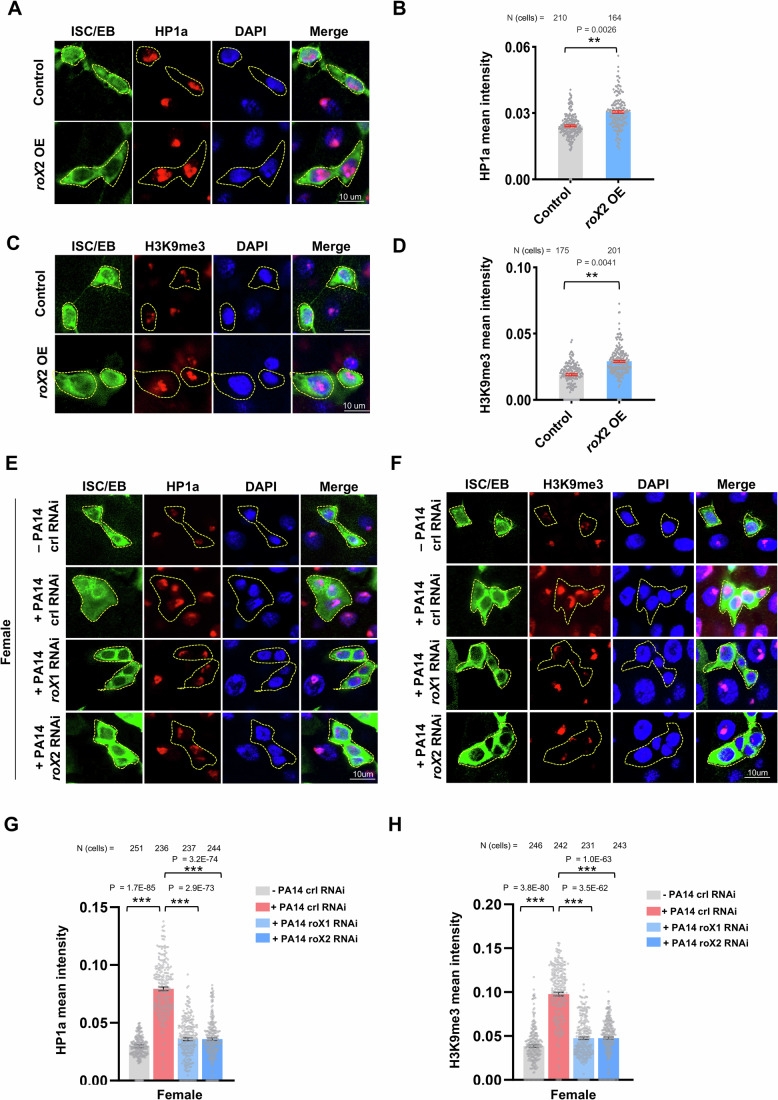
*roX* RNAs promote heterochromatin formation in ISCs. (**A**) The intensity of HP1a (red) in ISCs/EBs (green, dotted circle) in response to *roX*2 overexpression (*roX*2 OE). *UAS-roX*2 was driven by *esg-Gal4, UAS-GFP*, and the line carrying *esg-Gal4, UAS-GFP* served as the control. (**B**) Quantitation of the intensity of HP1a in (**A**). The cell numbers indicated on the graph represent the total count of ISCs/EBs analyzed per condition, combined from *N* = 3 independent biological replicates. (**C**) The change in H3K9me3 (red) in ISCs/EBs (green, dotted circle) after *roX*2 OE under the same procedure performed in (**A**). (**D**) Quantitation of the intensity of H3K9me3 in (**C**). The cell numbers indicated on the graph represent the total count of ISCs/EBs analyzed per condition, combined from *N* = 3 independent biological replicates. (**E**) Antibody staining shows the intensity of HP1a (red) in ISCs/EBs (green, dotted circle) under different conditions. The control group, which expressed luciferase RNAi driven by *esg-Gal4, UAS-GFP*, was either treated or not treated with PA14. *roX*1 or *roX*2 RNAi was also driven by *esg-Gal4, UAS-GFP*, and all were treated with PA14. (**F**) The intensity of H3K9me3 (red) in ISCs/EBs (green, dotted circle) under the same conditions as described in (**E**). (**G**) The bar graph shows the quantification of HP1a intensity in (**E**). *N* = 3. The number of cells is indicated on the graph. (**H**) The bar graph shows the quantification of the H3K9me3 intensity in (**F**). *N* = 3. The number of cells is indicated on the graph. The center values are the averages, and the error bars indicate the s.e.m. *P* values were obtained by two-tailed unpaired Student’s *t* test. n.s., not significant, *P* ≥ 0.05, *P < 0.05, **P < 0.01, ***P < 0.0001. All images were captured from adult female posterior midguts. Scale bar, 10 μm. Source data are available online for this figure.

The expansion of heterochromatin and ectopic expression of *roX* RNAs in the female hyperplastic ISCs indicate a potential role for *roX* RNAs in heterochromatin formation. To examine the causal relationship between *roX* RNA expression and heterochromatinization, we generated a transgenic *Drosophila* line that specifically overexpressed *roX*2 (Appendix Fig. S7A) to mimic its elevation in ISCs/EBs of pathogen-infected and aged flies, since *roX*2 functions similarly to *roX*1 but with a shorter transcript length, only one-quarter of *roX*1 (Ilik et al, 2013). As shown in Fig. 3A–D, ectopic expression of *roX*2 alone in ISCs/EBs under normal culture conditions caused a significant expansion of heterochromatin, indicated by the increased expression of HP1a and H3K9me3, suggesting that *roX* RNAs promote the heterochromatinization.

To further examine whether the expansion of heterochromatin in hyperplastic ISCs is due to the elevation of *roX* RNAs, we conditionally depleted *roX1* and *roX2* RNAs (Appendix Fig. S7B,C) from the ISCs of the pathogen-infected females. While PA14 pathogen infection leads to a significant accumulation of repressive heterochromatin as evidenced by HP1a and H3K9me3 expression (Fig. 3E–H), depletion of either *roX*1 or *roX*2 in ISCs/EBs significantly decreases HP1a and H3K9me3 expression to the extent as in the untreated ISCs/EBs controls (Fig. 3E–H). Taken together, *roX* RNAs are necessary and sufficient for heterochromatin expansion in female hyperplastic ISCs/EBs.

### *roX* RNAs are required for gene transcriptional silencing

**Figure 4:**
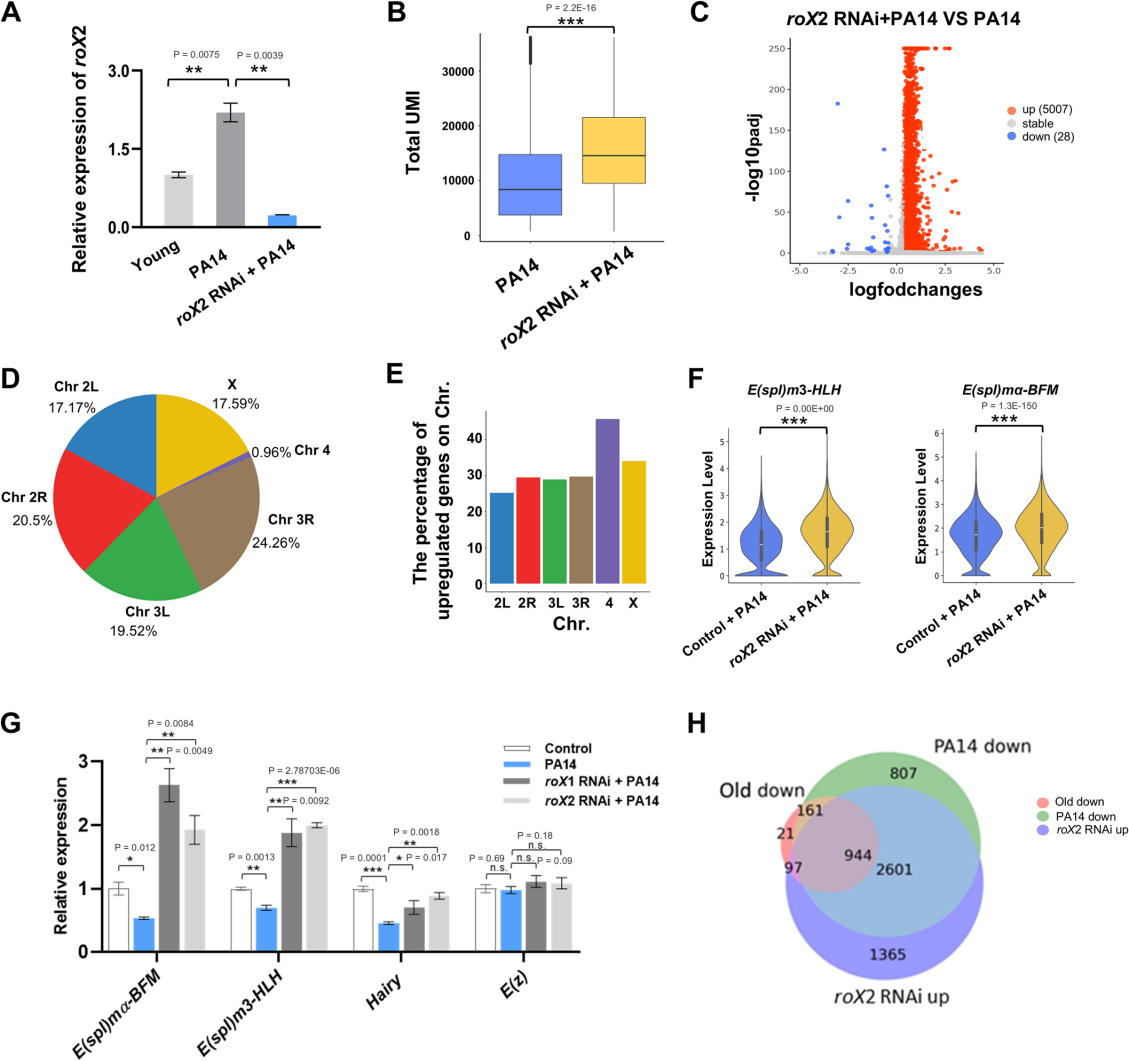
scRNA-seq shows *roX* RNAs are transcriptional repressors and modulate *Notch* genes in ISCs. (**A**) ISCs/EBs carrying *esg-Gal4, UAS-GFP* (controls, young flies treated without PA14), and treated with PA14 or crossed to *roX*2 hairpin lines under treatment with PA14. Real-time PCR was performed using two different primer sets probing *roX*2, and the results were first normalized against *rp*49, and then normalized to the first control (*esg-Gal4, UAS-GFP*) without PA14 treatment. Average results from three independent experiments are shown (*N* = 3). Error bars indicate the s.e.m. (**B**) Box plot of UMI counts showing the gene expression levels from control and *roX*2 RNAi (driven by *esg-Gal4, UAS-GFP*) under PA14 treatment. GFP-marked ISCs/EBs were isolated by FACS, and then scRNA-seq was performed. Data are presented as box plots. The lower whisker represents the 5th percentile, the lower box bound the 25th percentile, the center line the median (50th percentile), the upper box bound the 75th percentile, and the upper whisker the 95th percentile. Statistical significance was assessed by the Wilcoxon test, with *P* values shown on the graph. PA14 group, *n* = 13,136 cells; *roX*2 RNAi + PA14 group, *n* = 9580 cells. (**C**) The number of upregulated genes is nearly 180-fold more than downregulated genes after *roX*2 RNAi (driven by *esg-Gal4, UAS-GFP*) and control (*esg-Gal4, UAS-GFP*) groups. Point plot comparing the mean gene expression levels between groups (fold change threshold = 1.3, between −5 and +5). Every point shows a gene’s condition. (**D**) The percentage of upregulated genes on each chromosome, marked by different colors. (**E**) The percentage of the upregulated genes to the total number of genes on each chromosome. Mitochondrial genes are not included. Upregulated genes from RNA-seq are described in (**B**). (**F**) Expression levels of *E(spl)m*3*-HLH* and *E(spl)mα-BFM* in infected *Drosophila* with or without *roX*2 RNAi are shown in the violin chart. Data are presented as box plots. The lower whisker represents the 5th percentile, the lower box bound the 25th percentile, the center line the median (50th percentile), the upper box bound the 75th percentile, and the upper whisker the 95th percentile. Statistical significance was assessed by the Wilcoxon test, with *P* values shown on the graph. PA14 group, *n* = 13,136 cells; *roX*2 RNAi + PA14 group, *n* = 9580 cells. (**G**) qPCR shows the *Notch* target genes were regulated by *roX* RNAs, *E*(*z*) as a control (*N* = 3). (**H**) Venn diagram showing the intersection of genes induced by old (downregulated genes), PA14 infection (downregulated genes) and *roX*2 RNAi under PA14 infection (upregulated genes). The center values are the averages, and the error bars indicate the s.e.m. *P* values were obtained by two-tailed unpaired Student’s *t* test, except for (**F**). n.s., not significant, *P* ≥ 0.05, *P < 0.05, **P < 0.01, ***P < 0.001. Source data are available online for this figure.

The observation that *roX* RNAs intrinsically promote heterochromatinization prompted us to investigate whether alterations in *roX* RNAs affect gene silencing in ISCs/EBs. First, we assessed the knockdown efficiency of *roX*2, driven by *esg-Gal4 UAS-GFP*, under PA14-infected conditions. Using the FACS-purified ISCs/EBs from the PA14-infected females, the *roX2*-depleted PA14-infected flies, and the uninfected control females, we observed a significant reduction in the *roX*2 RNA levels following RNAi knockdown: with about 20% remaining compared to the uninfected control and 10% to the PA14-infected control (Fig. 4A), indicating that *roX*2 is knocked down efficiently in ISCs/EBs. Then, the remaining purified cells were used for further scRNA-seq analysis. Compared to the PA14-infected control, interestingly, the total UMI counts detected by scRNA-seq significantly increased after *roX*2 knockdown (Fig. 4B). Furthermore, more than 90% of those detected genes showed upregulated transcription after *roX*2 depletion, with fold change threshold is 1.3 (Fig. 4C), if switch the fold change threshold to 2, the same results were obtained (Appendix Fig. S8). Our further transcriptome analysis has identified over 5000 upregulated genes following *roX*2 knockdown, spanning all chromosomes, including the 4th chromosome. The fourth chromosome is highly heterochromatic (Sun et al, 2000, 2004), with abundant HP1a association. After *roX*2 depletion, the increase in gene transcription on the 4th chromosome, indicating *roX* RNAs are involved in heterochromatin formation (Fig. 4D,E). These results support that *roX*2 acts as an epigenetic repressor to silence gene expression in female hyperplastic ISCs/EBs.

Further GO-term analysis has also uncovered the potential mechanisms underlying the ISCs hyperplasia. These upregulated genes include the genes involved in cell growth, differentiation, signaling transduction, and cell development (Appendix Fig. S9), highlighting the essential role of *roX* RNAs in the hyperplasia of ISCs. Notably, the transcription levels of *E(spl)m*3*-HLH* and *E(spl)mα-BFM*, two target genes of the *Notch* signaling pathway, are significantly increased in the PA14-treated ISCs upon *roX*2 depletion (Fig. 4F). We used the FACS-isolated ISCs/EBs from the control, the PA14 infected control, the *roX1* or *roX2*-depleted PA14-treated one, and qRT-PCR to show that the downregulation of *Notch* target genes, such as *E(spl)m*3*-HLH, E(spl)mα-BFM*, and *Hairy*, by PA14 treatment can be significantly reversed upon *roX* RNAs depletion (Fig. 4G), further supporting the suppressive role of *roX*2 in gene expression. The loss of Notch function is associated with defective lineage commitment and exponential expansion of stem-like cells (Micchelli and Perrimon, 2006; Ohlstein and Spradling, 2006; Jiang and Edgar, 2012), while overactivating Notch signaling triggers rapid ISCs differentiation (Jiang et al, 2009). Therefore, these results demonstrate that *roX* RNAs are the driver for female ISCs hyperplasia by suppressing Notch signaling.

By comparing the *roX*2-repressed genes in the pathogen-infected ISCs with those downregulated in aged ISCs, over 80% of the genes overlapped (Fig. 4H), suggesting that the transcriptomic changes in aged ISCs/EBs are similar to those caused by pathogen infection. Importantly, both conditions are associated with the ectopic expression of *roX* RNAs, supporting the notion that *roX* RNAs serve as transcriptional repressors by promoting heterochromatin expansion in female ISCs/EBs in the contexts of both aging and pathogen infection.

### *roX* RNAs promote ISC hyperplasia and shorten the lifespan

**Figure 5:**
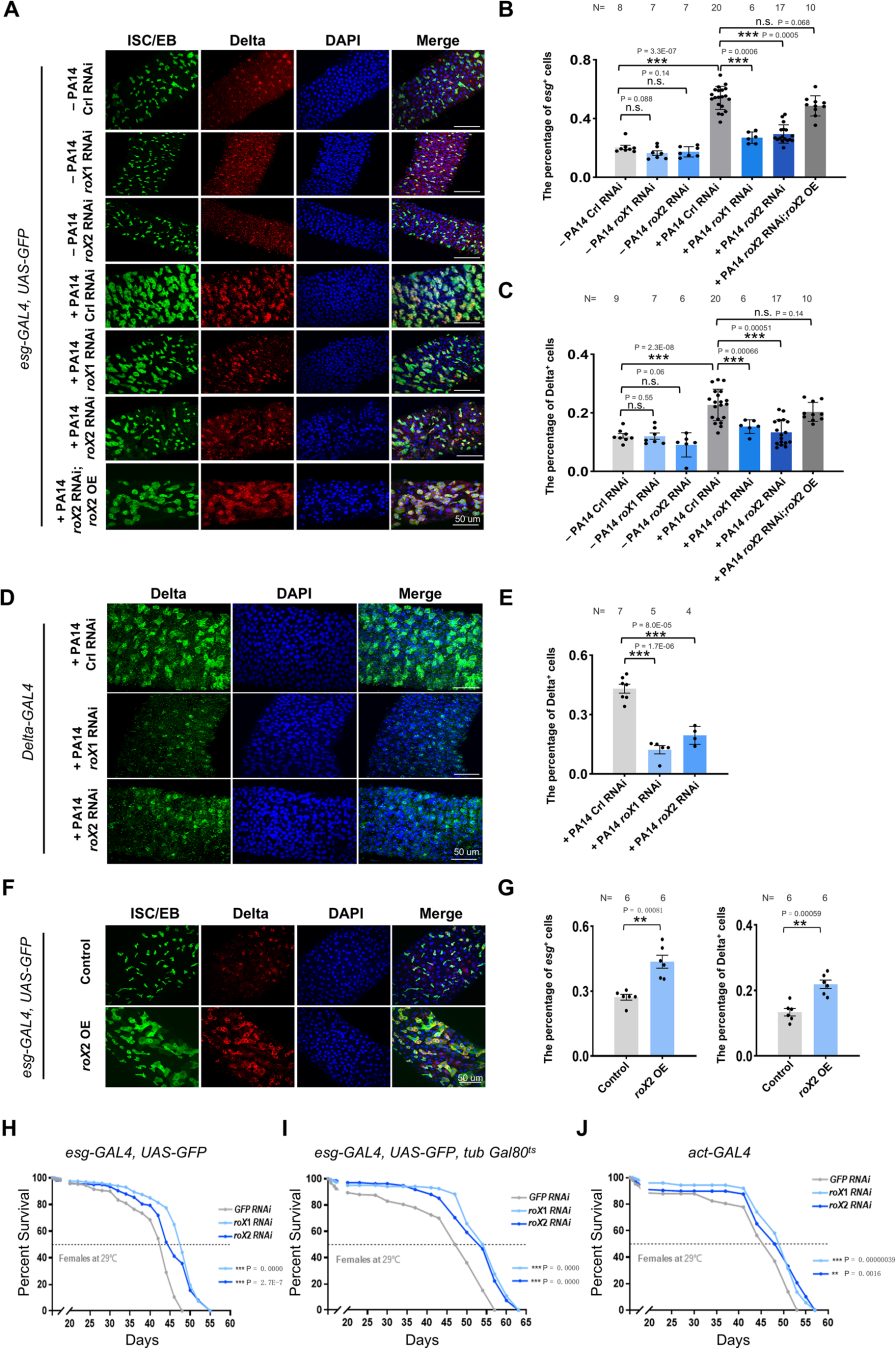
*roX* RNAs promote hyperplasia of ISCs and limit lifespan. (**A**) Images show the number/density of ISCs/EBs (GFP, green) and ISCs in the posterior midguts (Delta antibody recognizing ISC, red), either with (lower two panels) or without PA14 treatment (upper two panels). *roX*1 or *roX*2 RNAi is driven by *esg-Gal4, UAS-GFP*. Luciferase RNAi served as the control, expressed using the identical driver system. (**B**) Statistical analyses of the proportion of ISCs/EBs in (**A**). Data are from three independent biological replicates. The number of fields of view analyzed is indicated in the figure. (**C**) Statistical analyses of the ratio of ISCs in (**A**). Data are from three independent biological replicates. The number of microscopic fields analyzed is indicated in the figure. (**D**) The number of ISCs (green) in the posterior midguts after *roX*1 or *roX*2 RNAi via *Delta-Gal4* under PA14 infection. Delta marks the ISCs. (**E**) Quantification of the proportion of ISCs in the posterior midgut from (**D**). Data are from three independent biological replicates. The number of fields of view analyzed is indicated in the figure. (**F**) Ectopic expression of *roX*2 promotes hyperproliferation of ISCs in the posterior midguts. (**G**) Quantification of the proportion of ISCs in the posterior midgut from (**F**). Data are from three independent biological replicates. The number of microscopic fields analyzed is indicated in the figure. (**H**) Maximal and median longevity of females with RNAi against control *GFP*, *roX*1, or *roX*2 by *esg-Gal4, UAS-GFP*; the dotted line indicates the median longevity (*N* ≥ 3, *n* = 200 flies for each group). (**I**) Maximal and median longevity of females with RNAi against control *GFP*, *roX*1, or *roX*2 specifically during adulthood driven by *esg-Gal*4*; tub-Gal*80^*ts*^ under 29 °C; the dotted line indicates the median longevity (*N* ≥ 3, *n* = 200 flies for each group). (**J**) The maximal and median longevity of females after RNAi against control *GFP*, *roX*1, or *roX*2 by *act-Gal*4; the dotted line indicates the median longevity (*N* ≥ 3, *n* = 200 flies for each group). The center values are the averages, and the error bars indicate the s.e.m. *P* values were obtained by two-tailed unpaired Student’s *t* test. log-rank *t* test was used to calculate statistical differences of lifespan data. n.s., not significant, *P* ≥ 0.05, *P < 0.05, **P < 0.01, ***P < 0.0001. Scale bar, 50 μm. Source data are available online for this figure.

To examine whether the upregulation of *roX* RNAs correlates with the hyperplasia of female ISCs/EBs, we conditionally depleted *roX* RNAs using RNAi. Under normal culture conditions, the number of Delta-positive ISCs following *roX*1 or *roX*2 depletion remained similar to that in the controls (Fig. 5A–C). Interestingly, the *roX*1 or *roX*2 depletion causes both the number of Delta-positive ISCs and GFP-labeled ISCs/EBs to be decreased to half of those observed under the pathogen-infected condition (Fig. 5A–C), along with a significant decrease in mitotic ISCs (Appendix Fig. S10A,B), suggesting that *roX*1 or *roX*2 is required for the hyperproliferation of ISCs/EBs induced by bacterial pathogens. Moreover, specific depletion of *roX*1 or *roX*2 in ISCs using a *Delta-Gal4* driver significantly decreased the number of both ISCs and mitotic ISCs after PA14 infection (Fig. 5D,E; Appendix Fig. S10C,D), further confirming the essential role of *roX* RNAs in ISCs hyperplasia. Conversely, ectopic expression of *roX* RNAs alone under normal culture conditions efficiently promoted the hyperplasia of ISC/EBs (Fig. 5F,G; Appendix Fig. S10E,F). Furthermore, we performed a rescue assay by overexpressing *roX*2 in the *roX*2 RNAi background to test whether it could restore ISC hyperplasia. The increase of *roX*2 RNA was confirmed by qPCR (Appendix Fig. S11A). As we expected, the overexpression of *roX*2 successfully rescued the phenotype, which reduced the number of ISCs caused by *roX*2 depletion under PA14 treatment (Fig. 5A–C; Appendix Fig. S11C), further supporting the intrinsic requirement of *roX* RNAs for ISCs hyperplasia.

A molecular microenvironment that promotes a healthy gut during aging is expected to be beneficial for animal longevity (Onuma et al, 2023). Given that excessive ISC proliferation associated with inflammatory bowel disease can lead to a reduced lifespan in metazoans (Biteau et al, 2010), we asked whether attenuating the hyperproliferation of ISCs/EBs by *roX*1 or *roX*2 RNAi could potentially extend longevity. As shown in Fig. 5H, specific depletion of *roX*1 or *roX*2 in ISCs/EBs using *esg-Gal4* driver at 29 °C in female flies results in a 10–20% increase in both median and maximal lifespan compared to the GFP-RNAi control. To rule out any potential early developmental defects of *roX*1 or *roX*2, we conditionally depleted *roX*1 or *roX*2 transcripts in adult females using *esg-Gal*4 combined with *tub-Gal*80^*ts*^. Again, reducing *roX* RNAs expression in ISCs/EBs significantly prolonged the lifespan (Fig. 5I), supporting the essential role of *roX* RNAs involved in intestinal plasticity for longevity. Furthermore, we investigated the overall effect of *roX*1 or *roX*2 on animal longevity by depleting *roX*1 or *roX*2 transcripts through *act-Gal*4*/Cyo,ubi-GFP* driver, which is ubiquitously expressed in all cells. Remarkably, the ubiquitous reduction of *roX*1 or *roX*2 in adult females also extended their lifespan (Fig. 5J), but it did not affect the lifespan of males (Appendix Fig. S12). The results show that *roX* RNAs specifically regulate the lifespan of *Drosophila* females.

### *roX* RNAs promote heterochromatinization by recruiting HP1a and Su(var)3-9

**Figure 6:**
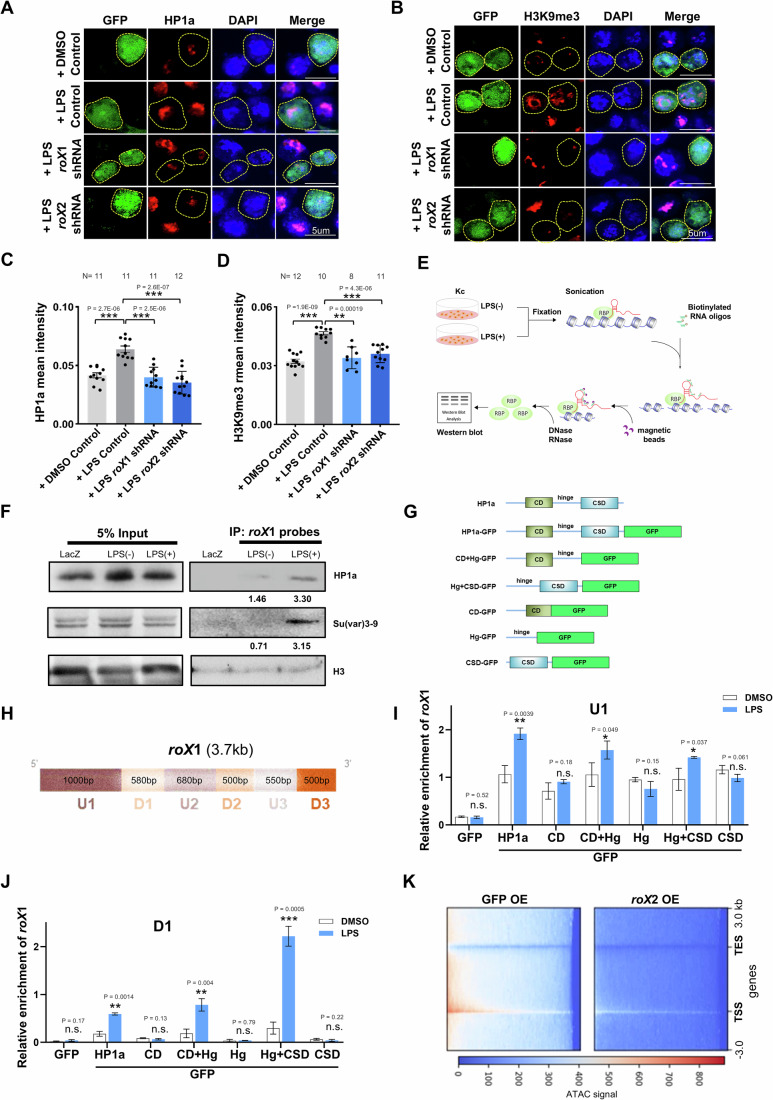
*roX* RNAs associate with heterochromatin proteins by specific RNA domains. (**A**) The association of HP1a (red) on chromatin in Kc cells with or without LPS treatment; the treated Kc cells were specifically depleted of *roX*1 or *roX*2 by shRNA. Upper two panels, control vector expresses only GFP. Lower two panels, vector expresses both GFP and shRNAs against *roX*1 or *roX*2. RNAi cells are marked by GFP (dotted circle). (**B**) Antibody staining showing the intensity of H3K9me3 (red) on chromatin in Kc cells under the same conditions as in A. GFP (dotted circle) labels the transfected cells. (**C**) Quantitation of the intensity of HP1a under the different conditions described in (**A**). Data are from three independent biological replicates. The number of fields of view analyzed is indicated in the figure. (**D**) Quantitation of the intensity of H3K9me3 under the different conditions shown in (**B**). Data are from three independent biological replicates. The number of fields of view analyzed is indicated in the figure. (**E**) The experimental strategy of *roX*1 RNA pull-down. (**F**) Western blot showing the results from *roX*1 RNA pull-down. Biotinylated *roX*1-complementary oligonucleotide probes were precipitated with HP1a and Su(var)3-9, and biotinylated *lacZ* probes were used as a control. The number below the blots indicates the intensity of HP1a and Su(var)3-9 enrichment relative to their input. H3 was used as a loading control (*N* = 3). (**G**) The graphic HP1a truncations fused with GFP. (**H**) The graphic RNA domains within the 3.7 kb of *roX1. roX*1 has six domains, three are D domains (D1, D2, and D3) and the other three are intervening U domains (U1, U2, and U3). The size of each domain is shown. (**I**, **J**) RIP-crosslink coupled with Q-PCR experiments show CD+Hg and Hg+CSD specifically interact with U1(I) and D1(J) domains of *roX*1 (*N* = 3). (**K**) ATAC heatmap shows that after *roX*2 OE treatment, the chromatin accessibility has decreased. Each line in the heatmap represents a transcript; the color is normalized ATAC level. Display range is TSS-3k to TES+3k; 20 bp window. The center values are the averages, and the error bars indicate the s.e.m. *P* values were obtained by two-tailed unpaired Student’s *t* test. n.s., not significant, *P* ≥ 0.05, *P < 0.05, **P < 0.01, ***P < 0.0001. Scale bar, 5 μm. Source data are available online for this figure.

**Figure EV3:**
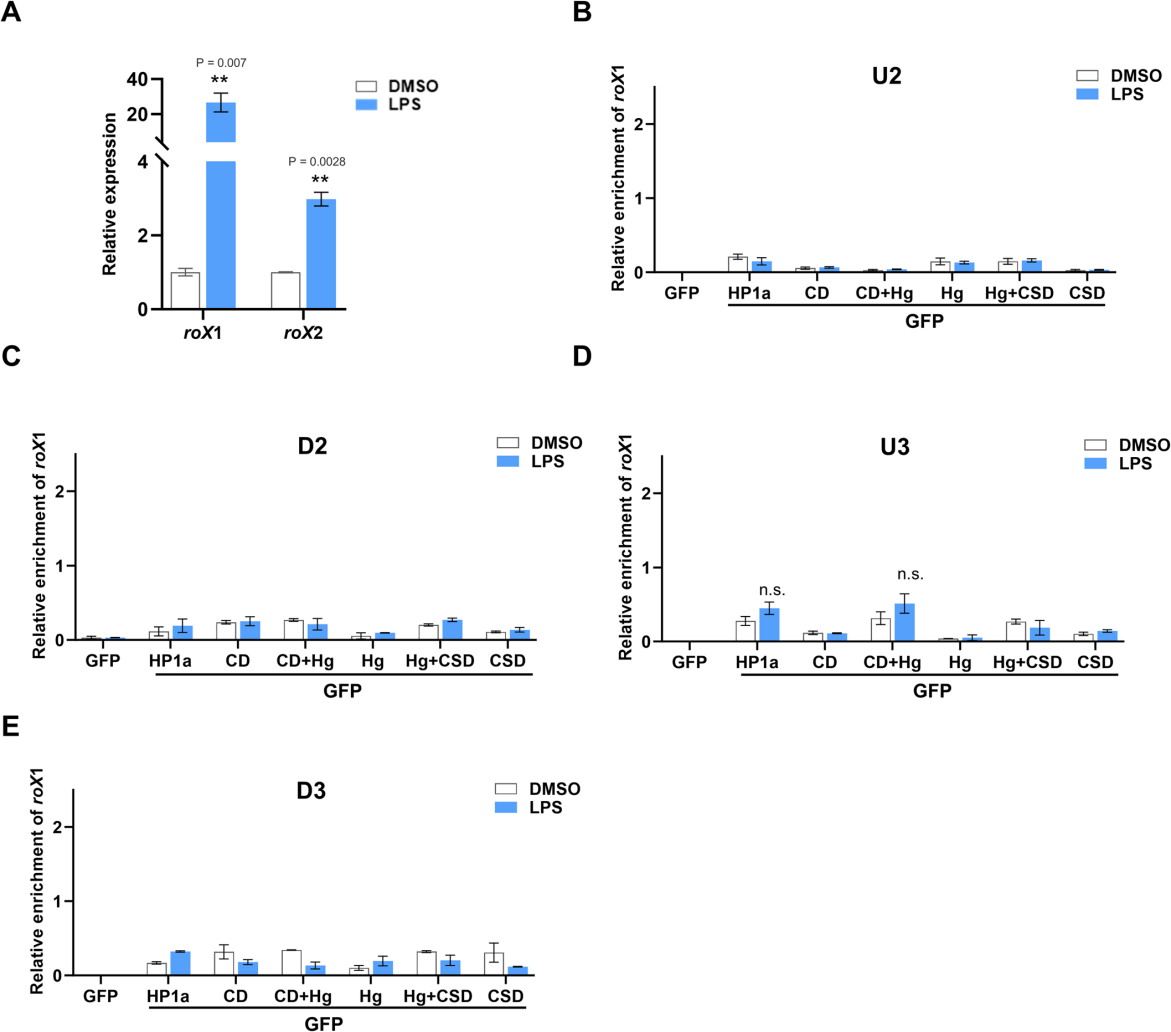
Other regions in the *roX*1 did not participate in the interaction with the heterochromatin protein. (**A**) LPS treatment promotes *roX* RNAs expression compared to the control. *N* = 3. (**B**–**E**) RIP-crosslink coupled with Q-PCR experiments show no interaction between different domains of HP1a and U2, D2, U3, and D3 of *roX*1. *N* = 3. The center values are the averages, and the error bars indicate the s.e.m. *P* values were obtained by two-tailed unpaired Student’s *t* test. n.s., not significant, *P* ≥ 0.05, *P < 0.05, **P < 0.01, ***P < 0.0001.

The limited number of ISCs/EBs poses challenges for further study into the molecular mechanisms of *roX* RNAs in heterochromatinization via biochemical approaches. Thus, we tested whether *roX*1 or *roX*2 RNA-mediated heterochromatinization also occurs in other female cell types. Initially, we performed a cytological assay using cultured *Drosophila* female embryonic Kc cells treated with lipopolysaccharide (LPS), a potent innate immune-activating stimulus from Gram-negative anaerobic bacteria, including PA14 (Simpson and Trent, 2019), to mimic bacterial pathogen-infected ISCs. Remarkably, treatment of Kc cells with LPS significantly elevates HP1a and H3K9me3 expression along with the increased *roX* RNAs expression based on qRT-PCR analysis (Figs. 6A–D and  EV3A), suggesting that cultured Kc cells can recapitulate the acquired heterochromatinization events observed in ISCs/EBs. Notably, *roX*1 or *roX*2 knockdown (Appendix Fig. S7D,E) and double knockdown (Appendix Fig. S7G,H) can sufficiently reduce the expression levels of HP1a and H3K9me3, while double knockdown generated more severe effects with HP1a and H3K9me3 (Fig. 6A–D; Appendix Fig. S13A–C), indicating *roX* RNAs are required for heterochromatin formation and function redundantly. These results confirm that the acquired heterochromatinization in Kc cells is also dependent on *roX* RNAs as in the ISCs of the midgut.

These findings prompted us to explore the interaction between *roX* RNAs and heterochromatin proteins by performing *roX* RNA-based pull-down assays, also known as chromatin isolation by RNA purification using biotinylated complementary oligonucleotides of *roX*1 as bait and biotinylated complementary oligonucleotides of *lacZ* as the control (Fig. 6E). Interestingly, both HP1a and the H3K9 methyltransferase- Su(var)3-9 can be brought down from Kc cell lysate by the *roX*1 RNA but not by the control *LacZ* RNA, indicating physical interactions between *roX*1 and heterochromatin proteins. Moreover, more HP1a and Su(var)3-9 proteins can be pulled down by *roX1* after LPS treatment, suggesting that *roX* RNAs promote the expansion of heterochromatinization by recruiting key heterochromatin components (Fig. 6F). In addition, we also performed RNA immunoprecipitation with HP1a antibody followed by qPCR, a significant increase of *roX* RNAs were observed after LPS treatment (Appendix Fig. S14A,B), indicating enhanced association of HP1a with *roX* RNAs, further supporting the interaction between them. To map the domains and the RNA regions involved in the protein-RNA interactions, we performed RIP-crosslink coupled with qPCR experiments using chromatin extracts from Kc cells expressing GFP-tagged entire or subdomains of HP1a (Fig. 6G; Appendix Fig. S15), such as chromo domain (CD), hinge (Hg), and chromo shadow domain (CSD). Interestingly, the U1 and D1 regions of *roX*1 RNA (Fig. 6H), but not other regions, are associated with the HP1a heterochromatin complex (Figs. 6I,J and EV3B–E). Similarly, the fragments covering CD+Hg and Hg+CSD of HP1a mediated the interaction with *roX1* (Figs. 6I,J and EV3), and the interaction at the U1 or D1 region was also enhanced after LPS treatment (Fig. 6I,J).

Given *roX* RNAs are required for heterochromatin expansion in our cytological assays, we hypothesize that the increase of *roX* RNAs shall condense chromatin to reduce chromatin accessibility, to confirm our hypothesis, we analyzed chromatin accessibility by ATAC-seq after *roX*2 overexpression in Kc cells (Appendix Fig. S7F). Following analysis, in contrast to the control, we found a significant decrease in chromatin accessibility near the transcription start sites (TSS) of genes upon *roX*2 overexpression (Fig. 6K), supporting an elevated level of heterochromatinization and the association between *roX* RNAs and heterochromatin proteins. Taken together, the above results support the idea that *roX* RNAs promote heterochromatinization and epigenetic gene silencing by interacting with HP1a and Su(var)3-9.

### *roX* RNAs promote heterochromatinization in the salivary gland

**Figure 7:**
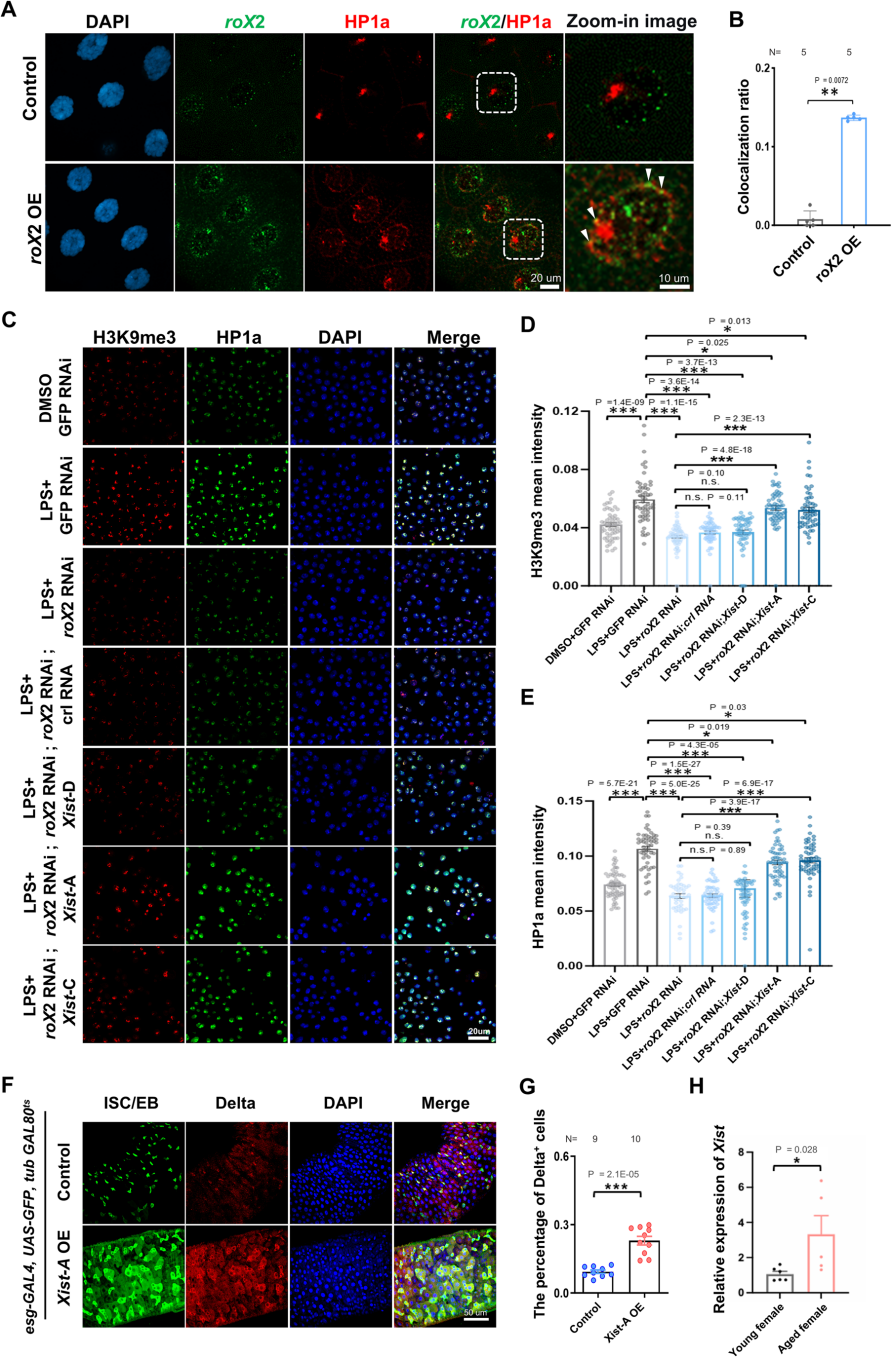
*Xist* RNA functions similarly to *roX* RNAs to promote heterochromatin expansion. (**A**) Ectopic expression of *roX*2 RNA drives heterochromatin expansion in salivary gland cells. The white boxes denote representative chromosome regions that are shown as enlarged views; the white arrows indicate co-localized signals. (**B**) The co-localization between *roX*2 and HP1a was quantified using the co-localization ratio (Pearson’s correlation coefficient). The number of biological replicates (N) is indicated on the graph. (**C**) In contrast to control RNA (ctrl RNA) or *Xist-D*, the expression of *Xist-A* and *Xist-C* restored heterochromatin levels reduced by *roX*2 RNAi (*roX*2 dsRNA). We designed a scrambled RNA sequence as a control. This was generated by randomizing the nucleotide sequence of *Xist-A* fragment using an online algorithm (molbiotool’s Random Sequence Generator), which preserved the identical nucleotide composition while abolishing any potential biological sequence specificity. (**D**, **E**) Quantification of the H3K9me3 (**D**) and HP1a (**E**) under different conditions (*N* = 3, *n* ≥ 50 cells). (**F**, **G**) Conditional expression of *Xist-A* in adult females induces ISC hyperplasia. Representative images in (**F**) and quantified in (**G**). Data are from three independent biological replicates. The number of fields of view analyzed is indicated in the figure. (**H**) The relative transcriptional level of human *Xist* RNA in young and aged women. (*N* = 3). The center values are the averages, and the error bars indicate the s.e.m. *P* values were obtained by two-tailed unpaired Student’s *t* tests. n.s., not significant, *P* ≥ 0.05, *P < 0.05, **P < 0.01, ***P < 0.0001. Scale bar, 20 μm. Source data are available online for this figure.

**Figure EV4:**
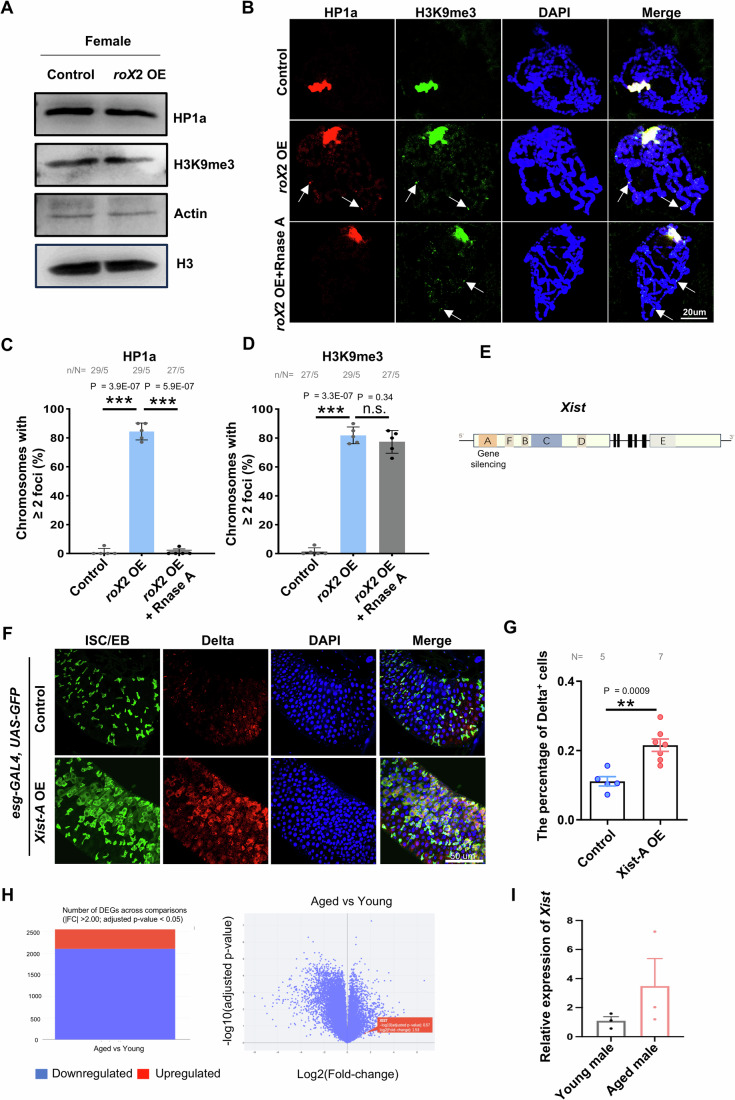
Ectopic expression of *roX*2 promotes the heterochromatinization. (**A**) Western blot shows no change in HP1a and H3K9me3 after *roX*2 overexpression. (**B**) The polytene chromosome staining shows the spreading of HP1a and H3K9me3 after *roX*2 overexpression, and the increase of HP1a is sensitive to RNaseA treatment. The arrow indicates the site of HP1a or H3K9me3 spreading. (**C**, **D**) Quantification of the percentage of chromosomes with HP1a (**C**) or H3K9me3 (**D**) spreading in (**B**). The observation of two or more HP1a or H3K9me3 signal foci on salivary gland chromosomes is defined as spreading. *N* = 5, *n* ≥ 27 Chromosomes. (**E**) The different regions of *Xist* RNA in humans. (**F**) IF showing the ISCs/EBs (GFP, green) and ISCs (indicated by Delta antibody, red) in control *Drosophila* (upper panel) and *Xist-A* overexpression *Drosophila* driven by *esg-GAL4 UAS-GFP* (lower panel). (**G**) The percentage of ISCs in (**F**) was quantified. Data are from three independent biological replicates. The number of microscopic fields analyzed is indicated in the figure. (**H**) The column plot for the differentially expressed genes (left) and volcano plot for the details (right) from the previous RNA-seq data of human endothelial cells in young and aged people, the red dot indicates the *Xist*. (**I**) The relative expression level of *Xist* RNA in men (*N* = 3). *P* value was obtained by a two-tailed unpaired Student’s *t* test. n.s., not significant, *P *≥ 0.05, *P < 0.05, **P < 0.01, ***P < 0.0001. Scale bar, 20 μm or 50 μm.

The *Drosophila* salivary gland cell serves as an excellent model for examining the localization of transcriptional factors along polytene chromatin. To validate the physical interaction between *roX* RNAs and heterochromatin proteins, we performed hybridization chain reaction RNA fluorescent in situ hybridization (HCR RNA-FISH) coupled with antibody staining against HP1a to observe the co-localization of *roX*2 with HP1a on the chromatin in female salivary gland cells. Overexpression of *roX*2 by *1824-GAL4* sufficiently caused the expansion of heterochromatin, evidenced by enhanced HP1a enrichment, and HP1a was also co-localized with the *roX2* RNA (Fig. 7A,B). Notably, the increase in heterochromatinization is not caused by the increased expression of heterochromatin proteins based on Western blot results (Fig. EV4A). To better visualize the expansion of heterochromatin, we performed polytene chromosome staining after *roX*2 overexpression. In contrast to the control, the percentage of heterochromatin foci marked by either HP1a or H3K9me3 was increased in the euchromatic arms of the polytene chromosomes (Fig. EV4B–D). Interestingly, following RNase A treatment, the HP1a foci on the euchromatic arms can be reverted to the levels of the control without a noticeable reduction in H3K9me3, suggesting the dominant role of *roX*2 RNA in the spreading of HP1a on the euchromatin (Fig. EV4B–D). These results indicate that the *roX*2 RNA promotes heterochromatinization by recruiting heterochromatin proteins.

### *Xist* RNA functions similarly to *roX* RNAs in Kc cells to promote heterochromatinization

Given that the overexpression of *roX*1 or *roX*2 RNA acts as a transcriptional repressor to facilitate heterochromatin spreading in female flies under stress conditions, resembling the role of *Xist*/*XIST* RNA in humans, a long noncoding RNA necessary for X-chromosome-linked gene inactivation (Brockdorff et al, 2020; Colognori et al, 2019; Dossin and Heard, 2021). We next asked whether *Xist* RNA functions similarly to *roX* RNAs, then rescue experiments were performed to determine whether *Xist* RNA can rescue the *roX* RNAs knockdown-generated phenotype. First, overexpression of *roX*2 in a *roX*2 RNAi background successfully restored the reduced heterochromatinization caused by knockdown (Appendix Fig. S13A–C), suggesting the rescue experiments work well. Using this rescue system, different regions of *Xist* RNA were overexpressed (Fig. EV4E), with a scrambled RNA as a negative control (Appendix Fig. S7I–L). Compared with LPS-treated control, knockdown of *roX*2 resulted in a reduction in heterochromatinization, marked by H3K9me3 and HP1a, to the level of non-LPS-treated controls (Fig. 7C–E). Interestingly, expression of the region A (*Xist*-A) and the region C (*Xist*-C), two regions in *Xist* RNA involved in PcG recruitment or gene silencing (Sarma et al, 2014; Colognori et al, 2020), can significantly reverse the reduction of heterochromatin in Kc cells after depletion of *roX* RNAs under LPS treatment. However, similar to the control RNA, the region D exhibited no obvious effect on the rescue (Fig. 7C–E). Most interestingly, elevation of *Xist*-A alone by *esg*-Gal4/tub-Gal80, also promoted ISCs hyperplasia during adulthood (Figs. 7F,G and  EV4F,G). These results suggest that mammalian *Xist* RNA functions similarly to *roX* RNAs in promoting *Drosophila* heterochromatin formation and stem cell hyperplasia. With the previously published gene expression dataset from endothelial cells (Drekolia et al, 2022), we performed data analysis. Interestingly, we found that the majority of affected genes due to aging were downregulated, whereas the transcription of *Xist* RNA appeared to increase (Fig. EV4H). To confirm this result, we detected the *Xist* RNA from the blood of young and aged people by qRT-PCR, strikingly, in contrast to the young people, the *Xist* RNA significantly increased in aged people, which is similar to the phenomenon from flies (Figs. 7H and  EV4I). Altogether, the above results support that the role of *roX* RNAs in heterochromatinization is functionally analogous to that of *Xist* in humans.

## Discussion

**Figure 8:**
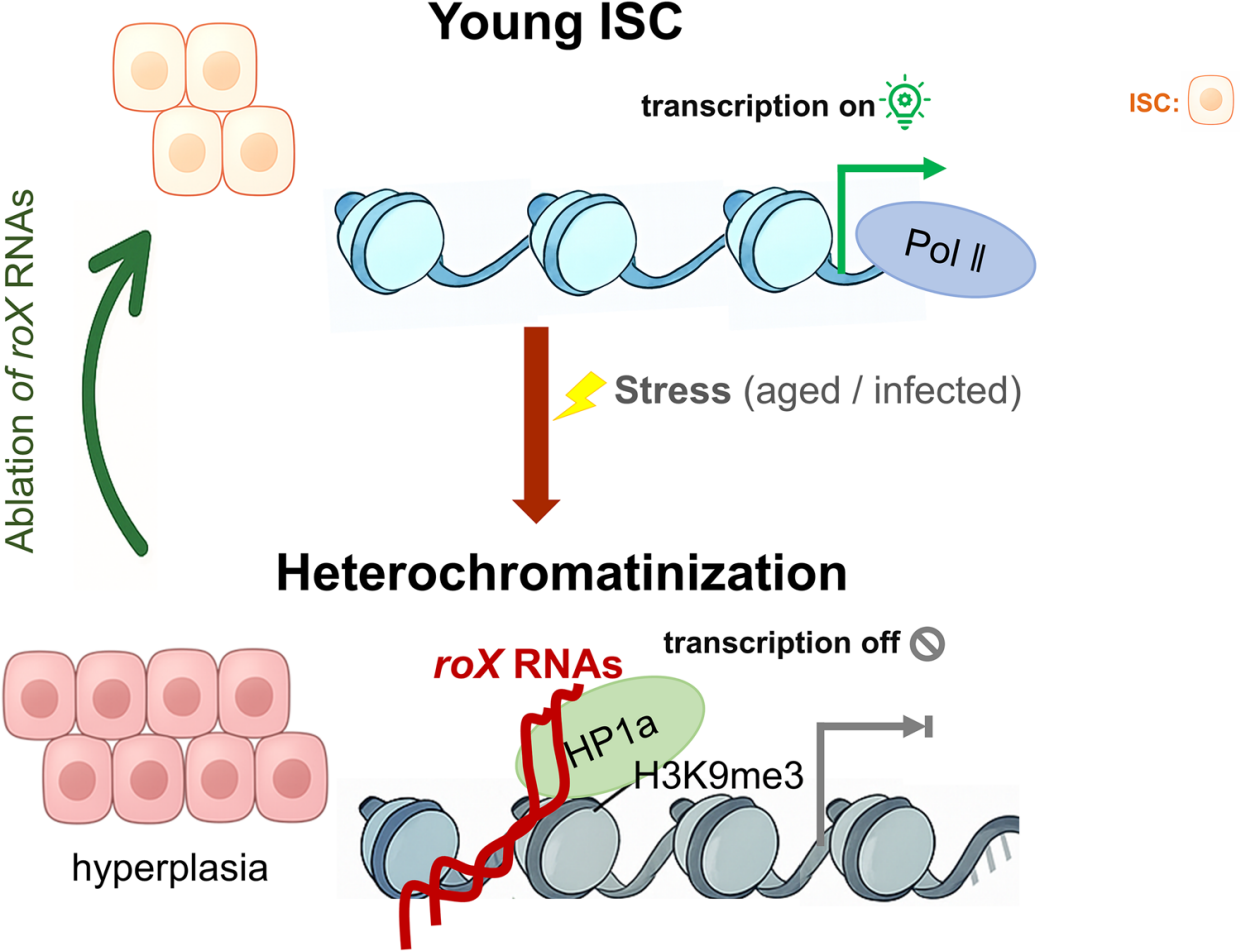
A simple graphical model for *roX* RNA in regulating heterochromatin dynamics and intestinal homeostasis during aging. In healthy young females, the maintenance of homeostasis by ISCs/EBs is characterized by balanced gene transcription and appropriate cell numbers. Upon aging or infection stress, the expression of *roX* RNA is induced, primarily in ISCs. These *roX* RNA promote the recruitment and stabilization of HP1a onto chromatin, facilitating the formation of heterochromatin. This results in transcriptional silencing and ISC/EB hyperplasia. Strikingly, ablation of *roX* RNA triggers the dissociation of HP1a from chromatin, which restores transcription and ISC/EB homeostatic proliferation.

Eukaryotic ASCs display sophisticated adaptability to their microenvironment through epigenomic modifications. Under stressed conditions such as pathogen infection and aging, a sophisticated response mechanism is activated in ISCs in the female midgut to cause hyperplasia, thereby shortening the organismal lifespan. However, the underlying cause remains poorly defined. This study has identified the elevation of *roX* RNAs in ISCs as a key driver for promoting heterochromatin expansion, ISC hyperplasia, and shortening lifespan. This expansion is facilitated by the physical interaction of *roX* RNAs with heterochromatin proteins, effectively silencing target gene transcription, including the genes involved in Notch signaling. The resulting hyperplasia of ISCs (Figs. EV1A and 2A) is deleterious to gut health and reduces the female lifespan. The depletion of *roX* RNA triggers the disassociation of HP1a from chromatin and eventually restores both gene transcription and the number of ISCs (Fig. 8). Therefore, this study reveals a novel role of *roX* RNAs in transcriptional repression to regulate the ISC fate and lifespan of animals.

Our cytological, genetic, and biochemical analyses, however, reveal unexpected roles of *roX*1 or *roX*2 in heterochromatin formation, epigenetic silencing, and ISC hyperplasia in females under the aging condition or pathogen treatment. Elevated levels of *roX* RNAs interact with heterochromatin proteins to orchestrate heterochromatin formation. This contrasts with the well-known function of *roX* RNAs in male *Drosophila*, where they regulate X chromosome dosage compensation (Meller et al, 1997; Samata and Akhtar, 2018). In male flies, *roX* RNAs interacts with the male-specific lethal (MSL) complex to upregulate the transcription of genes on the X chromosome, thereby equalizing the transcription of X-chromosome genes from the two X chromosomes in females (Akhtar and Becker, 2000; Bashaw and Baker, 1995; Belote and Lucchesi, 1980; Deng and Meller, 2006; Hilfiker, 1997; Kelley et al, 1995, 1997; Conrad et al, 2012). However, the function of *roX* RNAs in female flies was overlooked due to its relatively low expression and lack of developmental effects (Franke and Baker, 1999; Meller, 2002). Previous studies have revealed the non-canonical function for autosomal gene repression of *roX* RNA (Li et al, 2025; Deng et al, 2009; Koya and Meller, 2015), and our work significantly extends this understanding by revealing that *roX* RNAs interact with heterochromatin proteins to promote heterochromatin formation. Specifically, under conditions of infection or aging in females, the increase of *roX* RNAs triggers broad gene repression, destroys intestinal homeostasis, and impacts flies' lifespan. Through *roX* RNAs pull-down assay and RIP-crosslink coupled with qPCR experiments, we observed the interaction between *roX* RNAs and HP1a, a key component of heterochromatin. Specifically, regions U1 and D1 of *roX*1 RNA interacted with the CD+Hg or Hg+CSD domains of HP1a. RNA-FISH coupled with antibody staining on the polytene chromosomes further validated their interaction. Our findings also reveal the involvement of *roX* RNAs in newly acquired heterochromatinization. The distinct mechanism by which *roX* RNAs function in male and female flies is unknown. One of the potential reasons is that the presence of active chromatin regulators, such as MSLs and MOF, in males may limit *roX* RNAs association with heterochromatin through competition. Conversely, the absence of functional MSL in females may facilitate the association of HP1a and Su(var)3-9 with *roX* RNAs, thereby promoting heterochromatinization and gene silencing. Based on our findings, a model was proposed, *roX* RNAs act as multifacial noncoding RNAs whose functions depend on specific conditions, including sex and physiological stress. In males, *roX* RNAs primarily function in dosage compensation to activate gene expression. In females, under normal conditions, *roX* RNAs are expressed at relatively low levels; however, under stress such as infection or aging, the expression is activated, where they play a transcriptional repression via heterochromatinization. While stress-induced heterochromatin expansion occurs in both males and females, *roX* upregulation was observed only in females (Appendix Fig. S6). In males, basal levels of *roX* may be sufficient to support heterochromatin expansion upon infection. Alternatively, other *roX*-independent pathways may exist that respond to stress and induce heterochromatin expansion. This uncoupling between *roX* RNA upregulation and heterochromatinization suggests that *roX* RNAs are not the only drivers of stress-induced heterochromatinization. Further investigation is necessary to elucidate the molecular mechanisms involved.

In mammals, X chromosome gene inactivation relies on X-inactive specific transcript (*Xist*), which acts as a repressor of X chromosome gene transcription by interacting with the PcG group (Robert Finestra and Gribnau, 2017). Consistent with the competition model, the regions of *Xist* important for its association with PcG proteins can sufficiently replace the function of *roX*1 or *roX*2 in promoting heterochromatin formation in *Drosophila* cells. With cytological analysis, conditionally ectopic overexpression of *Xist* RNA also promoted ISC hyperproliferation in *Drosophila*. Interestingly, similar to the elevation of *roX* RNAs in aged *Drosophila*, *Xist* RNA also significantly increased in the elder people, further indicating its coordination with aging. We extracted total RNA from entire blood, in this case, we can not avoid the byproduct from dead cells, to increase the accuracy, future experiments shall use the blood cells which removed the plasma after centrifugation.

The observation that increased *roX* expression is detected in only a subset of Delta⁺ ISCs (~15–20%) upon stress raises the question: increased *roX* expression in a relatively small fraction of ISCs could account for the broader heterochromatin expansion observed across the ISC population? One possibility is that the heterochromatin state established in *roX*-positive ISCs may be inherited by their progeny during cell division, leading to the spread of heterochromatinization across the ISC population.

*roX*1 or *roX*2 is preferentially expressed in ISCs, so why do EBs show comparable heterochromatinization? One explanation is that EBs retain ISC-derived heterochromatin proteins after differentiation. Another possibility is that cytokines or other inflammatory signals from the local microenvironment could activate pathways, such as JAK/STAT, to induce the expression of epigenetic modifiers that promote heterochromatin formation on EBs. Finally, the possibility that other conserved stress-responsive factors, such as histone methyltransferases, might be activated in EBs in response to aging or infection to drive heterochromatinization.

The ubiquitous depletion of either *roX*1 or *roX*2 promotes sex-biased animal longevity. Previous studies have shown that deleting both *roX*1 and *roX*2 resulted in male-specific lethality, while depleting either *roX*1 or *roX*2 showed no visible adult phenotypes (Franke and Baker, 1999; Robert Finestra and Gribnau, 2017), but whether the loss of *roX*1 or *roX*2 RNA influences animal lifespan remains unexplored. We demonstrate here that the pan-depletion of *roX*1 or *roX*2 in flies using an *act*-driver (Figs. 5J and  EV4A) specifically increases the lifespan of females, but not males, which is in line with the no expression change under stress conditions in males, further supporting a sex-biased role for *roX*1 or *roX*2 in chromatin architecture and animal longevity. Considering that *roX* RNAs are highly expressed in wild-type male flies, which have a considerably shorter lifespan than females, the high expression levels of *roX* RNAs in males may contribute to the shorter lifespan of males. The observed lack of change in the lifespan of *roX*1 or *roX*2 knockdown males could be due to knockdown efficiency or no roles in regulating heterochromatin formation in males. Altogether, our study reveals a novel role of *roX* RNAs in female *Drosophila*, and further studies are warranted to elucidate the molecular mechanism through which *roX* RNAs differentially modulate chromatin architecture and gene regulation in a sex-biased manner.

**Figure EV5:**
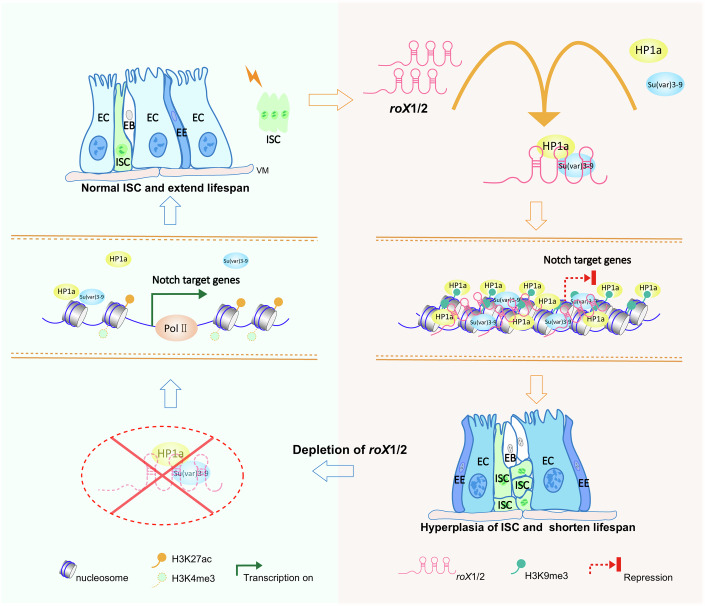
Schematic for the present study. Under stressed conditions, such as pathogen treatment or aging, the increase of *roX* RNAs associated with heterochromatin proteins then guides heterochromatin spreading to target essential genes of ISC homeostasis. If it reduces the levels of *roX* RNAs, then it prevents heterochromatinization, releases the inhibition on genes of ISC homeostasis, and eventually extends the animal lifespan.

Lastly, it is noteworthy that aging and intestinal stress caused by bacterial pathogens in *Drosophila* ISCs induce similar ISC hyperplasia and expansion of heterochromatin, resulting in the modulation or silencing of the same set of genes in transcription. This suggests a direct link between intestinal exposure to microbial stresses and the aging-coupled hyperplasia of ISCs. For example, the accumulation of pathogenic bacteria or microbial dysbiosis leads to increased heterochromatinization of Notch pathway genes, resulting in impaired differentiation and aberrant expansion of ISC-like cells (Fig. EV5). This disruption of intestinal homeostasis eventually leads to or accelerates the aging process. Our study highlights heterochromatinization as a key determinant of animal longevity, consistent with findings in *C. elegans* and mammals (Huang et al, 2022; Yang et al, 2023). Taken together, this study has uncovered an unexpected role of *roX* RNAs in regulating heterochromatinization and controlling longevity.

## Methods

**Table:** Reagents and tools table

Reagent/resource	Reference or source	Identifier or catalog number
***Drosophila melanogaster*** **strains**		
y w; *esg-Gal4 UAS-GFP/Cyo*(esg-Gal4)	TsingHua Fly Center	TB00044
y w; *esg-*Gal4*, UAS-GFP, tub Gal80ts* (*esg, Gal-80*)	TsingHua Fly Center	TB00137
*delta-*GAL4*/Cyo*	TsingHua Fly Center	Ni lab
*actin 5 C* Gal4/ Cyo, Ubi-GFP	TsingHua Fly Center	TB00047
1824*-*GAL4	TsingHua Fly Center	Ni lab
UAS-*roX2* OE	TsingHua Fly Center	TH15406.S
UAS-*roX2* OE	TsingHua Fly Center	TH15405.S
UAS-*GFP* OE	TsingHua Fly Center	TH10512.S
UAS-*GFP* RNAi	TsingHua Fly Center	TH00781.N
UAS-*Luciferase* RNAi	TsingHua Fly Center	Ni lab
UAS-*roX1* RNAi	TsingHua Fly Center	THU1906
UAS-*roX2* RNAi	TsingHua Fly Center	THU1961
UAS-*Xist A* OE	TsingHua Fly Center	Ni lab
U7: y w; Sco/CyO; Sb/TM6B	TsingHua Fly Center	Ni lab
UAS- *roX1* RNAi; *roX*2 OE	This study	N/A
**Cell lines**		
KC167 cells (*D. melanogaster*)	Drosophila Genomics Resource Center	FBtc0000001
**Recombinant DNA**		
pOplE2-HP1a-GFP	This study	N/A
pOplE2-CD-GFP	This study	N/A
pOplE2-Hg-GFP	This study	N/A
pOplE2-CSD-GFP	This study	N/A
pOplE2-CD-Hg-GFP	This study	N/A
pOplE2-Hg-CSD-GFP	This study	N/A
pOplE2-A(Xist)	This study	N/A
pOplE2-C(Xist)	This study	N/A
pOplE2-D(Xist)	This study	N/A
pOplE2-*roX*2	This study	N/A
**Antibodies**		
Mouse anti-HP1a	DSHB	C1A9
Rabbit anti-HP1a	From Sarah Eglin lab	N/A
Rabbit anti-H3K4me3	Abcam	ab8580
Rabbit anti-H3K9me3	Abcam	ab8898
Rabbit anti-H3K27ac	Abcam	ab4729
Mouse anti-Delta	DSHB	C594.9B-s
Rabbit anti-PolIIser2	Abcam	ab5989
Rabbit anti-GFP	Abcam	ab6556
Mouse anti-GFP	Abcam	Ab1218
Chicken anti-GFP	Aves	GFP-1020
Rabbit anti-PH3	Abcam	Ab5176
Alexa Fluor 555-conjugated donkey anti-rabbit	Abcam	ab150154
Alexa Fluor 555-conjugated donkey anti-mouse	Abcam	ab150106
Alexa Fluor 488 conjugated donkey anti-rabbit	Abcam	ab150073
Alexa Fluor 488 conjugated goat anti-chicken	Jackson ImmunoResearch Laboratories	703-545-155
Donkey Anti-Rabbit IgG H&L (Alexa Fluor® 647)	Abcam	ab150075
Rabbit anti-H3	Cell Signaling Technology	#9715
Mouse anti-actin	Santa Cruz Biotechnology	sc-47778
Rabbit anti-Su(var)3-9	Boster Bio	DZ33950
Goat Anti-Rabbit IgG (H + L) HRP	Affinity Biosciences	#S0001
Anti-mouse IgG, HRP-linked Antibody	Cell Signaling Technology	#7076
HRP-conjugated Rabbit anti-Chicken IgY (IgG) (H + L)	Abclonal	AS030
**Oligonucleotides and other sequence-based reagents**		
RT-qPCR primers	This study	Table EV1
Cloning primers	This study	Table EV1
dsRNA primers	This study	Table EV1
Oligonucleotides for *roX1* probes	This study	Table EV1
**Chemicals, enzymes, and other reagents**		
LPS	Sigma	#2369
Streptavidin magnetic C1 beads	Invitrogen	#65001
HiScribe T7 High Yield RNA Synthesis Kit	NEB	E2040S
cellfectin	Invitrogen	10362100
**Software**		
Image J	Image J Software	https://imagej.nih.gov/ij/
GraphPad Prism	GraphPad Software	https://www.graphpad.com
oasis2 software	https://sbi.postech.ac.kr/oasis2/	N/A
Cellranger v3.0.2 and v6.0.2	https://support.10xgenomics.com/single-cell-gene-expression/software/overview/welcome	N/A
Scanpy v1.5.1	https://scanpy.readthedocs.io/en/stable	N/A
Python v3.7.3	https://www.python.org/	N/A
R v4.0.3	https://www.r-project.org/	N/A
Seurat v4.0.0	https://satijalab.org/seurat	N/A
SeuratDisk v0.0.0.9013	https://mojaveazure.github.io/seurat-disk/,	N/A
MAST v1.16.0	https://github.com/RGLab/MAST/	N/A
ggplot2 v3.3.5	https://github.com/tidyverse/ggplot2	N/A
ggpubr v0.4.	https://CRAN.R-project.org/package=ggpubr, https://rpkgs.datanovia.com/ggpubr/	N/A
Venn Painter v1.2.0	https://github.com/linguoliang/VennPainter	N/A

### Ethical statement

The blood extraction from humans in this study was conducted in accordance with the ethical guidelines of the First Affiliated Hospital of Zhengzhou University Ethics Board (Approval Number: 2025-KY-0617-001).

### Cell culture

*Drosophila* Kc cells were cultured in Schneider’s *Drosophila* Medium (Gibco, 21720024) containing 10% FBS (Gibco, A5256701) and 1% penicillin/streptomycin (HYClone, SV30010) at 27 °C.

### *Drosophila* stocks and rearing

Flies were cultured in vials containing cornmeal medium (in wt/vol, 1% agar, 5.9% brewer’s yeast, 5.2% glucose, 2.6% brown sugar, and 5.9% cornmeal). The stocks were routinely maintained at 25 °C with 12/12 h dark/light cycles in humidity-controlled incubators, with the exception of those specifically mentioned. For the transgenic conditional expression systems, *y w; esg-Gal4, UAS-GFP, tub Gal*80^*ts*^ was used to drive UAS-linked transgenes. Flies were allowed to develop at 18 °C, and then the adult flies were transferred to 29 °C to induce gene expression. The UAS-*roX*2 flies, which are driven by the *esg-Gal4*, *UAS-GFP*, were cultured at 29 °C for 6 days before being utilized for the subsequent assessment of intestinal stem cell numbers. And, the *esg-Gal4*, *UAS-GFP* flies served as a control under identical culture conditions. *Drosophila* strains were listed in the Reagents and Tools Table.

### Bacterial infection

PA14 infection was performed according to a previously reported method (Limmer et al, 2011). In brief, the PA14 strain (gift from Dr. Wei Li’s lab) was cultured in LB medium overnight at 37 °C and collected by centrifugation. The bacterial culture was diluted with LB until the OD_600_ of the solution was 2.0, followed by diluting ten times with 5% sucrose water. This bacterial solution was then added to cotton balls in vials. The flies were fed on the cotton balls containing the bacteria for 6 days at 25 °C.

### Immunofluorescence (IF) staining

The guts of female flies were used in all IF experiments unless otherwise indicated. The intact adult guts were dissected in cold PBS, immediately fixed for 40 min in 4% formaldehyde (Sigma, F8775) at room temperature, and then washed three times with PBST (0.1% Triton X-100). For immunostaining with Delta antibody, guts were fixed for 30 min, washed twice with methanol for 10 min each time, and transferred to gradient methanol washing (66%, 50%, 33%) for 5 min each, followed by washing with PBST three times. The primary antibodies in 5% donkey serum were incubated at 4 °C overnight. After washing with PBST, the samples were incubated with secondary antibodies for 2 h at room temperature. DAPI was used to stain nuclei for 10 min at room temperature. A list of primary and secondary antibodies used in this study is provided in the Reagents and Tools Table. Finally, the samples were mounted and analyzed under a Nikon A1R confocal microscope. All images and analyzed data were obtained from the R5 (P4) region of the adult posterior midgut.

IF staining using Kc cells was performed as follows. Cells on coverslips were fixed with 4% formaldehyde for 10 min and washed three times with PBST. Samples were blocked with 5% BSA (Sigma, A1933) for 1 h and incubated with primary antibodies at 4 °C overnight. After washing with PBST, the samples were incubated with secondary antibodies for 2 h at room temperature. After washing with PBS once again, the samples were incubated with DAPI (Beyotime, C1002) for 10 min. Then, the samples were covered with mounting medium and analyzed using a Nikon A1R confocal microscope.

### Immunostaining of polytene chromosomes and RNase A treatment

Salivary glands were dissected and fixed in solution containing 1.85% formaldehyde and 45% acetic acid in water for 15 min. For RNase treatment, glands were incubated in PBS containing RNase A (Takara, 2158) (10 μg/ml) at room temperature for 30 min, followed by three washes with PBST. The squashed polytene chromosomes were incubated with the following antibodies: anti-HP1a, anti-H3K9me3. The slides were labeled with secondary antibodies and stained with DAPI. Polytene chromosomes were analyzed using a Zeiss LSM880 confocal microscope.

### Image processing and quantitative analysis

Images were captured utilizing a ×60 or ×100 objective lens on a confocal microscope. For IF quantification, the nuclear region was first defined based on the DAPI signal. The total fluorescence intensity within this region was measured after applying a consistent threshold in ImageJ. The mean fluorescence intensity (mean optical density) for each antibody was then calculated as the total intensity divided by the nuclear area. The percentage of Delta+ and *esg* + (GFP + ) cells was obtained by overlaying the images through scanning, and then calculating the ratio of the number of Delta+ or GFP+ cells to the number of DAPI-positive cells.

### Single-cell preparation

Approximately 200 adult female fly midguts were dissected in ice-cold PBS/1% BSA solution and transferred into a tube on ice. All samples were processed within 2 h. The solution was removed, and the samples were washed with PBS/1% BSA. Subsequently, 500 μl of 0.5% trypsin-EDTA (Gibco, 15400054) solution was added. The guts were cut with scissors, and the samples were vortexed and incubated by gently rotating at room temperature for 25 min. The samples were kept on ice for 5 min to allow any intact midguts to sink to the bottom of the tube. The cell suspension was carefully transferred into a fresh tube and filtered through a 40-µm nylon mesh. The strained cells were spun down at 100 × *g* for 5 min at 4 °C. The trypsin solution was then carefully transferred back to the original sample tube containing the remaining intact midgut tissue, and the cell pellet was gently resuspended in 300 µl of cold PBS/1% BSA. The microcentrifuge tubes containing the dissociated cells were kept on ice and protected from light. These steps were repeated until all midgut tissue had been digested. The dissociated cells were combined, and the cell suspension was transferred to a 5 ml round-bottomed Falcon tube with filtering through a 40 µm nylon mesh. DAPI was added to the cell suspension at a final concentration of 50 ng/ml, and then the cells were sorted by FACS for further single-cell sequencing analysis.

### ScRNA-seq analysis

ScRNA-seq datasets were mapped to genome dm6 by cellranger (v6.0.2). Gene expression matrices were processed in the Scanpy (v 1.9.3) workflow in the Python 3.10.6 environment. Cells with a number of UMIs or genes between the median±5*MAD (median absolute deviation) were kept for further downstream analysis. Cells were also required that the gene *esg* should be detected. After normalization and log1p transformation, the 3000 most highly variable genes were selected on gene union set for further analysis. The batch effects were removed by scanpy.pp.combat method. Twenty in 100 PCs were used for finding neighbors. Leiden was used as the algorithm to find clusters with a resolution of 1. UMAP (uniform manifold approximation and projection for dimension reduction) was calculated with the parameter min dist=0.3 and recomputed with PAGA initialization. Differentially expressed genes (DEGs) were identified by the scanpy.tl.rank_gene_groups method and Wilcoxon algorithm, using 1.3 as the fold change threshold and 0.05 as the p_adj threshold. Some results were loaded to the R (4.0.3) environment and processed with Seurat (v4.0.0) to visualize.

### Real-time PCR

Total RNA was isolated from GFP+ cells sorted by FACS using Trizol reagent, M-MLV reverse transcriptase (Takara, 2641Q) was used to synthesize cDNA from the total RNA. For the total RNA from human blood, 400 μL of blood from young (20-25 years old) and old (70–76 years old) people was used for total RNA extraction. Real-time PCR analyses were carried out on an ABI 7500 system with SYBR Green Master Mix (Selleck, B21202). Each sample was measured three times. *rp*49 and human *actin* were used as an internal reference gene. See the Table EV1 for the primer sequences.

### Lifespan analysis

Newly eclosed male and female flies were collected. Lifespan experiments were performed using 10 tubes with 20 male and female flies each at 29 °C. Flies were transferred to fresh vials every 2–3 days, and the number of dead flies was counted. Lifespan data were analyzed by oasis2 software, and *P* values were obtained using the log-rank test.

### RNAi in Kc cells

For RNAi experiments utilizing shRNA in Kc cells, the *roX*1 or *roX*2 shRNA was custom-ordered from GeneChem, in a plasmid vector carrying a GFP. Then, the shRNA plasmid was introduced into the cells through transfection using Cellfectin (Invitrogen, 10362100). Following a 48-h incubation, the cells were collected for further analysis.

For the application of double-stranded RNA (dsRNA) in Kc cells to induce RNAi, the sequence of the *Drosophila roX*2 gene was initially amplified using primers that incorporated both the gene sequence and the sequence of a T7 promoter. Subsequently, single-stranded RNA (ssRNA) was synthesized utilizing the HiScribe T7 High Yield RNA Synthesis Kit (NEB, E2040S). This ssRNA then undergoes annealing to form double-stranded RNA (dsRNA). The RNAi experiment was conducted by introducing *roX*2 dsRNA (20 μg) into each well of a 12-well plate, with the incubation period set to either 4 days, respective sequences are listed in the Table EV1.

### LPS treatment in Kc cells

On the day before the treatment, plate 2 × 10^5^ Kc cells per well in a 12-well plate. The following day, LPS treatment was administered at a final concentration of 10 ug/ml (Sigma, #2369), and the cells were incubated for 6 h. Cells treated with an equal concentration of DMSO (MP Biomedicals, 67-68-5) were cultured under the same conditions as a control.

### Chromatin isolation by RNA purification (ChIRP)

*Drosophila* Kc cells (5 × 10^8^ cell equivalents) were treated with DMSO or LPS (10 μg/ml) for 6 h and then harvested and fixed with 1% formaldehyde for 10 min at room temperature. Crosslinking was then quenched with 0.125 M glycine (Biofroxx, 56-40-6) for 5 min and washed with cold PBS twice. Cell pellets were then resuspended at 100 mg/ml in lysis buffer (50 mM Tris 7.0, 10 mM EDTA and 1% SDS with 1 mM DTT, 1 mM PMSF, P.I., and 0.1 µ/μl SUPERase-In added before use) on ice for 10 min and sonicated using a Bioruptor (45 s on/45 s off) until most of the chromatin had solubilized and the DNA was in the size range of 100–500 bp. Then, two volumes of hybridization buffer (750 mM NaCl, 1% SDS, 50 mM Tris 7.0, 1 mM EDTA and 15% formamide with 1 mM DTT, 1 mM PMSF, P.I., and 0.1 U/μl SUPERase-In freshly added) and 1 μl of 100 pmol probes were added and mixed by end-to-end rotation at 37 °C for 4 h. Streptavidin magnetic C1 beads (Invitrogen, #65001) were washed three times in lysis buffer, blocked with 1 mg/ml BSA for 1 h at room temperature, and washed three times again in lysis buffer. Then, 100 μl of washed/blocked C1 beads was added per 100 pmol of probes, and the whole reaction was mixed for another 30 min at 37 °C. The mixture was then washed five times with wash buffer (2 × SSC, 0.5% SDS, add 1 mM DTT and 1 mM PMSF fresh). For protein elution, beads were resuspended in 30 μl DNase buffer (100 mM NaCl, 0.1% NP-40, 100 μg/ml RNase A (Takara, 2158), 0.1 U/μl RNase H (Takara, 2151), and 100 U/ml DNase I(Takara, 2270))at 37 °C for 30 min. The protein eluent was supplemented with a 0.2 volume of 5× SDS loading buffer (Beyotime, P0015) and boiled for 30 min. The protein was subjected to western blotting.

### Constructs

The pIB/V5-His-TOPO construct expressing truncated HP1a fused with GFP was constructed by fusing the GFP-tag with the truncations of *D. melanogaster* HP1a, and then subcloned into the vector. To express mouse *Xist* repeat A, C, or D in Kc cells, vectors were constructed by subcloning the specific regions of *Xist* that were amplified by PCR into pIB/V5-His-TOPO. Primer sequences are provided in Table EV1.

### Establishing stable lines in Kc cells

The transfection of Kc cells with the constructs was performed following a standard protocol by Invitrogen. Briefly, 2 × 10^5^ cells per well were plated in a 12-well plate. During the transfection, 2 μg of DNA was added to serum-free medium and mixed with 3 μl of Cellfectin (Invitrogen, 10362100) also in serum-free medium, followed by a 30-min incubation at room temperature. Subsequently, 1 ml of serum-free medium was added to each well, and the mixture of DNA and Cellfectin was introduced to the cells, followed by a 6-h incubation period before switching to normal growth medium. Forty-eight hours post-transfection, antibiotic selection was applied to eliminate cells without integration of the foreign gene.

### RNA immunoprecipitation (RIP)

Kc cells were transfected with wild-type or containing different domains of HP1a expression plasmids and treated with DMSO or LPS (10 μg/ml) for 6 h, and then harvested and fixed with 1% formaldehyde for 10 min at room temperature. Crosslinking was then quenched with 0.25 M glycine for 5 min and washed with cold PBS twice. Fixed cell pellets were then resuspended in 2 ml lysis buffer (50 mM Tris-HCl, pH 7.5, 150 mM NaCl, 1% NP-40, 0.5% sodium deoxycholate, 1 mM EDTA, and 0.05% SDS with protease inhibitors and 0.1 µ/μl SUPERase-In added before use) on ice for 10 min and sonicated using a Bioruptor (45 s on/45 s off) until most of the chromatin had solubilized. Insoluble material is removed by microcentrifugation at 14,000 rpm for 10 min at 4 °C. The supernatant was added 20 μl protein G beads and nonspecific competitor tRNA (Invitrogen, AM7119) (100 µg/ml) to preclear the lysate. After removing beads, the supernatant was added to the GFP (gta-20) antibody or HP1a antibody (C1A9) for overnight at 4 °C. The beads are washed five times with 1 ml of wash buffer (50 mM Tris-HCl, pH 7.5, 800 mM NaCl, 1% NP-40, 1% sodium deoxycholate, 1 mM EDTA, 1 M urea, and 0.1% SDS with protease inhibitors, 0.1 µ/μl SUPERase-In and 0.2 mM PMSF by 10 min rotation at room temperature. The beads containing the immunoprecipitated samples are collected and resuspended in 100 μl of 50 mM Tris-HCl, pH 7.0, 5 mM EDTA, 10 mM DTT, and 1% SDS and incubated at 70 °C for 45 min. RNA purification using Trizol and reverse transcriptase according to the manufacturer’s protocol. The cDNA was subjected to QPCR, which was run in a Roche Light Cycler 96 thermocycler as follows: one cycle of 95 °C for 1 min; 40 cycles of 95 °C for 1 min, 60 °C for 1 min, and 72 °C for 1 min; and one cycle of 72 °C for 5 min. The primer sequences for different regions of *roX*2 were utilized as previously reported (Nature Biotechnology 10.1038/nbt.2943). The data from the immunoprecipitates were normalized with the data from the 1% input samples.

### RNA fluorescence in situ hybridization

Briefly, five pairs of salivary glands were dissected during the third-instar larvae in PBS and then fixed in 4% formaldehyde for 20 min at room temperature. After the fixation, rinsed the tissues in 0.1% PBST three times for 5 min each. Then using 100%/75%/50%/25% methanol in 0.1% PBST to rehydrate the tissues, wash them three times with 0.1% PBST, and incubated the tissues in 10 μg/mL proteinase K (Takara, 9034) for 5 min. Following three washes in 0.1% PBST, the tissues were fixed again in 4% PFA for 20 min. For the rest steps, we followed the multiplexed RNA-FISH protocol provided by Molecular Instruments (https://files.molecularinstruments.com/MI-Protocol-RNAFISH-GenericSolution-Rev7.pdf). *roX*2 probes were a lot. RTK597. Amplifier: Molecular Instruments.

### Western blot analysis

Salivary glands from third-instar larvae were dissected and homogenized in 4% SDS. The lysate was heated in loading buffer at 95 °C for 10 min. For Western blotting using Kc stable lines, cells were lysed in 5× SDS loading buffer (Beyotime, P0015) and boiled for 10 min. The supernatant was collected upon centrifugation. Protein extracts were electrophoresed on a 12% SDS/PAGE gel and transferred to PVDF membranes. The following antibodies were used: anti-HP1a, anti-H3K9me3, anti-H3, anti-actin.

### ATAC-seq analysis

Kc cells were harvested 48 h post-transfection and lysed in cold lysis buffer. Following lysis, nuclei were pelleted by centrifugation at 1500 × *g* for 10 min at 4 °C. The nuclear pellet was then resuspended in a transposase reaction mixture containing 10 µL of 5× TD buffer, 1 µL of transposase, and 40 µL of nuclease-free water. The transposition reaction was performed at 37 °C for 30 min. After transposition, the DNA was purified using a ZYMO DNA Clean & Concentrator kit. Library fragments were amplified with 1× PCR master mix and custom Nextera PCR primers under the following cycling conditions: 72 °C for 5 min; 98 °C for 30 s; followed by 12 cycles of 98 °C for 10 s, 63 °C for 30 s, and 72 °C for 1 min. Finally, the libraries were purified using AMPure XP beads and subjected to high-throughput sequencing.

For data analysis, sequencing files were removed of adapters by fastp with parameter --detect_adapter_for_pe. Then clean reads mapped to genome dm6 and MG1655 (*Escherichia coli* strain K12) by bowtie2 with parameters: --local --very-sensitive-local --no-mixed --no-discordant --no-unal --phred33 –X 2000. Low mapping quality reads were removed by samtools view with -F 0×904 -q 20. Duplications were removed by sambamba markdup –r, and reads mapped to unusual chromosomes (e.g., chrM, chrUn or random) were also removed. Bam files mapped to dm6 were transferred to bedgraph and bigwig (20 bp window) by bedtools[7] genomecov and bamCoverage, using scale factors 10,000,000/(reads mapped to MG1655). Heatmap was calculated by computeMatrix scale-regions with --missingDataAsZero -a 3000 -b 3000 -m 5000 -bs 20 and plotted by plotHeatmap. Peaks were called by macs3 callpeak with -g dm --shift -100 --extsize 200 --nomodel -B -f BAMPE, and peaks with normalized value lower than 50 were removed. Differential peaks were called by macs3 bdgdiff.

### Quantification and statistical analysis

Statistics and graph creation were performed using GraphPad Prism 6. Data are shown as the mean values and standard error (SEM). The significance of differences (*P* values) was analyzed by Student’s unpaired two-tailed *t* test, unless otherwise indicated. N (number) is shown for each experiment. *P* < 0.05 was deemed indicative of statistical significance.

## Supplementary information

Table EV1

Appendix

Peer Review File

Source data Fig. 1

Source data Fig. 2

Source data Fig. 3

Source data Fig. 4

Source data Fig. 5

Source data Fig. 6

Source data Fig. 7

Expanded View Figures

## Data Availability

The accession numbers for the sequencing data reported in this paper are GEO: GSE140893, GEO: GSE190886. The main code for scRNA-seq analysis is hosted at: https://gitfront.io/r/yhli/sG6nFFmoABrb/Dm-roX-codes/, with additional supplemental analysis code at https://gitfront.io/r/yhli/nGz7ZsWSHPa7/Dm-roX-supplement-codes/. The main code for ATAC-seq analysis is hosted at: https://gitfront.io/r/yhli/Z6mfnvWkuWNe/Dm-roX2OE-ATAC-codes/. All data supporting the findings of this study are available from the corresponding author upon request. The source data of this paper are collected in the following database record: biostudies:S-SCDT-10_1038-S44319-026-00791-8.
