# Supplementary material for: roX1 and roX2 lncRNAs promote heterochromatinization in intestinal stem cells and impair longevity
Source: EMBO Rep. 2026 May 9;27(12):3394–423. doi: 10.1038/s44319-026-00791-8 (PMC13303914; doi:10.1038/s44319-026-00791-8)
Supplement: Supplementary file 2 — Appendix [file 44319_2026_791_MOESM2_ESM.pdf]

Appendix for

*roX*1 and *roX*2 lncRNAs promote heterochromatinization in intestinal stem cells and impair longevity

**Table of Contents**	Page
Appendix Figure S1	2
Appendix Figure S2	5
Appendix Figure S3	6
Appendix Figure S4	9
Appendix Figure S5	11
Appendix Figure S6	12
Appendix Figure S7	13
Appendix Figure S8	15
Appendix Figure S9	16
Appendix Figure S10	17
Appendix Figure S11	19
Appendix Figure S12	20
Appendix Figure S13	21
Appendix Figure S14	22
Appendix Figure S15	23

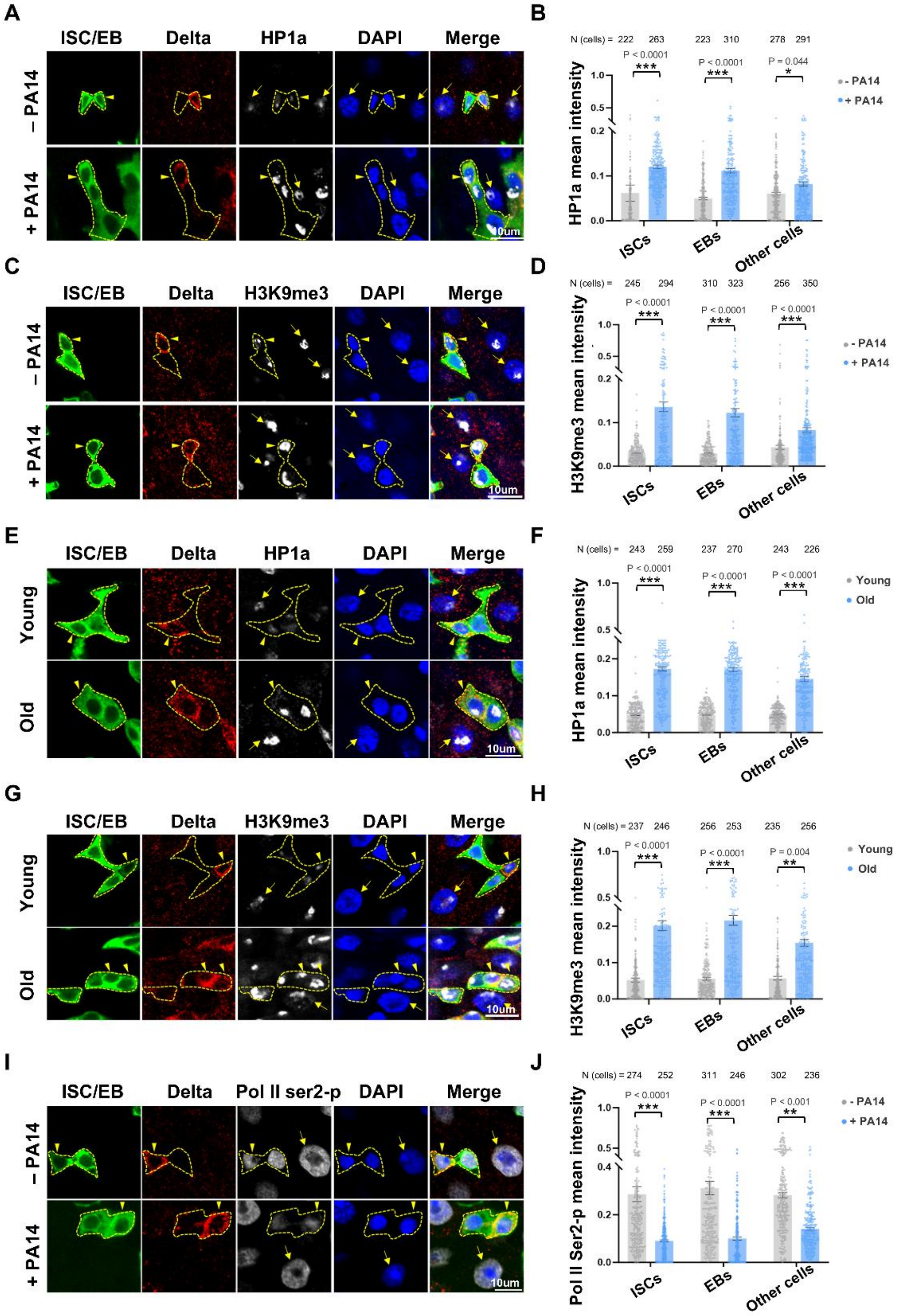

Appendix Figure S1. Stress-induced heterochromatin expansion in the female intestines.

(A) Representative images of HP1a immunofluorescence staining in Esg^{+}Delta^{+} (ISC), Esg^{+}Delta^{-} (EB), and Esg^{-}Delta^{-} (other cells) cells from the midgut of *esg-Gal4, UAS-GFP* female flies after a 6-day infection with PA14. Dashed outlines delineate the Esg^{+} cells (ISCs and EBs). Arrowheads

indicate the $Delta^+$ ISCs, and arrows point to the Esg^-Delta^- (other) cells.

(B) Quantification of HP1a intensity in (A). HP1a intensity was quantified in the three defined cell types: Esg^+Delta^+ (ISC), Esg^+Delta^- (EB), and Esg^-Delta^- (other) cells. N = 3.

(C) Representative images of H3K9me3 immunofluorescence staining in Esg^+Delta^+ (ISC), Esg^+Delta^- (EB), and Esg^-Delta^- (other cells) cells from the midgut of *esg-Gal4, UAS-GFP* female flies after a 6-day infection with PA14. Dashed outlines delineate the Esg^+ cells (ISCs and EBs). Arrowheads indicate the $Delta^+$ ISCs, and arrows point to the Esg^-Delta^- (other) cells.

(D) Quantification of H3K9me3 intensity in (C). H3K9me3 intensity was quantified in the three defined cell types: Esg^+Delta^+ (ISC), Esg^+Delta^- (EB), and Esg^-Delta^- (other) cells. N = 3.

(E) HP1a immunofluorescence in the midgut of young and old *esg-Gal4, UAS-GFP* female flies. Dashed outlines delineate the Esg^+ cells (ISCs and EBs). Arrowheads indicate the $Delta^+$ ISCs, and arrows point to the Esg^-Delta^- (other) cells.

(F) Quantification of HP1a intensity in (E) across the three cell types: Esg^+Delta^+ (ISC), Esg^+Delta^- (EB), and Esg^-Delta^- (other) cells. N = 3.

(G) H3K9me3 immunofluorescence in the midgut of young and old *esg-Gal4, UAS-GFP* female flies. Dashed outlines delineate the Esg^+ cells (ISCs and EBs). Arrowheads indicate the $Delta^+$ ISCs, and arrows point to the Esg^-Delta^- (other) cells.

(H) Quantification of H3K9me3 intensity in (G) across the three cell types: Esg^+Delta^+ (ISC), Esg^+Delta^- (EB), and Esg^-Delta^- (other) cells. N = 3.

(I) Representative images of Pol II Ser2p immunofluorescence from both untreated and PA14-infected midguts of female flies. Staining is shown in the three cell types: Esg^+Delta^+ (ISC), Esg^+Delta^- (EB), and Esg^-Delta^- (other) cells from *esg-Gal4, UAS-GFP* female flies. Dashed

outlines delineate the Esg^{+} cells (ISCs and EBs). Arrowheads indicate the $Delta^{+}$ ISCs, and arrows point to the $Esg^{-}Delta^{-}$ (other) cells.

(J) Quantification of Pol II Ser2p intensity in (I) across the three cell types: $Esg^{+}Delta^{+}$ (ISC), $Esg^{+}Delta^{-}$ (EB), and $Esg^{-}Delta^{-}$ (other) cells. N = 3. Data are from three independent biological replicates. The number of cells is indicated on the graph. The center values are the averages, and the error bars indicate the s.e.m. P values were obtained by two-tailed unpaired Student's t-test. n.s., not significant, $p \geqslant 0.05$, $*p < 0.05$, $**p < 0.01$, $***p < 0.0001$. All images were captured from adult female posterior midguts. Scale bar, 10 μm.

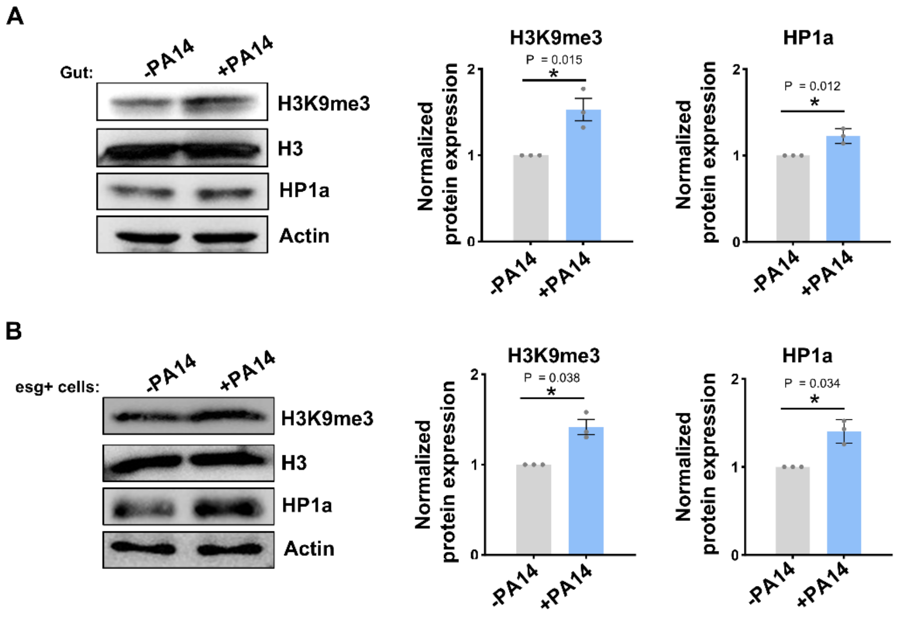

Appendix Figure S2. Western blot analysis of heterochromatin markers in females.

(A) Western blot analysis was performed to detect the levels of HP1a and H3K9me3 in the midgut. Actin and histone H3 were used as loading controls. Band intensities were quantified by densitometry, normalized to the internal loading control, and expressed relative to the uninfected control (−PA14, set to 1). N = 3.

(B) Analysis of HP1a and H3K9me3 by Western blot in FACS-sorted esg$^+$ cells (ISCs/EBs), with Actin and H3 serving as loading controls. Band intensities were quantified by densitometry, normalized to the internal loading control, and expressed relative to the uninfected control (−PA14, set to 1). N = 3. The center values are the averages, and the error bars indicate the s.e.m. P values were obtained by two-tailed unpaired Student's t-test. n.s., not significant, $p \geqslant 0.05$, $^*p < 0.05$, $^{**}p < 0.01$, $^{***}p < 0.0001$.

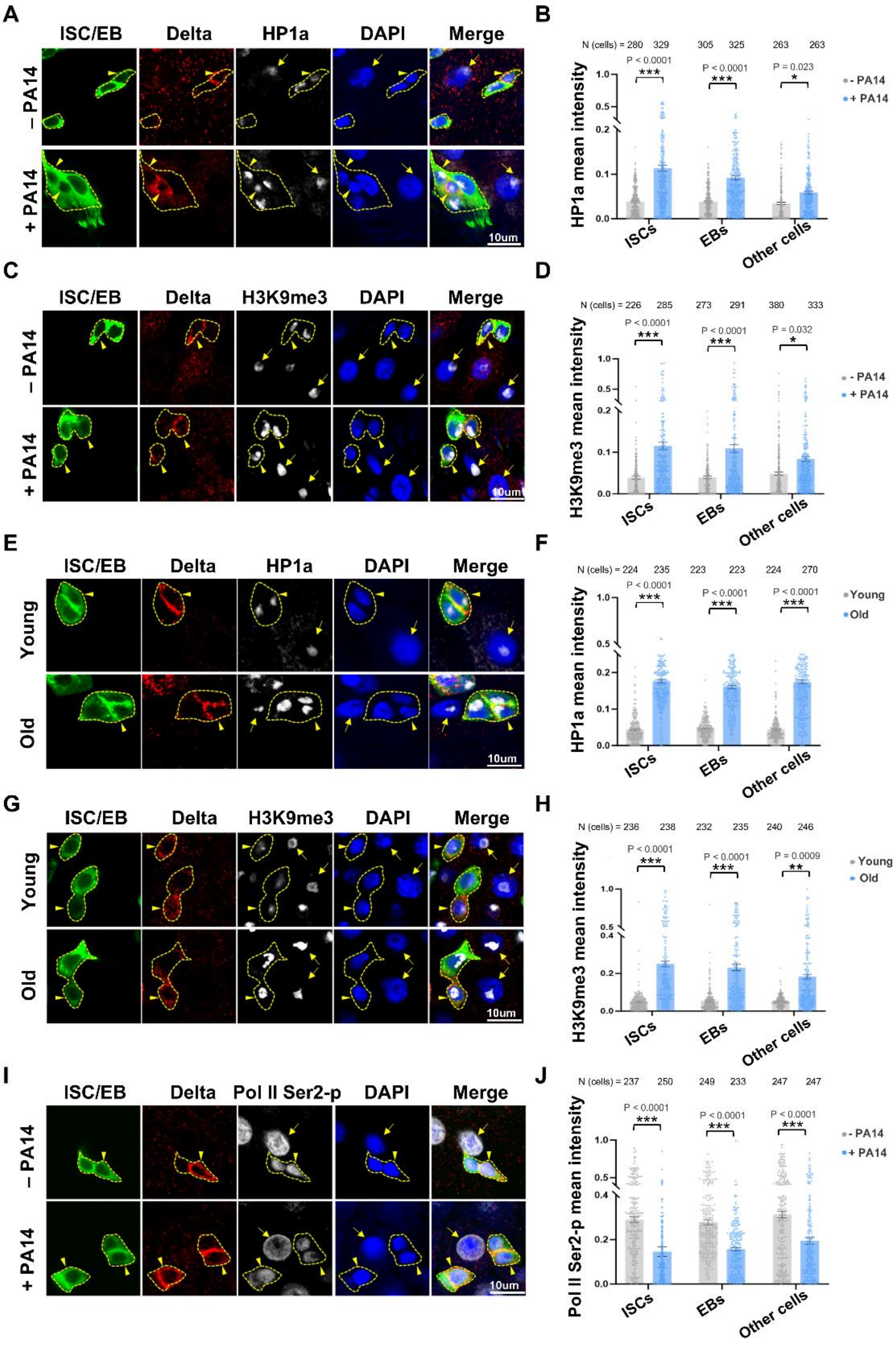

Appendix Figure S3. Stress-induced heterochromatin expansion is also observed in male intestines.

(A) Representative images of HP1a immunofluorescence staining in Esg$^+$Delta$^+$ (ISC), Esg$^+$Delta$^-$ (EB), and Esg$^-$Delta$^-$ (other cells) cells from the midgut of *esg-Gal4, UAS-GFP* male flies after a 6-day infection with PA14. Dashed outlines delineate the Esg$^+$ cells (ISCs and EBs). Arrowheads

indicate the $Delta^+$ ISCs, and arrows point to the Esg^-Delta^- (other) cells.

(B) Quantification of HP1a intensity in (A). HP1a intensity was quantified in the three defined cell types: Esg^+Delta^+ (ISC), Esg^+Delta^- (EB), and Esg^-Delta^- (other) cells. N = 3.

(C) Representative images of H3K9me3 immunofluorescence staining in Esg^+Delta^+ (ISC), Esg^+Delta^- (EB), and Esg^-Delta^- (other cells) cells from the midgut of esg-Gal4, UAS-GFP male flies after a 6-day infection with PA14. Dashed outlines delineate the Esg^+ cells (ISCs and EBs). Arrowheads indicate the $Delta^+$ ISCs, and arrows point to the Esg^-Delta^- (other) cells.

(D) Quantification of H3K9me3 intensity in (C). H3K9me3 intensity was quantified in the three defined cell types: Esg^+Delta^+ (ISC), Esg^+Delta^- (EB), and Esg^-Delta^- (other) cells. N = 3.

(E) HP1a immunofluorescence in the midgut of young and old esg-Gal4, UAS-GFP male flies. Dashed outlines delineate the Esg^+ cells (ISCs and EBs). Arrowheads indicate the $Delta^+$ ISCs, and arrows point to the Esg^-Delta^- (other) cells.

(F) Quantification of HP1a intensity in (E) across the three cell types: Esg^+Delta^+ (ISC), Esg^+Delta^- (EB), and Esg^-Delta^- (other) cells. N = 3.

(G) H3K9me3 immunofluorescence in the midgut of young and old *esg-Gal4, UAS-GFP* male flies. Dashed outlines delineate the Esg^+ cells (ISCs and EBs). Arrowheads indicate the $Delta^+$ ISCs, and arrows point to the Esg^-Delta^- (other) cells.

(H) Quantification of H3K9me3 intensity in (G) across the three cell types: Esg^+Delta^+ (ISC), Esg^+Delta^- (EB), and Esg^-Delta^- (other) cells. N = 3.

(I) Representative images of Pol II Ser2p immunofluorescence in male midguts, from both untreated and PA14-infected conditions. Dashed outlines delineate the Esg^+ cells (ISCs and EBs). Arrowheads indicate the $Delta^+$ ISCs, and arrows point to the Esg^-Delta^- (other) cells.

(J) Quantification of Pol II Ser2p intensity in (I) across the three cell types. N = 3. Data are from three independent biological replicates. The number of cells is indicated on the graph. The center values are the averages, and the error bars indicate the s.e.m. P values were obtained by two-tailed unpaired Student's t-test. n.s., not significant, $p \geqslant 0.05$, *$p < 0.05$, **$p < 0.01$, ***$p < 0.0001$. All images were captured from adult male posterior midguts. Scale bar, 10 μm.

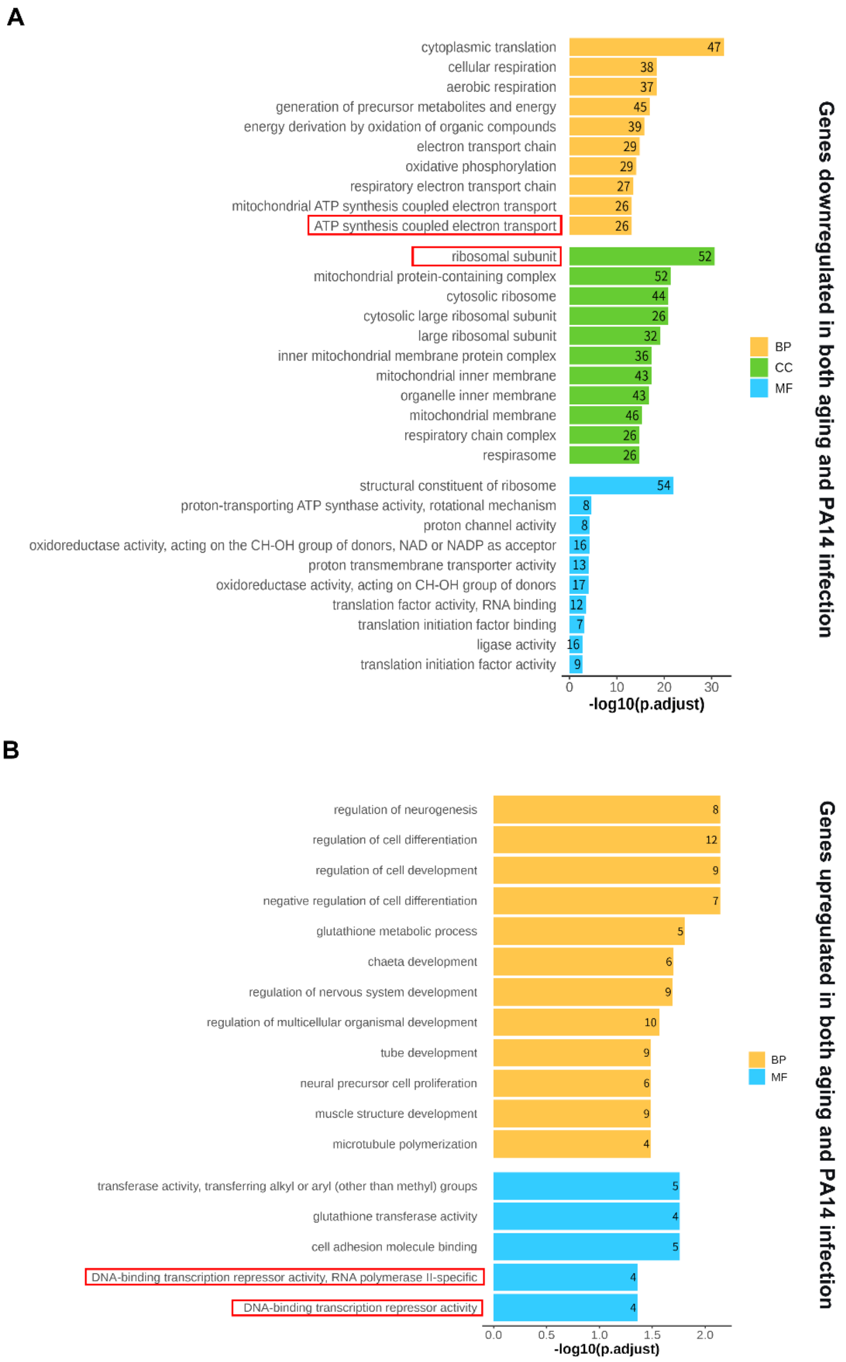

Appendix Figure S4. Pathways are affected by both aging and PA14 infection.

(A) GO analysis of genes downregulated in both aging and PA14 infection. The downregulated genes were significantly associated with "ATP synthesis" and "ribosome biogenesis" (highlighted by a red box), indicating a decline in metabolic and translational capacity during aging or infection. The classifications of GO terms are shown in different colours. Light orange, biological process

(BP); green, cellular component (CC); blue, molecular function (MF). The length of each column represents the -log10(p. adjust) of the associated GO term. The number of genes in each enriched function is marked in the column.

(B) GO analysis of genes upregulated in both aging and PA14 infection. Genes upregulated in both conditions were strongly enriched for the GO term "DNA-binding transcription repressor activity" (highlighted by a red box).

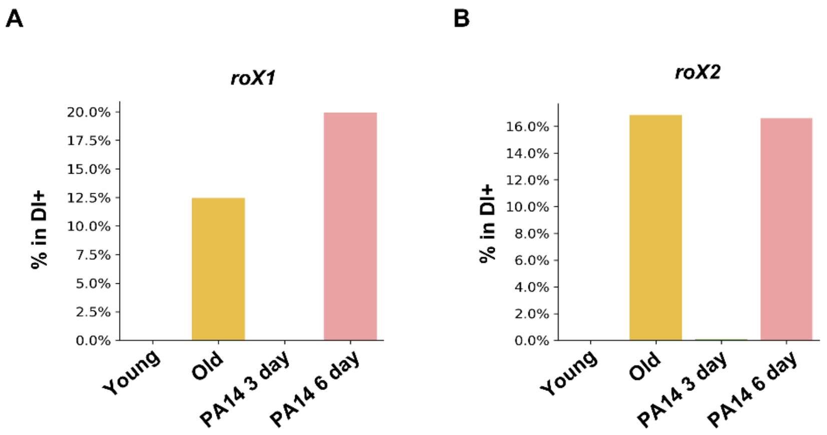

Appendix Figure S5. Proportion of Delta⁺ ISCs with detectable *roX*1 or *roX*2 RNA across conditions.

(A) The percentage of Dl+ cells in which *roX*1 RNA was detected. (B) The percentage of Dl+ cells containing detectable *roX*2 RNA.

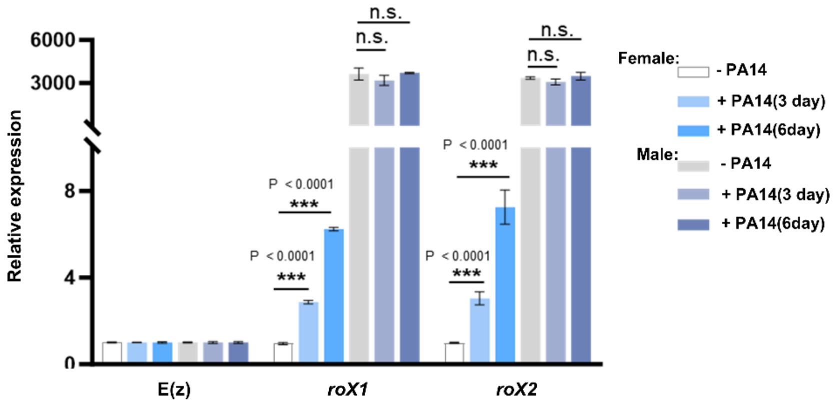

Appendix Figure S6. *roX* RNA expression in male and female ISCs/EBs after infection.

(A) qPCR analysis of roX RNA levels in FACS-sorted esg^+ cells (ISCs/EBs) after PA14 infection. *roX* RNA expression is specifically upregulated in females upon infection, with no significant change detected in males. *E(z)* serves as a negative control. N = 3, the center values are the averages, and the error bars indicate the s.e.m. P values were obtained by two-tailed unpaired Student's t-test. n.s., not significant, p ⩾ 0.05, *p < 0.05, **p < 0.01, ***p < 0.0001.

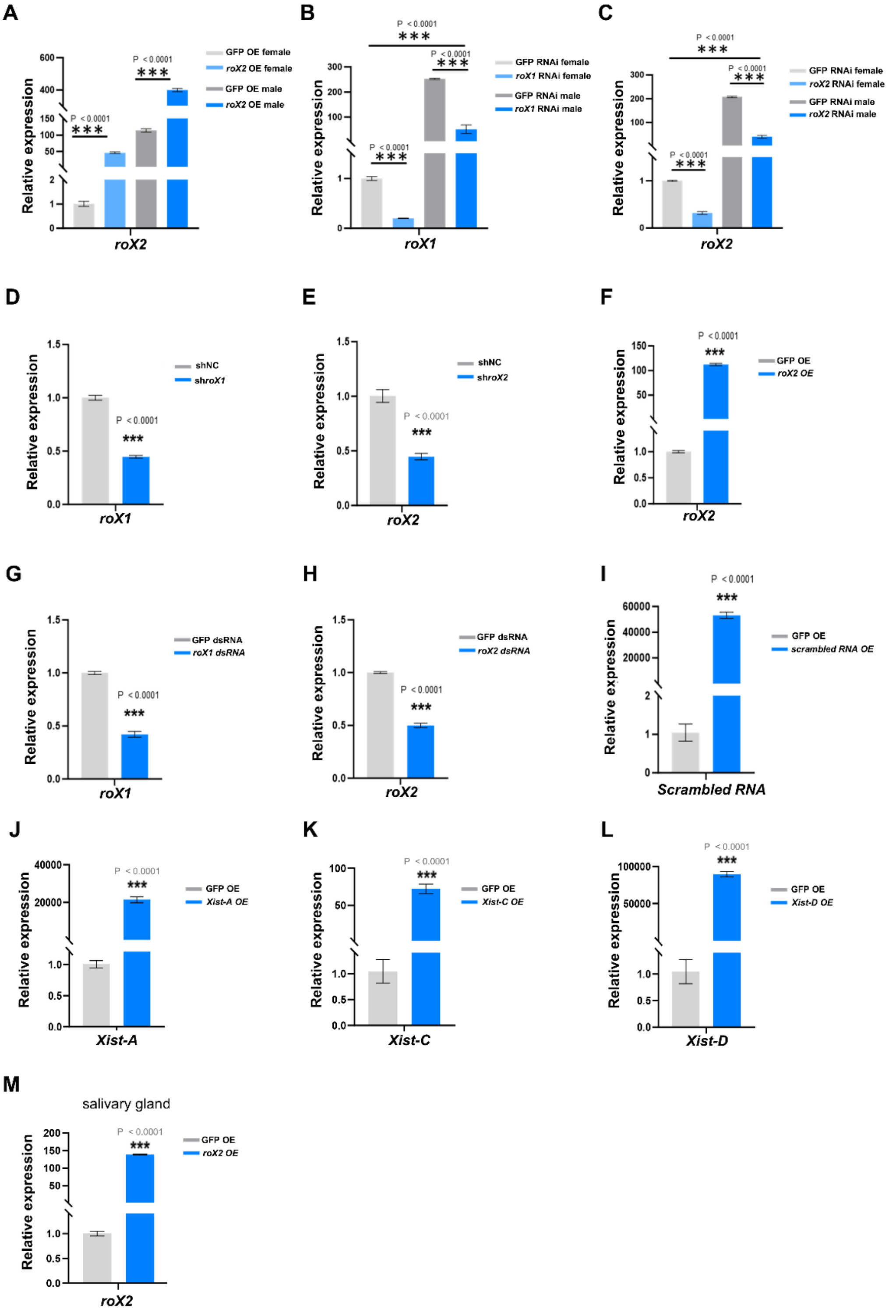

Appendix Figure S7. Validation of overexpression and knockdown efficiency.

(A) qPCR analysis of *roX2* levels in both male and female flies with *roX2* overexpression driven by *actin-GAL4*. N = 3.

(B-C) Validation of *roX*1 (B) and *roX*2 (C) RNAi efficiency. The *act-GAL4* driver was used to ubiquitously express the RNAi construct in both male and female flies. N = 3.

(D-E) Validation of knockdown efficiency for *roX*1 (D) and *roX*2 (E) shRNA in KC cells. N = 3.

(F) qPCR analysis of *roX*2 transcript levels in KC cells to validate the overexpression efficiency. N = 3.

(G-H) Validation of knockdown efficiency for *roX*1 (D) and *roX*2 (E) dsRNA in KC cells. N = 3.

(I-L) Detection of expression levels for the scrambled RNA control (I), *Xist-A* (J), *Xist-C* (K), and *Xist-D* (L) in KC cells. N = 3.

(M) qPCR analysis of *roX*2 transcript levels in salivary glands to validate the overexpression efficiency. N = 3. Data are from three independent biological replicates. The center values are the averages, and the error bars indicate the s.e.m. P values were obtained by two-tailed unpaired Student's t-test. n.s., not significant, $p \geqslant 0.05$, *$p < 0.05$, **$p < 0.01$, ***$p < 0.0001$.

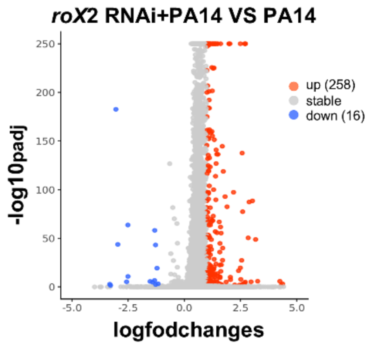

Appendix Figure S8. Volcano plot under a stringent fold-change threshold.

A version of the plot that applies a higher significance threshold (fold change = 2).

A

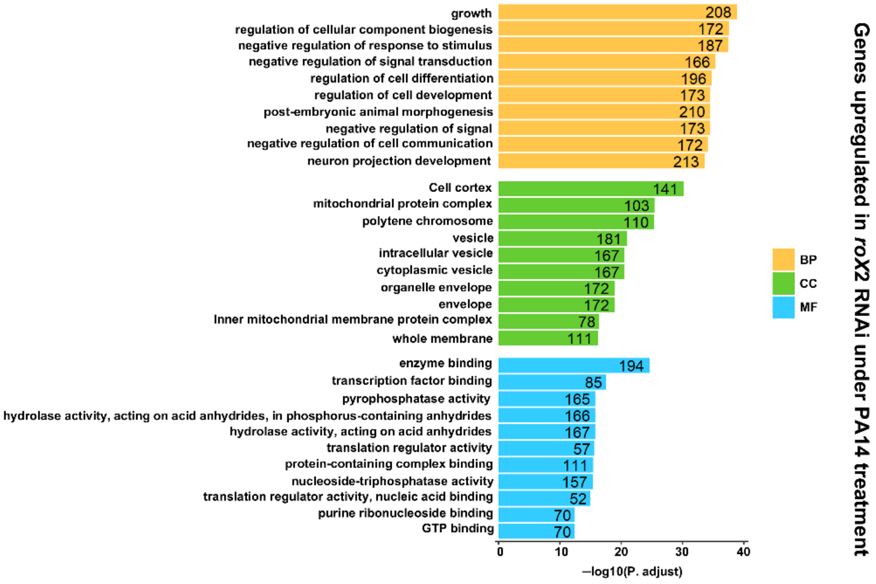

Appendix Figure S9. Gene Ontology analysis after *roX2* knockdown.

(A) Results of GO analysis of genes upregulated in roX2 RNAi vs. control cells under PA14 treatment. Enrichment of the GO term "transcription factor binding" upon roX2 knockdown indicates enhanced transcriptional activity (highlighted by a red box). The classifications of GO terms are shown in different colours. Light orange, biological process (BP); green, cellular component (CC); blue, molecular function (MF). The length of each column represents the -log10(p. adjust) of the associated GO term. The number of genes in each enriched function is marked in the column.

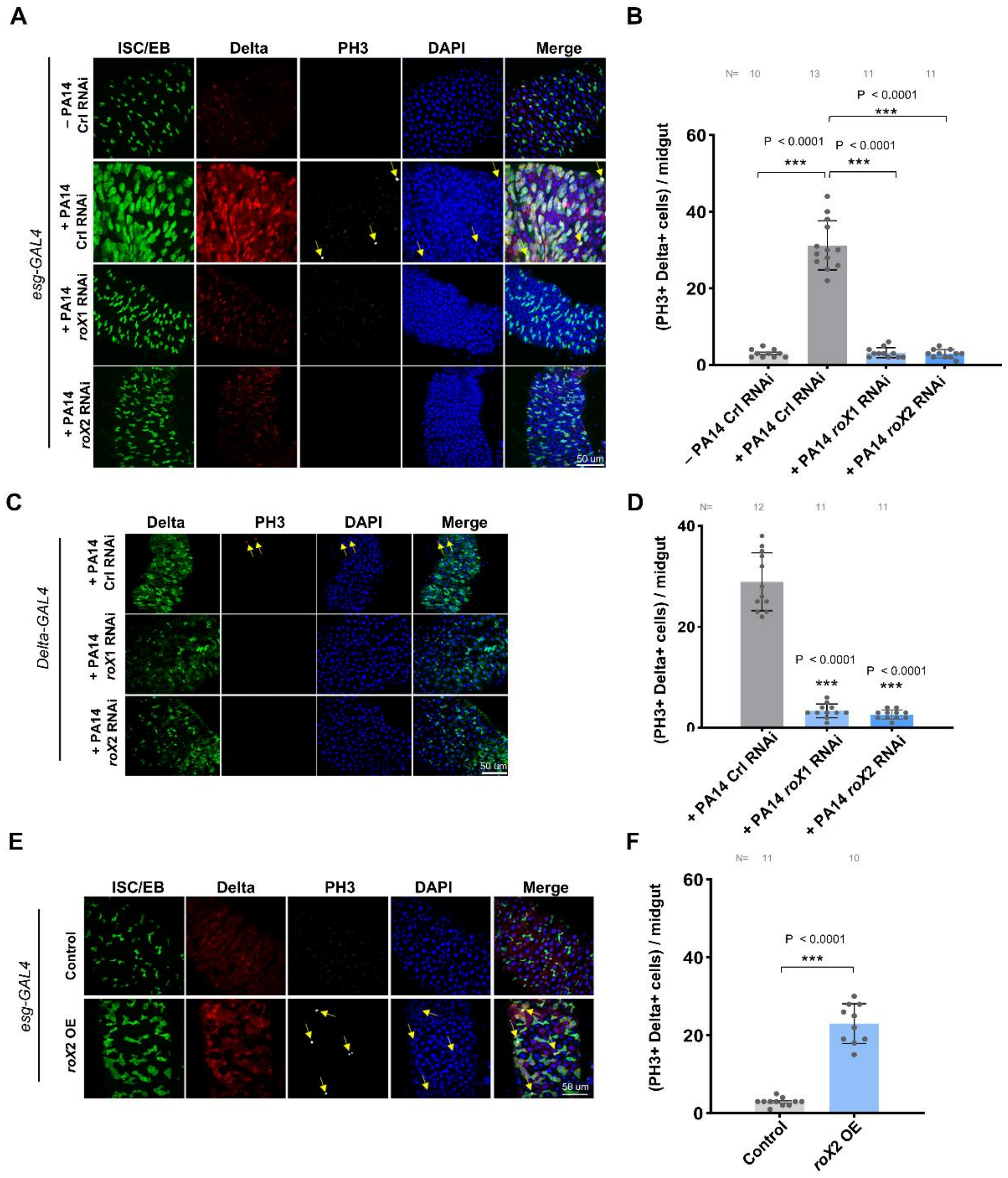

Appendix Figure S10. Quantification of mitotic ISCs by PH3 immunofluorescence.

(A) Representative images of midguts stained for pH3. *roX*1/2 RNAi is driven by *esg-Gal4, UAS-GFP*. Luciferase RNAi served as the control. Arrows indicate PH3$^+$Delta$^+$ cells.

(B) The number of PH3$^+$Delta$^+$ cells per midgut in (A) was quantified. N = 3, the number of midguts is indicated on the graph.

(C) Representative images of midguts stained for pH3. *roX*1/2 RNAi is driven by *Delta-Gal4.* Luciferase RNAi served as the control. Arrows indicate PH3$^+$Delta$^+$ cells.

(D) Quantification of the number of PH3$^+$Delta$^+$ cells per midgut shown in (C). N = 3, the number of midguts is indicated on the graph.

(E) PH3 staining in the midgut upon *roX2* overexpression (driven by *esg-Gal4, UAS-GFP*). Arrows indicate PH3$^+$Delta$^+$ cells.

(F) Quantification of the number of PH3$^+$Delta$^+$ cells per midgut shown in (E). N = 3. The number of midguts is indicated on the graph. The center values are the averages, and the error bars indicate the s.e.m. P values were obtained by two-tailed unpaired Student's t-test. n.s., not significant, $p \geqslant 0.05$, $*p < 0.05$, $**p < 0.01$, $***p < 0.0001$.

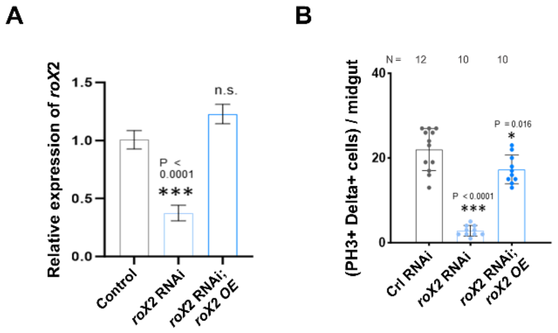

Appendix Figure S11 Rescue assay involving re-expression of *roX*2 in a *roX*2 RNAi background to restore ISCs hyperplasia under PA14 infection.

(A) To validate the re-expression of *roX*2 in the knockdown background, *roX*2 levels were quantified by qPCR. N = 3.

(B) Quantification of the proportion of total ISCs and PH3$^+$ ISCs in the posterior midgut. N = 3. The number of midguts is indicated on the graph. The center values are the averages, and the error bars indicate the s.e.m. P values were obtained by two-tailed unpaired Student's t-test. n.s., not significant, $p \geqslant 0.05$, $*p < 0.05$, $**p < 0.01$, $***p < 0.0001$.

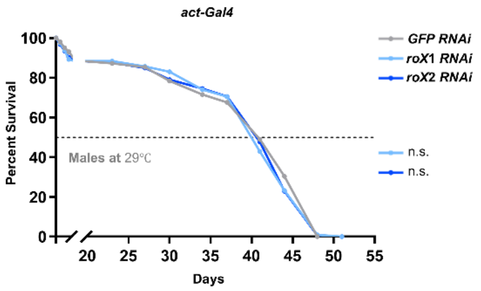

Appendix Figure S12. Lifespan for the male flies with depletion of roX RNAs in the whole body.

The maximal and median longevity of males after RNAi against GFP or *roX*1/2 by *actin-Gal4*; the dotted line indicates the median longevity (N = 3, n = 200 flies for each group). The centre values are the averages.

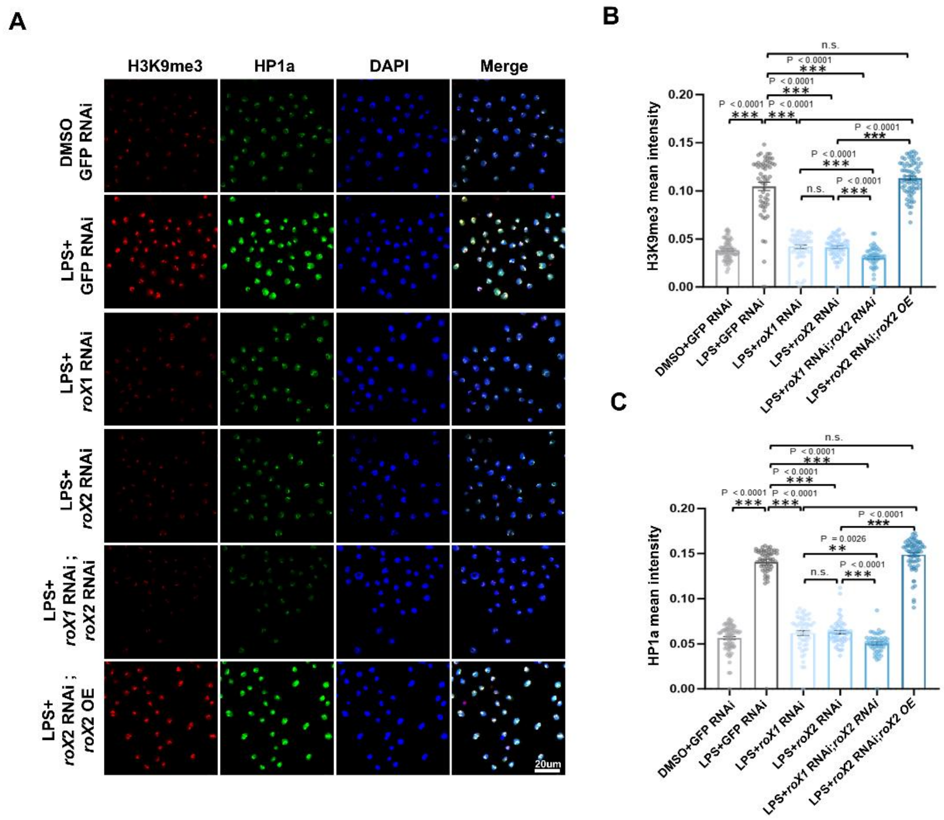

Appendix Figure S13. Rescue of heterochromatin defects upon *roX* knockdown by *roX* expression.

(A) HP1a and H3K9me3 staining in KC cells under LPS treatment. Compared to single *roX*1 or *roX*2 knockdown (dsRNA), double knockdown results in a significant loss of heterochromatin. This loss is rescued by re-expression of *roX*2 in the *roX*2 knockdown (dsRNA) background.

(B-C) Quantitative data for H3K9me3 (B) and HP1a (C) intensity in the groups from (A). N = 3, n ≥ 50 cells. The center values are the averages, and the error bars indicate the s.e.m. P values were obtained by two-tailed unpaired Student's t-test. n.s., not significant, p ≥ 0.05, *p < 0.05, **p < 0.01, ***p < 0.0001.

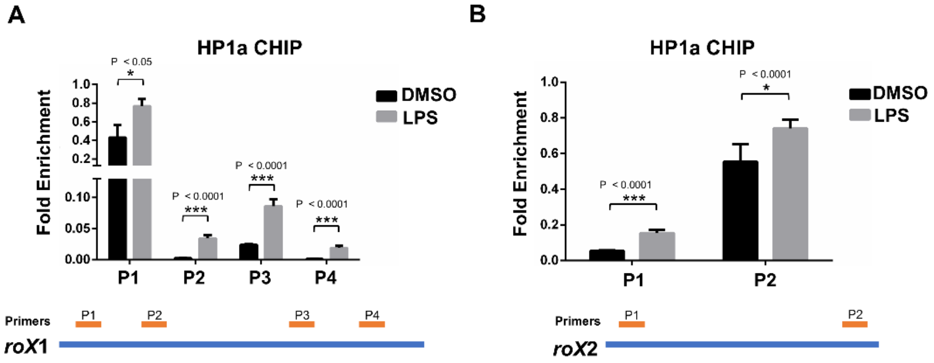

Appendix Figure S14. RNA immunoprecipitation with HP1a antibody

(A-B) RIP was performed with an anti-HP1a antibody in LPS^{-} and LPS^{+} conditions. The association of HP1a with *roX*1(A) *or roX*2(B) RNA genomic regions was quantified by qPCR. N = 3. The centre values are the averages, and the error bars indicate the s.e.m. P values were obtained by two-tailed unpaired Student's t test. n.s., not significant, $p \geq 0.05$, $*p < 0.05$, $**p < 0.01$, $***p < 0.0001$.

A

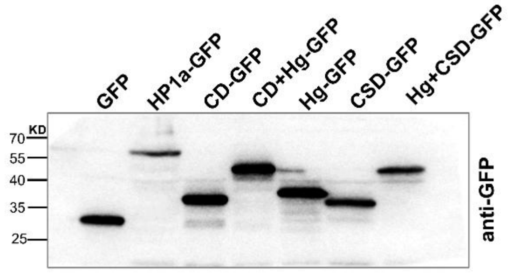

Appendix Figure S15. Validation of GFP-HP1α fusion protein expression by Western blot.

(A) Validation of fusion protein expression in KC cells by Western blot using an anti-GFP antibody. The predicted molecular weights are as follows: GFP (~25 kDa), HP1a-GFP (~57 kDa), CD-GFP (~35 kDa), CD+Hg-GFP (~41 kDa), Hg-GFP (~38 kDa), Hg+CSD-GFP (~40 kDa), and CSD-GFP (~33 kDa). N = 3.
