# Supplementary figures and images for: roX1 and roX2 lncRNAs promote heterochromatinization in intestinal stem cells and impair longevity (part 1 of 3)
Source: EMBO Rep. 2026 May 9;27(12):3394–423. doi: 10.1038/s44319-026-00791-8 (PMC13303914; doi:10.1038/s44319-026-00791-8)

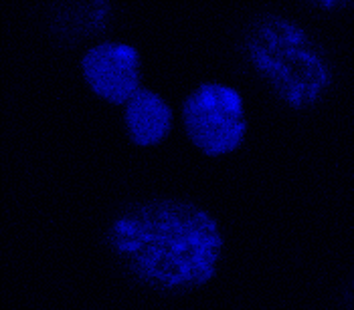

Supplement: Supplementary file 4 — Source data Fig. 1 [file 44319_2026_791_MOESM4_ESM.zip › Figure 1/B/+PA14-DAPI.png]

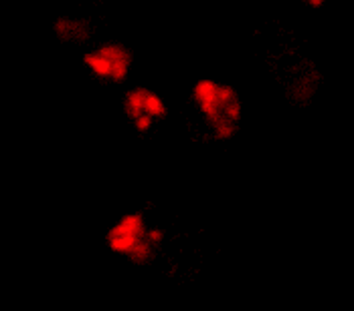

Supplement: Supplementary file 4 — Source data Fig. 1 [file 44319_2026_791_MOESM4_ESM.zip › Figure 1/B/+PA14-HP1a.png]

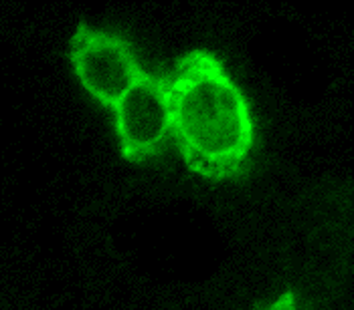

Supplement: Supplementary file 4 — Source data Fig. 1 [file 44319_2026_791_MOESM4_ESM.zip › Figure 1/B/+PA14-ISC-EB.png]

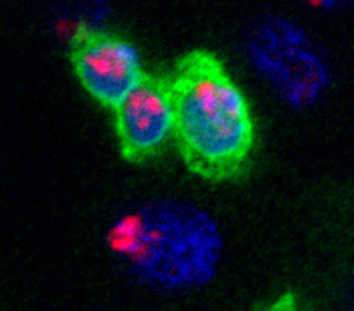

Supplement: Supplementary file 4 — Source data Fig. 1 [file 44319_2026_791_MOESM4_ESM.zip › Figure 1/B/+PA14-merge.png]

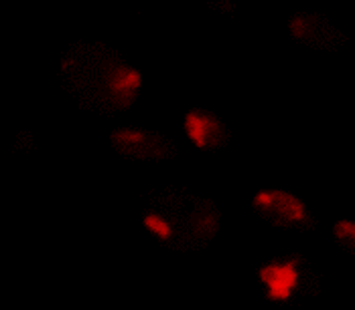

Supplement: Supplementary file 4 — Source data Fig. 1 [file 44319_2026_791_MOESM4_ESM.zip › Figure 1/B/-PA14-HP1a.png]

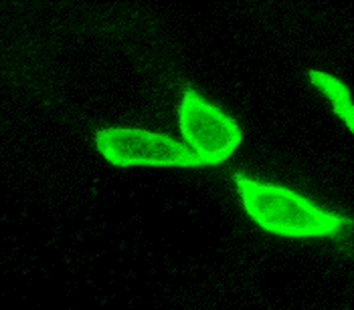

Supplement: Supplementary file 4 — Source data Fig. 1 [file 44319_2026_791_MOESM4_ESM.zip › Figure 1/B/-PA14-ISC-EB.png]

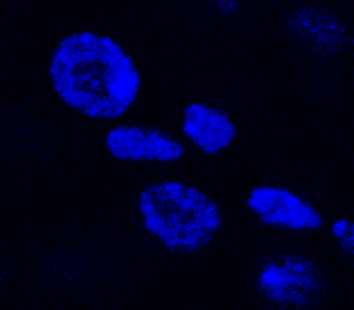

Supplement: Supplementary file 4 — Source data Fig. 1 [file 44319_2026_791_MOESM4_ESM.zip › Figure 1/B/-PA14-dapi.png]

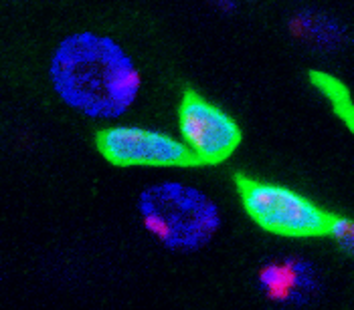

Supplement: Supplementary file 4 — Source data Fig. 1 [file 44319_2026_791_MOESM4_ESM.zip › Figure 1/B/-PA14-merge.png]

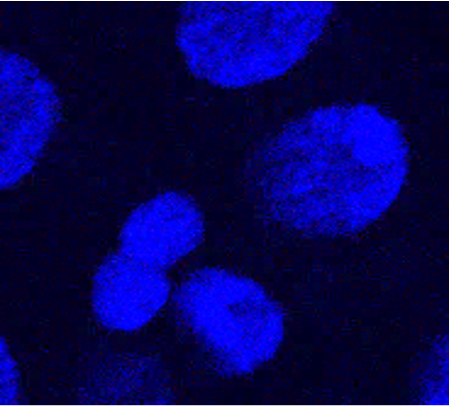

Supplement: Supplementary file 4 — Source data Fig. 1 [file 44319_2026_791_MOESM4_ESM.zip › Figure 1/D/+PA14-DAPI.png]

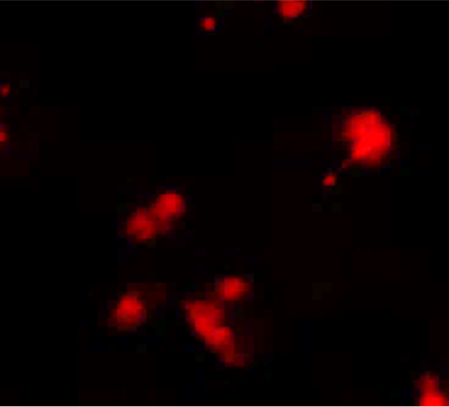

Supplement: Supplementary file 4 — Source data Fig. 1 [file 44319_2026_791_MOESM4_ESM.zip › Figure 1/D/+PA14-H3K9me3.png]

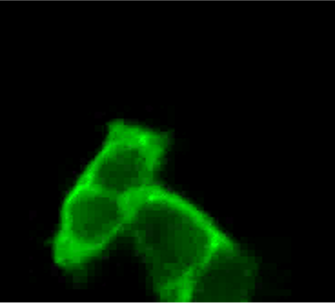

Supplement: Supplementary file 4 — Source data Fig. 1 [file 44319_2026_791_MOESM4_ESM.zip › Figure 1/D/+PA14-ISC-EB.png]

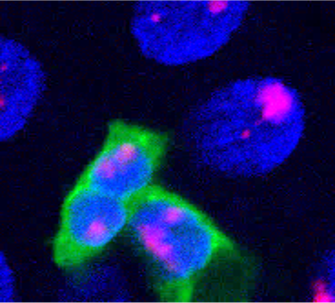

Supplement: Supplementary file 4 — Source data Fig. 1 [file 44319_2026_791_MOESM4_ESM.zip › Figure 1/D/+PA14-merge.png]

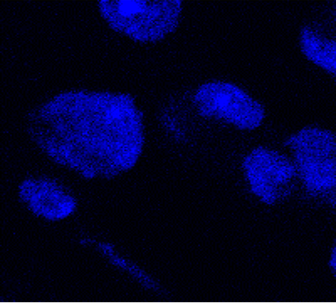

Supplement: Supplementary file 4 — Source data Fig. 1 [file 44319_2026_791_MOESM4_ESM.zip › Figure 1/D/-PA14-DAPI.png]

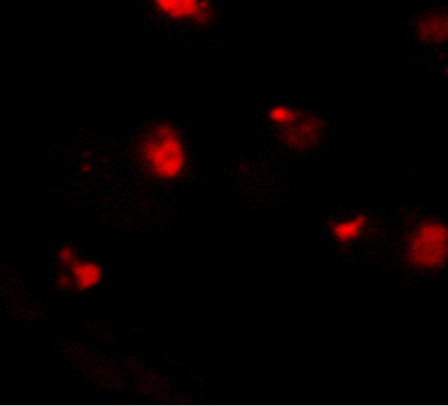

Supplement: Supplementary file 4 — Source data Fig. 1 [file 44319_2026_791_MOESM4_ESM.zip › Figure 1/D/-PA14-H3K9me3.png]

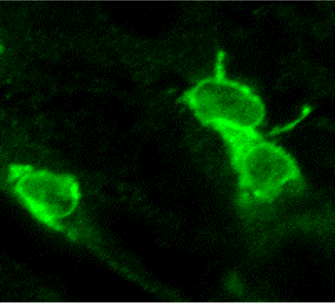

Supplement: Supplementary file 4 — Source data Fig. 1 [file 44319_2026_791_MOESM4_ESM.zip › Figure 1/D/-PA14-ISC-EB.png]

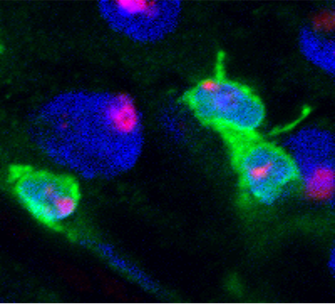

Supplement: Supplementary file 4 — Source data Fig. 1 [file 44319_2026_791_MOESM4_ESM.zip › Figure 1/D/-PA14-merge.png]

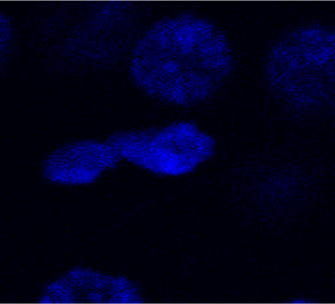

Supplement: Supplementary file 4 — Source data Fig. 1 [file 44319_2026_791_MOESM4_ESM.zip › Figure 1/F/OLD-DAPI.png]

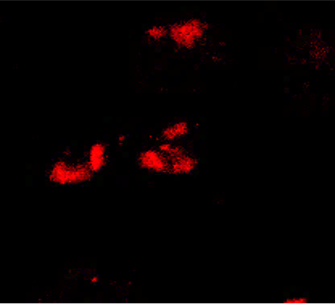

Supplement: Supplementary file 4 — Source data Fig. 1 [file 44319_2026_791_MOESM4_ESM.zip › Figure 1/F/OLD-HP1a.png]

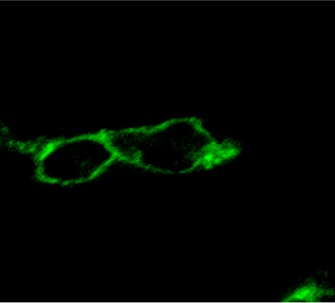

Supplement: Supplementary file 4 — Source data Fig. 1 [file 44319_2026_791_MOESM4_ESM.zip › Figure 1/F/OLD-ISC-EB.png]

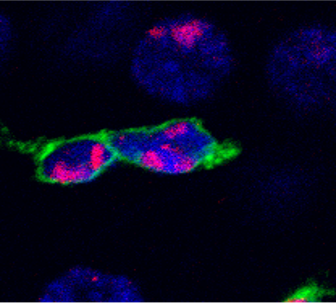

Supplement: Supplementary file 4 — Source data Fig. 1 [file 44319_2026_791_MOESM4_ESM.zip › Figure 1/F/OLD-MERGE.png]

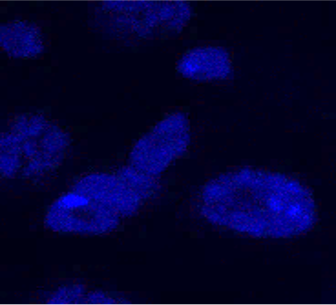

Supplement: Supplementary file 4 — Source data Fig. 1 [file 44319_2026_791_MOESM4_ESM.zip › Figure 1/F/YOUNG-DAPI.png]

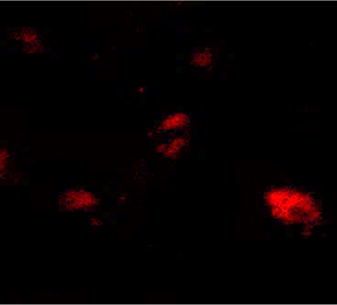

Supplement: Supplementary file 4 — Source data Fig. 1 [file 44319_2026_791_MOESM4_ESM.zip › Figure 1/F/YOUNG-HP1a.png]

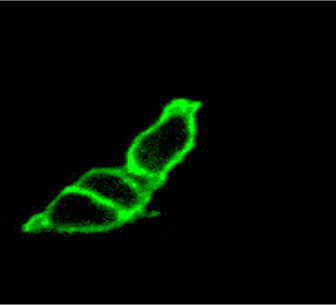

Supplement: Supplementary file 4 — Source data Fig. 1 [file 44319_2026_791_MOESM4_ESM.zip › Figure 1/F/YOUNG-ISC-EB.png]

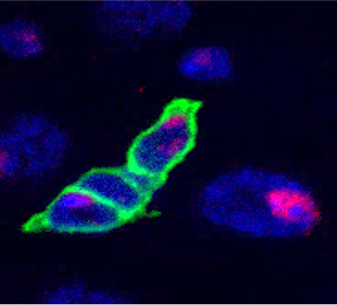

Supplement: Supplementary file 4 — Source data Fig. 1 [file 44319_2026_791_MOESM4_ESM.zip › Figure 1/F/YOUNG-MERGE.png]

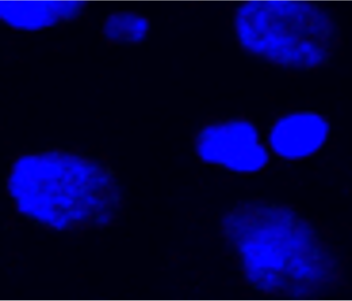

Supplement: Supplementary file 4 — Source data Fig. 1 [file 44319_2026_791_MOESM4_ESM.zip › Figure 1/H/OLD-DAPI.png]

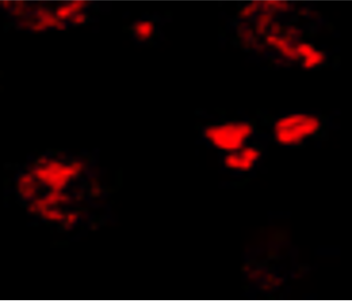

Supplement: Supplementary file 4 — Source data Fig. 1 [file 44319_2026_791_MOESM4_ESM.zip › Figure 1/H/OLD-H3K9me3.png]

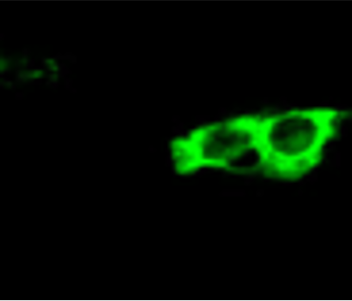

Supplement: Supplementary file 4 — Source data Fig. 1 [file 44319_2026_791_MOESM4_ESM.zip › Figure 1/H/OLD-ISC-EB.png]

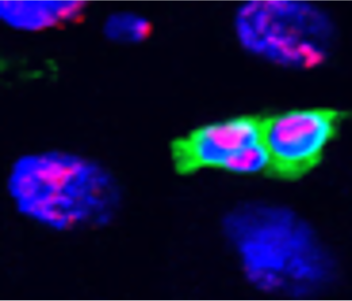

Supplement: Supplementary file 4 — Source data Fig. 1 [file 44319_2026_791_MOESM4_ESM.zip › Figure 1/H/OLD-MERGE.png]

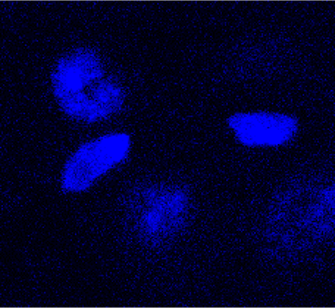

Supplement: Supplementary file 4 — Source data Fig. 1 [file 44319_2026_791_MOESM4_ESM.zip › Figure 1/H/YOUNG-DAPI.png]

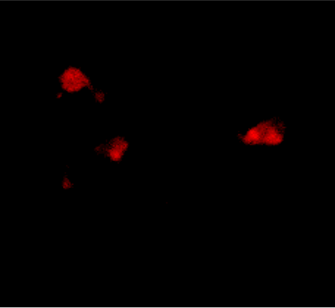

Supplement: Supplementary file 4 — Source data Fig. 1 [file 44319_2026_791_MOESM4_ESM.zip › Figure 1/H/YOUNG-H3K9me3.png]

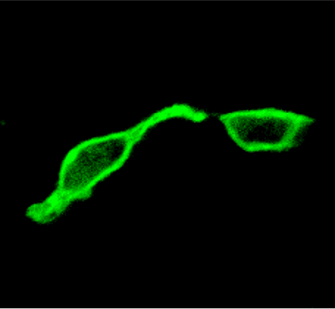

Supplement: Supplementary file 4 — Source data Fig. 1 [file 44319_2026_791_MOESM4_ESM.zip › Figure 1/H/YOUNG-ISC-EB.png]

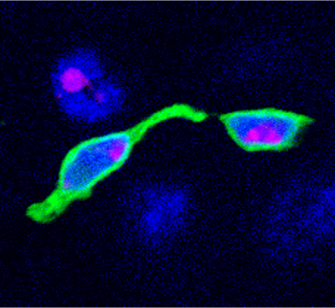

Supplement: Supplementary file 4 — Source data Fig. 1 [file 44319_2026_791_MOESM4_ESM.zip › Figure 1/H/YOUNG-MERGE.png]

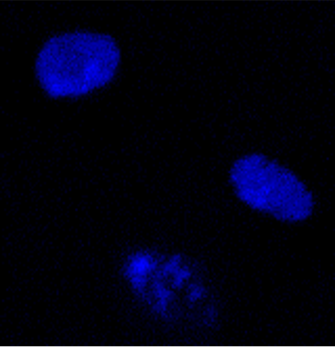

Supplement: Supplementary file 6 — Source data Fig. 3 [file 44319_2026_791_MOESM6_ESM.zip › Figure 3/A/Control-dapi.png]

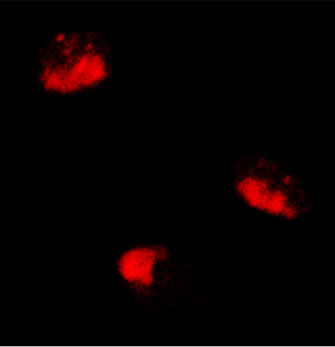

Supplement: Supplementary file 6 — Source data Fig. 3 [file 44319_2026_791_MOESM6_ESM.zip › Figure 3/A/Control-HP1a.png]

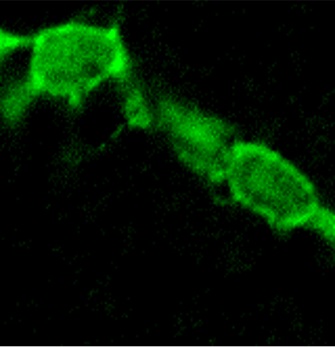

Supplement: Supplementary file 6 — Source data Fig. 3 [file 44319_2026_791_MOESM6_ESM.zip › Figure 3/A/Control-ISC-EB.png]

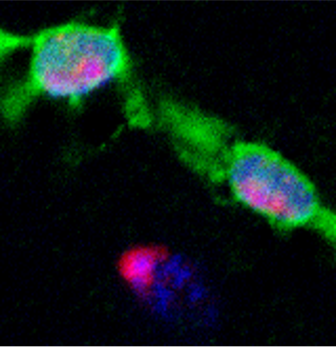

Supplement: Supplementary file 6 — Source data Fig. 3 [file 44319_2026_791_MOESM6_ESM.zip › Figure 3/A/Control-merge.png]

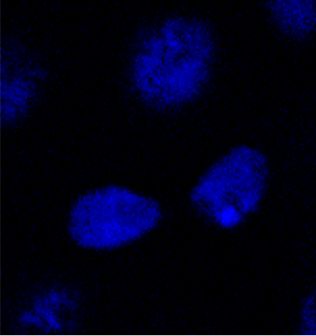

Supplement: Supplementary file 6 — Source data Fig. 3 [file 44319_2026_791_MOESM6_ESM.zip › Figure 3/A/rox2 OE-DAPI.png]

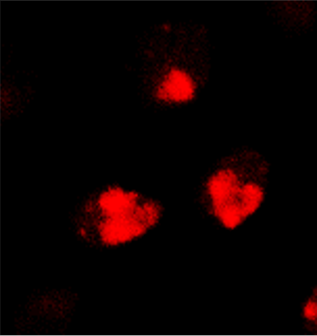

Supplement: Supplementary file 6 — Source data Fig. 3 [file 44319_2026_791_MOESM6_ESM.zip › Figure 3/A/rox2 OE-HP1a.png]

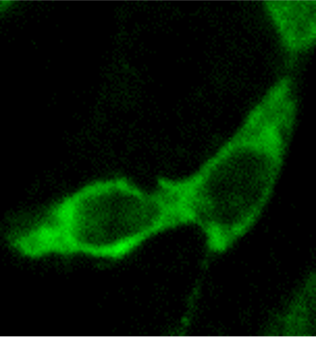

Supplement: Supplementary file 6 — Source data Fig. 3 [file 44319_2026_791_MOESM6_ESM.zip › Figure 3/A/rox2 OE-ISC-EB.png]

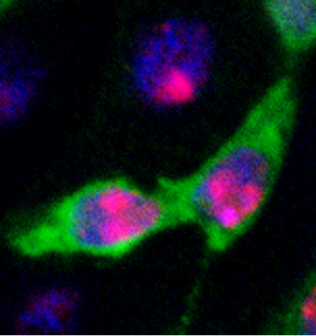

Supplement: Supplementary file 6 — Source data Fig. 3 [file 44319_2026_791_MOESM6_ESM.zip › Figure 3/A/rox2 OE-MERGE.png]

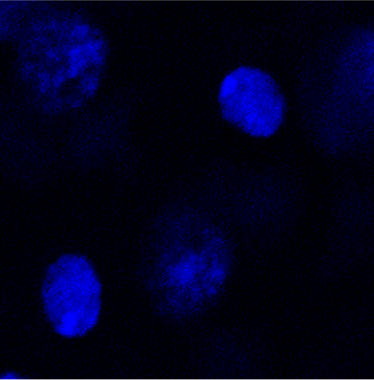

Supplement: Supplementary file 6 — Source data Fig. 3 [file 44319_2026_791_MOESM6_ESM.zip › Figure 3/C/Control-dapi.png]

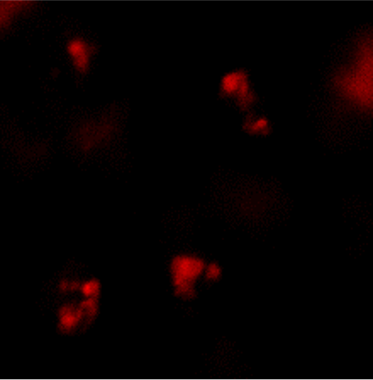

Supplement: Supplementary file 6 — Source data Fig. 3 [file 44319_2026_791_MOESM6_ESM.zip › Figure 3/C/Control-H3K9me3.png]

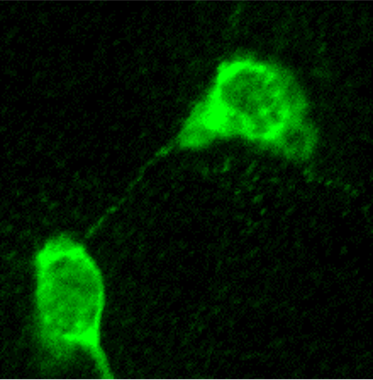

Supplement: Supplementary file 6 — Source data Fig. 3 [file 44319_2026_791_MOESM6_ESM.zip › Figure 3/C/Control-ISC-EB.png]

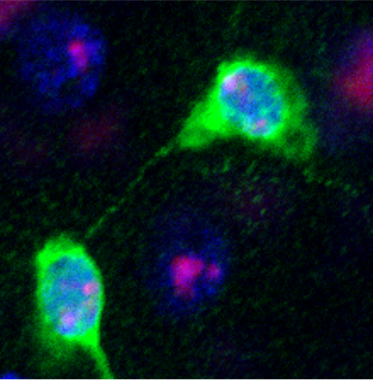

Supplement: Supplementary file 6 — Source data Fig. 3 [file 44319_2026_791_MOESM6_ESM.zip › Figure 3/C/Control-merge.png]

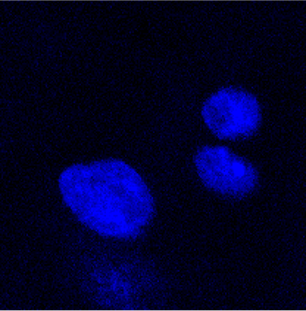

Supplement: Supplementary file 6 — Source data Fig. 3 [file 44319_2026_791_MOESM6_ESM.zip › Figure 3/C/roX2 OE-dapi.png]

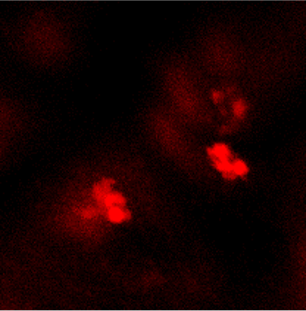

Supplement: Supplementary file 6 — Source data Fig. 3 [file 44319_2026_791_MOESM6_ESM.zip › Figure 3/C/roX2 OE-H3K9me3.png]

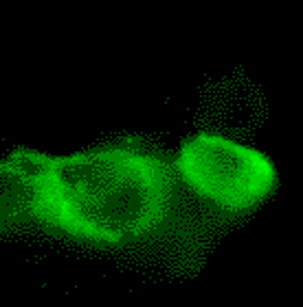

Supplement: Supplementary file 6 — Source data Fig. 3 [file 44319_2026_791_MOESM6_ESM.zip › Figure 3/C/roX2 OE-ISC-EB.png]

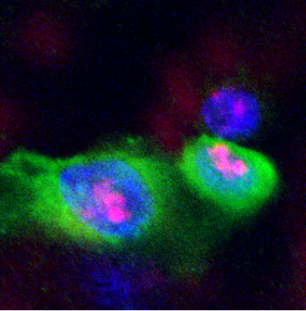

Supplement: Supplementary file 6 — Source data Fig. 3 [file 44319_2026_791_MOESM6_ESM.zip › Figure 3/C/roX2 OE-merge.png]

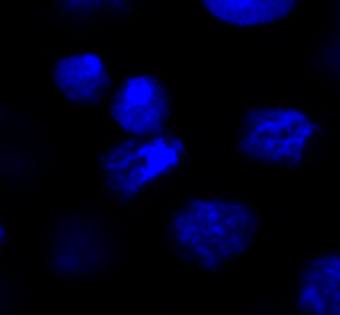

Supplement: Supplementary file 6 — Source data Fig. 3 [file 44319_2026_791_MOESM6_ESM.zip › Figure 3/E/+PA14-Crl RNAi-dapi.tif]

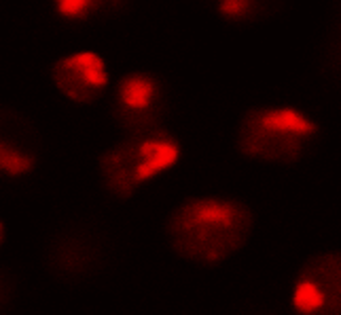

Supplement: Supplementary file 6 — Source data Fig. 3 [file 44319_2026_791_MOESM6_ESM.zip › Figure 3/E/+PA14-Crl RNAi-HP1a.tif]

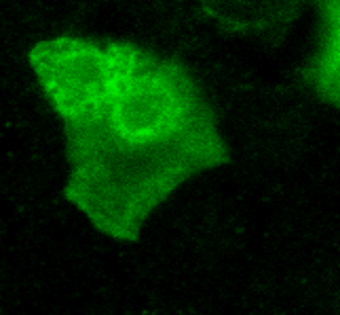

Supplement: Supplementary file 6 — Source data Fig. 3 [file 44319_2026_791_MOESM6_ESM.zip › Figure 3/E/+PA14-Crl RNAi-ISC-EB.tif]

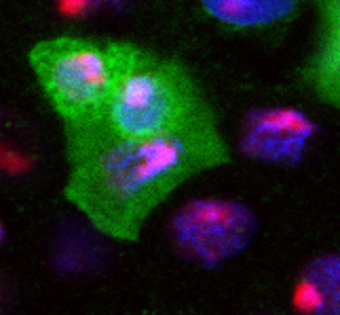

Supplement: Supplementary file 6 — Source data Fig. 3 [file 44319_2026_791_MOESM6_ESM.zip › Figure 3/E/+PA14-Crl RNAi-merge.tif]

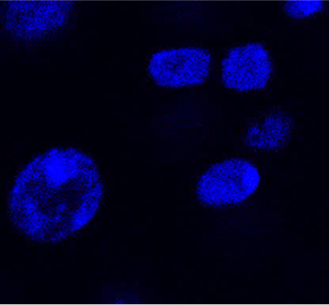

Supplement: Supplementary file 6 — Source data Fig. 3 [file 44319_2026_791_MOESM6_ESM.zip › Figure 3/E/+PA14-roX1 RNAi-dapi.png]

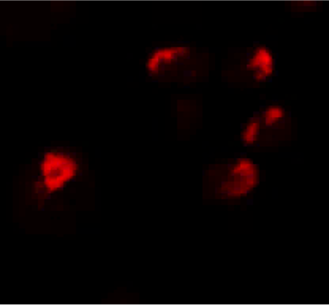

Supplement: Supplementary file 6 — Source data Fig. 3 [file 44319_2026_791_MOESM6_ESM.zip › Figure 3/E/+PA14-roX1 RNAi-HP1a.png]

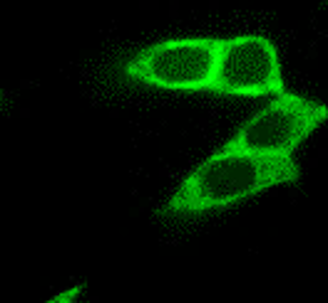

Supplement: Supplementary file 6 — Source data Fig. 3 [file 44319_2026_791_MOESM6_ESM.zip › Figure 3/E/+PA14-roX1 RNAi-ISC-EB.png]

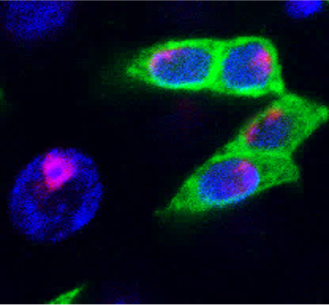

Supplement: Supplementary file 6 — Source data Fig. 3 [file 44319_2026_791_MOESM6_ESM.zip › Figure 3/E/+PA14-roX1 RNAi-merge.png]

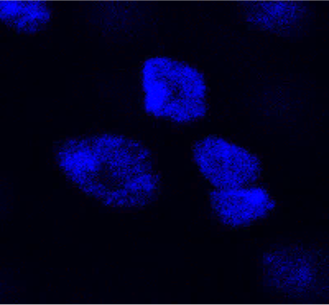

Supplement: Supplementary file 6 — Source data Fig. 3 [file 44319_2026_791_MOESM6_ESM.zip › Figure 3/E/+PA14-roX2 RNAi-dapi.png]

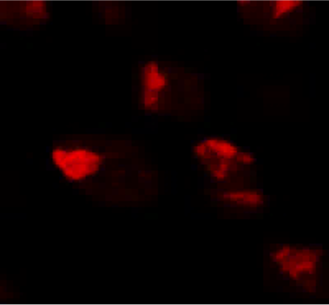

Supplement: Supplementary file 6 — Source data Fig. 3 [file 44319_2026_791_MOESM6_ESM.zip › Figure 3/E/+PA14-roX2 RNAi-HP1a.png]

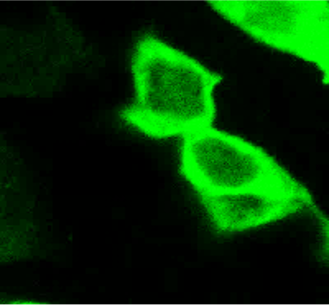

Supplement: Supplementary file 6 — Source data Fig. 3 [file 44319_2026_791_MOESM6_ESM.zip › Figure 3/E/+PA14-roX2 RNAi-ISC-EB.png]

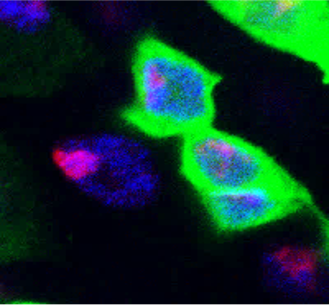

Supplement: Supplementary file 6 — Source data Fig. 3 [file 44319_2026_791_MOESM6_ESM.zip › Figure 3/E/+PA14-roX2 RNAi-merge.png]

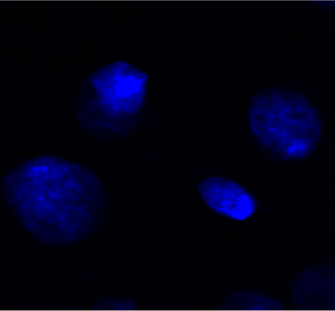

Supplement: Supplementary file 6 — Source data Fig. 3 [file 44319_2026_791_MOESM6_ESM.zip › Figure 3/E/-PA14 crl RNAi-dapi.png]

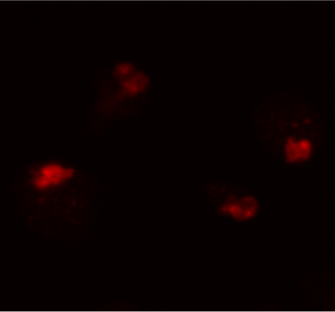

Supplement: Supplementary file 6 — Source data Fig. 3 [file 44319_2026_791_MOESM6_ESM.zip › Figure 3/E/-PA14 crl RNAi-HP1a.png]

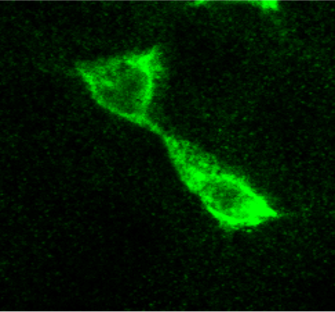

Supplement: Supplementary file 6 — Source data Fig. 3 [file 44319_2026_791_MOESM6_ESM.zip › Figure 3/E/-PA14 crl RNAi-ISC-EB.png]

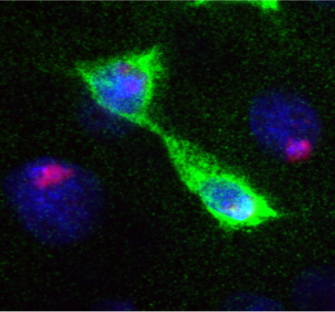

Supplement: Supplementary file 6 — Source data Fig. 3 [file 44319_2026_791_MOESM6_ESM.zip › Figure 3/E/-PA14 crl RNAi-merge.png]

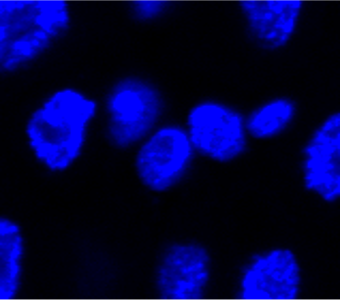

Supplement: Supplementary file 6 — Source data Fig. 3 [file 44319_2026_791_MOESM6_ESM.zip › Figure 3/F/+PA14 Crl RNAi-dapi.png]

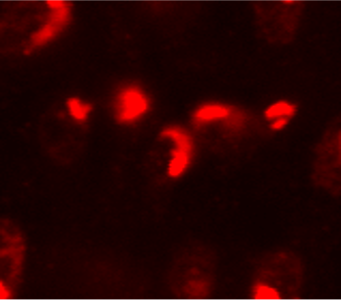

Supplement: Supplementary file 6 — Source data Fig. 3 [file 44319_2026_791_MOESM6_ESM.zip › Figure 3/F/+PA14 Crl RNAi-H3K9me3.png]

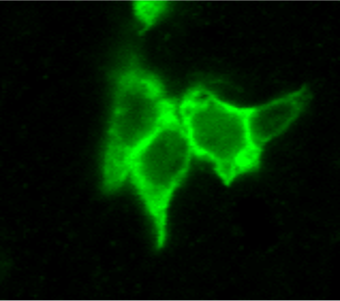

Supplement: Supplementary file 6 — Source data Fig. 3 [file 44319_2026_791_MOESM6_ESM.zip › Figure 3/F/+PA14 Crl RNAi-ISC-EB.png]

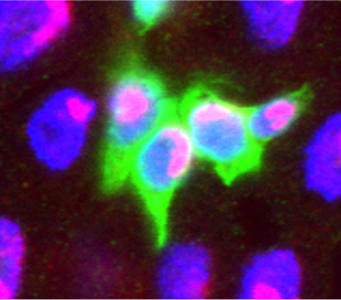

Supplement: Supplementary file 6 — Source data Fig. 3 [file 44319_2026_791_MOESM6_ESM.zip › Figure 3/F/+PA14 Crl RNAi-merge.png]

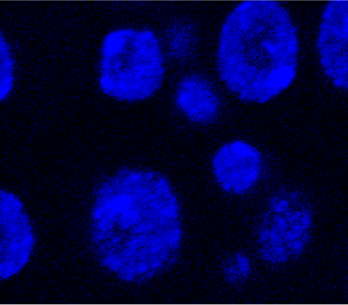

Supplement: Supplementary file 6 — Source data Fig. 3 [file 44319_2026_791_MOESM6_ESM.zip › Figure 3/F/+PA14 roX1 RNAi-dapi.png]

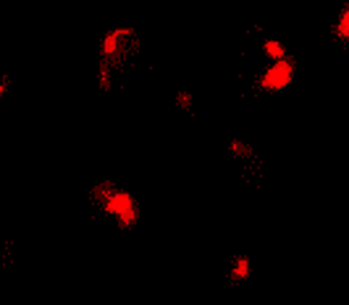

Supplement: Supplementary file 6 — Source data Fig. 3 [file 44319_2026_791_MOESM6_ESM.zip › Figure 3/F/+PA14 roX1 RNAi-H3K9me3.png]

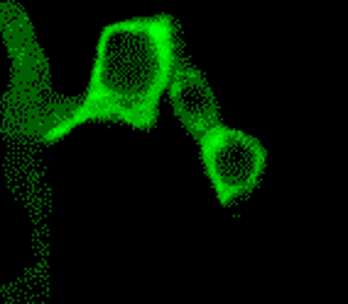

Supplement: Supplementary file 6 — Source data Fig. 3 [file 44319_2026_791_MOESM6_ESM.zip › Figure 3/F/+PA14 roX1 RNAi-ISC-EB.png]

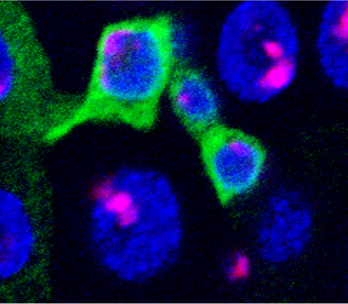

Supplement: Supplementary file 6 — Source data Fig. 3 [file 44319_2026_791_MOESM6_ESM.zip › Figure 3/F/+PA14 roX1 RNAi-merge.png]

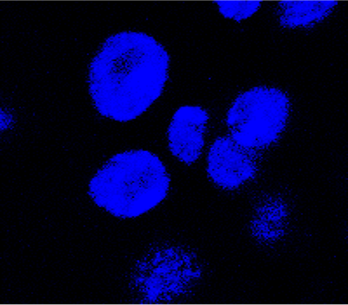

Supplement: Supplementary file 6 — Source data Fig. 3 [file 44319_2026_791_MOESM6_ESM.zip › Figure 3/F/+PA14 roX2 RNAi-dapi.png]

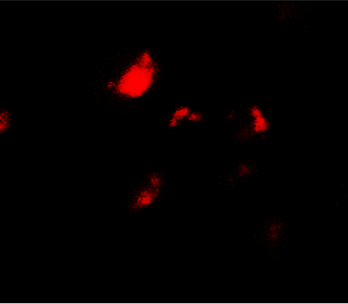

Supplement: Supplementary file 6 — Source data Fig. 3 [file 44319_2026_791_MOESM6_ESM.zip › Figure 3/F/+PA14 roX2 RNAi-H3K9me3.png]

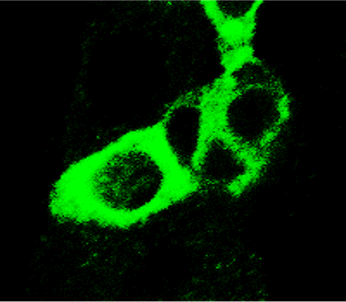

Supplement: Supplementary file 6 — Source data Fig. 3 [file 44319_2026_791_MOESM6_ESM.zip › Figure 3/F/+PA14 roX2 RNAi-ISC-EB.png]

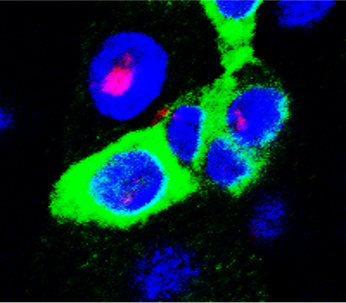

Supplement: Supplementary file 6 — Source data Fig. 3 [file 44319_2026_791_MOESM6_ESM.zip › Figure 3/F/+PA14 roX2 RNAi-merge.png]

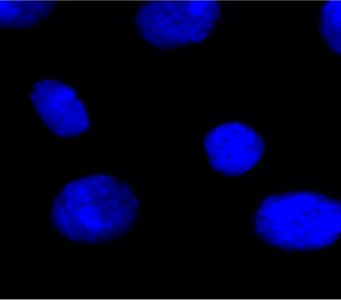

Supplement: Supplementary file 6 — Source data Fig. 3 [file 44319_2026_791_MOESM6_ESM.zip › Figure 3/F/-PA14 Crl RNAi-dapi.png]

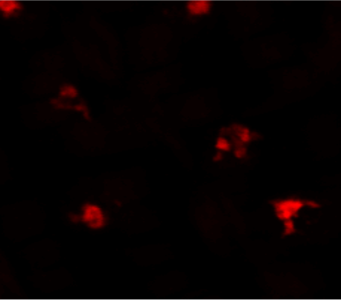

Supplement: Supplementary file 6 — Source data Fig. 3 [file 44319_2026_791_MOESM6_ESM.zip › Figure 3/F/-PA14 Crl RNAi-H3K9me3.png]

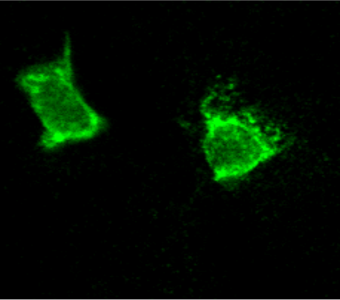

Supplement: Supplementary file 6 — Source data Fig. 3 [file 44319_2026_791_MOESM6_ESM.zip › Figure 3/F/-PA14 Crl RNAi-ISC-EB.png]

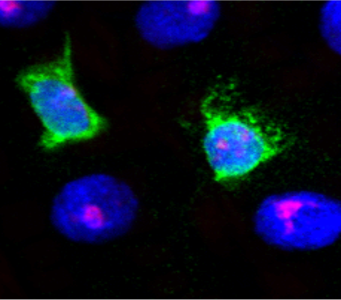

Supplement: Supplementary file 6 — Source data Fig. 3 [file 44319_2026_791_MOESM6_ESM.zip › Figure 3/F/-PA14 Crl RNAi-merge.png]

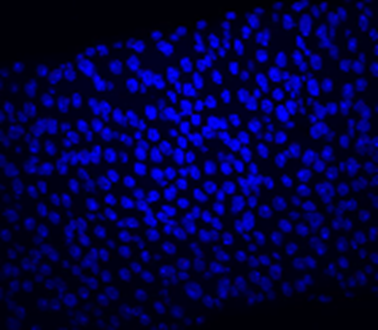

Supplement: Supplementary file 8 — Source data Fig. 5 [file 44319_2026_791_MOESM8_ESM.zip › Figure 5/A/+PA14 crl RNAi-DAPI.tif]

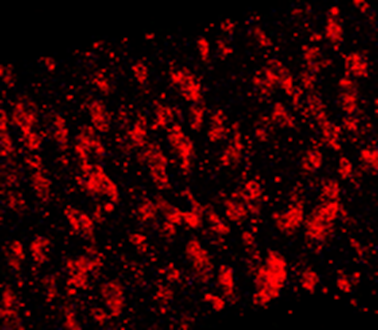

Supplement: Supplementary file 8 — Source data Fig. 5 [file 44319_2026_791_MOESM8_ESM.zip › Figure 5/A/+PA14 crl RNAi-Delta.tif]

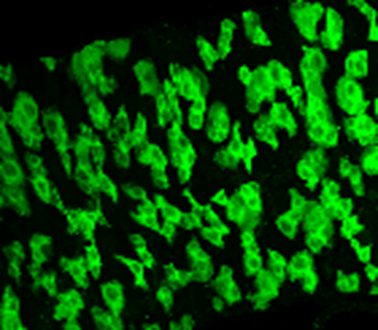

Supplement: Supplementary file 8 — Source data Fig. 5 [file 44319_2026_791_MOESM8_ESM.zip › Figure 5/A/+PA14 crl RNAi-ISC-EB.tif]

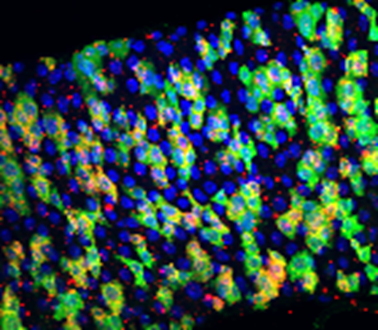

Supplement: Supplementary file 8 — Source data Fig. 5 [file 44319_2026_791_MOESM8_ESM.zip › Figure 5/A/+PA14 crl RNAi-MERGE.tif]

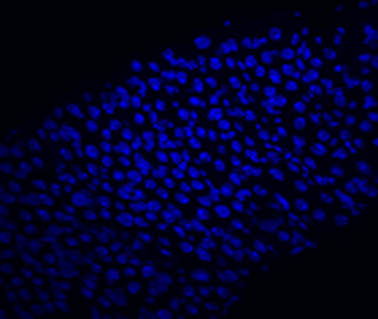

Supplement: Supplementary file 8 — Source data Fig. 5 [file 44319_2026_791_MOESM8_ESM.zip › Figure 5/A/+PA14 roX1 RNAi-DAPI.tif]

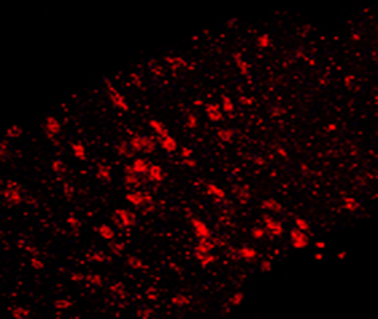

Supplement: Supplementary file 8 — Source data Fig. 5 [file 44319_2026_791_MOESM8_ESM.zip › Figure 5/A/+PA14 roX1 RNAi-Delta.tif]

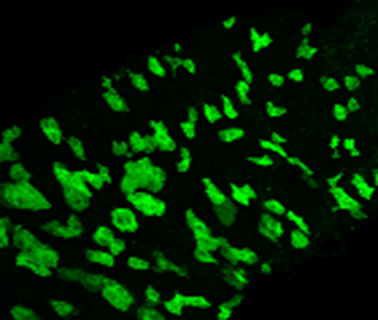

Supplement: Supplementary file 8 — Source data Fig. 5 [file 44319_2026_791_MOESM8_ESM.zip › Figure 5/A/+PA14 roX1 RNAi-ISC-EB.tif]

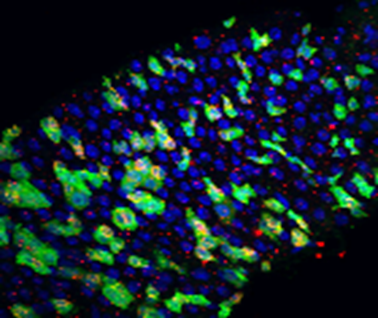

Supplement: Supplementary file 8 — Source data Fig. 5 [file 44319_2026_791_MOESM8_ESM.zip › Figure 5/A/+PA14 roX1 RNAi-MERGE.tif]

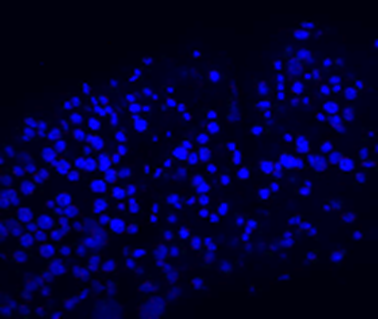

Supplement: Supplementary file 8 — Source data Fig. 5 [file 44319_2026_791_MOESM8_ESM.zip › Figure 5/A/+PA14 roX2 RNAi-DAPI.tif]

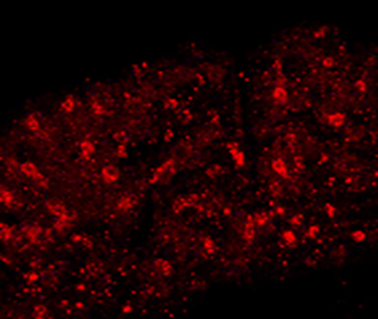

Supplement: Supplementary file 8 — Source data Fig. 5 [file 44319_2026_791_MOESM8_ESM.zip › Figure 5/A/+PA14 roX2 RNAi-Delta.tif]

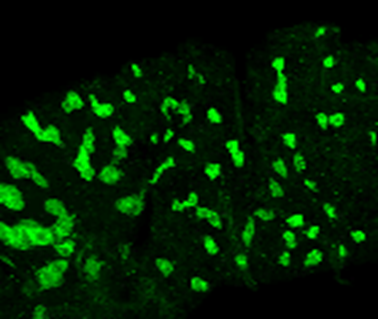

Supplement: Supplementary file 8 — Source data Fig. 5 [file 44319_2026_791_MOESM8_ESM.zip › Figure 5/A/+PA14 roX2 RNAi-ISC-EB.tif]

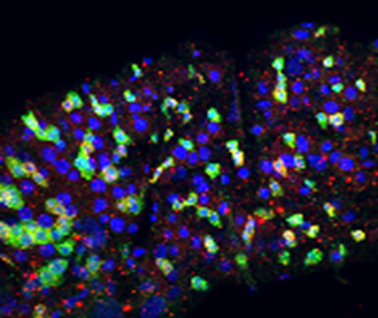

Supplement: Supplementary file 8 — Source data Fig. 5 [file 44319_2026_791_MOESM8_ESM.zip › Figure 5/A/+PA14 roX2 RNAi-MERGE.tif]

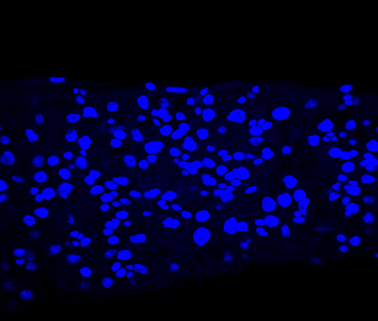

Supplement: Supplementary file 8 — Source data Fig. 5 [file 44319_2026_791_MOESM8_ESM.zip › Figure 5/A/+PA14 roX2 RNAi;roX2 OE-DAPI.tif]

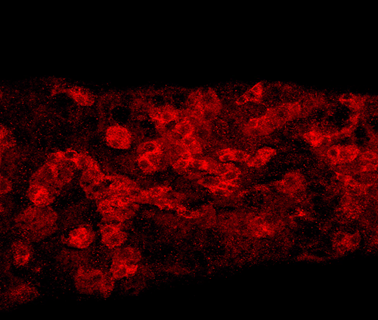

Supplement: Supplementary file 8 — Source data Fig. 5 [file 44319_2026_791_MOESM8_ESM.zip › Figure 5/A/+PA14 roX2 RNAi;roX2 OE-Delta.tif]

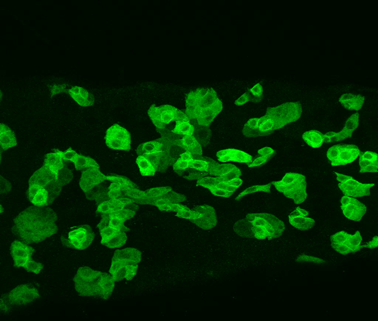

Supplement: Supplementary file 8 — Source data Fig. 5 [file 44319_2026_791_MOESM8_ESM.zip › Figure 5/A/+PA14 roX2 RNAi;roX2 OE-ISC-EB.tif]

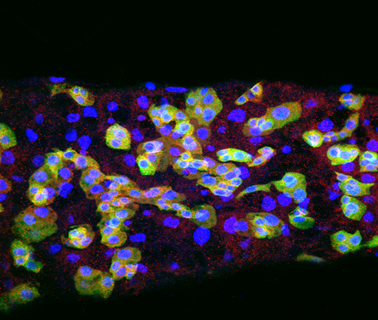

Supplement: Supplementary file 8 — Source data Fig. 5 [file 44319_2026_791_MOESM8_ESM.zip › Figure 5/A/+PA14 roX2 RNAi;roX2 OE-MERGE.tif]

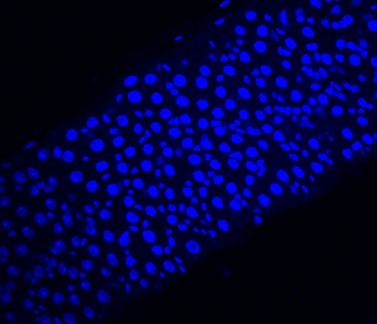

Supplement: Supplementary file 8 — Source data Fig. 5 [file 44319_2026_791_MOESM8_ESM.zip › Figure 5/A/-PA14 crl RNAi-DAPI.tif]

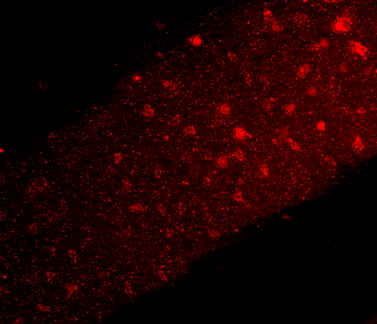

Supplement: Supplementary file 8 — Source data Fig. 5 [file 44319_2026_791_MOESM8_ESM.zip › Figure 5/A/-PA14 crl RNAi-Delta.tif]

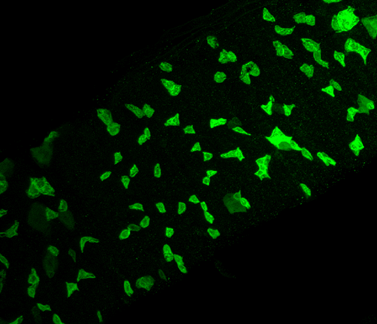

Supplement: Supplementary file 8 — Source data Fig. 5 [file 44319_2026_791_MOESM8_ESM.zip › Figure 5/A/-PA14 crl RNAi-ISC-EB.tif]

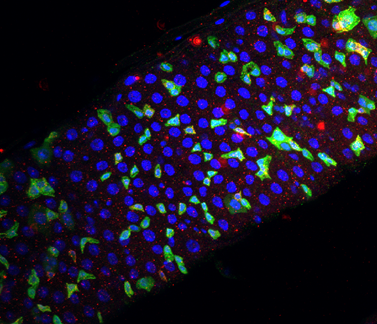

Supplement: Supplementary file 8 — Source data Fig. 5 [file 44319_2026_791_MOESM8_ESM.zip › Figure 5/A/-PA14 crl RNAi-MERGE.tif]
