# Supplementary figures and images for: roX1 and roX2 lncRNAs promote heterochromatinization in intestinal stem cells and impair longevity (part 2 of 3)
Source: EMBO Rep. 2026 May 9;27(12):3394–423. doi: 10.1038/s44319-026-00791-8 (PMC13303914; doi:10.1038/s44319-026-00791-8)

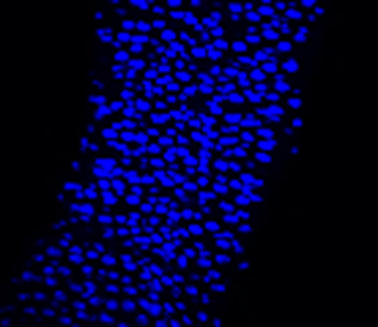

Supplement: Supplementary file 8 — Source data Fig. 5 [file 44319_2026_791_MOESM8_ESM.zip › Figure 5/A/-PA14 roX1 RNAi-DAPI.tif]

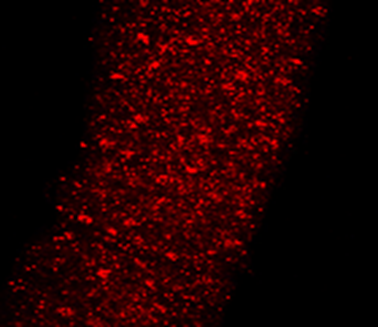

Supplement: Supplementary file 8 — Source data Fig. 5 [file 44319_2026_791_MOESM8_ESM.zip › Figure 5/A/-PA14 roX1 RNAi-Delta.tif]

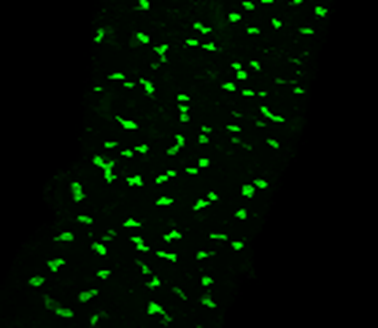

Supplement: Supplementary file 8 — Source data Fig. 5 [file 44319_2026_791_MOESM8_ESM.zip › Figure 5/A/-PA14 roX1 RNAi-ISC-EB.tif]

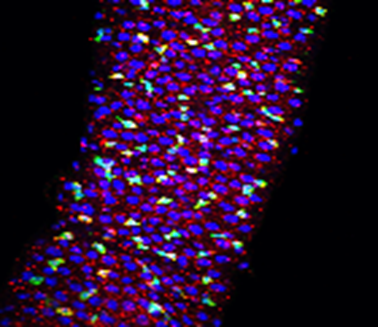

Supplement: Supplementary file 8 — Source data Fig. 5 [file 44319_2026_791_MOESM8_ESM.zip › Figure 5/A/-PA14 roX1 RNAi-MERGE.tif]

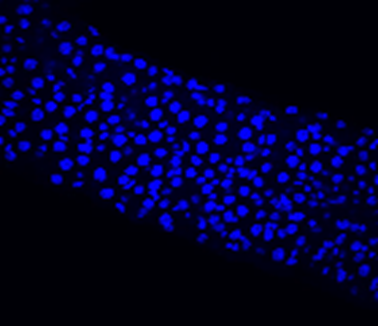

Supplement: Supplementary file 8 — Source data Fig. 5 [file 44319_2026_791_MOESM8_ESM.zip › Figure 5/A/-PA14 roX2 RNAi-DAPI.tif]

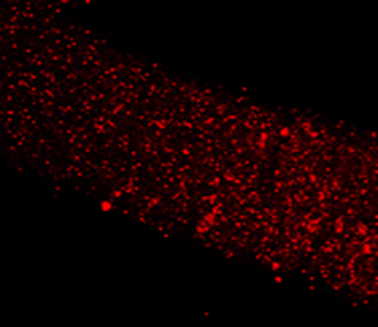

Supplement: Supplementary file 8 — Source data Fig. 5 [file 44319_2026_791_MOESM8_ESM.zip › Figure 5/A/-PA14 roX2 RNAi-Delta.tif]

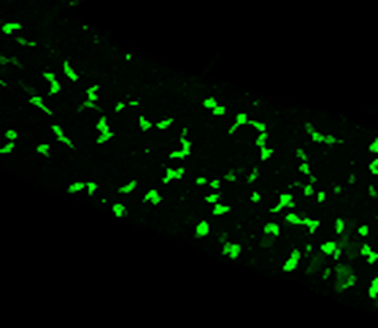

Supplement: Supplementary file 8 — Source data Fig. 5 [file 44319_2026_791_MOESM8_ESM.zip › Figure 5/A/-PA14 roX2 RNAi-ISC-EB.tif]

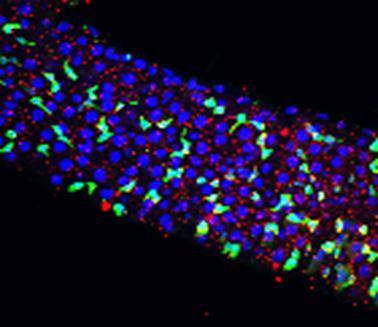

Supplement: Supplementary file 8 — Source data Fig. 5 [file 44319_2026_791_MOESM8_ESM.zip › Figure 5/A/-PA14 roX2 RNAi-MERGE.tif]

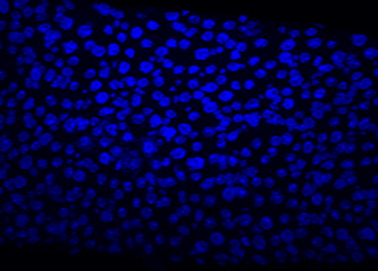

Supplement: Supplementary file 8 — Source data Fig. 5 [file 44319_2026_791_MOESM8_ESM.zip › Figure 5/D/+PA14 Crl RNAi-DAPI.tif]

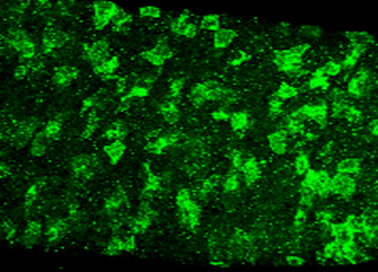

Supplement: Supplementary file 8 — Source data Fig. 5 [file 44319_2026_791_MOESM8_ESM.zip › Figure 5/D/+PA14 Crl RNAi-Delta.tif]

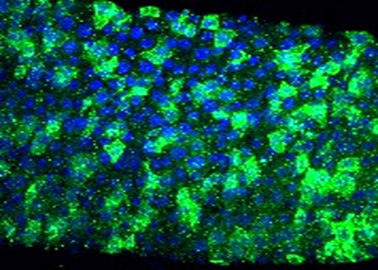

Supplement: Supplementary file 8 — Source data Fig. 5 [file 44319_2026_791_MOESM8_ESM.zip › Figure 5/D/+PA14 Crl RNAi-MERGE.tif]

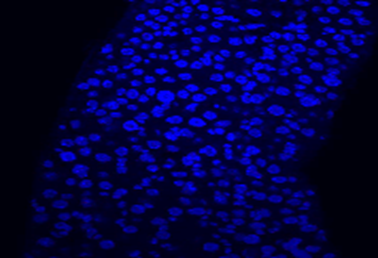

Supplement: Supplementary file 8 — Source data Fig. 5 [file 44319_2026_791_MOESM8_ESM.zip › Figure 5/D/+PA14 rox1 RNAi-DAPI.tif]

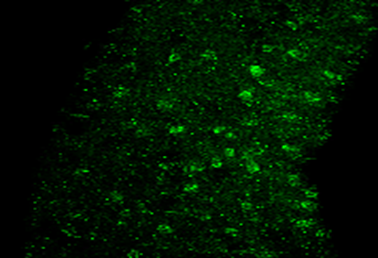

Supplement: Supplementary file 8 — Source data Fig. 5 [file 44319_2026_791_MOESM8_ESM.zip › Figure 5/D/+PA14 rox1 RNAi-Delta.tif]

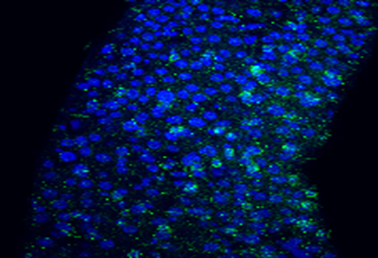

Supplement: Supplementary file 8 — Source data Fig. 5 [file 44319_2026_791_MOESM8_ESM.zip › Figure 5/D/+PA14 rox1 RNAi-MERGE.tif]

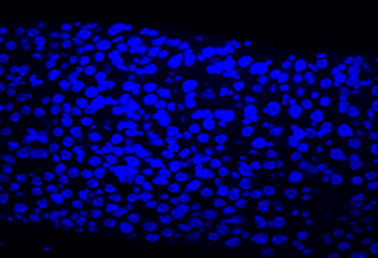

Supplement: Supplementary file 8 — Source data Fig. 5 [file 44319_2026_791_MOESM8_ESM.zip › Figure 5/D/+PA14 rox2 RNAi-DAPI.tif]

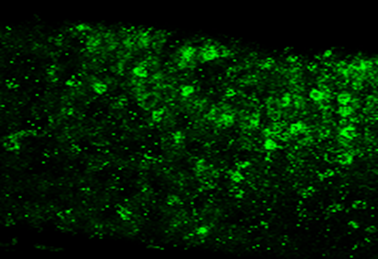

Supplement: Supplementary file 8 — Source data Fig. 5 [file 44319_2026_791_MOESM8_ESM.zip › Figure 5/D/+PA14 rox2 RNAi-Delta.tif]

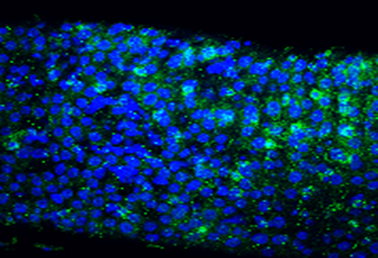

Supplement: Supplementary file 8 — Source data Fig. 5 [file 44319_2026_791_MOESM8_ESM.zip › Figure 5/D/+PA14 rox2 RNAi-MERGE.tif]

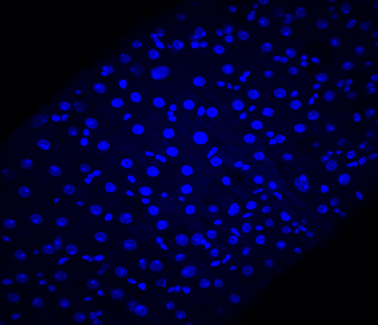

Supplement: Supplementary file 8 — Source data Fig. 5 [file 44319_2026_791_MOESM8_ESM.zip › Figure 5/F/Control-DAPI.tif]

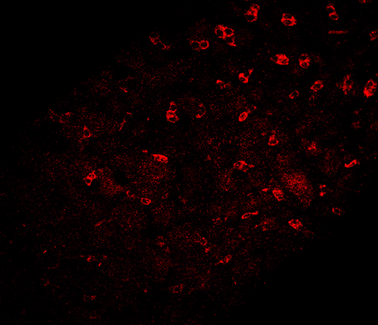

Supplement: Supplementary file 8 — Source data Fig. 5 [file 44319_2026_791_MOESM8_ESM.zip › Figure 5/F/Control-Delta.tif]

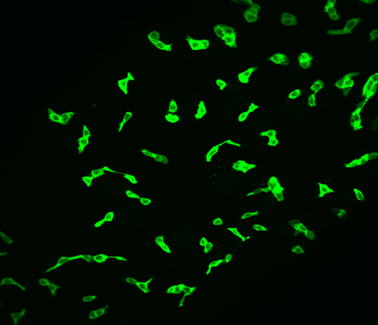

Supplement: Supplementary file 8 — Source data Fig. 5 [file 44319_2026_791_MOESM8_ESM.zip › Figure 5/F/Control-ISC-EB.tif]

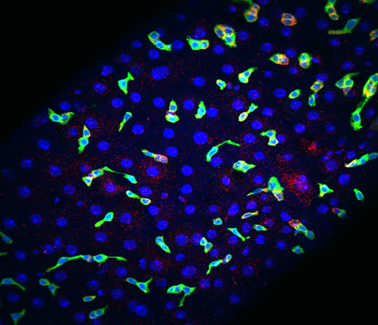

Supplement: Supplementary file 8 — Source data Fig. 5 [file 44319_2026_791_MOESM8_ESM.zip › Figure 5/F/Control-MERGE.tif]

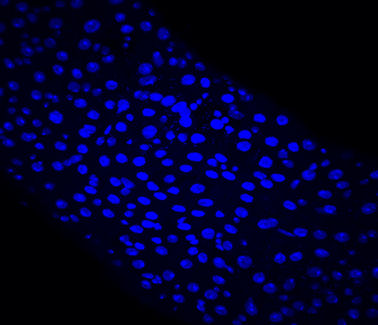

Supplement: Supplementary file 8 — Source data Fig. 5 [file 44319_2026_791_MOESM8_ESM.zip › Figure 5/F/roX2 OE-DAPI.tif]

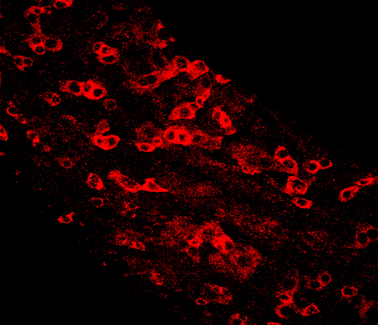

Supplement: Supplementary file 8 — Source data Fig. 5 [file 44319_2026_791_MOESM8_ESM.zip › Figure 5/F/roX2 OE-Delta.tif]

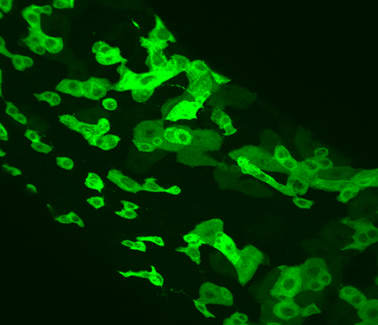

Supplement: Supplementary file 8 — Source data Fig. 5 [file 44319_2026_791_MOESM8_ESM.zip › Figure 5/F/roX2 OE-ISC-EB.tif]

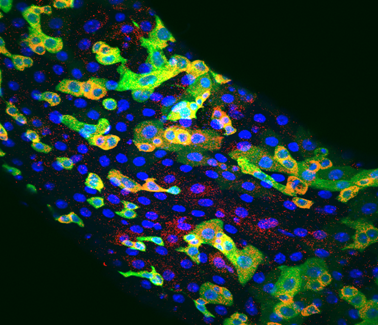

Supplement: Supplementary file 8 — Source data Fig. 5 [file 44319_2026_791_MOESM8_ESM.zip › Figure 5/F/roX2 OE-MERGE.tif]

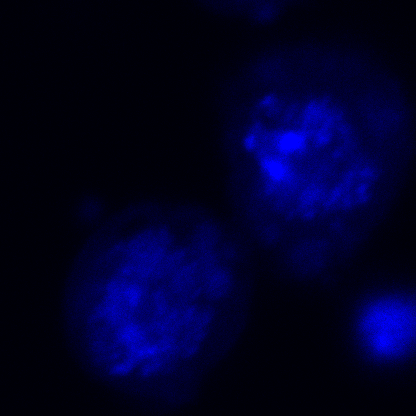

Supplement: Supplementary file 9 — Source data Fig. 6 [file 44319_2026_791_MOESM9_ESM.zip › Figure 6/A/dmso-crl shrna-DAPI.tif]

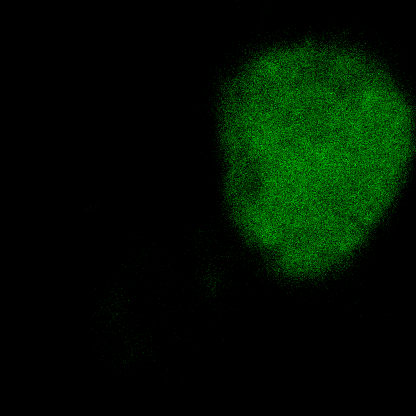

Supplement: Supplementary file 9 — Source data Fig. 6 [file 44319_2026_791_MOESM9_ESM.zip › Figure 6/A/dmso-crl shrna-GFP.tif]

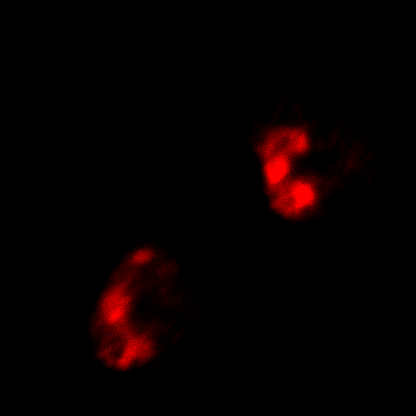

Supplement: Supplementary file 9 — Source data Fig. 6 [file 44319_2026_791_MOESM9_ESM.zip › Figure 6/A/dmso-crl shrna-hp1a.tif]

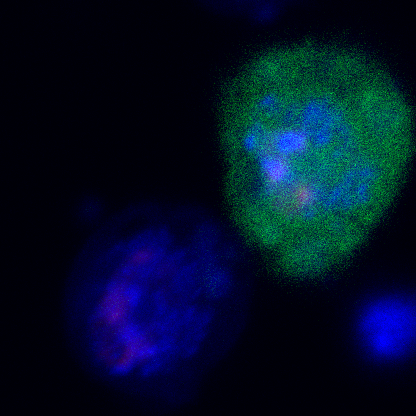

Supplement: Supplementary file 9 — Source data Fig. 6 [file 44319_2026_791_MOESM9_ESM.zip › Figure 6/A/dmso-crl shrna-MERGE.tif]

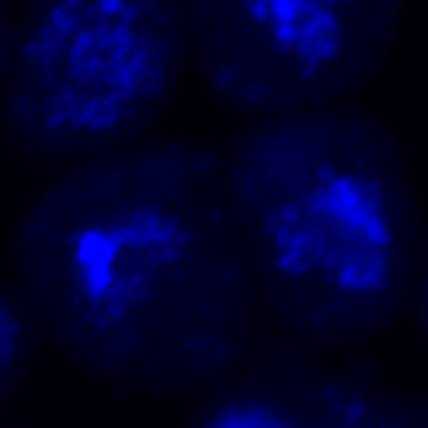

Supplement: Supplementary file 9 — Source data Fig. 6 [file 44319_2026_791_MOESM9_ESM.zip › Figure 6/A/lps-crl shrna-DAPI.tif]

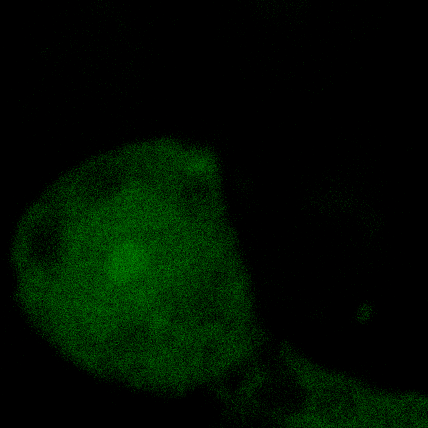

Supplement: Supplementary file 9 — Source data Fig. 6 [file 44319_2026_791_MOESM9_ESM.zip › Figure 6/A/lps-crl shrna-GFP.tif]

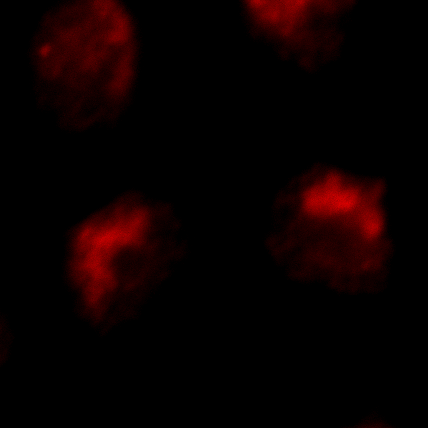

Supplement: Supplementary file 9 — Source data Fig. 6 [file 44319_2026_791_MOESM9_ESM.zip › Figure 6/A/lps-crl shrna-hp1a.tif]

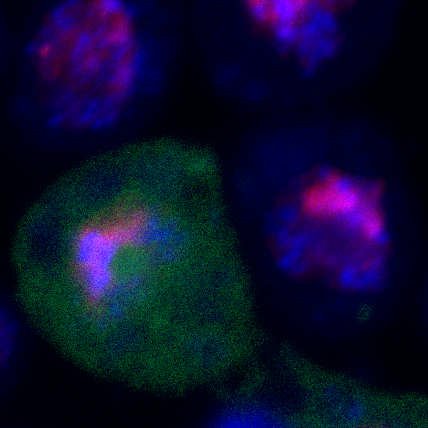

Supplement: Supplementary file 9 — Source data Fig. 6 [file 44319_2026_791_MOESM9_ESM.zip › Figure 6/A/lps-crl shrna-MERGE.tif]

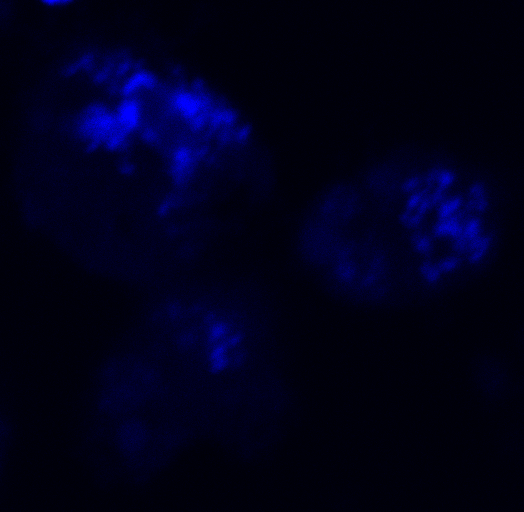

Supplement: Supplementary file 9 — Source data Fig. 6 [file 44319_2026_791_MOESM9_ESM.zip › Figure 6/A/lps-roX1 shrna-DAPI.tif]

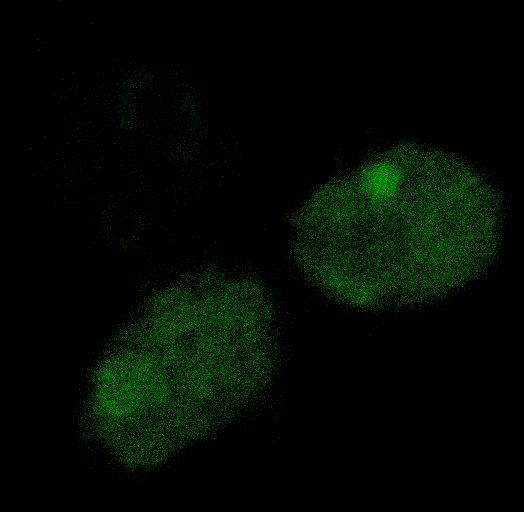

Supplement: Supplementary file 9 — Source data Fig. 6 [file 44319_2026_791_MOESM9_ESM.zip › Figure 6/A/lps-roX1 shrna-GFP.tif]

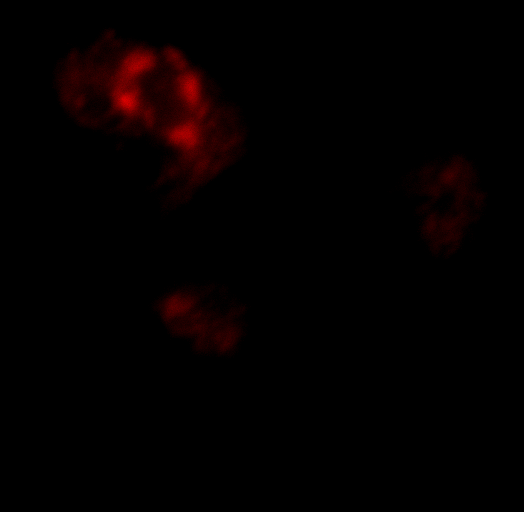

Supplement: Supplementary file 9 — Source data Fig. 6 [file 44319_2026_791_MOESM9_ESM.zip › Figure 6/A/lps-roX1 shrna-hp1a.tif]

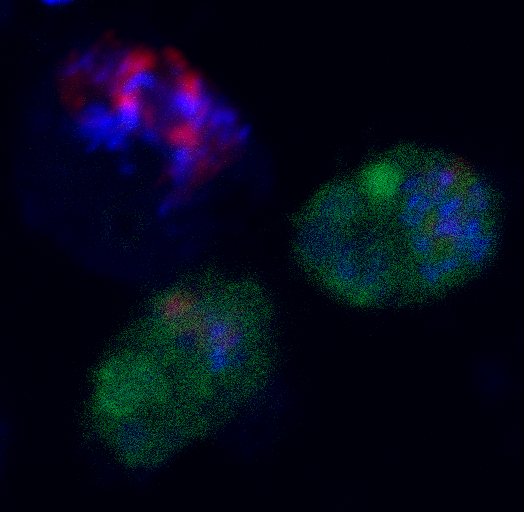

Supplement: Supplementary file 9 — Source data Fig. 6 [file 44319_2026_791_MOESM9_ESM.zip › Figure 6/A/lps-roX1 shrna-MERGE.tif]

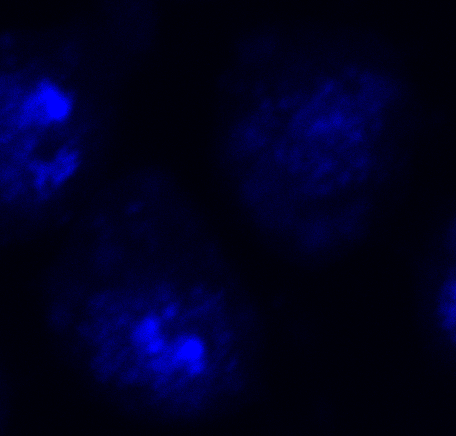

Supplement: Supplementary file 9 — Source data Fig. 6 [file 44319_2026_791_MOESM9_ESM.zip › Figure 6/A/lps-roX2 shrna-DAPI.tif]

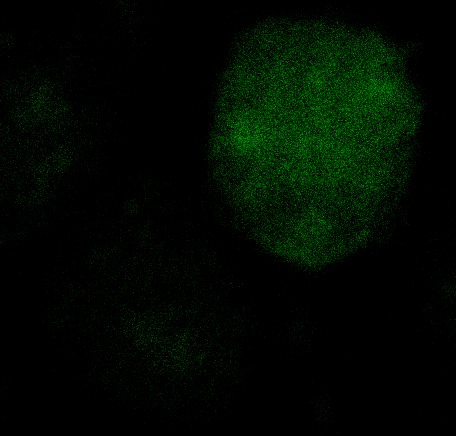

Supplement: Supplementary file 9 — Source data Fig. 6 [file 44319_2026_791_MOESM9_ESM.zip › Figure 6/A/lps-roX2 shrna-GFP.tif]

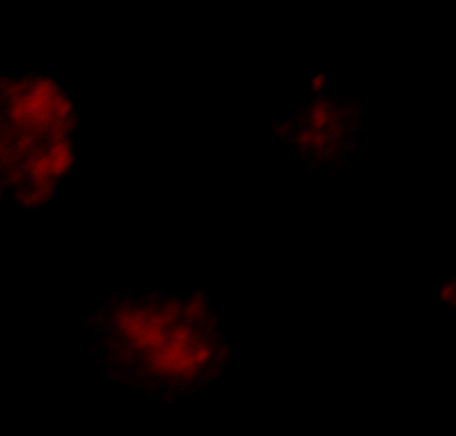

Supplement: Supplementary file 9 — Source data Fig. 6 [file 44319_2026_791_MOESM9_ESM.zip › Figure 6/A/lps-roX2 shrna-hp1a.tif]

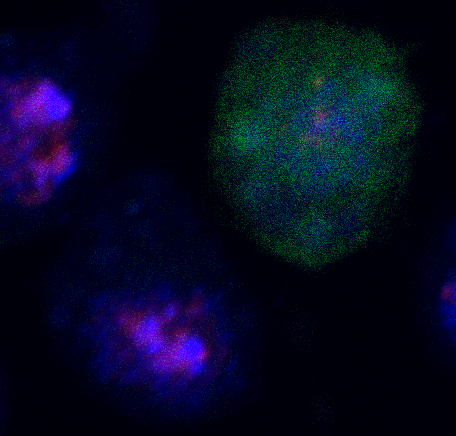

Supplement: Supplementary file 9 — Source data Fig. 6 [file 44319_2026_791_MOESM9_ESM.zip › Figure 6/A/lps-roX2 shrna-MERGE.tif]

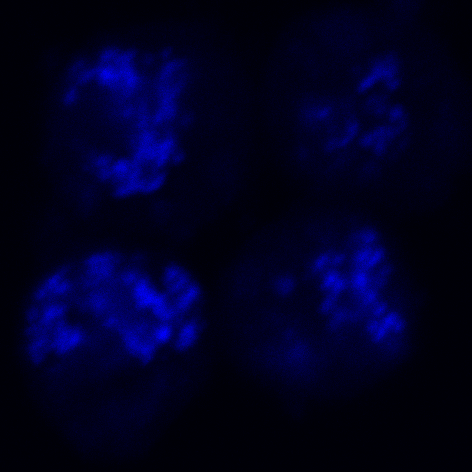

Supplement: Supplementary file 9 — Source data Fig. 6 [file 44319_2026_791_MOESM9_ESM.zip › Figure 6/B/dmso-crl shrna-DAPI.tif]

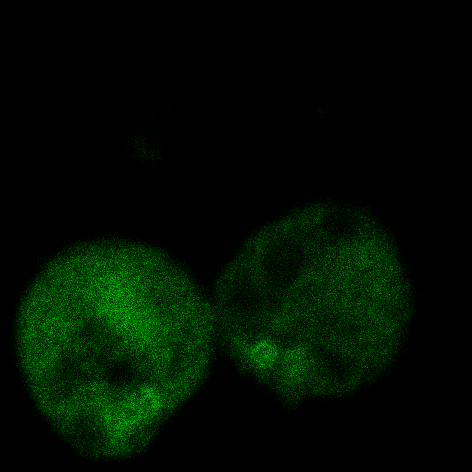

Supplement: Supplementary file 9 — Source data Fig. 6 [file 44319_2026_791_MOESM9_ESM.zip › Figure 6/B/dmso-crl shrna-GFP.tif]

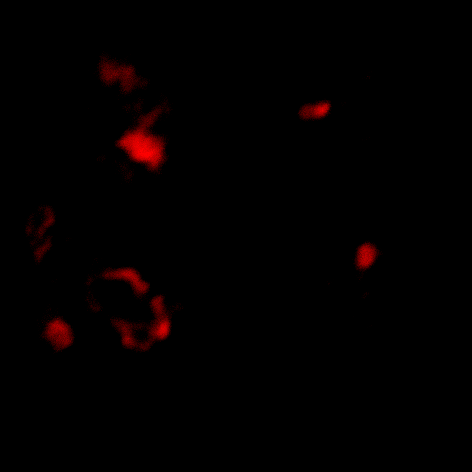

Supplement: Supplementary file 9 — Source data Fig. 6 [file 44319_2026_791_MOESM9_ESM.zip › Figure 6/B/dmso-crl shrna-k9me3.tif]

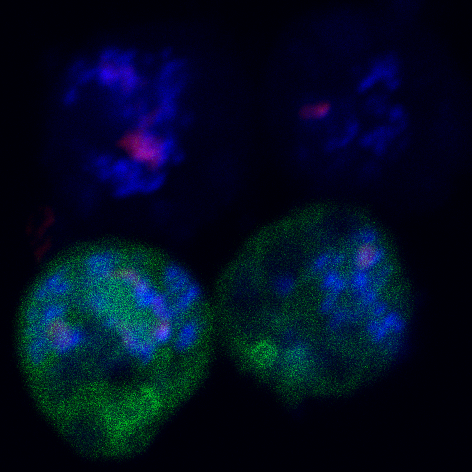

Supplement: Supplementary file 9 — Source data Fig. 6 [file 44319_2026_791_MOESM9_ESM.zip › Figure 6/B/dmso-crl shrna-MERGE.tif]

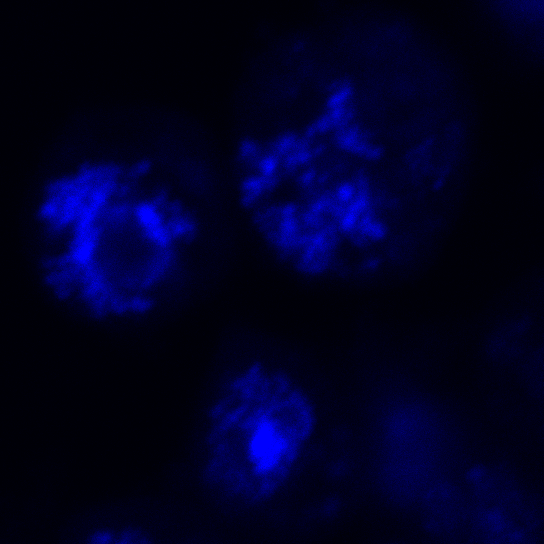

Supplement: Supplementary file 9 — Source data Fig. 6 [file 44319_2026_791_MOESM9_ESM.zip › Figure 6/B/lps-crl shrna-DAPI.tif]

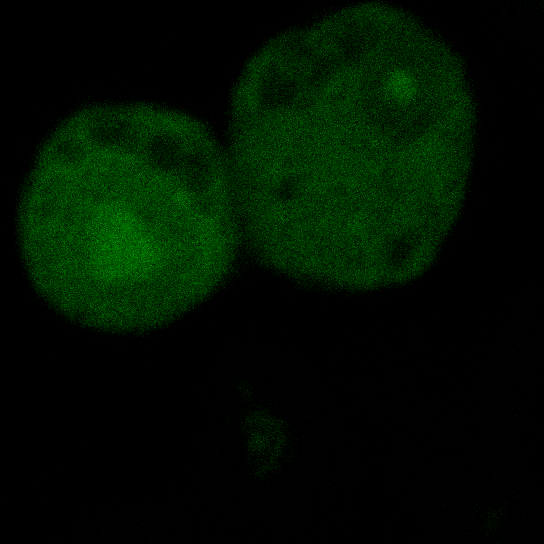

Supplement: Supplementary file 9 — Source data Fig. 6 [file 44319_2026_791_MOESM9_ESM.zip › Figure 6/B/lps-crl shrna-GFP.tif]

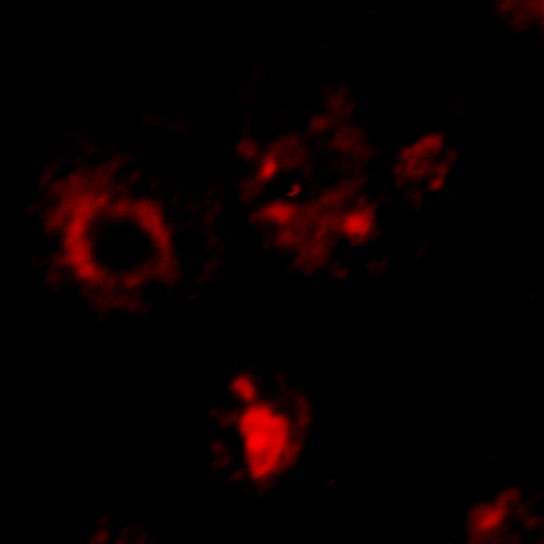

Supplement: Supplementary file 9 — Source data Fig. 6 [file 44319_2026_791_MOESM9_ESM.zip › Figure 6/B/lps-crl shrna-k9me3.tif]

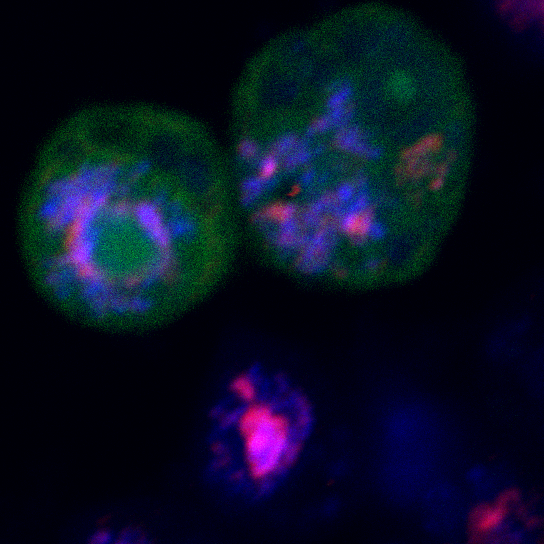

Supplement: Supplementary file 9 — Source data Fig. 6 [file 44319_2026_791_MOESM9_ESM.zip › Figure 6/B/lps-crl shrna-MERGE.tif]

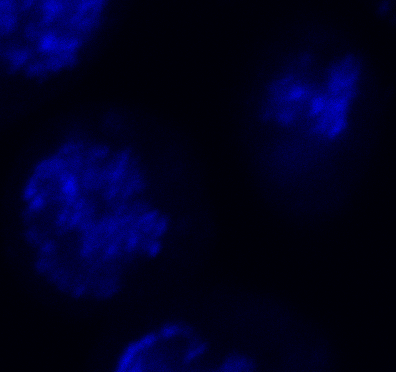

Supplement: Supplementary file 9 — Source data Fig. 6 [file 44319_2026_791_MOESM9_ESM.zip › Figure 6/B/lps-rox1 shrna-DAPI.tif]

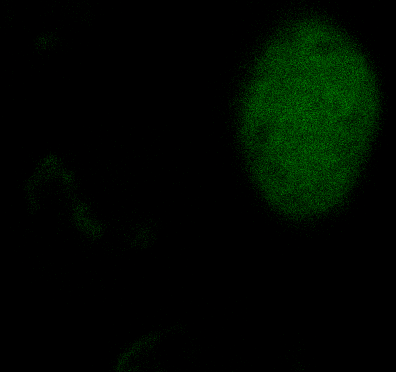

Supplement: Supplementary file 9 — Source data Fig. 6 [file 44319_2026_791_MOESM9_ESM.zip › Figure 6/B/lps-rox1 shrna-GFP.tif]

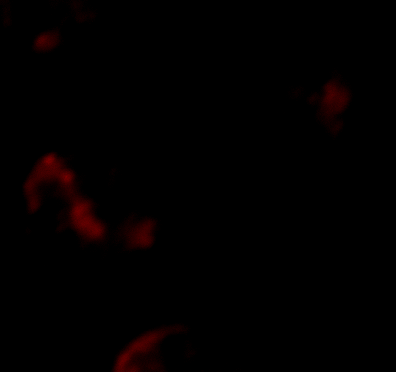

Supplement: Supplementary file 9 — Source data Fig. 6 [file 44319_2026_791_MOESM9_ESM.zip › Figure 6/B/lps-rox1 shrna-k9me3.tif]

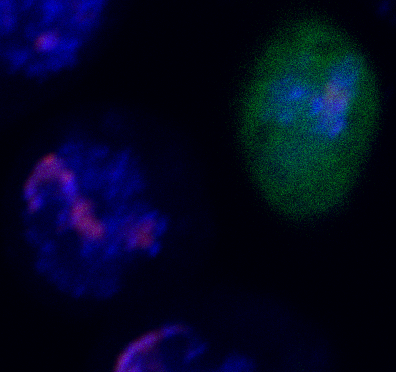

Supplement: Supplementary file 9 — Source data Fig. 6 [file 44319_2026_791_MOESM9_ESM.zip › Figure 6/B/lps-rox1 shrna-MERGE.tif]

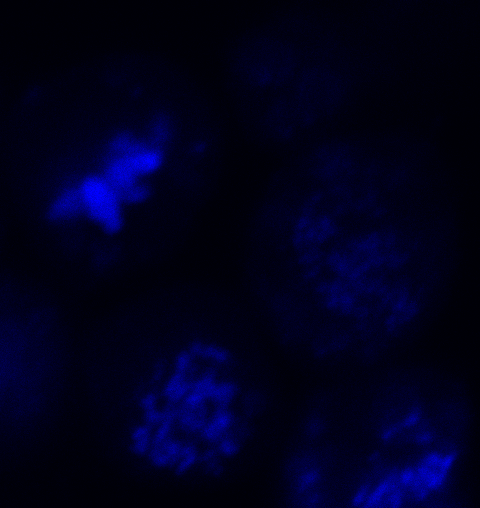

Supplement: Supplementary file 9 — Source data Fig. 6 [file 44319_2026_791_MOESM9_ESM.zip › Figure 6/B/lps-rox2 shrna-DAPI.tif]

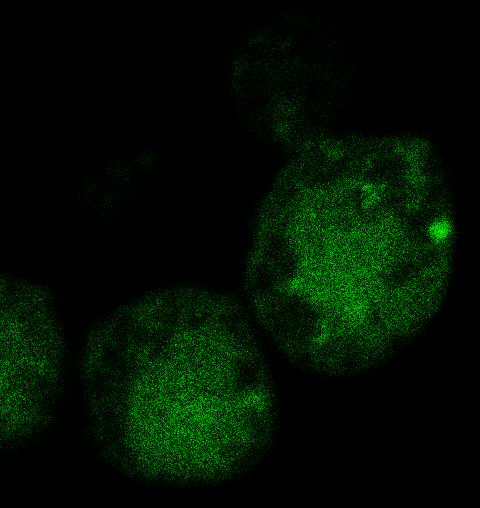

Supplement: Supplementary file 9 — Source data Fig. 6 [file 44319_2026_791_MOESM9_ESM.zip › Figure 6/B/lps-rox2 shrna-GFP.tif]

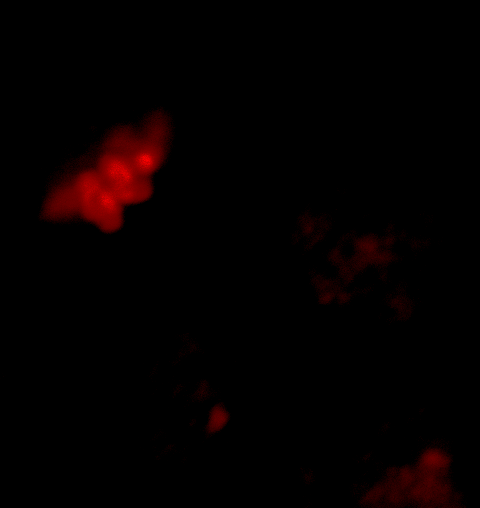

Supplement: Supplementary file 9 — Source data Fig. 6 [file 44319_2026_791_MOESM9_ESM.zip › Figure 6/B/lps-rox2 shrna-k9me3.tif]

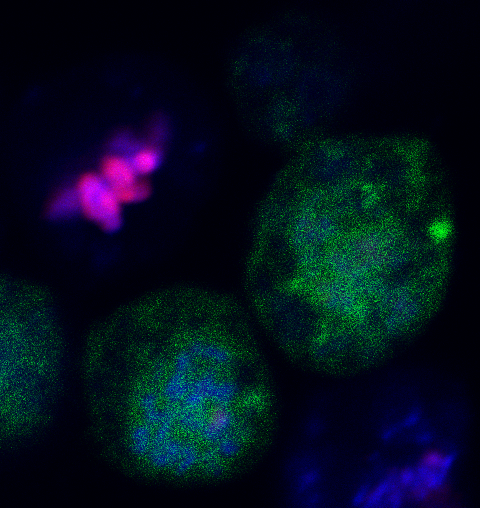

Supplement: Supplementary file 9 — Source data Fig. 6 [file 44319_2026_791_MOESM9_ESM.zip › Figure 6/B/lps-rox2 shrna-MERGE.tif]

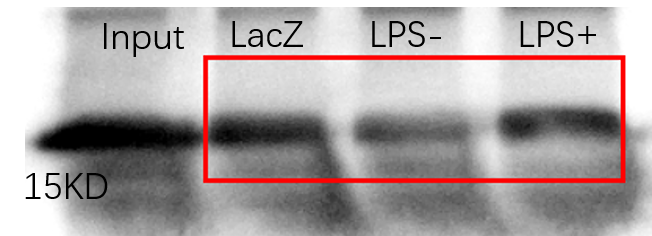

Supplement: Supplementary file 9 — Source data Fig. 6 [file 44319_2026_791_MOESM9_ESM.zip › Figure 6/F/Input-H3.png]

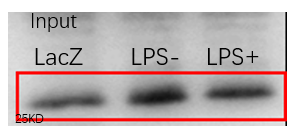

Supplement: Supplementary file 9 — Source data Fig. 6 [file 44319_2026_791_MOESM9_ESM.zip › Figure 6/F/Input-HP1a.png]

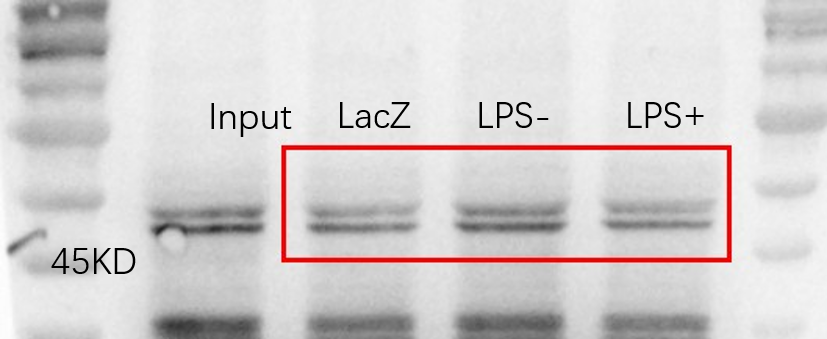

Supplement: Supplementary file 9 — Source data Fig. 6 [file 44319_2026_791_MOESM9_ESM.zip › Figure 6/F/Input-su(var)3-9.tif]

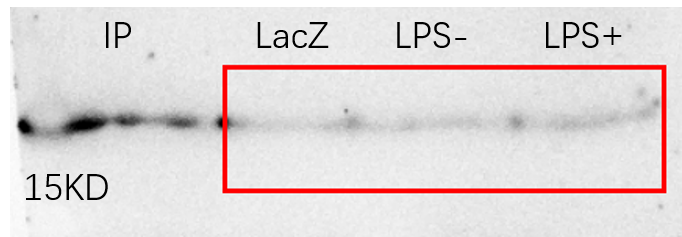

Supplement: Supplementary file 9 — Source data Fig. 6 [file 44319_2026_791_MOESM9_ESM.zip › Figure 6/F/IP-H3.png]

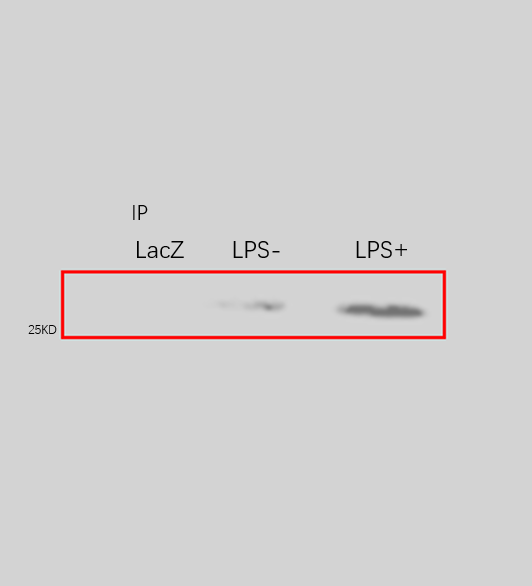

Supplement: Supplementary file 9 — Source data Fig. 6 [file 44319_2026_791_MOESM9_ESM.zip › Figure 6/F/IP-HP1a.png]

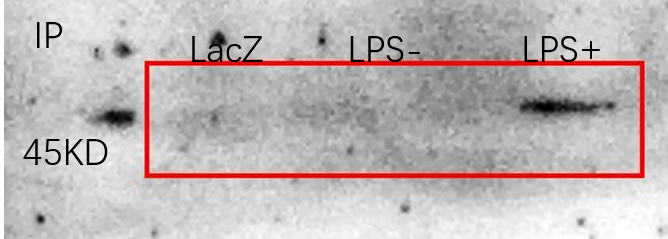

Supplement: Supplementary file 9 — Source data Fig. 6 [file 44319_2026_791_MOESM9_ESM.zip › Figure 6/F/IP-su(var)3-9.tif]

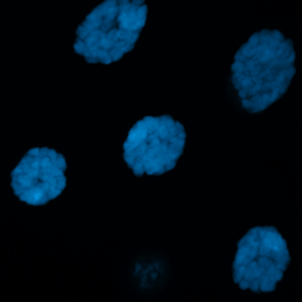

Supplement: Supplementary file 10 — Source data Fig. 7 [file 44319_2026_791_MOESM10_ESM.zip › Figure 7/A/control-DAPI.tif]

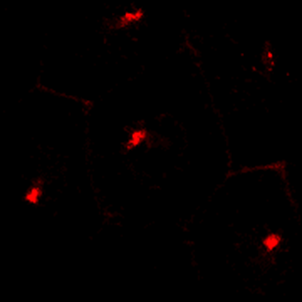

Supplement: Supplementary file 10 — Source data Fig. 7 [file 44319_2026_791_MOESM10_ESM.zip › Figure 7/A/control-HP1a.tif]

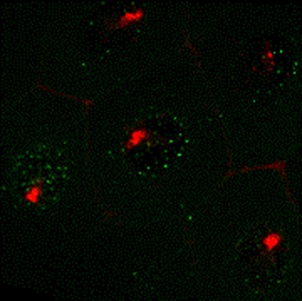

Supplement: Supplementary file 10 — Source data Fig. 7 [file 44319_2026_791_MOESM10_ESM.zip › Figure 7/A/control-roX and HP1a.tif]

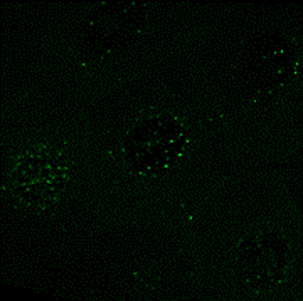

Supplement: Supplementary file 10 — Source data Fig. 7 [file 44319_2026_791_MOESM10_ESM.zip › Figure 7/A/control-roX2.tif]

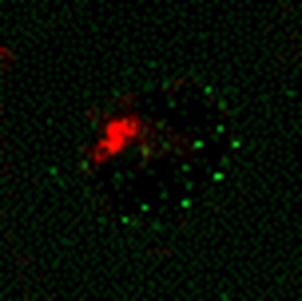

Supplement: Supplementary file 10 — Source data Fig. 7 [file 44319_2026_791_MOESM10_ESM.zip › Figure 7/A/control-zoom in image.tif]

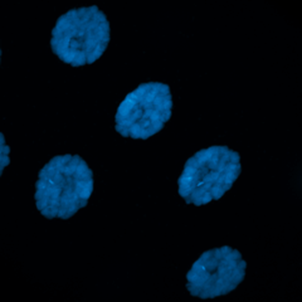

Supplement: Supplementary file 10 — Source data Fig. 7 [file 44319_2026_791_MOESM10_ESM.zip › Figure 7/A/roX2 OE-DAPI.tif]

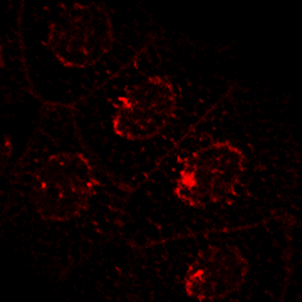

Supplement: Supplementary file 10 — Source data Fig. 7 [file 44319_2026_791_MOESM10_ESM.zip › Figure 7/A/roX2 OE-HP1a.tif]

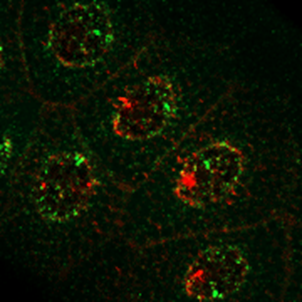

Supplement: Supplementary file 10 — Source data Fig. 7 [file 44319_2026_791_MOESM10_ESM.zip › Figure 7/A/roX2 OE-roX2 and HP1a.tif]

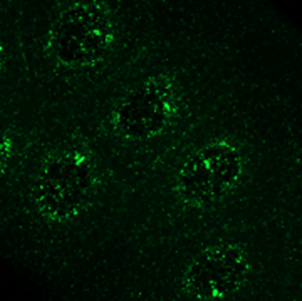

Supplement: Supplementary file 10 — Source data Fig. 7 [file 44319_2026_791_MOESM10_ESM.zip › Figure 7/A/roX2 OE-roX2.tif]

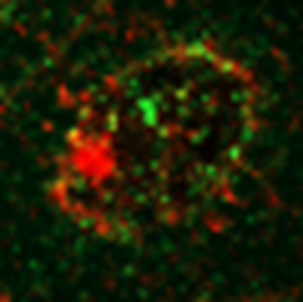

Supplement: Supplementary file 10 — Source data Fig. 7 [file 44319_2026_791_MOESM10_ESM.zip › Figure 7/A/roX2 OE-zoom in image.tif]

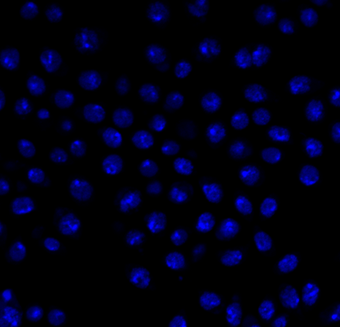

Supplement: Supplementary file 10 — Source data Fig. 7 [file 44319_2026_791_MOESM10_ESM.zip › Figure 7/C/DMSO-GFP RNAi-dapi.tif]

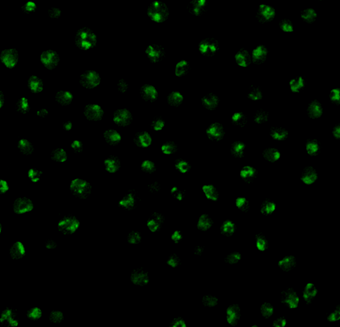

Supplement: Supplementary file 10 — Source data Fig. 7 [file 44319_2026_791_MOESM10_ESM.zip › Figure 7/C/DMSO-GFP RNAi-HP1a.tif]

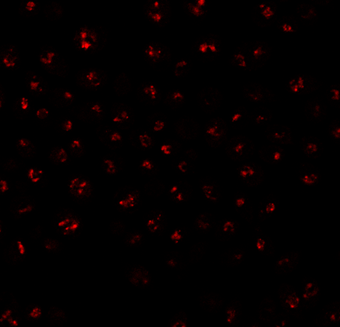

Supplement: Supplementary file 10 — Source data Fig. 7 [file 44319_2026_791_MOESM10_ESM.zip › Figure 7/C/DMSO-GFP RNAi-k9m3.tif]

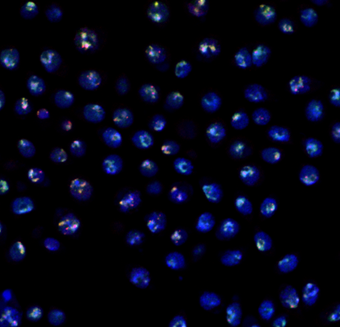

Supplement: Supplementary file 10 — Source data Fig. 7 [file 44319_2026_791_MOESM10_ESM.zip › Figure 7/C/DMSO-GFP RNAi-merge.tif]

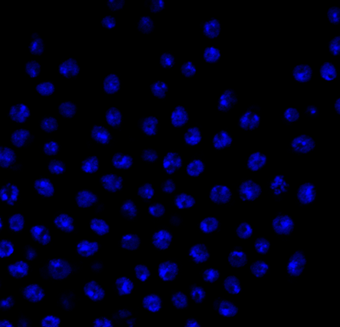

Supplement: Supplementary file 10 — Source data Fig. 7 [file 44319_2026_791_MOESM10_ESM.zip › Figure 7/C/Lps-GFP RNAi-dapi.tif]

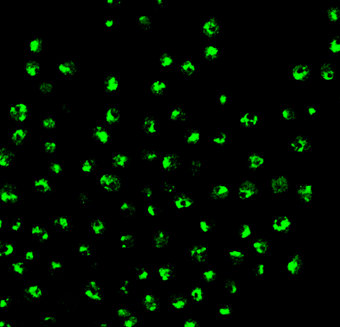

Supplement: Supplementary file 10 — Source data Fig. 7 [file 44319_2026_791_MOESM10_ESM.zip › Figure 7/C/Lps-GFP RNAi-HP1a.tif]

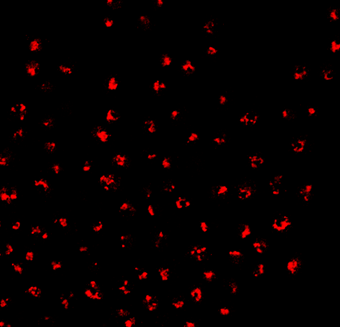

Supplement: Supplementary file 10 — Source data Fig. 7 [file 44319_2026_791_MOESM10_ESM.zip › Figure 7/C/Lps-GFP RNAi-k9m3.tif]

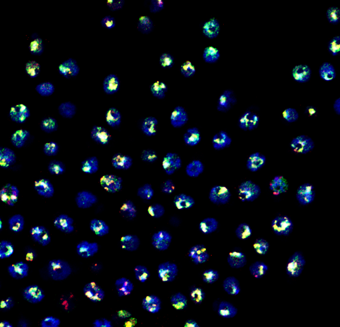

Supplement: Supplementary file 10 — Source data Fig. 7 [file 44319_2026_791_MOESM10_ESM.zip › Figure 7/C/Lps-GFP RNAi-merge.tif]

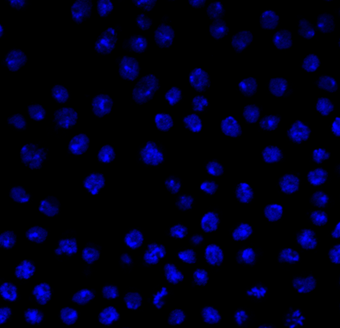

Supplement: Supplementary file 10 — Source data Fig. 7 [file 44319_2026_791_MOESM10_ESM.zip › Figure 7/C/Lps-roX2 RNAi+crl RNA-DAPI.tif]

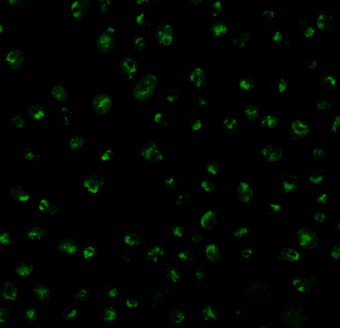

Supplement: Supplementary file 10 — Source data Fig. 7 [file 44319_2026_791_MOESM10_ESM.zip › Figure 7/C/Lps-roX2 RNAi+crl RNA-HP1a.tif]

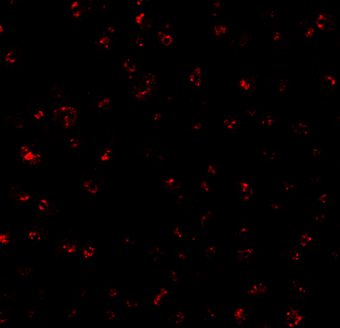

Supplement: Supplementary file 10 — Source data Fig. 7 [file 44319_2026_791_MOESM10_ESM.zip › Figure 7/C/Lps-roX2 RNAi+crl RNA-k9me3.tif]

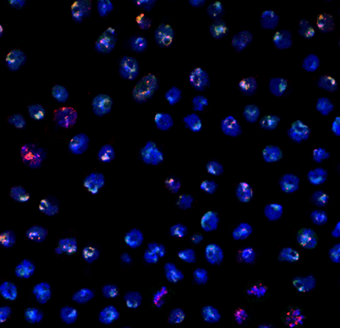

Supplement: Supplementary file 10 — Source data Fig. 7 [file 44319_2026_791_MOESM10_ESM.zip › Figure 7/C/Lps-roX2 RNAi+crl RNA-merge.tif]

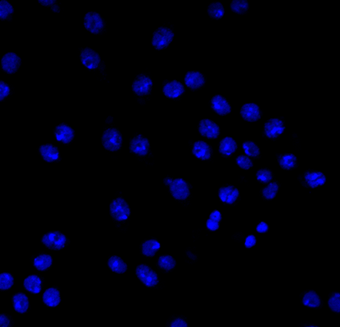

Supplement: Supplementary file 10 — Source data Fig. 7 [file 44319_2026_791_MOESM10_ESM.zip › Figure 7/C/Lps-roX2 RNAi+Xist-A RNA-dapi.tif]

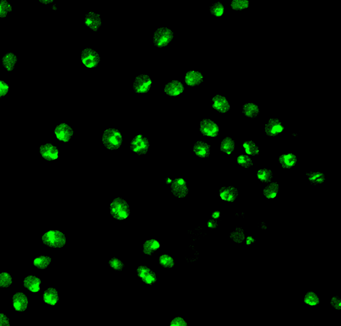

Supplement: Supplementary file 10 — Source data Fig. 7 [file 44319_2026_791_MOESM10_ESM.zip › Figure 7/C/Lps-roX2 RNAi+Xist-A RNA-HP1a.tif]

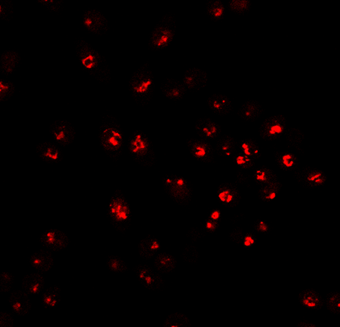

Supplement: Supplementary file 10 — Source data Fig. 7 [file 44319_2026_791_MOESM10_ESM.zip › Figure 7/C/Lps-roX2 RNAi+Xist-A RNA-k9me3.tif]

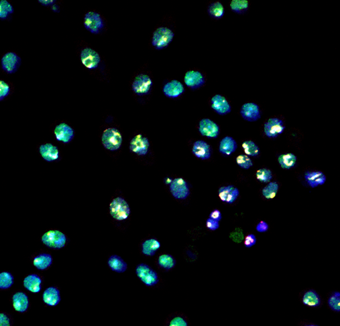

Supplement: Supplementary file 10 — Source data Fig. 7 [file 44319_2026_791_MOESM10_ESM.zip › Figure 7/C/Lps-roX2 RNAi+Xist-A RNA-merge.tif]

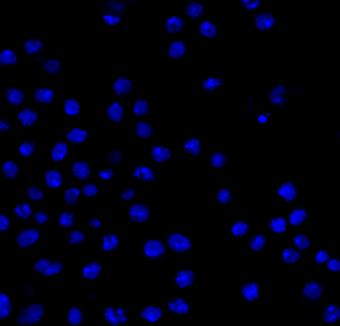

Supplement: Supplementary file 10 — Source data Fig. 7 [file 44319_2026_791_MOESM10_ESM.zip › Figure 7/C/Lps-roX2 RNAi+Xist-C RNA-DAPI.tif]

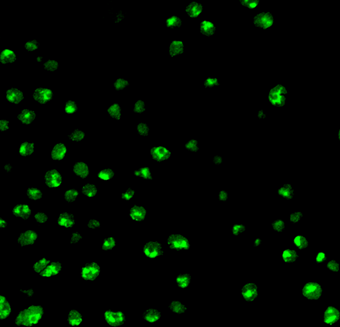

Supplement: Supplementary file 10 — Source data Fig. 7 [file 44319_2026_791_MOESM10_ESM.zip › Figure 7/C/Lps-roX2 RNAi+Xist-C RNA-HP1a.tif]

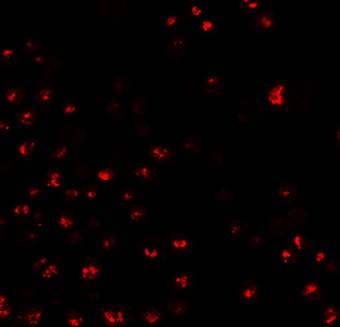

Supplement: Supplementary file 10 — Source data Fig. 7 [file 44319_2026_791_MOESM10_ESM.zip › Figure 7/C/Lps-roX2 RNAi+Xist-C RNA-k9me3.tif]

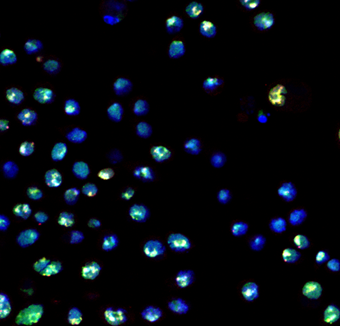

Supplement: Supplementary file 10 — Source data Fig. 7 [file 44319_2026_791_MOESM10_ESM.zip › Figure 7/C/Lps-roX2 RNAi+Xist-C RNA-merge.tif]

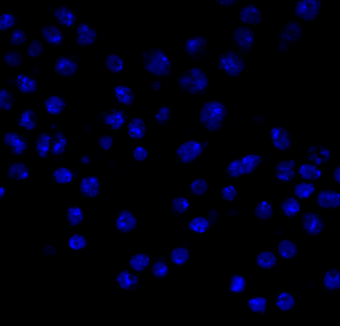

Supplement: Supplementary file 10 — Source data Fig. 7 [file 44319_2026_791_MOESM10_ESM.zip › Figure 7/C/Lps-roX2 RNAi+Xist-D RNA-DAPI.tif]

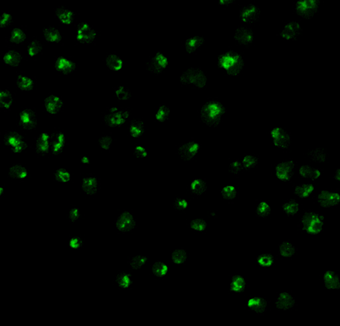

Supplement: Supplementary file 10 — Source data Fig. 7 [file 44319_2026_791_MOESM10_ESM.zip › Figure 7/C/Lps-roX2 RNAi+Xist-D RNA-HP1a.tif]

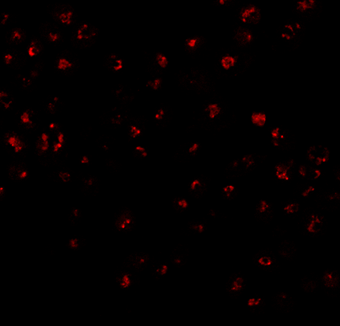

Supplement: Supplementary file 10 — Source data Fig. 7 [file 44319_2026_791_MOESM10_ESM.zip › Figure 7/C/Lps-roX2 RNAi+Xist-D RNA-k9me3.tif]

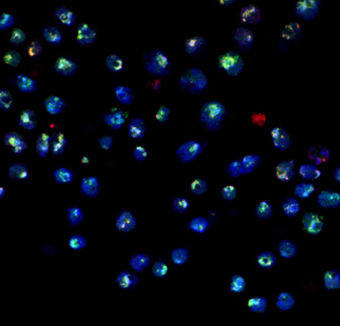

Supplement: Supplementary file 10 — Source data Fig. 7 [file 44319_2026_791_MOESM10_ESM.zip › Figure 7/C/Lps-roX2 RNAi+Xist-D RNA-merge.tif]

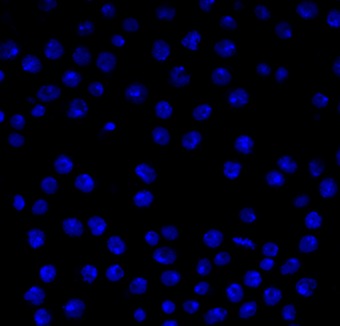

Supplement: Supplementary file 10 — Source data Fig. 7 [file 44319_2026_791_MOESM10_ESM.zip › Figure 7/C/Lps-roX2 RNAi-DAPI.tif]

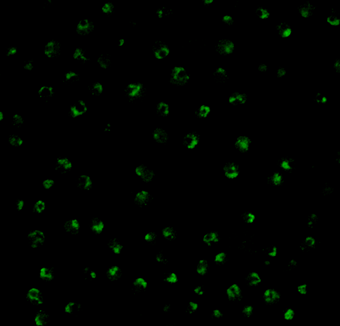

Supplement: Supplementary file 10 — Source data Fig. 7 [file 44319_2026_791_MOESM10_ESM.zip › Figure 7/C/Lps-roX2 RNAi-HP1a.tif]

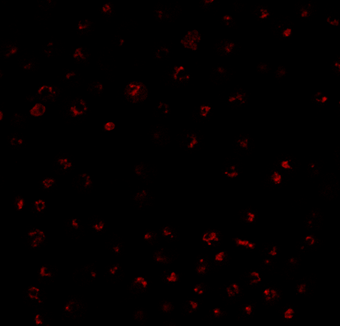

Supplement: Supplementary file 10 — Source data Fig. 7 [file 44319_2026_791_MOESM10_ESM.zip › Figure 7/C/Lps-roX2 RNAi-k9me3.tif]
