# Supplementary figures and images for: roX1 and roX2 lncRNAs promote heterochromatinization in intestinal stem cells and impair longevity (part 3 of 3)
Source: EMBO Rep. 2026 May 9;27(12):3394–423. doi: 10.1038/s44319-026-00791-8 (PMC13303914; doi:10.1038/s44319-026-00791-8)

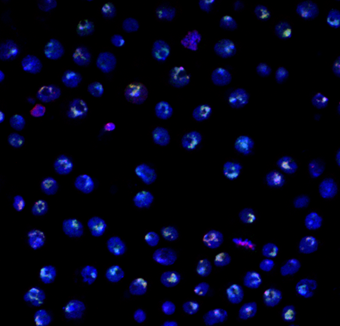

Supplement: Supplementary file 10 — Source data Fig. 7 [file 44319_2026_791_MOESM10_ESM.zip › Figure 7/C/Lps-roX2 RNAi-merge.tif]

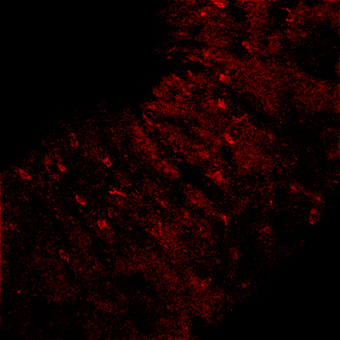

Supplement: Supplementary file 10 — Source data Fig. 7 [file 44319_2026_791_MOESM10_ESM.zip › Figure 7/F/Control-Delta.tif]

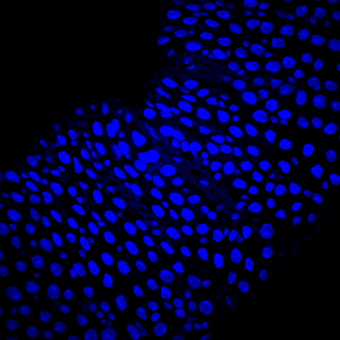

Supplement: Supplementary file 10 — Source data Fig. 7 [file 44319_2026_791_MOESM10_ESM.zip › Figure 7/F/Control-ISC-dapi.tif]

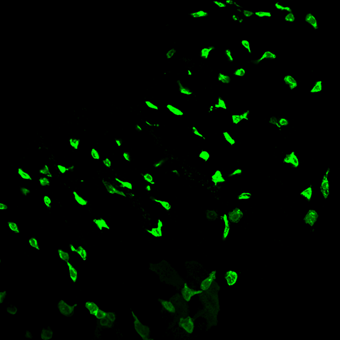

Supplement: Supplementary file 10 — Source data Fig. 7 [file 44319_2026_791_MOESM10_ESM.zip › Figure 7/F/Control-ISC-EB.tif]

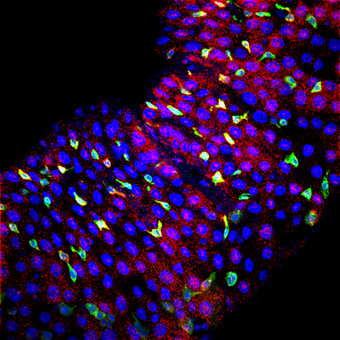

Supplement: Supplementary file 10 — Source data Fig. 7 [file 44319_2026_791_MOESM10_ESM.zip › Figure 7/F/Control-ISC-merge.tif]

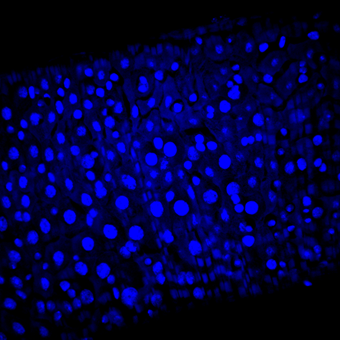

Supplement: Supplementary file 10 — Source data Fig. 7 [file 44319_2026_791_MOESM10_ESM.zip › Figure 7/F/roX2 OE-DAPI.tif]

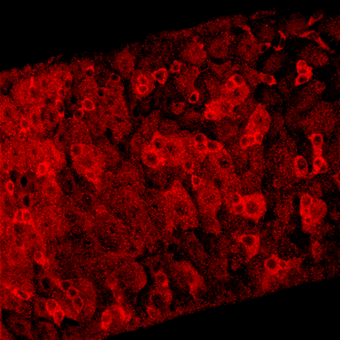

Supplement: Supplementary file 10 — Source data Fig. 7 [file 44319_2026_791_MOESM10_ESM.zip › Figure 7/F/roX2 OE-Delta.tif]

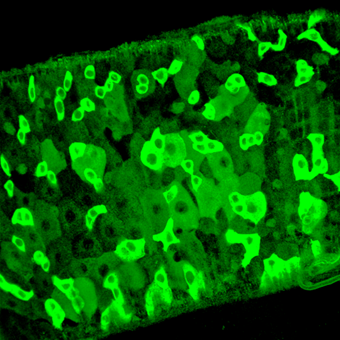

Supplement: Supplementary file 10 — Source data Fig. 7 [file 44319_2026_791_MOESM10_ESM.zip › Figure 7/F/roX2 OE-ISC-EB.tif]

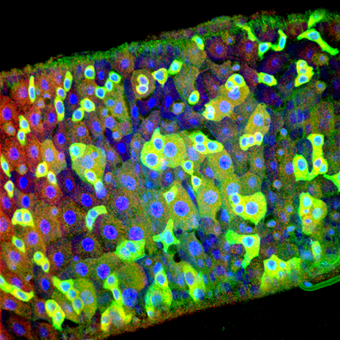

Supplement: Supplementary file 10 — Source data Fig. 7 [file 44319_2026_791_MOESM10_ESM.zip › Figure 7/F/roX2 OE-MERGE.tif]
