# Supplementary material for: roX1 and roX2 lncRNAs promote heterochromatinization in intestinal stem cells and impair longevity
Source: EMBO Rep. 2026 May 9;27(12):3394–423. doi: 10.1038/s44319-026-00791-8 (PMC13303914; doi:10.1038/s44319-026-00791-8)
Supplement: Supplementary file 11 — Expanded View Figures [file 44319_2026_791_MOESM11_ESM.pdf]

Expanded View Figures

Figure EV1. Pathogen stress promotes ISC hyperplasia with a reduction of euchromatinization.

(**A**) IF showing the ISCs/EBs (GFP, green) and ISCs (indicated by Delta antibody, red) in control and infected (fed with PA14) *Drosophila*. (**B**) Statistical results of the ISCs in (**A**). Data are from three independent biological replicates. The number of fields of view analyzed is indicated in the figure. (**C**) Statistical results of ISCs/EBs in (**A**). Data are from three independent biological replicates. The number of fields of view analyzed is indicated in the figure. (**D**) Gating strategies for identifying esg$^+$ cells (ISCs/EBs) and Delta$^+$ ISCs from the midguts of *esg-Gal4, UAS-GFP* or *Dl-Gal4* driven *UAS-GFP* female flies under aging and infection conditions." (**E**) Quantification of the percentage of esg$^+$ cells (ISCs/EBs) shows a significant increase in both aged and PA14-infected conditions compared to controls. $N = 3$. (**F**) The percentage of Delta$^+$ cells (ISCs) was quantified under infected conditions. $N = 3$. (**G**) PH3 staining of *esg-GAL4* female flies under PA14-infected and uninfected conditions. Arrows indicate PH3$^+$Delta$^+$ cells. (**H**) The number of PH3$^+$Delta$^+$ cells per midgut was quantified. ($N = 3$, the number of midguts is labeled on the graph). (**I**) Antibody staining shows the intensity of H3K4me3 (red) in ISCs/EBs (green, dotted circle) of control and PA14-infected *Drosophila*. Upper panel, midgut from uninfected *Drosophila* as a control (genotype: *esg-Gal4, UAS-GFP*); lower panel, midgut from PA14-infected *Drosophila*, same genotype as a control. (**J**) The bar graph shows quantification of H3K4me3 intensity from the control and infected gut. $N = 3$. The number of cells counted is labeled at the top of the bar graph, and the *P* value is included. (**K**) IF shows the intensity of H3K27ac (red) in ISCs/EBs (green, dotted circle) of control and PA14-infected *Drosophila*. Upper panel, midgut from uninfected *Drosophila* as a control (genotype: *esg-Gal4, UAS-GFP*); lower panel, midgut from PA14-infected *Drosophila*, same genotype as a control. (**L**)The bar graph indicates quantification of H3K27ac intensity from the control and infected gut. $N = 3$. The number of cells is indicated on the graph., and the *P* value is included. The center values are the averages, and the error bars indicate the s.e.m. *P* values were obtained by two-tailed unpaired Student's *t* test. n.s., not significant, $P \geq 0.05$, $^*P < 0.05$, $^{**}P < 0.01$, $^{***}P < 0.0001$. All images were captured from adult female posterior midguts. Scale bar, 10 μm.

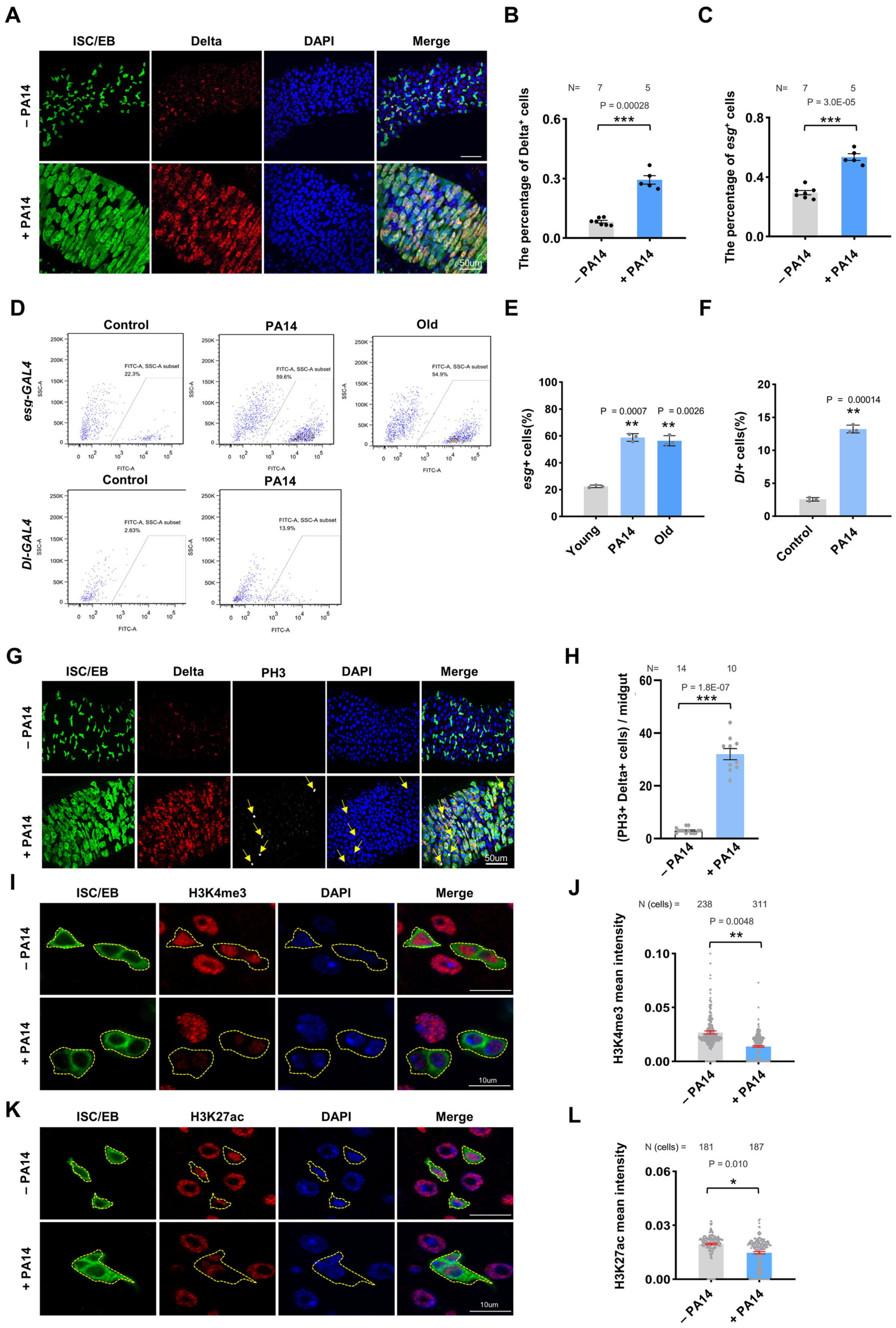
A
ISC/EB
Delta
DAPI
Merge
– PA14
+ PA14
50um
B
N= 7 5
P = 0.00028
***
The percentage of Delta+ cells
– PA14
+ PA14
C
N= 7 5
P = 3.0E-05
***
The percentage of esg+ cells
D
Control
PA14
Old
esg-GAL4
Dl-GAL4
FITC-A, SSC-A subset
22.3%
59.6%
54.9%
2.83%
13.9%
SSC-A
FITC-A
E
P = 0.0007
P = 0.0026
esg+ cells(%)
Young
PA14
Old
F
P = 0.00014
Dl+ cells(%)
Control
PA14
G
ISC/EB
Delta
PH3
DAPI
Merge
50um
H
N= 14 10
P = 1.8E-07
(PH3+ Delta+ cells) / midgut
I
ISC/EB
H3K4me3
DAPI
Merge
10um
J
N (cells) = 238 311
P = 0.0048
H3K4me3 mean intensity
K
ISC/EB
H3K27ac
DAPI
Merge
10um
L
N (cells) = 181 187
P = 0.010
H3K27ac mean intensity

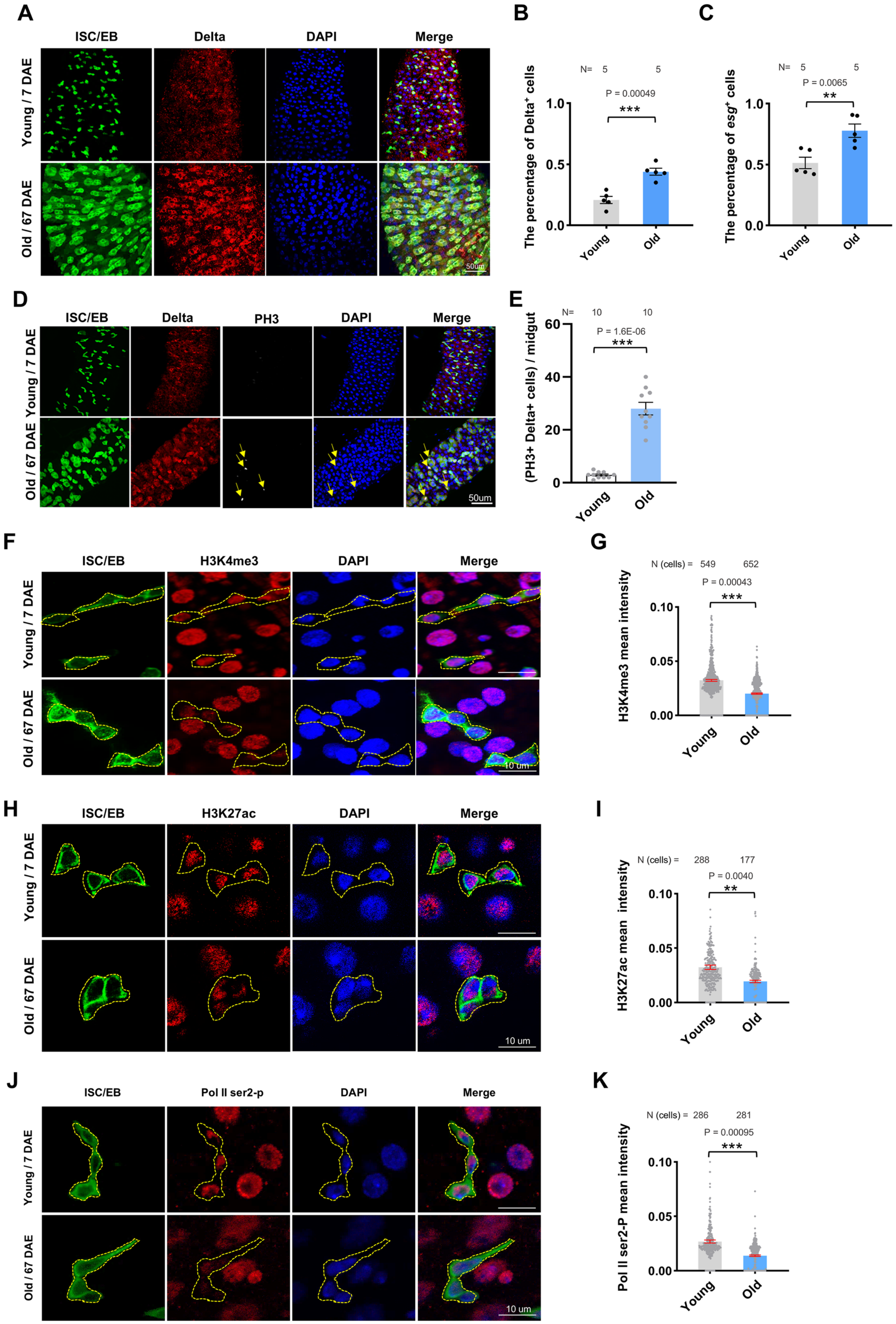
A
ISC/EB
Delta
DAPI
Merge
Young / 7 DAE
Old / 67 DAE
50um
B
N= 5 5
P = 0.00049
***
The percentage of Delta+ cells
1.0
0.5
0.0
Young
Old
C
N= 5 5
P = 0.0065
**
The percentage of esg+ cells
1.0
0.5
0.0
Young
Old
D
ISC/EB
Delta
PH3
DAPI
Merge
Young / 7 DAE
Old / 67 DAE
50um
E
N= 10 10
P = 1.6E-06
***
(PH3+ Delta+ cells) / midgut
60
40
20
0
Young
Old
F
ISC/EB
H3K4me3
DAPI
Merge
Young / 7 DAE
Old / 67 DAE
10 um
G
N (cells) = 549 652
P = 0.00043
***
H3K4me3 mean intensity
0.10
0.05
0.00
Young
Old
H
ISC/EB
H3K27ac
DAPI
Merge
Young / 7 DAE
Old / 67 DAE
10 um
I
N (cells) = 288 177
P = 0.0040
**
H3K27ac mean intensity
0.10
0.05
0.00
Young
Old
J
ISC/EB
Pol II ser2-p
DAPI
Merge
Young / 7 DAE
Old / 67 DAE
10 um
K
N (cells) = 286 281
P = 0.00095
***
Pol II ser2-P mean intensity
0.10
0.05
0.00
Young
Old

Figure EV2. ISC hyperplasia with a decrease of euchromatinization in aging.

(**A**) IF shows the ISCs/EBs (GFP, green) and ISCs alone (Delta antibody recognizing ISC, red) in Drosophila (*esg-Gal4*, *UAS-GFP*) at 7 DAE and 67 DAE. (**B**) Statistical analysis of the ISCs in (**A**). Data are from three independent biological replicates. The number of fields of view analyzed is indicated in the figure. (**C**) Statistical analysis of ISCs/EBs in (**A**). Data are from three independent biological replicates. The number of fields of view analyzed is indicated in the figure. (**D**) PH3 staining of *esg-GAL4* female flies from young and aged groups. Arrows indicate $PH3^{+}Delta^{+}$ cells. (**E**) The number of $PH3^{+}Delta^{+}$ cells per midgut was quantified. $N = 3$. The number of counted midguts is marked at the top. (**F**) Antibody staining shows the intensity of H3K4me3 (red) in ISCs/EBs (green, dotted circle) in young and old posterior midguts. Upper panel, midgut from young Drosophila as a control (genotype: *esg-Gal4, UAS-GFP*), 7 DAE; lower panel, midgut from aged Drosophila, same genotype as young, 67 DAE. (**G**) The bar graph shows the quantification of H3K4me3 intensity from young and old midguts. $N = 3$. The number of cells is indicated on the graph, and the *P* value is included. (**H**) Antibody staining showing the intensity of H3K27ac (red) in ISCs/EBs (green, dotted circle) from young and old midguts, the same genotype and the same condition as used in (**F**). (**I**) The bar graph indicates the quantification of H3K27ac intensity from young and old midguts. $N = 3$. The number of cells is indicated on the graph, and *P* value is included. (**J**) IF showing the intensity of Pol II Ser2-p (red) in ISCs/EBs (green, dotted circle) from young and old midguts with the same genotype and the same conditions as used in (**F**). (**K**) The bar graph shows the quantification of Pol II Ser2-p intensity in young and old midguts. $N = 3$. The number of cells is indicated on the graph, and *P* values are included. The center values are the averages, and the error bars indicate the s.e.m. *P* values were obtained by two-tailed unpaired Student's *t* test. n.s., not significant, $P \geq 0.05$, $^{*}P < 0.05$, $^{**}P < 0.01$, $^{***}P < 0.0001$. All images were captured from adult female posterior midguts. Scale bar, 10 μm.

 © The Author(s)

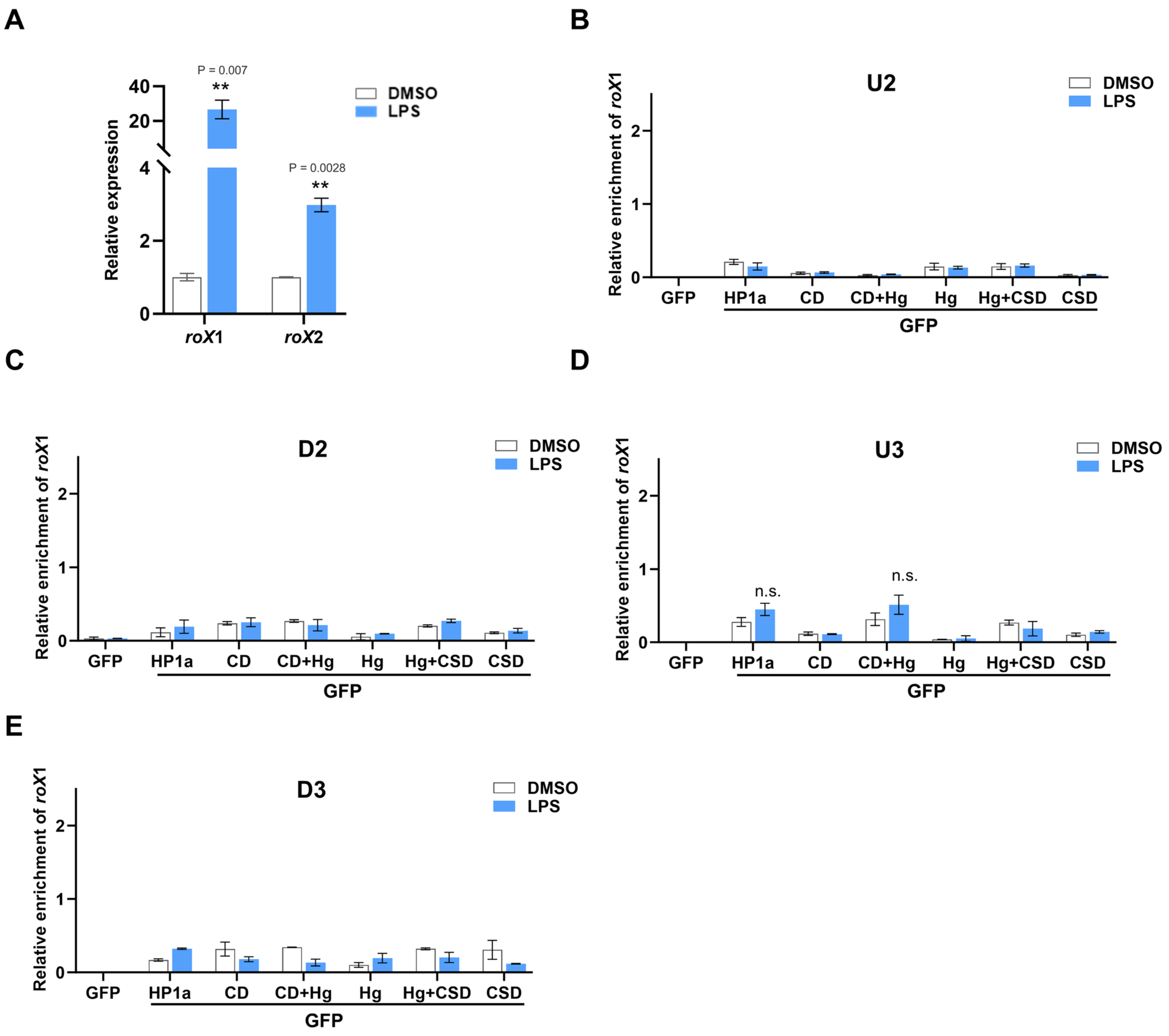

Figure EV3. Other regions in the *roX*1 did not participate in the interaction with the heterochromatin protein.

(**A**) LPS treatment promotes *roX* RNAs expression compared to the control. $N = 3$. (**B–E**) RIP-crosslink coupled with Q-PCR experiments show no interaction between different domains of HP1a and U2, D2, U3, and D3 of *roX*1. $N = 3$. The center values are the averages, and the error bars indicate the s.e.m. *P* values were obtained by two-tailed unpaired Student's *t* test. n.s., not significant, $P \geq 0.05$, $^{*}P < 0.05$, $^{**}P < 0.01$, $^{***}P < 0.0001$.

© The Author(s)

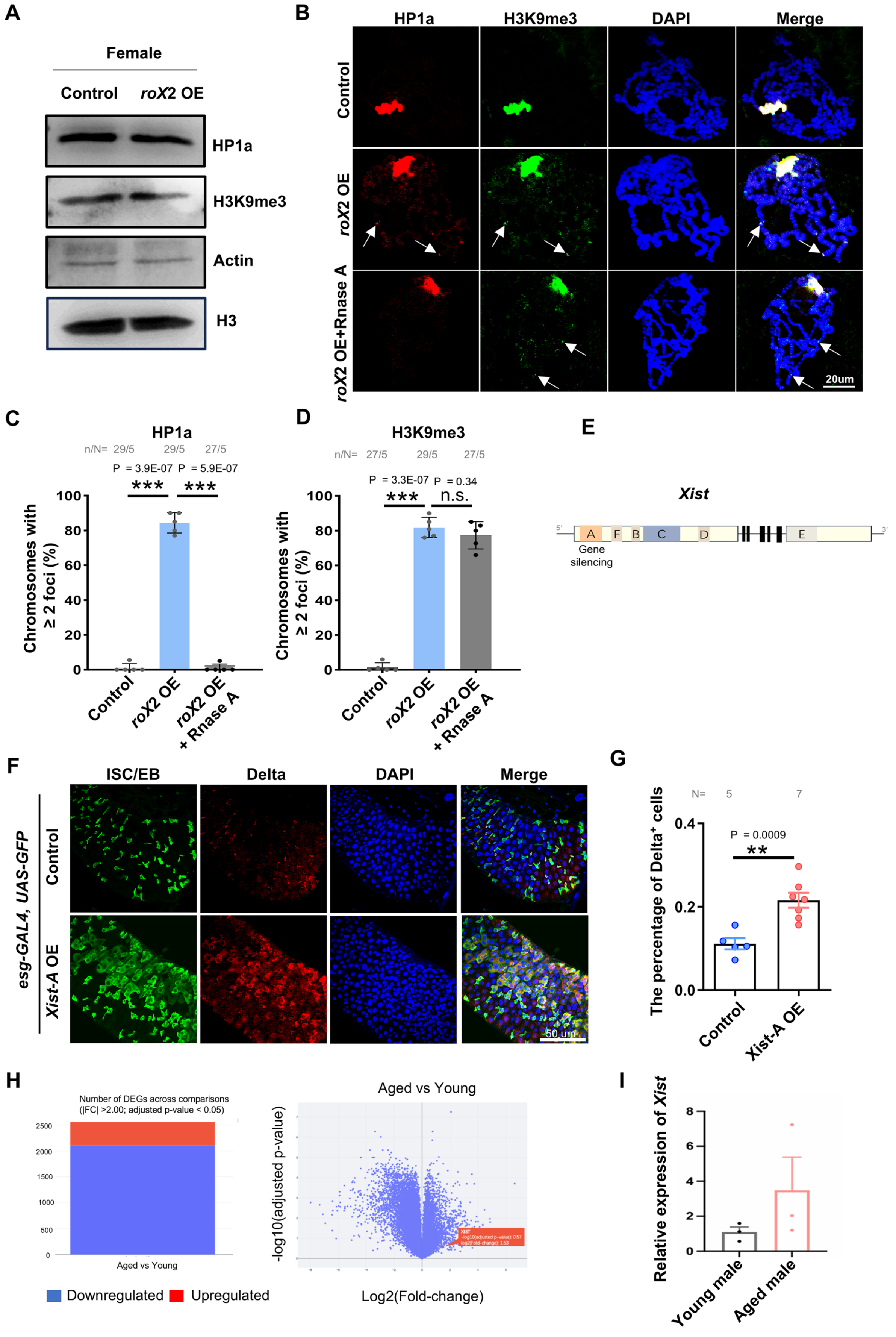
A
Female
Control roX2 OE
HP1a
H3K9me3
Actin
H3
B
HP1a H3K9me3 DAPI Merge
Control
roX2 OE
roX2 OE+Rnase A
20um
C
HP1a
n/N= 29/5 29/5 27/5
P = 3.9E-07 P = 5.9E-07
***
Chromosomes with ≥ 2 foci (%)
Control
roX2 OE
roX2 OE + Rnase A
D
H3K9me3
n/N= 27/5 29/5 27/5
P = 3.3E-07 P = 0.34
n.s.
E
Xist
A F B C D E
Gene silencing
F
ISC/EB Delta DAPI Merge
esg-GAL4, UAS-GFP
Control
Xist-A OE
50 um
G
N= 5 7
P = 0.0009
**
The percentage of Delta+ cells
Control
Xist-A OE
H
Number of DEGs across comparisons
(|FC| >2.00; adjusted p-value < 0.05)
Aged vs Young
Downregulated Upregulated
-log10(adjusted p-value)
Log2(Fold-change)
I
Relative expression of Xist
Young male
Aged male

Figure EV4. Ectopic expression of *roX2* promotes the heterochromatinization.

(**A**) Western blot shows no change in HP1a and H3K9me3 after *roX*2 overexpression. (**B**) The polytene chromosome staining shows the spreading of HP1a and H3K9me3 after *roX*2 overexpression, and the increase of HP1a is sensitive to RNaseA treatment. The arrow indicates the site of HP1a or H3K9me3 spreading. (**C**, **D**) Quantification of the percentage of chromosomes with HP1a (**C**) or H3K9me3 (**D**) spreading in (**B**). The observation of two or more HP1a or H3K9me3 signal foci on salivary gland chromosomes is defined as spreading. $N = 5$, $n \geq 27$ Chromosomes. (**E**) The different regions of *Xist* RNA in humans. (**F**) IF showing the ISCs/EBs (GFP, green) and ISCs (indicated by Delta antibody, red) in control *Drosophila* (upper panel) and *Xist-A* overexpression *Drosophila* driven by *esg-GAL4 UAS-GFP* (lower panel). (**G**) The percentage of ISCs in (**F**) was quantified. Data are from three independent biological replicates. The number of microscopic fields analyzed is indicated in the figure. (**H**) The column plot for the differentially expressed genes (left) and volcano plot for the details (right) from the previous RNA-seq data of human endothelial cells in young and aged people, the red dot indicates the *Xist*. (**I**) The relative expression level of *Xist* RNA in men ($N = 3$). *P* value was obtained by a two-tailed unpaired Student's *t* test. n.s., not significant, $P \geq 0.05$, $^*P < 0.05$, $^{**}P < 0.01$, $^{***}P < 0.0001$. Scale bar, 20 μm or 50 μm.

© The Author(s)

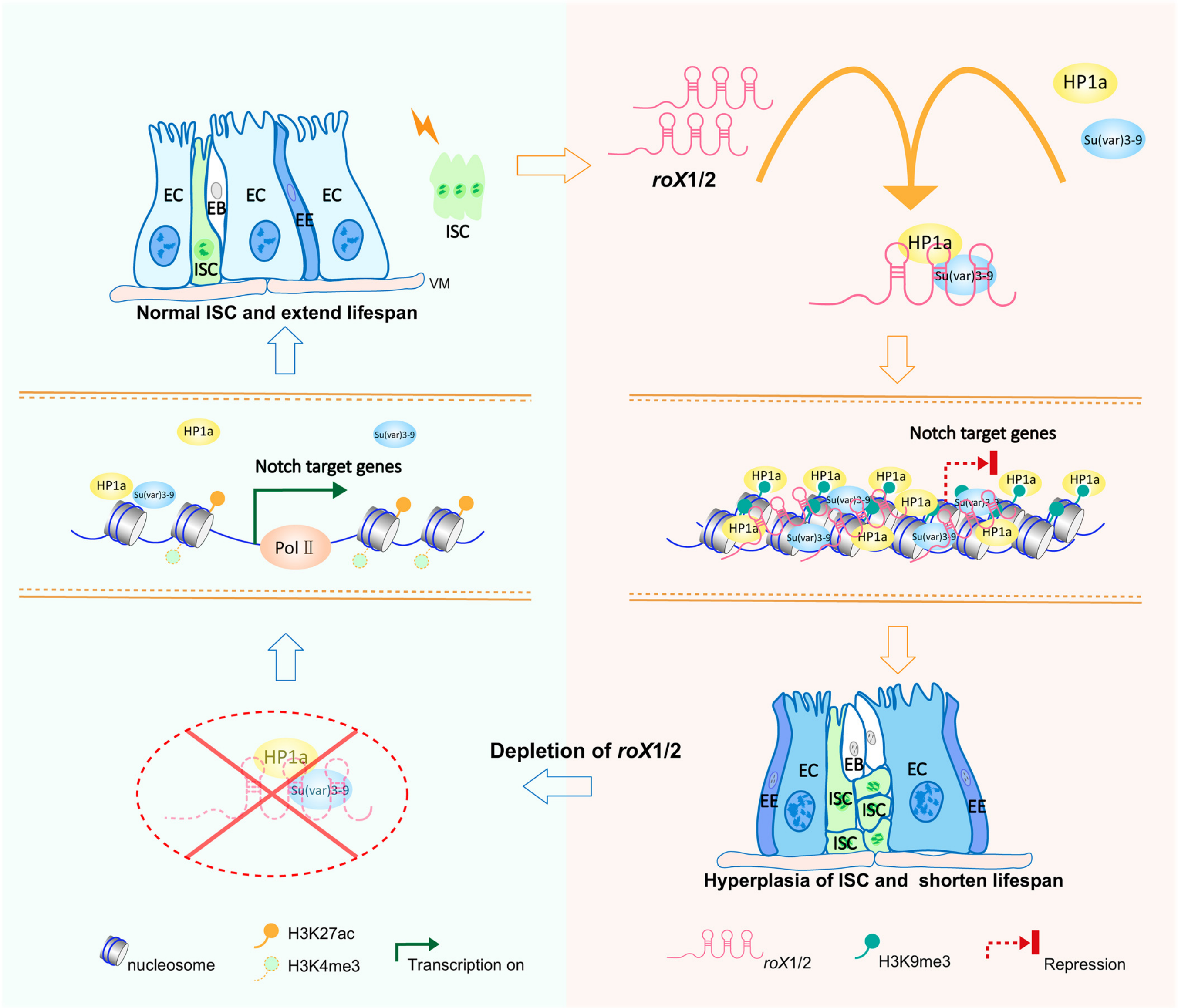

Figure EV5. Schematic for the present study.

Under stressed conditions, such as pathogen treatment or aging, the increase of *roX* RNAs associated with heterochromatin proteins then guides heterochromatin spreading to target essential genes of ISC homeostasis. If it reduces the levels of *roX* RNAs, then it prevents heterochromatinization, releases the inhibition on genes of ISC homeostasis, and eventually extends the animal lifespan.

 © The Author(s)
