# Supplementary material for: roX1 and roX2 lncRNAs promote heterochromatinization in intestinal stem cells and impair longevity
Source: EMBO Rep. 2026 May 9;27(12):3394–423. doi: 10.1038/s44319-026-00791-8 (PMC13303914; doi:10.1038/s44319-026-00791-8)
Supplement: Supplementary file 3 — Peer Review File [file 44319_2026_791_MOESM3_ESM.pdf]

EMBO reports

roX1 and roX2 lncRNAs promote heterochromatinization in intestinal stem cells and impair longevity

Kai-Le Li, Chen Wang, Yong-Hao Li, Bo Peng, Li Zheng, Qinyun Che, Xinyi Lu, Donglin Li, Yutong Li, Huimin Wei, Jian-Feng Chang, Wen-Fei Wang, Mengmeng Liu, Xiangnan Li, Fangfang JIn, Kai Liu, Yabin Li, Li-Wei Liu, Mingyang Li, Jian-Quan Ni, Ting Xie, and Fang-Lin Sun

Corresponding author(s): Jian-Quan Ni (nijq@mail.tsinghua.edu.cn) , Fang-Lin Sun (sfl@tongji.edu.cn), Ting Xie (tgx@ust.hk)

Review Timeline:

Submission Date:	4th Jul 25
Editorial Decision:	19th Aug 25
Revision Received:	3rd Dec 25
Editorial Decision:	9th Mar 26
Revision Received:	24th Mar 26
Accepted:	20th Apr 26

Editor: Esther Schnapp

Transaction Report:

(Note: With the exception of the correction of typographical or spelling errors that could be a source of ambiguity, letters and reports are not edited. Depending on transfer agreements, referee reports obtained elsewhere may or may not be included in this compilation. Referee reports are anonymous unless the Referee chooses to sign their reports.)

1st Editorial Decision **19th Aug 2025**

Dear Dr. Ni,

Thank you for the transfer of your manuscript to EMBO reports. We have now received the full set of referee reports that is pasted below.

As you will see, the referees acknowledge that the findings are potentially interesting. However, together they raise a number of concerns that all would need to be addressed for the publication of your study here. I understand that this means extensive revisions but apart from point 9 by referee 3 and point 3 by referee 2 I think all points are important and should be addressed. Please let me know in case you disagree and we can discuss the exact revision requirements further, also in a video chat, if you like.

I would thus like to invite you to revise your manuscript with the understanding that the referee concerns must be fully addressed and their suggestions taken on board. Please address all referee concerns in a complete point-by-point response. Acceptance of the manuscript will depend on a positive outcome of a second round of review. It is EMBO reports policy to allow a single round of major revision only and acceptance or rejection of the manuscript will therefore depend on the completeness of your responses included in the next, final version of the manuscript.

We realize that it is difficult to revise to a specific deadline. In the interest of protecting the conceptual advance provided by the work, we recommend a revision within 3 months (19th Nov 2025). Please discuss the revision progress ahead of this time with the editor if you require more time to complete the revisions.

IMPORTANT NOTE: we perform an initial quality control of all revised manuscripts before re-review. Your manuscript will FAIL this control and the handling will be DELAYED if the following APPLIES:
1) A data availability section providing access to data deposited in public databases is missing. If you have not deposited any data, please add a sentence to the data availability section that explains that.
2) Your manuscript contains statistics and error bars based on n=2. Please use scatter blots in these cases. No statistics should be calculated if n=2.

When submitting your revised manuscript, please carefully review the instructions that follow below. Failure to include requested items will delay the evaluation of your revision.

1) a .docx formatted version of the manuscript text (including legends for main figures, EV figures and tables). Please make sure that the changes are highlighted to be clearly visible.

2) individual production quality figure files as .eps, .tif, .jpg (one file per figure). See https://wol-prod-cdn.literatumonline.com/pb-assets/embo-site/EMBOPress_Figure_Guidelines_061115-1561436025777.pdf for more info on how to prepare your figures.

3) We replaced Supplementary Information with Expanded View (EV) Figures and Tables that are collapsible/expandable online. A maximum of 5 EV Figures can be typeset. EV Figures should be cited as 'Figure EV1, Figure EV2" etc... in the text and their respective legends should be included in the main text after the legends of regular figures.

- For the figures that you do NOT wish to display as Expanded View figures, they should be bundled together with their legends in a single PDF file called *Appendix*, which should start with a short Table of Content. Appendix figures should be referred to in the main text as: "Appendix Figure S1, Appendix Figure S2" etc. See detailed instructions regarding expanded view here: <https://www.embopress.org/page/journal/14693178/authorguide#expandedview>

- Additional Tables/Datasets should be labeled and referred to as Table EV1, Dataset EV1, etc. Legends have to be provided in a separate tab in case of .xls files. Alternatively, the legend can be supplied as a separate text file (README) and zipped together with the Table/Dataset file.

4) a .docx formatted letter INCLUDING the reviewers' reports and your detailed point-by-point responses to their comments. As part of the EMBO Press transparent editorial process, the point-by-point response is part of the Review Process File (RPF), which will be published alongside your paper.

5) a complete author checklist, which you can download from our author guidelines <https://www.embopress.org/page/journal/14693178/authorguide>. Please insert information in the checklist that is also reflected in the manuscript. The completed author checklist will also be part of the RPF.

6) Please note that all corresponding authors are required to supply an ORCID ID for their name upon submission of a revised manuscript (<https://orcid.org/>). Please find instructions on how to link your ORCID ID to your account in our manuscript tracking system in our Author guidelines <https://www.embopress.org/page/journal/14693178/authorguide#authorshipguidelines>

7) Before submitting your revision, primary datasets produced in this study need to be deposited in an appropriate public database (see https://www.embopress.org/page/journal/14693178/authorguide#datadeposition). Please remember to provide a reviewer password if the datasets are not yet public. The accession numbers and database should be listed in a formal "Data Availability" section placed after Materials & Method (see also https://www.embopress.org/page/journal/14693178/authorguide#datadeposition). Please note that the Data Availability Section is restricted to new primary data that are part of this study. * Note - All links should resolve to a page where the data can be accessed. *
If your study has not produced novel datasets, please mention this fact in the Data Availability Section.

8) At EMBO Press we ask authors to provide source data for the main manuscript figures. You will receive a separate email with instructions for providing source data with your revised manuscript, including how to upload and organize the files.

9) Our journal also encourages inclusion of *data citations in the reference list* to directly cite datasets that were re-used and obtained from public databases. Data citations in the article text are distinct from normal bibliographical citations and should directly link to the database records from which the data can be accessed. In the main text, data citations are formatted as follows: "Data ref: Smith et al, 2001" or "Data ref: NCBI Sequence Read Archive PRJNA342805, 2017". In the Reference list, data citations must be labeled with "[DATASET]". A data reference must provide the database name, accession number/identifiers and a resolvable link to the landing page from which the data can be accessed at the end of the reference. Further instructions are available at https://www.embopress.org/page/journal/14693178/authorguide#referencesformat

10) Regarding data quantification (see Figure Legends: https://www.embopress.org/page/journal/14693178/authorguide#figureformat)

The following points must be specified in each figure legend:

- the name of the statistical test used to generate error bars and P values,

- the number (n) of independent experiments (please specify technical or biological replicates) underlying each data point,

- the nature of the bars and error bars (s.d., s.e.m.),

- If the data are obtained from n Program fragment delivered error ``Can't locate object method "less" via package "than" (perhaps you forgot to load "than"?) at //ejpvfs23/sites23b/embor_www/letters/embor_decision_revise_and_review.txt line 56.' 2, use scatter blots showing the individual data points.

Discussion of statistical methodology can be reported in the materials and methods section, but figure legends should contain a basic description of n, P and the test applied.

- Please also include scale bars in all microscopy images.

11) The journal requires a statement specifying whether or not authors have competing interests (defined as all potential or actual interests that could be perceived to influence the presentation or interpretation of an article). In case of competing interests, this must be specified in your disclosure statement. Further information: https://www.embopress.org/competing-interests

12) All Materials and Methods need to be described in the main text using our 'Structured Methods' format, which is required for all research articles. According to this format, the Methods section includes a separate Reagents and Tools Table file (listing key reagents, experimental models, software and relevant equipment and including their sources and relevant identifiers) and a Methods and Protocols section describing the methods using a step-by-step protocol format. The aim is to facilitate adoption of the methodologies across labs. More information on how to adhere to this format as well as a downloadable template (.docx) for the Reagents and Tools Table can be found in our author guidelines: https://www.embopress.org/page/journal/14693178/authorguide#structuredmethods.

An example of a Method paper with Structured Methods can be found here: https://www.embopress.org/doi/full/10.1038/s44320-024-00037-6#sec-4

We would also welcome the submission of cover suggestions, or motifs to be used by our Graphics Illustrator in designing a cover.

As part of the EMBO publication's Transparent Editorial Process, EMBO reports publishes online a Review Process File (RPF) to accompany accepted manuscripts. This File will be published in conjunction with your paper and will include the referee reports, your point-by-point response and all pertinent correspondence relating to the manuscript.

You are able to opt out of this by letting the editorial office know (emboreports@embo.org). If you do opt out, the Review Process File link will point to the following statement: "No Review Process File is available with this article, as the authors have chosen not to make the review process public in this case."

I look forward to seeing a revised form of your manuscript when it is ready.

Best regards,
Esther

Esther Schnapp, PhD
Senior Editor
EMBO reports

Referee #1:

Overall summary and major criticisms
This very intriguing study finds a role for the noncoding Rox RNAs in heterochromatization in aging and stressed female midguts. The authors present some convincing data but are prone to over interpretation. Several findings that counter well-established features of heterochromatin or dosage compensation are briefly presented but not further commented on. While the authors may indeed have discovered remarkable new properties of these systems, they owe the reader a more in depth treatment. The authors might consider introducing a graphical model of the relationship between RNA, heterochromatin, stress, hyperplasia and ageing in the main body of the paper. Something akin to S8 but simpler.
The primary claim to novelty is a non-canonical role for Rox RNA in female flies, but the authors fail to address previous studies revealing repressive, autosomal roles of these RNAs. Early this year a study reporting association of Rox RNA with Polycomb complexes, and a role for Rox RNA in repression of developmental genes, was published (doi.org/10.1038/s41467-024-55711-y). Intriguingly, the affected genes have female-biased expression. Rox mutations have also been found to suppress variegation of insertions in autosomal heterochromatin (10.1534/genetics.109.102087; 10.1371/journal.pone.0140259). In contrast with the present study, suppression of variegation and misregulation of heterochromatic genes was limited to males. It would be a definite contribution to the field if the authors put forth a model that incorporates previously reported aspects of autosomal gene regulation with their current findings!
The markers used for heterochromatin formation - HP1a and H3K9me3 have little resolution. As the authors know the genes affected, a chromatin accessibility assay like ATAC seq would provide confirmation of changes in chromatin.
Rox1 and Rox2 are redundant for dosage compensation, but many of the studies presented here knockdown just one. Could the authors speculate on why the single knockdowns achieve identical results? They also present the striking finding that knockdown of both Rox RNAs with a strong driver has no effect on males but extends female lifespan. As the endogenous levels of Rox are vastly different in the two sexes, measurement of total RNA after knockdown might be informative.
Referring to Xist as a Rox homolog suggests an evolutionary relationship that does not exist (line 42). Please refine this description to acknowledge similarities between molecules that lack a shared ancestor. Drosophila is very unlikely to provide proper molecular context for Xist behavior in cells. In stretching for parallels between Xist and Rox the authors do a disservice to their work.
Female life extension by knockdown of a single Rox gene (Figure 5) is very intriguing! If this is indeed true, the authors should be able to replicate this finding with Rox mutants.
Figure S7 is problematic on several levels. Panel B presents antibody staining of three preparations as justification for the claim that Rox overexpression expands heterochromatin, and this is reversed by RNase. There is no indication of scoring criteria or number of preparations examined. Given the variability between preparations done on the same genotype, this is flimsy evidence for the claim that RNase treatment reduces HP1 localization.
Minor criticisms
Explain what delta antibody recognizes and why it is used as a marker for stem cells.
Lines 154-156. Are all the midguts from females?
Lines 157-158. The authors state that overall gene expression decreases, but do not explain how reads are normalized. How do they know that some preparations simply had less mRNA?
Line 191. Explain why the esg driver is used.
Lines 213-214 appears to lack citations for Notch activation.
Line 300-302 states that Rox RNA, not H3K9me3, is responsible for HP1 recruitment. This is a novel suggestion that upends conventional wisdom about HP1 properties. See major criticism about the quality of data this statement is based on.
Line 323 refers to detection of Xist in blood. This is presumably a byproduct of cell death and might be a better marker of inflammation and apoptosis than of cellular levels of Xist.
Line 326 suggests a false relationship between Xist and Rox RNAs.
Line 363 "Conversely, the absence of such regulators, such as MSL or MOF, in the female . . . " needs to be more specific. The only MSL absent in females is MSL2, and even this is controversial.
Lines 368 -71 double down on functional equivalency between Xist and Rox, suggesting that Xist regions interacting with PcG can rescue Rox function. Do the authors believe that these regions interact with PcG or HP1 in flies?

Figure 2. Please label the scale bar in 2E. A more complete description of the comparisons presented in this figure would be appreciated. What do the red circles represent? For Figures 2(F) and 2(G) and their corresponding texts, it would be easier for the readers to appreciate the magnitude of significance if specific fold changes for the qPCR validation were mentioned.
In figure 4E the authors find that genes on the 4th chromosome increase expression following Rox2 KD. As this chromosome is famously heterochromatic, this is of interest but not commented on by the authors.

Referee #2:

In this manuscript, Li et al. investigate the role of long noncoding roX RNAs in regulating heterochromatin formation in intestinal stem cells (ISCs) and enteroblasts (EBs) in Drosophila. They report that elevated expression of roX RNAs in ISCs/EBs promotes heterochromatinization and represses gene expression by directly recruiting heterochromatin proteins such as HP1a and Su(var)3-9. Furthermore, they demonstrate that overexpression of roX RNAs leads to ISC hyperplasia, while their inactivation mitigates dysplasia and extends lifespan. While the study presents interesting and potentially impactful findings, I have the following concerns:

Major Concerns:
1. The authors claim ISC hyperproliferation based on the number of ISC/EBs, using esg>GFP+ cells as a readout. However, GFP+ cells represent both ISCs and EBs. To more accurately assess proliferation, I recommend quantifying mitotic ISCs using a specific mitosis marker such as PH3 across all relevant conditions.
2. Colocalization of HP1a and roX2 RNA: In line 294, the authors state that HP1a is colocalized with roX2 RNA. However, the figure does not clearly demonstrate this. It would strengthen the claim to highlight the colocalized signals.
3. Mechanistic validation of roX2 RNA function: Since the authors propose that roX2 RNA recruits HP1a and Su(var)3-9 to induce heterochromatin formation, it would be important to test whether the phenotypes caused by roX2 RNA overexpression can be rescued by knockdown of HP1a or Su(var)3-9. This would provide stronger evidence for the functional relevance of these interactions.

Minor Concerns:
In the Methods section, the sources of fly stocks and antibodies should be clearly specified to improve reproducibility.

Referee #3:

In this manuscript, Kai-Le Li et al. investigate a novel role for the long noncoding RNAs roX1 and roX2 in promoting heterochromatin expansion in Drosophila intestinal stem and progenitor cells (ISCs/EBs) under conditions of pathogen-induced stress and aging. Using scRNA-seq, immunofluorescence imaging, RNA pulldown, and functional genetic assays, the authors show that roX RNAs are upregulated in female ISCs during stress, where they promote heterochromatinization potentially through interactions with HP1α and Su(var)3-9. Based on overexpression and RNAi experiments, the authors propose that roX1/roX2 RNAs are necessary and sufficient for driving increased heterochromatinization, ISC hyperplasia, and reduced lifespan in females upon infection or aging, revealing a role for these lncRNAs beyond their classical function in male dosage compensation. The authors further report that human Xist RNA can functionally substitute for roX1/roX2 RNA in LPS-treated female cells, and that Xist expression is significantly elevated in aged human female blood cells compared to young. These observations are used to suggest a conserved role for these lncRNAs in stress- or age-induced heterochromatinization across species.
While the study presents an intriguing new role for roX RNAs in heterochromatin regulation, key mechanistic and technical issues limit the strength of the authors' conclusions. In particular, the causal role of roX RNAs in heterochromatin expansion is not fully supported in the absence of rescue experiments. The proposed mechanism by which roX RNAs directly recruit heterochromatin-associated proteins such as HP1α and Su(var)3-9 to drive heterochromatinization is insufficiently supported by the current data. Moreover, the apparent restriction of roX upregulation to ISCs raises important questions about the cell-type specificity of both the chromatin changes and the underlying mechanism reported under stress conditions. The authors should address these limitations by including a concise discussion of possible mechanisms that could explain their observations. While such mechanisms need not be presented as definitive conclusions, framing the findings within a broader biological context would enhance the manuscript's scientific rigor. Additional quantitative analyses, appropriate controls and more experiments are required to strengthen the manuscript's central claims. These concerns should be addressed in a revision.
Major points:

1. The authors report chromatin state changes in female ISC/EBs upon PA14 infection and aging but do not clarify whether these findings also apply to male flies. Are HP1α, Pol II Ser2ph, and histone marks similarly affected in response to infection or aging in males? Addressing this would be important to determine whether the observed effects and underlying mechanisms suggested are sex-specific.
2. Quantification of chromatin marks (Figs. 1, 3, 5) is limited to Esg^+ cells, which include both ISCs and EBs. Given that Delta staining is available, the authors should stratify their analysis into Esg^+Delta^+ (ISCs), Esg^+Delta^- (EBs), and Esg^-Delta^- (other cells) to clarify whether heterochromatin changes are ISC/EB-specific or more widespread across the gut epithelium.

3. In Fig. 2B: The observed reductions in UMI and gene counts may reflect technical factors such as sequencing depth, RNA integrity, or cell viability, rather than true global transcriptional downregulation. Please provide appropriate quality control metrics (e.g., sequencing depth, % mitochondrial reads, cell viability) to support a biological interpretation. Figs. 2C-D: Please indicate the total number of differentially expressed genes (DEGs) analyzed in each condition to contextualize the proportions shown.
4. Throughout the manuscript, there is a general lack of validation for the expression levels of key genetic constructs. For instance, the newly generated UAS-roX2 transgenic line is not validated by qRT-PCR, and no data are shown confirming the efficiency of roX1/2 overexpression or knockdown in either the Drosophila gut or Kc cells. Similarly, expression of the Xist constructs in Kc cells is not assessed. This issue also extends to the various stable Kc cell lines expressing GFP-tagged HP1α domain constructs, where expression is not validated at the RNA or protein level (e.g., via qPCR or Western blot). These validations are essential to support the interpretations drawn from the phenotypic and biochemical assays, and should be included for all gain- and loss-of-function experiments.
5. For the RNAi-based knockdown experiments targeting roX1 and roX2, appropriate mock or control RNAi lines are not included in most assays, with the exception of the lifespan experiments. This also applies to the Kc cell-based RNAi experiments. These controls are essential.
6. Although both ISCs and EBs exhibit increased HP1α and heterochromatin marks with aging or infection, the upregulation of roX1/2 appears to be largely restricted to Delta$^+$ ISCs (Fig. 2E). Could the authors clarify what proportion of Delta$^+$ ISCs show detectable or elevated roX1/2 RNA expression in young vs old and uninfected vs infected conditions? Additionally, since roX1/2 expression is limited to ISCs, this raises the question of how heterochromatin expansion is induced in Delta$^-$ Esg$^+$ EBs. Is this process independent of roX RNAs? The authors should discuss whether alternative pathways, such as inflammatory signaling or other stress-responsive epigenetic regulators, could contribute to the observed heterochromatin expansion and hyperplasia in both cell types under stress or aging conditions.
7. To establish a causal role for roX RNAs in promoting heterochromatinization during infection or aging in females, a key experiment is missing: a rescue assay. Specifically, the authors should test whether re-expression of roX2 in a roX2 RNAi background can rescue the loss-of-function phenotype. This would directly demonstrate the specificity of the RNAi effect and strengthen the conclusion that roX2 is both necessary and sufficient for inducing heterochromatinization in this context.
8. In females, roX RNAs are known to be expressed at very low basal levels. Upon infection or aging, while an increase is reported in the ISCs, it remains unclear how substantial this upregulation is. Do the roX RNA levels approach those observed in males, or do they remain relatively low? Additionally, where do roX1/2 rank in terms of expression relative to other genes in the ISC transcriptome in these conditions? Clarifying the magnitude and context of this upregulation would help interpret their sufficiency and relevance in driving chromatin changes.
9. Since roX RNAs are associated with the MSL complex in male cells and require components like MLE and MSL2 for their stability and expression, have the authors assessed the expression levels of MSL complex proteins in females upon infection or aging? This analysis would clarify whether roX RNAs act independently of the MSL complex in females, or whether partial MSL complex activity may be reactivated under these conditions.
10. For the roX1 RNA pulldown experiments, the authors should clarify in the main text that crosslinking was performed using formaldehyde. Given this method, the interactions detected between roX1 RNA and proteins such as HP1α and Su(var)3-9 may be indirect, and this limitation should be acknowledged. The amount of roX1 RNA recovered in the pulldown ({plus minus}LPS) should be quantified by qRT-PCR, and specificity should be assessed by comparing enrichment over an unrelated RNA (e.g., U1 snRNA or GAPDH mRNA). Input and IP samples should be loaded on the same blot with indication of the % input loaded. The Su(var)3-9 blot is difficult to interpret due to technical issues and should be repeated. A loading control such as histone H3 or H4 should be included, and the number of replicates reported.
11. The authors should perform roX2 RNA FISH in male cells as a positive control for signal specificity and localization. In the current images, the observed colocalization between roX2 and HP1α appears weak and should be quantitatively assessed (Fig. 7A). Fig. S7B also lacks quantifications. The differences in H3K9me3 staining intensity upon roX2 overexpression are difficult to interpret based on the current images, as they could result from sample-to-sample variability. Given the limited evidence for direct interaction and modest colocalization, the authors should significantly tone down their claim that roX2 RNA directly recruits HP1α and other heterochromatin proteins. At present, the data do not support this conclusion. Strengthening this claim would require additional experiments such as HP1α ChIP or roX2 ChIRP to demonstrate physical co-occupancy at heterochromatin loci.
12. Xist RNA expression is not significantly upregulated in aged versus young females in endothelial cells, as shown in Fig. S7F. This should be clearly stated in the main text. Additionally, the RT-qPCR validation was performed using human blood cells, which are developmentally and functionally distinct from the intestinal stem cells (ISCs) studied in Drosophila. These differences raise questions about the cell-type specificity of the observed effects. Can the authors comment on this?

Minor points:

1. To account for consistency of immunostaining across cells and biological replicates, the authors normalize signal intensity to DAPI as an internal control. However, given that DAPI staining is influenced by chromatin state, its use as a normalization reference may be problematic in a study focused on heterochromatinization. A more stable control, such as total histone H3 or H4, might be more reliable.
2. In several quantifications, the authors report the number of nuclei analyzed (n) but do not specify the number of independent biological replicates (N). Please ensure that the number of biological replicates is clearly indicated for all quantified experiments.
3. Since many experiments rely on Esg-Gal4/Delta-Gal4 to drive expression in ISCs and EBs, it is important to confirm that its activity remains stable across experimental conditions. Have the authors assessed whether Esg-Gal4/Delta-Gal4 driver activity

is altered upon PA14 infection or during aging? A simple control experiment using UAS-GFP could clarify this.
4. The authors observe changes in heterochromatin markers (HP1α, H3K9me3) by immunofluorescence in the gut and use FACS to isolate GFP^+ ISCs/EBs for scRNA-seq. Can the authors use the FACS-sorted ISCs/EBs for Western blot analysis of HP1α or H3K9me3 upon infection or aging? This would provide direct biochemical validation of the chromatin changes observed by IF.
5. The authors should perform Gene Ontology (GO) term enrichment for the differentially expressed genes (Fig. 2C) identified in each comparison (young vs. aged and uninfected vs. PA14-infected). This would help determine whether similar biological pathways are affected under both conditions.
6. In Fig. 2E, the authors should clarify what the scale represents (RPKM, Z-score etc.)
7. In Fig. 4C, the volcano plot shows a large number of upregulated genes upon roX2 knockdown in PA14-infected cells, but the scale compresses the visibility of individual DEGs. The authors should provide a zoomed-in version of the plot (e.g., focusing on log2 fold changes between -5 and +5) to better visualize and interpret the distribution and identity of the DEGs. Additionally, it would be helpful to present a version of the plot using a higher log2 fold change threshold (e.g., {plus minus}0.5 or {plus minus}1) to highlight genes with more substantial expression changes.
8. In Fig. 4F, no statistical significance tests appear to be included in the panel, yet the text describes the differences as statistically significant. Please provide appropriate statistical analysis.
9. In Figs 5D and 5E, the colors used to represent different chromosomes are inconsistent between the pie chart and the bar graph. The authors should standardize the color scheme across both panels.
10. In males, roX1 and roX2 are known to function redundantly in dosage compensation. It remains unclear whether a similar functional redundancy exists in females with respect to their role in promoting heterochromatinization. To address this, the authors should consider performing a double knockdown of roX1 and roX2 and assessing whether the combined depletion has an additive effect on heterochromatin markers and ISC phenotypes compared to single knockdowns.
11. Can the authors use FACS to quantify $Delta^+$ and Esg^+ cells under infection or aging conditions, using their existing Delta-Gal4 and Esg-Gal4 lines in combination with UAS-GFP? This would serve as an orthogonal and more quantitative approach to support the immunofluorescence-based findings and strengthen conclusions about ISC/EB population dynamics.
12. For the RNAi experiments in Kc cells, the methods section describes two distinct approaches : shRNA and dsRNA. The authors should clarify which RNAi method was used in each case and provide data demonstrating the knockdown efficiency for all relevant experiments.
13. The manuscript refers to Xist RNAs as homologs of roX RNAs at multiple points. This is inaccurate. Please correct this.
14. In Fig. EV7B, the reported increase in HP1α and H3K9me3 at euchromatic arms should be more clearly indicated, for example by using arrowheads in the figure. Additionally, to confirm that roX2 is indeed overexpressed, the authors should perform roX2 RNA FISH or use an alternative method to validate overexpression.
15. The authors state in the discussion that MSLs are absent in females. This is not accurate. MSL1, MSL3, MOF and MLE are also expressed in females, albeit at low levels 1,2. roX2 pulldown-MS based experiments in both male and female cells may help assess which proteins are preferentially bound by the RNA in the different cases.
16. The statement that HP1α shows enhanced interaction with the U1 and D1 regions after LPS treatment may be misleading. This apparent increase could simply reflect elevated HP1α protein levels under LPS conditions, rather than a specific change in binding affinity or localization. To address this, the authors should quantify the expression levels of all HP1α constructs in both LPS^+ and LPS^- conditions, as well as the input levels of roX1 RNA.
17. Without appropriate expression levels of Xist RNA fragments in Drosophila ISC/EB cells, it is difficult to determine whether the lack of rescue by Xist-D is due to insufficient expression, reduced RNA stability, or an inherent lack of functional activity (Fig. 7). Along similar lines, the authors should express an unrelated lncRNA as a control, to ensure the observed effects are unique to roX/Xist RNAs.
18. The Gal4 lines used must be consistently mentioned for all the main figures and extended figures (for example- Fig.1 vs Fig. 5).
19. Line 174 needs to be supported with an appropriate citation.
20. The heading in line 304 needs to be reworded for clarity
21. Please provide access to the code used for data analysis such as your scRNA-seq in a public repository such as github, or as supplementary files.

References

1. Kelley, R. L. et al. Expression of msl-2 causes assembly of dosage compensation regulators on the X chromosomes and female lethality in Drosophila. Cell 81, 867-877 (1995).
2. Gorman, M., Franke, A. & Baker, B. S. Molecular characterization of the male-specific lethal-3 gene and investigations of the regulation of dosage compensation in Drosophila. Development 121, 463-475 (1995).

Point-by-point response (in blue) to reviewers' comments (in black):

Referee #1:

Overall summary and major criticisms

This very intriguing study finds a role for the noncoding Rox RNAs in heterochromatization in aging and stressed female midguts. The authors present some convincing data but are prone to overinterpretation. Several findings that counter well-established features of heterochromatin or dosage compensation are briefly presented but not further commented on. While the authors may indeed have discovered remarkable new properties of these systems, they owe the reader a more in-depth treatment. The authors might consider introducing a graphical model of the relationship between RNA, heterochromatin, stress, hyperplasia and ageing in the main body of the paper. Something akin to S8 but simpler.

Response: We thank the reviewer for taking time to review our paper and giving valuable comments. We have plotted a graphic about the novel relationship between RNA, heterochromatin, stress, hyperplasia, and ageing in Figure 8 of our revised manuscript, which enables it to be more straightforward for readers.

The primary claim to novelty is a non-canonical role for Rox RNA in female flies, but the authors fail to address previous studies revealing repressive, autosomal roles of these RNAs. Early this year a study reporting association of Rox RNA with Polycomb complexes, and a role for Rox RNA in repression of developmental genes, was published (doi.org/10.1038/s41467-024-55711-y). Intriguingly, the affected genes have female-biased expression. Rox mutations have also been found to suppress variegation of insertions in autosomal heterochromatin (10.1534/genetics.109.102087; 10.1371/journal.pone.0140259). In contrast with the present study, suppression of variegation and misregulation of heterochromatic genes was limited to males. It would be a definite contribution to the field if the authors put forth a model that incorporates previously reported aspects of autosomal gene regulation with their current findings!

Response: Thanks for reviewer's comments, we have added previous work into the discussion part.

Previous studies have revealed the non-canonical function for autosomal gene repression of *roX* RNA (Li *et al*, 2025; Deng *et al*, 2009; Koya & Meller, 2015), and our work significantly extends this understanding by revealing that *roX* RNAs interact with heterochromatin proteins to promote heterochromatin formation. Specifically, under conditions of infection or aging in females, the increase of *roX* RNAs triggers broad gene repression, destroys intestinal homeostasis, and impacts flies lifespan. Based on our findings, a model was proposed, *roX* RNAs act as multifacial non-coding RNAs whose functions depend on specific conditions, including sex and physiological stress. In males, *roX* RNAs primarily function in dose compensation to activate gene expression. In females, under normal conditions, *roX* RNAs are expressed at relative low levels; however, under stress such as infection or aging, the expression is activated, where they play transcriptional repression via heterochromatinization.

We have now incorporated this interpretation into our revised Discussion section at in line 408-427.

The markers used for heterochromatin formation - HP1a and H3K9me3 have little resolution. As the authors know the genes affected, a chromatin accessibility assay like ATAC seq would provide confirmation of changes in chromatin.

Response: As reviewer suggested, we performed ATAC-seq experiments for high resolution of genome status change. ATAC-seq demonstrated that *roX*2 overexpression resulted in decreased chromatin accessibility compared to the GFP control. We incorporated the respective results in our revised manuscript for Figure 6K.

Rox1 and Rox2 are redundant for dosage compensation, but many of the studies presented here knockdown just one. Could the

authors speculate on why the single knockdowns achieve identical results? They also present the striking finding that knockdown of both Rox RNAs with a strong driver has no effect on males but extends female lifespan. As the endogenous levels of Rox are vastly different in the two sexes, measurement of total RNA after knockdown might be informative.

Response: Previous studies on *roX*1 and *roX*2 in males showed no visible phenotypes when one of the genes was mutated, most likely due to compensation during early development, while double mutation caused severe developmental defects, leading to larval lethality. In females, even a double mutation did not affect visible development. Here, we temporally and spatially knocked down *roX*1 or *roX*2 individually in the female ISCs/EBs, and observed no obvious defects, which is consistent with previous mutagenesis assays. However, upon pathogen treatment, the cellular requirement for *roX* RNAs increases to silence certain genes. Under such conditions, temporally knocking down a single *roX* RNA may not be sufficiently compensated for by the other, leading to the observed gut phenotypes.

Additionally, we validated the knockdown efficiency of *roX1* and *roX2* via qPCR. In both males and females, Transcript levels of *roX1* and *roX2* were significantly reduced following their respective knockdowns (please see Appendix Figure S6B-C).

Referring to Xist as a Rox homolog suggests an evolutionary relationship that does not exist (line 42). Please refine this description to acknowledge similarities between molecules that lack a shared ancestor. Drosophila is very unlikely to provide proper molecular context for Xist behavior in cells. In stretching for parallels between Xist and Rox the authors do a disservice to their work.

Response: Thanks for the reviewer's comment, we have refined the description by replacing "homolog" with "functional analogue". Respective change in our revised manuscript is line 42.

Female life extension by knockdown of a single Rox gene (Figure 5) is very intriguing! If this is indeed true, the authors should be able to replicate this finding with Rox mutants.

Response: Thanks for the reviewer suggestion, we placed the order for the mutation lines from Bloomington, however, we did not get them due to customs restrictions. In addition, we are on the way to generating mutants with CRISPR technology.

We are very confident about the knockdown results, we have two different shRNA transgenic RNAi lines against either *roX*1/*roX*2, and they generated similar phenotypes, such as rescuing the hyperproliferation of ISCs/EBs under stressed conditions to a normal level, indicating this is not an off-target effect. On the other hand, the knockdown and overexpression show opposite defects in the *Drosophila* gut, further limiting the off-target effect. Furthermore, both *roX*1 and *roX*2 knockdowns show expanded lifespan in female flies, most importantly, the rescue experiments by overexpression of *roX*2 in *roX*2 knockdown background can significantly rescue the *roX*2 knockdonw generating the reduction of ISCs/EBs number under stressed conditions. Altogther, above experiments support the specificity of the knockdown.

Figure S7 is problematic on several levels. Panel B presents antibody staining of three preparations as justification for the claim that Rox overexpression expands heterochromatin, and this is reversed by RNase. There is no indication of scoring criteria or number of preparations examined. Given the variability between preparations done on the same genotype, this is flimsy evidence for the claim that RNase treatment reduces HP1 localization.

Response: We thank the reviewer for the rigorous comments! We added the scoring criteria, if more than two foci beyond the main constitutive heterochromatin foci, it was characterized as heterochromatin expansion. According the criteria, we have quantified the changes of heterochromatin after *roX*2 overexpression, with the number of experimental preparations examined indicated. Please see Figure EV4C-D in our revised manuscript for more details.

Minor criticisms

Explain what delta antibody recognizes and why it is used as a marker for stem cells.

Response: The Delta antibody recognizes the Delta protein, which is a paracrine ligand secreted from ISCs that activates the Notch signaling pathway in adjacent progenitor cells, thus Delta is widely used marker for ISCs.

Lines 154-156. Are all the midguts from females?

Response: Yes, all the guts are from female *Drosophila*.

Lines 157-158. The authors state that overall gene expression decreases, but do not explain how reads are normalized. How do they know that some preparations simply had less mRNA?

Response: Thanks for the comment. After filtering datasets, methods normalizing total from scanpy and numpy.log1p were used for normalization. Total detected reads were normalized to same level and calculated ln(x+1) value. This approach ensures that differences in gene expression levels reflect biological variation rather than technical differences in mRNA input.

Line 191. Explain why the esg driver is used.

Response: As *Esg*-Gal4 is is specifically expressed in stem cells and their progenitor cells based on immunostaining assays, which is currently used to study the homeostasis of intestine stem cells.

Lines 213-214 appears to lack citations for Notch activation.

Response: Thanks for your comment, we have added the citations for Notch activation (dio: 10.1016/j.cell.2009.05.014). Respective change can be found in the line 249 in our revised manuscript.

Line 300-302 states that Rox RNA, not H3K9me3, is responsible for HP1 recruitment. This is a novel suggestion that upends conventional wisdom about HP1 properties. See major criticism about the quality of data this statement is based on.

Response: In our work, based on the scoring criteria and number of preparations examined in the Figure EV4C-D, we found the HP1a foci on the euchromatic arms can be reverted to the levels of the control without a noticeable reduction in H3K9me3 after RNase A treatment, indicating that certain HP1a associates on chromatin depends on *roX* RNAs, while the majority of HP1a still relies on H3K9me3. This likely represents a dynamic regulatory mechanism for facultative heterochromatinization in euchromatic regions, allowing the organism to respond to environmental stress.

Line 323 refers to detection of Xist in blood. This is presumably a byproduct of cell death and might be a better marker of inflammation and apoptosis than of cellular levels of Xist.

Response: We thank the reviewer for raising this point. Total RNA was extracted from 400 µL of whole blood collected from each donor. The samples were immediately lysed in Trizol, followed by RNA extraction using a standard protocol. In this case, we cannot limit the possibility that *Xist* is a byproduct of cell death. To limit the above possibility, the fresh blood is centrifigated and remove the plasma, then with Trizol to lysis entire blood cells, which may give the level of *Xist* in living blood cells. We have therefore incorporated this perspective into the revised Discussion (line 435-438).

Line 326 suggests a false relationship between Xist and Rox RNAs.

Response: We thank the reviewer for this comment. We have revised the description to: "Altogether, the above results support the role of *roX* RNAs in heterochromatinization is functionally analogous to that of *Xist* in humans." (Line 380-381 in our revised manuscript).

Line 363 "Conversely, the absence of such regulators, such as MSL or MOF, in the female . . . " needs to be more specific. The only MSL absent in females is MSL2, and even this is controversial.

Response: Thanks for the referee pointing out, we refined the description more specifically from "the absence of such regulators, such as MSL or MOF" to "the absence of a functional MSL". Respective change can be found in line 421 in our revised manuscript.

Lines 368 -71 double down on functional equivalency between Xist and Rox, suggesting that Xist regions interacting with PcG can rescue Rox function. Do the authors believe that these regions interact with PcG or HP1 in flies?

Response: Actually, we performed the *in vitro* experiments by *roX*2 knockdown after LPS treatment, then expressed *Xist*-C region, which includes the region that interacts with the PcG group. Interestingly, the overexpression can rescue the knockdown-triggered condensed heterochromatin to expanded status, suggesting the *Xist*-C region functions similarly to *roX* RNAs, which may also interact with HP1a in flies. We will perform biochemical experiments to confirm the interaction of between *Xist*-C and PcG or HP1a in fly, which will be put in next manuscript.

Figure 2. Please label the scale bar in 2E. A more complete description of the comparisons presented in this figure would be appreciated. What do the red circles represent? For Figures 2(F) and 2(G) and their corresponding texts, it would be easier for the readers to appreciate the magnitude of significance if specific fold changes for the qPCR validation were mentioned.

Response: We thank the reviewer for this important suggestion. We have now clearly labeled the scale bar in Figure 2E as "log1p". The scale indicates the log1p-transformed normalized expression value of the indicated gene (this is also indicated in the corresponding figure legend).

The red circles highlight the the regions in the UMAP where cells form a distinct cluster with detectable Delta expression (Delta-positive cells). Figure 2E visualizes UMAP embeddings colored by the normalized expression of Delta and *roX*1/*roX*2 in both young and aged samples. Under aged conditions, we observe a clear increase in the expression of both Delta and *roX* RNAs. Notably, the upregulation of *roX*1 and *roX*2 is preferentially concentrated within the Delta-positive compartment (marked by red circles), indicating that these RNAs are selectively induced in ISCs during ageing.

We have now included the quantitative fold-change values from the qPCR validation in the main text for Figures 2(F) and 2(G). The revisions are as follows: As for the aged flies, the expression of *roX*1 and *roX*2 was significantly upregulated by approximately 2-fold and 3-fold, respectively. Upon PA14 infection, the expression of *upd3*, which is a positive marker for infection, was robustly induced by approximately 3-fold, suggesting the infection was successful. Then we detected both *roX*1 and *roX*2, their expression in ISCs/EBs showed a significant increase of approximately 1.5-fold. Detailed annotations have been added to the revised manuscript (line 187-191).

In figure 4E the authors find that genes on the 4th chromosome increase expression following Rox2 KD. As this chromosome is famously heterochromatic, this is of interest but not commented on by the authors.

Response: Thanks for referee's appreciation, this is indeed interesting, 4th chromosome is a famous heterochromatic, which is enriched in HP1a, after pathogen treatment, the increase of *roX* RNAs will easily associate with HP1a there on 4th chromosome to further condense heterochromatin and silence target genes. If knockdowning *roX* RNAs, more *roX* RNAs will be depleted from

4th chromosome relative to other chromosomes, then resulting in more numbers of genes upregulated. We have discussed in the revised manuscript in line 232-235.

Referee #2:

In this manuscript, Li et al. investigate the role of long noncoding roX RNAs in regulating heterochromatin formation in intestinal stem cells (ISCs) and enteroblasts (EBs) in *Drosophila*. They report that elevated expression of roX RNAs in ISCs/EBs promotes heterochromatinization and represses gene expression by directly recruiting heterochromatin proteins such as HP1a and Su(var)3-9. Furthermore, they demonstrate that overexpression of roX RNAs leads to ISC hyperplasia, while their inactivation mitigates dysplasia and extends lifespan. While the study presents interesting and potentially impactful findings, I have the following concerns:

Major Concerns:

1. The authors claim ISC hyperproliferation based on the number of ISC/EBs, using esg>GFP+ cells as a readout. However, GFP+ cells represent both ISCs and EBs. To more accurately assess proliferation, I recommend quantifying mitotic ISCs using a specific mitosis marker such as PH3 across all relevant conditions.

Response: Thanks for your important suggestion. To specifically investigate the proliferation status of ISCs, we performed immunostaining with antibodies against Delta and PH3 under all relevant conditions. First, we confirmed the hyperproliferation of ISCs in aged or pathogen infected condition, both showing significant elevated proportion of double positive for Delta and PH3 signals. Most importantly, after the depletion of *roX* RNAs, the proportion of double positive ISCs was reverted to normal level. Conversely, the overexpression of either *roX* RNAs or *Xist-A* promoted ISC proliferation, resulting in a significant increase in the number of both Delta-positive and PH3-positive cells. Collectively, all these results further confirmed that *roX* RNAs overexpression leads to ISCs hyperproliferation during aging or pathogen infection. Please see Figure EV1D-E, EV2D-E, Appendix Figure S9, Appendix Figure S10B-D.

2. Colocalization of HP1a and roX2 RNA: In line 294, the authors state that HP1a is colocalized with roX2 RNA. However, the figure does not clearly demonstrate this. It would strengthen the claim to highlight the colocalized signals.

Response: We thank the reviewer for this suggestion. We have included a zoomed-in image of the specific region to show the colocalization of HP1a and *roX*2 RNA, as indicated by the white arrows. The colocalization ratio between the two signals was quantified, and statistical analysis revealed a significant increase in colocalization following *roX*2 overexpression. We have incorporated these results in our revised manuscript, you can see more details in Figure 7A-B and respective figure legend.

3. Mechanistic validation of roX2 RNA function: Since the authors propose that roX2 RNA recruits HP1a and Su(var)3-9 to induce heterochromatin formation, it would be important to test whether the phenotypes caused by roX2 RNA overexpression can be rescued by knockdown of HP1a or Su(var)3-9. This would provide stronger evidence for the functional relevance of these interactions.

Response: Thanks for your rigorous suggestion. We are on the way to distinguishing the specific role of *roX* RNAs in heterochromatin expansion, especially exploring the role of HP1a and Su(var)3-9 playing in the process. We will timely incorporate new findings in our subsequent work.

Minor Concerns:

In the Methods section, the sources of fly stocks and antibodies should be clearly specified to improve reproducibility.

Response: We thank the reviewer for this suggestion. As requested, we have now clearly specified the sources for all fly stocks and antibodies used in this study. This information has been comprehensively detailed in the Reagents and Tools Table included in the Methods section.

Referee #3:

In this manuscript, Kai-Le Li et al. investigate a novel role for the long noncoding RNAs roX1 and roX2 in promoting heterochromatin expansion in *Drosophila* intestinal stem and progenitor cells (ISCs/EBs) under conditions of pathogen-induced stress and aging. Using scRNA-seq, immunofluorescence imaging, RNA pulldown, and functional genetic assays, the authors show that roX RNAs are upregulated in female ISCs during stress, where they promote heterochromatinization potentially through interactions with HP1α and Su(var)3-9. Based on overexpression and RNAi experiments, the authors propose that roX1/roX2 RNAs are necessary and sufficient for driving increased heterochromatinization, ISC hyperplasia, and reduced lifespan in females upon infection or aging, revealing a role for these lncRNAs beyond their classical function in male dosage compensation. The authors further report that human Xist RNA can functionally substitute for roX1/roX2 RNA in LPS-treated female cells, and that Xist expression is significantly elevated in aged human female blood cells compared to young. These observations are used to suggest a conserved role for these lncRNAs in stress- or age-induced heterochromatinization across species.

While the study presents an intriguing new role for roX RNAs in heterochromatin regulation, key mechanistic and technical issues limit the strength of the authors' conclusions. In particular, the causal role of roX RNAs in heterochromatin expansion is not fully supported in the absence of rescue experiments. The proposed mechanism by which roX RNAs directly recruit heterochromatin-associated proteins such as HP1α and Su(var)3-9 to drive heterochromatinization is insufficiently supported by the current data. Moreover, the apparent restriction of roX upregulation to ISCs raises important questions about the cell-type specificity of both the chromatin changes and the underlying mechanism reported under stress conditions. The authors should address these limitations by including a concise discussion of possible mechanisms that could explain their observations. While such mechanisms need not be presented as definitive conclusions, framing the findings within a broader biological context would enhance the manuscript's scientific rigor. Additional quantitative analyses, appropriate controls and more experiments are required to strengthen the manuscript's central claims. These concerns should be addressed in a revision.

Response: We sincerely thank the reviewer for their thoughtful and constructive feedback on our manuscript. The comments have been invaluable in helping us strengthen our study and clarify our conclusions. We have conducted additional experiments and analyses to address the points raised, as detailed below.

Major points:

1. The authors report chromatin state changes in female ISC/EBs upon PA14 infection and aging but do not clarify whether these findings also apply to male flies. Are HP1α, Pol II Ser2ph, and histone marks similarly affected in response to infection or aging in males? Addressing this would be important to determine whether the observed effects and underlying mechanisms suggested are sex-specific.

Response: We thank the reviewer for this suggestion. To address it, we performed immunofluorescence assays with PA14-treated or aged male *Drosophila*. Consistent with the findings in female *Drosophila*, both aging and PA14 infection led to expanded heterochromatin in ISCs/EBs for male flies, as evidenced by significantly increased levels of heterochromatin markers, H3K9me3 and HP1a, and decreased levels of Pol II Ser2ph. Respective data has been incorporated into Appendix Figure S3.

2. Quantification of chromatin marks (Figs. 1, 3, 5) is limited to Esg⁺ cells, which include both ISCs and EBs. Given that Delta staining is available, the authors should stratify their analysis into $Esg^{+}Delta^{+}$ (ISCs), $Esg^{+}Delta^{-}$ (EBs), and $Esg^{-}Delta^{-}$ (other cells) to clarify whether heterochromatin changes are ISC/EB-specific or more widespread across the gut epithelium.

Response: We appreciate the important suggestion. We stratified our analysis into three different types of cells as $Esg^{+}Delta^{+}$ (ISCs), $Esg^{+}Delta^{-}$ (EBs), and $Esg^{-}Delta^{-}$ (other cells). Consistent with results shown in our previous manuscript, aging or pathogen infection led to a significant increase in heterochromatin in $Esg^{+}Delta^{+}$ ISCs. More interestingly, we found the $Esg^{+}Delta^{-}$ EBs and $Esg^{-}Delta^{-}$ other cells also exhibited elevated heterochromatin leves, further confirming that the heterochromatin changes are related to aging and pathogen infection in the intestine. We incorporated these results into Appendix Figure S1 in our revised manuscript.

3. In Fig. 2B: The observed reductions in UMI and gene counts may reflect technical factors such as sequencing depth, RNA integrity, or cell viability, rather than true global transcriptional downregulation. Please provide appropriate quality control metrics (e.g., sequencing depth, % mitochondrial reads, cell viability) to support a biological interpretation. Figs. 2C-D: Please indicate the total number of differentially expressed genes (DEGs) analyzed in each condition to contextualize the proportions shown.

Response: We thank the reviewer for raising this important point regarding the potential influence of technical factors on the observed reductions in UMI and gene counts in Figure 2B. We have performed rigorous quality control (QC) on our single-cell RNA-seq data. All samples showed comparable total read counts, around $4x10^{8}$, this effectively rules out differences in sequencing depth as a cause for the reduced UMI/gene counts.

For mitochondrial reads, we used a reference genome excluding the mitochondrial genome to maximize the sequencing reads available for nuclear-encoded genes and to avoid any potential bias from mitochondrial transcripts. However, to definitively rule out the possibility that our results were confounded by differential mitochondrial RNA content, we re-processed the raw data against a standard genome including mitochondrial DNA. This confirmatory analysis demonstrated that the percentage of mitochondrial reads was uniformly low (<5%) and highly consistent across all groups.

Furthermore, cell viability was consistently >90% for all samples prior to library preparation.

Therefore, the comparable sequencing depth, low and consistent mitochondrial reads (<5%), and high cell viability collectively confirm that the reduced UMI and gene counts represent a biological phenomenon of global transcriptional downregulation in aged/infected ISCs/EBs. Please see Responsive Figure 1.

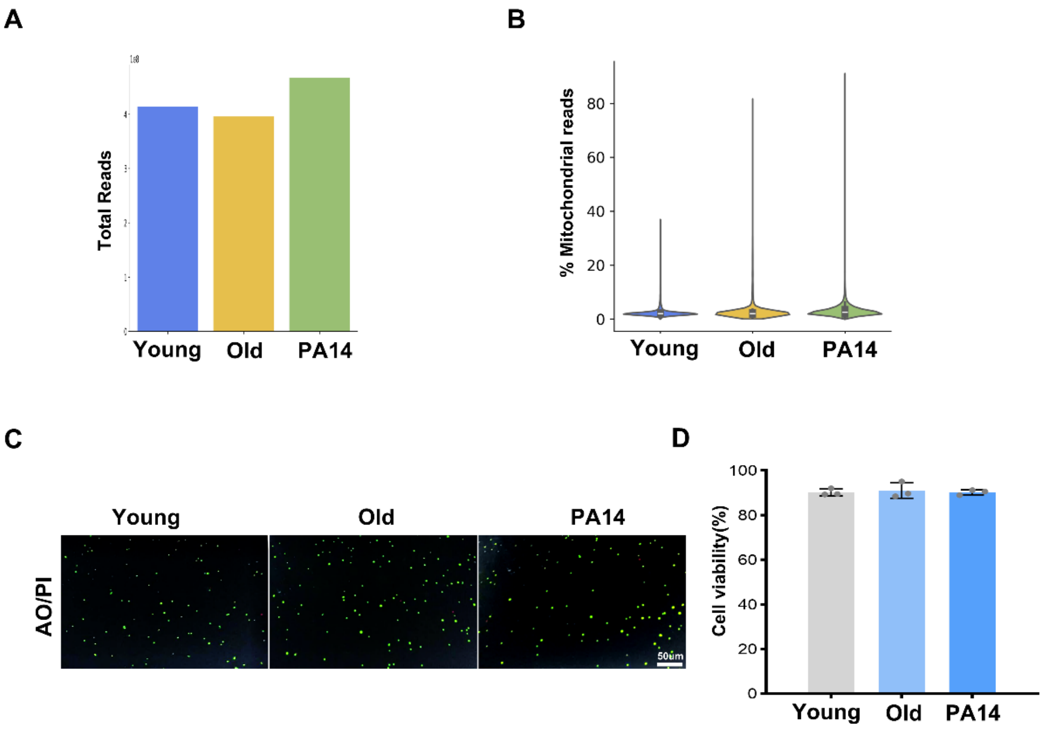

Responsive Figure 1. Quality control metrics for single-cell analysis.

(A) Single-cell RNA sequencing depth and consistency. All libraries were sequenced to a depth of approximately 400 million reads per sample, demonstrating consistent sequencing coverage across all samples. (B) Mitochondrial read percentages were consistently low (<5%) across all groups. (C) Cell viability assay by AO/PI staining. Viable cells fluoresce green (acridine orange, AO), while dead cells are labeled red (propidium iodide, PI). (D) Cell viability was approximately 90% across all groups.

As requested, the total number of up- and down-regulated DEGs for each condition in Figs. 2C-D is now explicitly indicated in the revised figures. Specifically, the analysis identified 221 upregulated and 1223 downregulated genes in aged ISCs/EBs, and 644 upregulated and 4513 downregulated genes in PA14-infected ISCs/EBs. This provides the necessary context for interpreting the scale of the transcriptional changes. Please see revised Figure 2C-D.

4. Throughout the manuscript, there is a general lack of validation for the expression levels of key genetic constructs. For instance, the newly generated UAS-roX2 transgenic line is not validated by qRT-PCR, and no data are shown confirming the efficiency of roX1/2 overexpression or knockdown in either the *Drosophila* gut or Kc cells. Similarly, expression of the Xist constructs in Kc cells is not assessed. This issue also extends to the various stable Kc cell lines expressing GFP-tagged HP1α domain constructs, where expression is not validated at the RNA or protein level (e.g., via qPCR or Western blot). These validations are essential to support the interpretations drawn from the phenotypic and biochemical assays, and should be included for all gain- and loss-of-function experiments.

Response: We appreciate the reviewer's suggestion. We performed qPCR experiments to detect construct efficiency. The transcript level of *roX* RNAs significantly changed after the overexpression or knockdown compared to the control, and we also detected the high expression of *Xist*-A, *Xist*-C, *Xist*-D transcripts, which were nearly undetectable in the control group. In addition, we performed the western blots to examine the different domain expression levels. The blotting results demonstrated

that our expression constructs for different domains of HP1a work well. All these results further confirmed our conclusions in our manuscript. We have incorporated the efficiency examination results into Appendix Figure S6 and Appendix Figure S14 in our revised manuscript.

5. For the RNAi-based knockdown experiments targeting roX1 and roX2, appropriate mock or control RNAi lines are not included in most assays, with the exception of the lifespan experiments. This also applies to the Kc cell-based RNAi experiments. These controls are essential.

Response: Thanks for your important comments, we supplemented the control lines (luciferase RNAi or GFP RNAi) for all RNAi-based knockdown experiments, following changes are shown in Figure 3E-H, Figures 5A-E, Figures 7C-E, and Appendix Figures S9 and S14 in our revised manuscript.

6. Although both ISCs and EBs exhibit increased HP1α and heterochromatin marks with aging or infection, the upregulation of roX1/2 appears to be largely restricted to Delta$^+$ ISCs (Fig. 2E). Could the authors clarify what proportion of Delta$^+$ ISCs show detectable or elevated roX1/2 RNA expression in young vs old and uninfected vs infected conditions? Additionally, since roX1/2 expression is limited to ISCs, this raises the question of how heterochromatin expansion is induced in Delta$^-$ Esg$^+$ EBs. Is this process independent of roX RNAs? The authors should discuss whether alternative pathways, such as inflammatory signaling or other stress-responsive epigenetic regulators, could contribute to the observed heterochromatin expansion and hyperplasia in both cell types under stress or aging conditions.

Response: We thank the reviewer for these insightful questions. In the aged condition, approximately 12% of Delta$^+$ ISCs showed detectable elevation of roX1, and 16% showed detectable elevation of *roX*2. Our data reveal a time-dependent increase in the proportion of Delta$^+$ ISCs expressing *roX* RNAs upon PA14 infection in females. Following a prolonged 6-day PA14 infection, approximately 20% and 16% of Delta$^+$ ISCs exhibited detectable expression of *roX*1 and *roX*2, respectively. Our qPCR results were also consistent with this observation. Please see Responsive Figure 2 and Appendix Figure S4.

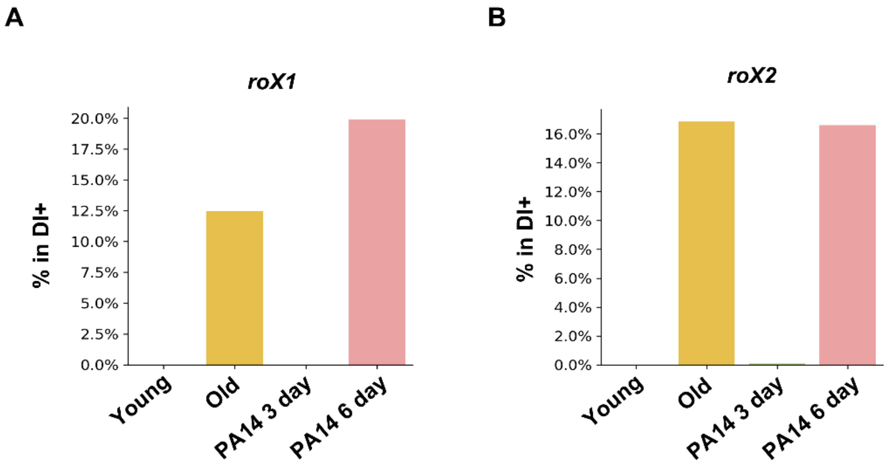

Responsive Figure 2. Proportion of Delta$^+$ ISCs with detectable roX1/2 RNA across conditions.
(A) The percentage of dl+ cells in which roX1 RNA was detected. (B) The percentage of dl+ cells containing detectable roX2 RNA.

The reviewer raises a pivotal point regarding the mechanism of heterochromatin expansion in Delta⁻ Esg⁺ EBs, which lack significant *roX* RNA upregulation. Although *roX*1/2 are largely ISC-restricted, EBs display comparable heterochromatin gains. One explanation is that EBs retain ISC-derived heterochromatin proteins after differentiation. Another possibility is that cytokines or other inflammatory signals from the local microenvironment could activate pathways, such as JAK/STAT, to induce the expression of epigenetic modifiers that promote heterochromatin formation on EBs. Finally, the possibility that other conserved stress-responsive factors, such as histone methyltransferases，might be activated in EBs in response to aging or infection to drive heterochromatinization. (line 440-446).

7. To establish a causal role for roX RNAs in promoting heterochromatinization during infection or aging in females, a key experiment is missing: a rescue assay. Specifically, the authors should test whether re-expression of roX2 in a roX2 RNAi background can rescue the loss-of-function phenotype. This would directly demonstrate the specificity of the RNAi effect and strengthen the conclusion that roX2 is both necessary and sufficient for inducing heterochromatinization in this context.

Response: Thanks for the rigorous suggestion, we have performed the rescue experiments. Specifically, we re-expressed *roX*2 in a *roX*2 RNAi background under pathogen infection conditions. Compared to the *roX*2 depletion, which reduced the heterochromatin level and the proliferation of ISC, re-expression of *roX*2 reinduced the increased heterochromatin levels and proliferation of ISCs, confirming *roX*2 is necessary and sufficient for heterochromatinization under the stressed condition. We have incorporated these results into Appendix Figures S10, S12.

8. In females, roX RNAs are known to be expressed at very low basal levels. Upon infection or aging, while an increase is reported in the ISCs, it remains unclear how substantial this upregulation is. Do the roX RNA levels approach those observed in males, or do they remain relatively low? Additionally, where do roX1/2 rank in terms of expression relative to other genes in the ISC transcriptome in these conditions? Clarifying the magnitude and context of this upregulation would help interpret their sufficiency and relevance in driving chromatin changes.

Response: We thank the reviewer for these insightful questions regarding the magnitude and context of *roX* RNA upregulation. Upon infection, we indeed observed a significant upregulation of *roX* RNA in females. However, the induced expression levels do not match the high levels detected in males. Please see Appendix Figure S5.

We analyzed the fold-change ranking of *roX*1 and *roX*2 among all differentially expressed genes in ISCs. This analysis reveals that *roX*1 and *roX*2 are the top two most strongly upregulated genes in the transcriptome under these conditions. Therefore, we propose that this substantial increase is indeed sufficient to be functionally relevant in driving the observed chromatin changes in females.

Responsive Table 1. *roX1* and *roX2* rank as the top two upregulated genes.

Rank(fold change)	Gene	P value	Log fold cahnge	status
1	*roX1*	0.005986	28.76179	up
2	*roX2*	4.52E-05	27.98375	up

9. Since roX RNAs are associated with the MSL complex in male cells and require components like MLE and MSL2 for their stability and expression, have the authors assessed the expression levels of MSL complex proteins in females upon infection or

aging? This analysis would clarify whether roX RNAs act independently of the MSL complex in females, or whether partial MSL complex activity may be reactivated under these conditions.

Response: Thanks for your rigorous suggestion, we are on the way to detecting the expression change of the MSL complex, we will timely clarify respective findings in our new manuscript.

10. For the roX1 RNA pulldown experiments, the authors should clarify in the main text that crosslinking was performed using formaldehyde. Given this method, the interactions detected between roX1 RNA and proteins such as HP1α and Su(var)3-9 may be indirect, and this limitation should be acknowledged. The amount of roX1 RNA recovered in the pulldown ({plus minus}LPS) should be quantified by qRT-PCR, and specificity should be assessed by comparing enrichment over an unrelated RNA (e.g., U1 snRNA or GAPDH mRNA). Input and IP samples should be loaded on the same blot with indication of the % input loaded. The Su(var)3-9 blot is difficult to interpret due to technical issues and should be repeated. A loading control such as histone H3 or H4 should be included, and the number of replicates reported.

Response: Thanks for the rigorous comments, we have now clarified our methods about the crosslink was used in the *roX*1 RNA pulldown experiments. As appropriately noted by the reviewer, we have also explicitly stated that the detected interactions with HP1α and Su(var)3-9 could be indirect. Accordingly, we have toned down our conclusion by removing direct (line 335).

In addition, we have quantified the recovered *roX*1 RNA by qPCR. The results demonstrate a strong and significant enrichment of *roX*1 RNA in the pulldown compared to the control LacZ. Crucially, this enrichment was specific, as there was no significant pull-down of the control GAPDH mRNA. Please see Responsive Figure 3.

Furthermore, we confirm that the Input and IP samples were indeed loaded on the same blot with indication of the 5% input loaded. Subsequently, the membrane was subsequently sectioned to allow for different exposure times, which was necessary to prevent overexposure of the strong Input signal while adequately capturing the weaker IP signal. This ensures the accurate representation of all data. As requested, we have repeated the Su(var)3-9 blot. The updated result, which includes H3 as a loading control and is representative of three independent replicates, clearly shows the specific enrichment of Su(var)3-9 in the *roX*1 RNA pulldown after LPS treatment. Please see Figure 6F.

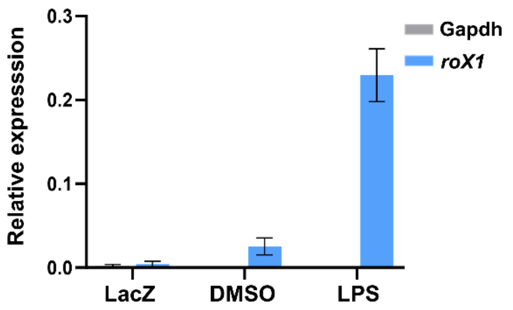

Responsive Figure 3. qPCR quantification to determine the amount of *roX*1 RNA recovered in the pulldown assays under both untreated and LPS-treated conditions. GAPDH mRNA was used as an unrelated RNA control to assess the specificity of the pulldown (N = 3).

11. The authors should perform *roX*2 RNA FISH in male cells as a positive control for signal specificity and localization. In the current images, the observed colocalization between *roX*2 and HP1α appears weak and should be quantitatively assessed (Fig. 7A). Fig. S7B also lacks quantifications. The differences in H3K9me3 staining intensity upon *roX*2 overexpression are difficult to

interpret based on the current images, as they could result from sample-to-sample variability. Given the limited evidence for direct interaction and modest colocalization, the authors should significantly tone down their claim that *roX*2 RNA directly recruits HP1α and other heterochromatin proteins. At present, the data do not support this conclusion. Strengthening this claim would require additional experiments such as HP1α ChIP or *roX*2 ChIRP to demonstrate physical co-occupancy at heterochromatin loci.

Response: We thank the reviewer for their insightful comments and constructive suggestions, which have helped us to strengthen our study. Accordingly, we performed *roX*2 RNA FISH in male salivary gland polytene chromosomes as suggested. The results reveal specific and intense *roX*2 signals that precisely localize to the DAPI-stained chromosomes, which validates the specificity of our FISH assay. Please see Responsive Figure 4.

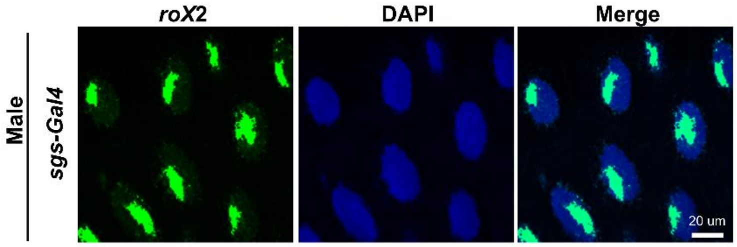

Responsive Figure 4. Validation of *roX*2 RNA FISH specificity and localization in males.
*roX*2 RNA FISH in male salivary gland polytene chromosomes. The experiment was performed using *sgs-GAL4* male flies.

For the colocalization in Fig. 7A, we calculated the colocalization ratio (Pearson's correlation coefficient) between the *roX*2 RNA and HP1a signals. The quantitative analysis revealed a statistically significant degree of colocalization. This data is now included in the revised Fig. 7A-B**.**

For H3K9me3 staining, we quantified its spreading by defining a phenotype where salivary gland chromosomes contained two or more H3K9me3 foci. Analysis of multiple biological replicates revealed a significant increase in this spreading upon *roX*2 overexpression. This quantification is now presented in the revised Figure EV4B-D.

Furthermore, we are grateful to the reviewer for suggesting the crucial experiment of HP1a ChIP to strengthen our claim. As recommended, we also performed RNA immunoprecipitation with HP1a antibody followed by qPCR, a significant increase of *roX* RNAs were observed after LPS treatment (Appendix Fig. S13A-B), indicating enhanced association of HP1a with *roX* RNAs, further supporting the interaction between them. Consistent with this finding and reviewer's comment, we have toned down the language regarding direct recruitment throughout the manuscript. The statement has been revised to describe that "*roX*2 RNA recruits heterochromatin proteins such as HP1a and Su(var)3-9 " (Line 40, 317, 353).

12. Xist RNA expression is not significantly upregulated in aged versus young females in endothelial cells, as shown in Fig. S7F. This should be clearly stated in the main text. Additionally, the RT-qPCR validation was performed using human blood cells, which are developmentally and functionally distinct from the intestinal stem cells (ISCs) studied in *Drosophila*. These differences raise questions about the cell-type specificity of the observed effects. Can the authors comment on this?

Response: We thank the reviewer for this comment. We have updated the main text to clarify that the change in ***Xist*** in aged endothelial cells (Fig. EV4H) was not significant, although an increasing trend was present.

We showed that *roX*2 elevation induces heterochromatin expansion not only in ISCs but also in other cell types like Kc cells and salivary glands, so maybe applicable to many cell types. As an ethic issue, we can only choose blood cells, compared to the young and old, which is similar to our findings in aged *Drosophila*.

Minor points:

1. To account for consistency of immunostaining across cells and biological replicates, the authors normalize signal intensity to DAPI as an internal control. However, given that DAPI staining is influenced by chromatin state, its use as a normalization reference may be problematic in a study focused on heterochromatinization. A more stable control, such as total histone H3 or H4, might be more reliable.

Response: Thanks for this suggestion. We quantified each chromatin marker by its mean fluorescence intensity (mean optical density). Nuclei were first segmented based on the DAPI signal. The total fluorescence intensity of the target protein was then measured within each nucleus using Image J after applying a uniform threshold. The mean fluorescence intensity was calculated as the total intensity divided by the corresponding nuclear area. This value represents the signal density within the nucleus and is independent of DAPI intensity levels. Furthermore, we stained the histone H3 and used it as an internal control, statistical analysis was also consistent with our previous results, further confirming our conclusion. We have modified the method in our manuscript (line 531-535).

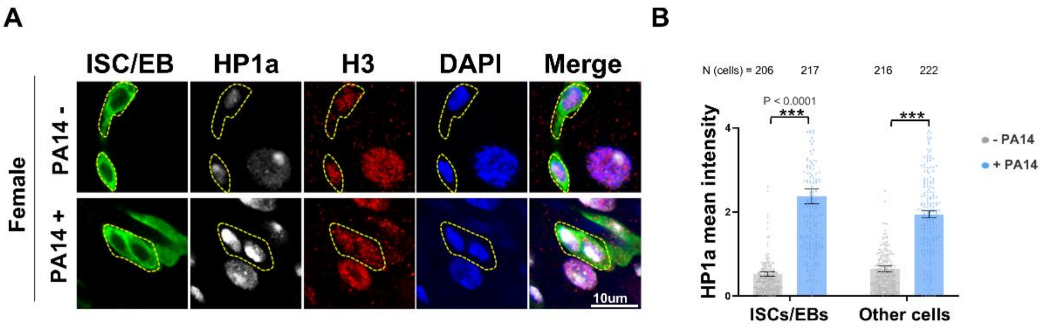

Responsive Figure 5. Consistent immunostaining quantification using H3 normalization.
(A) Representative images showing the HP1a and H3 in ISCs/ EBs (Esg+) or other cells (Esg-) in the midgut from esg-Gal4, UAS-GFP female flies under both uninfected and PA14-infected conditions. (B) Quantification of HP1a intensity in (A). HP1a signal intensity was normalized to H3. N = 3. The number of cells is indicated on the graph. The center values are the averages, and the error bars indicate the s.e.m. P values were obtained by two-tailed unpaired Student's t-test. n.s., not significant, $p \geq 0.05$, *$p < 0.05$, **$p < 0.01$, ***$p < 0.0001$. Scale bar, 10 μm.

2. In several quantifications, the authors report the number of nuclei analyzed (n) but do not specify the number of independent biological replicates (N). Please ensure that the number of biological replicates is clearly indicated for all quantified experiments.

Response: We thank the reviewer for this suggestion. We have clarified the number of independent biological replicates (N>=3) for all quantified experiments, and this information has been included in their respective figure legends.

3. Since many experiments rely on Esg-Gal4/Delta-Gal4 to drive expression in ISCs and EBs, it is important to confirm that its activity remains stable across experimental conditions. Have the authors assessed whether Esg-Gal4/Delta-Gal4 driver activity is altered upon PA14 infection or during aging? A simple control experiment using UAS-GFP could clarify this.

Response: Thanks for your rigorous comments, we assessed the driver activity upon PA14 infection and aging process, as immunostaining experiments results indicated the regular GFP expression level, statistical analysis exhibited no significant change for GFP intensity comparing with control group.

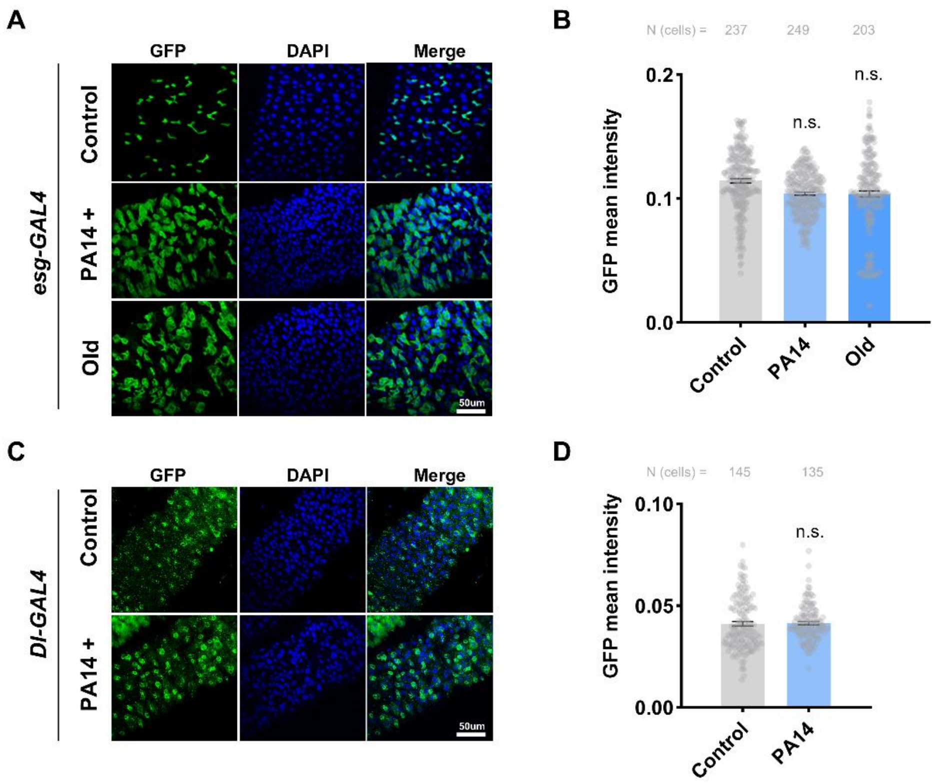

Responsive Figure 6. Assessment of Gal4 driver activity.

(A) Representative images of GFP signal in the midgut from *esg-Gal4, UAS-GFP* female flies under young, aged, and PA14-infected conditions. (B) The mean GFP fluorescence intensity in the conditions shown in (A) was quantified. N = 3. (C) Representative images of GFP signal in the midgut from female flies obtained by crossing *Dl-GAL4* drivers to UAS-GFP lines, under young, aged, and PA14-infected conditions. (D) The mean GFP fluorescence intensity in the conditions shown in (C) was quantified. N = 3. The number of cells analyzed for each group is shown in the figure. The center values are the averages, and the error bars indicate the s.e.m. P values were obtained by two-tailed unpaired Student's t-test. n.s., not significant, $p \geq 0.05$. Scale bar, 50 μm.

4. The authors observe changes in heterochromatin markers (HP1α, H3K9me3) by immunofluorescence in the gut and use FACS to isolate GFP^{+} ISCs/EBs for scRNA-seq. Can the authors use the FACS-sorted ISCs/EBs for Western blot analysis of HP1α or H3K9me3 upon infection or aging? This would provide direct biochemical validation of the chromatin changes observed by IF.

Response: We appreciate the important comments, we performed the western blot analysis for HP1a and H3K9me3 upon infection using gut and GFP-positive cells from FACS, compared to the control, samples from gut or ISCs/EBs sorted from FACS exhibited increased expression levels of HP1a and H3K9me3, providing the direct biochemical validation for chromatin changes observed from IF. We incorporated these results in our revised manuscript, you can find more details in Appendix Figure S2.

5. The authors should perform Gene Ontology (GO) term enrichment for the differentially expressed genes (Fig. 2C) identified in each comparison (young vs. aged and uninfected vs. PA14-infected). This would help determine whether similar biological pathways are affected under both conditions.

Response: We thank the reviewer for this excellent suggestion. We have now performed Gene Ontology (GO) term enrichment analysis on the differentially expressed genes (DEGs) from both the aging and PA14 infection comparisons (Fig. 2C-D), as requested. Most notably, The downregulated genes in both conditions were enriched for terms such as "ATP synthesis" and "ribosome biogenesis", indicating a coordinated decline in metabolic and translational capacity with aging or infection. Notably, the upregulated genes in both conditions were strongly enriched for "DNA-binding transcription repressor activity", which is consistent with our observation of heterochromatin expansion and the global reduction in transcriptional activity in aged or infected ISCs/EBs. Please see Appendix Figure S4**.** We have incorporated these findings into the results section of the revised manuscript, you can find more details in line 176-182. We thank the reviewer again for this valuable suggestion.

6. In Fig. 2E, the authors should clarify what the scale represents (RPKM, Z-score etc.)
Response: We thank the reviewer for pointing out the need for this clarification. The scale in Figure 2E represents log1p (Normalized Expression), where "Normalized Expression" refers to the normalized count data from our single-cell RNA-seq analysis. The log1p transformation (log (1 + x)) is a standard method for visualizing single-cell gene expression data, as it helps stabilize variance and better represent the normalized expression levels on a color scale. We have updated the figure and figure legend for Figure 2E to explicitly state the representation of the scale bar.

7. In Fig. 4C, the volcano plot shows a large number of upregulated genes upon *roX*2 knockdown in PA14-infected cells, but the scale compresses the visibility of individual DEGs. The authors should provide a zoomed-in version of the plot (e.g., focusing on log2 fold changes between -5 and +5) to better visualize and interpret the distribution and identity of the DEGs. Additionally, it would be helpful to present a version of the plot using a higher log2 fold change threshold (e.g., {plus minus}0.5 or {plus minus}1) to highlight genes with more substantial expression changes.
Response: Thanks for the comment. A new zoomed-in panel (please see revised Fig. 4C) has been added, focusing on the region with log2 fold changes between -5 and +5 to provide clearer resolution of individual DEGs. As suggested, we now also present a version of the plot in Appendix Figure S7 that applies a higher significance threshold (fold change threshold = 2), while the original analysis used a threshold of 1.3. This effectively highlights genes with more substantial expression changes upon *roX*2 knockdown.

8. In Fig. 4F, no statistical significance tests appear to be included in the panel, yet the text describes the differences as statistically significant. Please provide appropriate statistical analysis.
Response: We thank the reviewer for this important comment. The statistical comparisons were carried out using the Wilcoxon test. The updated version of Figure 4F now incorporates the standard asterisk notation to denote statistical significance, and the specific p-values have been added directly to the graph.

9. In Figs 5D and 5E, the colors used to represent different chromosomes are inconsistent between the pie chart and the bar graph. The authors should standardize the color scheme across both panels.
Response: We thank the reviewer for this comment. We believe the comment was intended to refer to the color scheme in Figure 4D and 4E. We have standardized the colors representing the different chromosomes across these two panels as suggested, and they are now consistent. We appreciate the reviewer's careful attention to this detail.

10. In males, *roX*1 and *roX*2 are known to function redundantly in dosage compensation. It remains unclear whether a similar functional redundancy exists in females with respect to their role in promoting heterochromatinization. To address this, the

authors should consider performing a double knockdown of *roX*1 and *roX*2 and assessing whether the combined depletion has an additive effect on heterochromatin markers and ISC phenotypes compared to single knockdowns.

Response: We appreciate the rigorous comments, we are generating the double knockdown transgenic flies, while we don't get the transgenic line till now. We performed the double knockdown experiments using Kc cells. Interestingly, compared with single knockdown, double knockdown further significantly reduced the intensity of heterochromatin marker signals, including H3K9me3 and HP1a, indicating *roX*1 and *roX*2 have an additive effect on heterochromatin expansion, supporting our conclusion. Please see Appendix Figure S12.

11. Can the authors use FACS to quantify Delta$^+$ and Esg$^+$ cells under infection or aging conditions, using their existing Delta-Gal4 and Esg-Gal4 lines in combination with UAS-GFP? This would serve as an orthogonal and more quantitative approach to support the immunofluorescence-based findings and strengthen conclusions about ISC/EB population dynamics.

Response: We thank reviewer for the important suggestion, we performed the FACS experiments, we quantified the Esg$^+$ cells under aging and infection conditions, results demonstrated the significant elevation of Esg$^+$ cells, Delta$^+$ cell also exhibited significant increasing tendency under PA14 infection, all these quantification results further support our conclusion. These results have been incorporated into Responsive Figure 7.

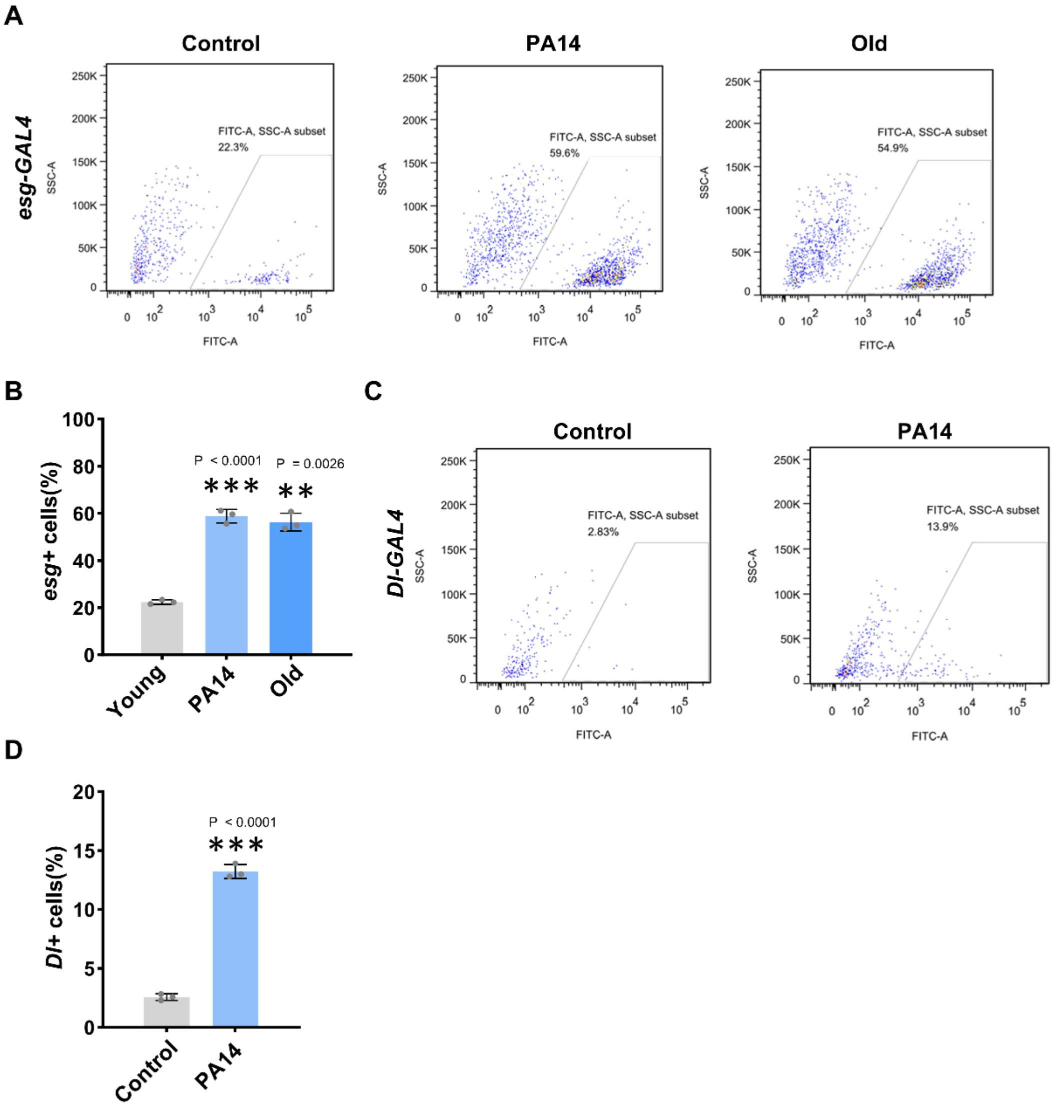

Responsive Figure 7. FACS quantification of Delta^{+} and Esg^{+} populations during ageing and infection.

(A) Gating strategy for identifying Esg^{+} cells (ISCs/EBs) from the midguts of *esg-Gal4, UAS-GFP* female flies. (B) Quantification of the percentage of Esg^{+} cells (ISCs/EBs) shows a significant increase in both aged and PA14-infected conditions compared to controls. N = 3. (C) Identification and gating of Delta^{+} ISCs in the midgut from female flies obtained by crossing *Dl-GAL4* drivers to UAS-GFP lines, under uninfected and PA14-infected conditions. (D) The percentage of Delta^{+} cells (ISCs) was quantified under the conditions shown in (C). N = 3. The center values are the averages, and the error bars indicate the s.e.m. P values were obtained by two-tailed unpaired Student's t-test. n.s., not significant, $p \geq 0.05$, *$p < 0.05$, **$p < 0.01$, ***$p < 0.0001$.

12. For the RNAi experiments in Kc cells, the methods section describes two distinct approaches: shRNA and dsRNA. The authors should clarify which RNAi method was used in each case and provide data demonstrating the knockdown efficiency for all relevant experiments.

Response: We thank reviewer for the kind comments, we have clarified RNAi method in respective figure legends, and the knockdown efficiency has been detected by qPCR experiments, results demonstrated that both two RNAi methods significantly depleted the *roX* RNAs compared to control. These results have been incorporated into Appendix Figure S6D-E, G-H, and refined figure legends are line (589, 593, 1072) in our revised manuscript.

13. The manuscript refers to Xist RNAs as homologs of roX RNAs at multiple points. This is inaccurate. Please correct this.
Response: We agree with the reviewer and have replaced the term "homolog" with "functional analogue" to more accurately describe the relationship between *Xist* and *roX* RNAs. Line 42, 381.

14. In Fig. EV7B, the reported XIST in HP1α and H3K9me3 at euchromatic arms should be more clearly indicated, for example by using arrowheads in the figure. Additionally, to confirm that roX2 is indeed overexpressed, the authors should perform roX2 RNA FISH or use an alternative method to validate overexpression.
Response: We thank reviewer for the important suggestion, we indicated the expansion foci at euchromatic arms using white arrowheads in the Figure EV4B. On the other hand, we performed the qPCR to examine the *roX*2 overexpression, the statistical results demonstrated the significant elevation of *roX*2 using our construct in the salivary gland. Please see Appendix Figure S6M.

15. The authors state in the discussion that MSLs are absent in females. This is not accurate. MSL1, MSL3, MOF and MLE are also expressed in females, albeit at low levels 1,2. roX2 pulldown-MS based experiments in both male and female cells may help assess which proteins are preferentially bound by the RNA in the different cases.
Response: We thank the reviewer for this critical correction. We have revised the text, and now state: "the absence of a functional MSL occupancy in female." Line 421.
The reviewer's comment has also prompted us to consider the role of other MSL complex members in females, and we plan to investigate this interesting question in our future work.

16. The statement that HP1α shows enhanced interaction with the U1 and D1 regions after LPS treatment may be misleading. This apparent increase could simply reflect elevated HP1α protein levels under LPS conditions, rather than a specific change in binding affinity or localization. To address this, the authors should quantify the expression levels of all HP1α constructs in both LPS^+ and LPS^- conditions, as well as the input levels of roX1 RNA.
Response: Thanks for this suggestion. We performed the western blot experiments to detect the overexpression amount for different constructs, results demonstrated that the expression levels for every construct under LPS^+ or LPS^- have no notable difference. The input levels *roX*1 RNA do increase upon LPS treatment. However, the enrichment fold in our ChIRP-qPCR data was already normalized to the respective input RNA levels. Thus, this normalization ensures that the reported enrichment specifically reflects changes in binding association. Therefore, the data therefore demonstrate that the increased signal represents a specific interaction between HP1a and the U1/D1 regions of *roX*1 following LPS treatment, rather than a reflection of elevated global RNA or protein levels.

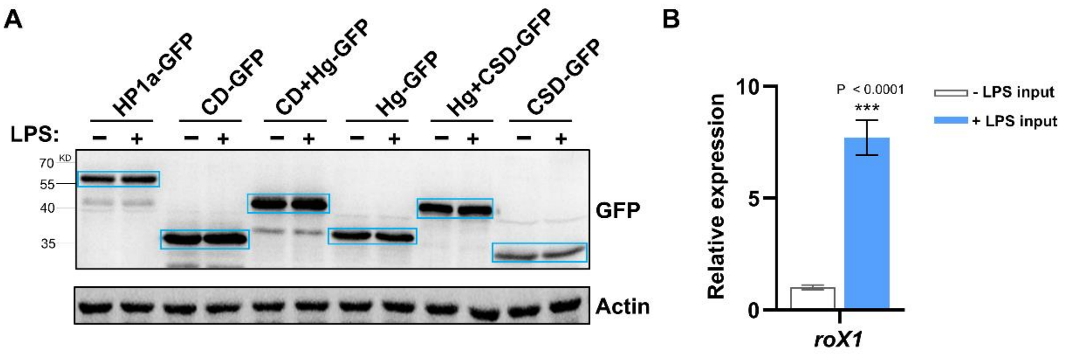

Responsive Figure 8. Expression levels of HP1α constructs and *roX*1 RNA input under LPS⁺ and LPS⁻ conditions.
(A) Protein levels of HP1a constructs remain unchanged by LPS, as determined by Western blot. The target bands are indicated with blue boxes. The predicted molecular weights are as follows: GFP (~25 kDa), HP1a-GFP (~57 kDa), CD-GFP (~35 kDa), CD+Hg-GFP (~41 kDa), Hg-GFP (~38 kDa), Hg+CSD-GFP (~40 kDa), and CSD-GFP (~33 kDa). (B) Measurement of input *roX*1 RNA levels by qPCR. N = 3. The center values are the averages, and the error bars indicate the s.e.m. P values were obtained by two-tailed unpaired Student's t-test. n.s., not significant, $p \geq 0.05$, *$p < 0.05$, **$p < 0.01$, ***$p < 0.0001$.

17. Without appropriate expression levels of Xist RNA fragments in *Drosophila* ISC/EB cells, it is difficult to determine whether the lack of rescue by Xist-D is due to insufficient expression, reduced RNA stability, or an inherent lack of functional activity (Fig. 7). Along similar lines, the authors should express an unrelated lncRNA as a control, to ensure the observed effects are unique to roX/Xist RNAs.

Response: Thanks for the rigorous comments. qRT-PCR analysis demonstrated that the *Xist-D* fragment was in fact overexpressed by more than 10,000-fold in the *Drosophila* KC cells (Appendix figure S6L). Indeed, its expression level was even higher than that of the functional *Xist-A* and *Xist-C* fragments (Appendix figure S6J-L). This confirms that the lack of rescue by *Xist-D* is due to a loss of function, not insufficient expression.

To ensure the observed rescues are unique to *roX*/*Xist* RNAs, we designed a scrambled-sequence control RNA. This was generated by randomizing the nucleotide sequence of the functional *Xist-A* fragment using an online algorithm (molbiotool's Random Sequence Generator), ensuring identical nucleotide composition but abolishing any biological sequence specificity. The sequences and cloning primers are detailed in Table EV1. QPCR analysis verified that the scrambled control RNA was substantially expressed (Appendix figure S6I). Importantly, this control RNA failed to rescue the heterochromatin loss induced by *roX*2 knockdown. In contrast, both *Xist*-A and *Xis*t-C fragments successfully restored heterochromatin levels. This result confirms that the rescue of heterochromatin is a specific function of the *roX*/*Xist* RNAs. The immunostaining results have been incorporated into Figure 7C-E.

18. The Gal4 lines used must be consistently mentioned for all the main figures and extended figures (for example- Fig.1 vs Fig. 5).

Response: We thank the reviewer for pointing out this inconsistency. We have now carefully revised the manuscript to ensure that the Gal4 drivers are consistently and explicitly mentioned in all relevant figure legends and the text for both the main and extended data figures (for example Fig.1 and Fig.5).

19. Line 174 needs to be supported with an appropriate citation.

Response: We thank the reviewer for the suggestion. We have cited respective references (doi: 10.1016/j.molcel.2013.07.001) in our revised manuscript (line 205).

20. The heading in line 304 needs to be reworded for clarity

Response: We thank the reviewer for this suggestion. The heading at line 355 in our revised manuscript has been reworded as suggested for better clarity. It now reads: "*Xist* RNA functions similarly to *roX*1/2 in Kc cells to promote heterochromatinization." Line 355.

21. Please provide access to the code used for data analysis such as your scRNA-seq in a public repository such as github, or as supplementary files.

Response: We thank the reviewer for this suggestion. All code for data analysis has been deposited in public repositories (github). The main code for scRNA-seq analysis is hosted at: https://gitfront.io/r/yhli/sG6nFFmoABrb/Dm-*roX*-codes/, with additional supplemental analysis code at https://gitfront.io/r/yhli/nGz7ZsWSHPa7/Dm-*roX*-supplement-codes/. The main code for ATAC-seq analysis is hosted at: https://gitfront.io/r/yhli/Z6mfnvWkuWNe/Dm-*roX*2OE-ATAC-codes/.These links will be permanently cited in the "Data Availability" section of the revised manuscript.

References

1. Kelley, R. L. et al. Expression of msl-2 causes assembly of dosage compensation regulators on the X chromosomes and female lethality in Drosophila. Cell 81, 867-877 (1995).

2. Gorman, M., Franke, A. & Baker, B. S. Molecular characterization of the male-specific lethal-3 gene and investigations of the regulation of dosage compensation in Drosophila. Development 121, 463-475 (1995).

Dear Dr. Ni,

Thank you for the submission of your revised manuscript, and I am sorry for the delay in getting back to you; it took quite some time to receive the report from referee 3, but I am glad it is in now, as this referee still has several, more minor, suggestions that will further improve your ms. Please address all final comments also in a point-by-point response to be submitted with your final ms.

A few editorial requests will also need to be addressed before we can proceed with the official acceptance of your manuscript:

- The following sections need to be removed from the ms file: RESOURCE AVAILABILITY, Lead contact, Materials Availability.

- The Data Availability Section (DAS) needs to be placed before the Acknowledgments

- The conflict of interest subheading needs to be corrected to "Disclosure and Competing Interests Statement" (DCIS)

- The following author name discrepancies need to be resolved as the format of the names needs to be First Name Last Name:
Chen Wang in the ms vs. Wang Chen in the system
Yabin Li in the ms vs. Li Yabin in the system
Li-Wei Liu in the ms vs. Liu Li-Wei in the system
Mingyang Li in the ms vs. Li Mingyang in the system

- The affiliation number 6 (Hangzhou Zhongnanfeier Biopharmaceutical Co., Ltd) seems to be a biotech company; employment in a biotech company should be stated in DCIS.

- The author credits need to be removed from the ms file. All credits need to be entered during online ms submission.

- There is one discrepancy in the funding information: 20221250020 is provided in the ms file while 2021YFA0805800 is provided in our online submission system, please correct.

- One callout is missing in the ms text for Fig. EV5, but a callout for Fig. EV8 is provided even though there are only 5 EV figures, please correct. Supplementary Table 1 and 2 have been called out but the files are missing. Also, "Supplementary" is not a correct callout and needs to be updated in all places to the correct nomenclature.

- The Reagents & Tools TABLE needs to be removed from the ms and uploaded as a separate file.

- The Ethical Statement should be placed somewhere in the Methods.

- Our routine image analysis detected a possible blot reuse between Figure 7F and EV4F that is not mentioned in the figure legends. Can you please clarify?

* Figure Legends - Comments *

- Please note that the exact p values are not provided in the legends of figures 1G, I;2G, 3G, H; 4B, F, G; 5G, 6C, D, I, J; 7D, E, G, H; EV1 E, EV2 E, EV3 A, EV4 C, D, G. Please provide exact values as reasonable.
- Please indicate the statistical test used for data analysis in the legend of figure 4B
- Please note that the box plots need to be defined in terms of minima, maxima, centre, bounds of box and whiskers, and percentile in the legends of figures 4B, F.
- Please note that information related to n is missing in the legends of figures 4B, F.

I would like to suggest some minor changes to the title and abstract. Please let me know whether you agree with the following:

roX1 and roX2 lncRNAs promote heterochromatinization in intestinal stem cells and impair longevity

Maintenance of the genome and epigenome stability is vital for animal longevity. The long noncoding RNAs roX1 and roX2 are known to be important in the male X chromosome dosage complex in Drosophila. However, their functions in Drosophila females have not been explored. Here we show that roX RNAs promote heterochromatin formation in intestinal stem cells (ISCs) and progenitor enteroblasts (EBs) of Drosophila females in response to pathogen infection or aging. Increased heterochromatin formation in ISCs/EBs is associated with decreased active epigenetic modifications and global gene repression. Elevation of roX RNAs in ISCs/EBs promotes heterochromatinization and represses gene expression by recruiting heterochromatin proteins such as HP1a and Su(var)3-9. Overexpression of roX RNAs promotes ISCs hyperplasia, while their inactivation mitigates ISCs

dysplasia and extends female lifespan. Interestingly, Xist RNA, the functional analogue of roX RNAs in mammals, also promotes heterochromatin formation and ISCs hyperplasia in Drosophila, and increases in aged humans. Our findings reveal a role for roX RNAs in promoting heterochromatin expansion, controlling cell fate [is this shown?] and animal longevity.

Please carefully review the full ms text and remove all overstatements. The study does not show that rox RNAs impair longevity through heterochromatin formation, for example.

EMBO press papers are accompanied online by A) a short (1-2 sentences) summary of the findings and their significance, B) 2-3 bullet points highlighting key results and C) a synopsis image that is exactly 550 pixels wide and 200-600 pixels high (the height is variable). The synopsis image should provide a sketch of the major findings, like a graphical abstract. Please note that text needs to be readable at the final size. Please send us this information along with the final manuscript.

I look forward to seeing a final version of your manuscript as soon as possible.

Best regards,
Esther

Esther Schnapp, PhD
Senior Editor
EMBO reports

Referee #1:

The authors revisions have considerably improved the quality of this manuscript. They have addressed most of the outstanding problems adequately and I appreciate inclusion of summary figures. I suggest a few remaining edits to make the manuscript more clear and accessible.
Does line 172 refer only to female tissue?
Replace roX1/2 by roX1 or roX2 (I believe the authors intend to communicate)
In line 349 the authors state that HP1a expression is increased, but two lines later they state that heterochromatinization is not caused by increases in heterochromatin proteins. I urge the authors to go over the manuscript carefully to ensure accuracy.

Referee #2:

My concerns have been appropriately addressed, and I have no further comments.

Referee #3:

The authors have made substantial efforts to address the concerns raised in the initial review, and the manuscript has been significantly strengthened both experimentally and conceptually. In particular, the inclusion of rescue experiments, validation of the genetic manipulations, incorporation of ATAC-seq analyses, and the additional experiments in males have enhanced the overall rigor of the study. The revised schematic model (Figure 8) further improves the clarity and interpretability of the work. Overall, this is an intriguing study identifying a stress-induced, female-specific role of roX RNAs in heterochromatin dynamics and intestinal stem cell regulation. The major concerns have been satisfactorily addressed. However, certain mechanistic interpretations remain stronger than the current evidence supports, and a few conceptual points would benefit from clarification. These are outlined below.

1. It is noteworthy that stress-induced heterochromatin expansion occurs in a sex-independent manner, whereas the increase in roX expression following PA14 infection is reported to be female-specific (Appendix Figure S5). This observation suggests that the mechanisms underlying heterochromatin expansion in response to infection stress may differ between males and females. While the authors discuss the sex-specific roles of roX RNAs in the context of dosage compensation in males and transcriptional repression in females (Lines 423-432), it would be important to explicitly acknowledge and discuss the apparent uncoupling between roX RNA upregulation and heterochromatinization in light of these sex-specific differences.
2. Figure 8 presents a model in which roX RNAs broadly mediate stress-induced heterochromatin expansion within the ISC/EB compartment. However, the data indicate that roX expression is preferentially restricted to a subset of $Delta^+$ ISCs, with limited evidence for expression in EBs. Although the discussion considers potential indirect, lineage-based, or parallel stress-responsive mechanisms to account for heterochromatin expansion in EBs, the current schematic appears to overstate the direct cellular scope of roX action. It would strengthen the model to more clearly distinguish roX-dependent effects in female ISCs from broader stress-induced chromatin remodeling processes that may operate in parallel or secondarily in other cell types.
3. In the response to reviewers, the authors show that increased roX expression is detected in only a subset of $Delta^+$ ISCs

(~15-20%) upon stress. This important quantitative detail is not clearly presented in the main manuscript. The authors should consider including this information in the results section and discussing how increased roX expression in a relatively small fraction of ISCs could account for the broader heterochromatin expansion observed across the ISC population under stress conditions.
4. The rescue experiments (Appendix Figure S10) are central to establishing the specificity of the roX2-dependent effects. The authors should consider integrating these key rescue data into main figure 5.
5. The FACS-based quantification of Esg^+ and $Delta^+$ populations presented in responsive figure 7 provides important quantitative validation of the ISC/EB expansion observed under aging and infection conditions. Given that these data directly support a central conclusion of the manuscript, they should be incorporated into the main or extended figures and discussed in the main text.
6. The increase in HP1a and H3K9me3 levels, upon infection as shown in Appendix Figure S2, appears modest. Quantification of band intensities across biological replicates would strengthen the conclusion that these data support heterochromatin expansion.
7. The manuscript would benefit from careful language editing to improve clarity and readability throughout.

Point-by-point response (in blue) to reviewers' comments (in black):

Dear Dr. Ni,

Thank you for the submission of your revised manuscript, and I am sorry for the delay in getting back to you; it took quite some time to receive the report from referee 3, but I am glad it is in now, as this referee still has several, more minor, suggestions that will further improve your ms. Please address all final comments also in a point-by-point response to be submitted with your final ms.

A few editorial requests will also need to be addressed before we can proceed with the official acceptance of your manuscript:

- The following sections need to be removed from the ms file: RESOURCE AVAILABILITY, Lead contact, Materials Availability.

Response: We have removed these sections from the manuscript file as requested.

- The Data Availability Section (DAS) needs to be placed before the Acknowledgments.

Response: We have moved the Data Availability Section (DAS) before the Acknowledgments as requested.

- The conflict of interest subheading needs to be corrected to "Disclosure and Competing Interests Statement" (DCIS)

Response: We have revised the subheading as requested.

- The following author name discrepancies need to be resolved as the format of the names needs to be First Name Last Name:

Chen Wang in the ms vs. Wang Chen in the system

Yabin Li in the ms vs. Li Yabin in the system

Li-Wei Liu in the ms vs. Liu Li-Wei in the system

Mingyang Li in the ms vs. Li Mingyang in the system

Response: The author names in the manuscript are correct, while the submission system can not be changed to the correct form, as they have already signed up using their respective email address.

- The affiliation number 6 (Hangzhou Zhongnanfeier Biopharmaceutical Co., Ltd) seems to be a biotech company; employment in a biotech company should be stated in DCIS.

Response: We have added a statement to the DCIS section.

- The author credits need to be removed from the ms file. All credits need to be entered during online ms submission.

Response: The author credits section has been removed from the manuscript file as requested. All contributions are now entered in the online submission system.

- There is one discrepancy in the funding information: 20221250020 is provided in the ms file while 2021YFA0805800 is provided in our online submission system, please correct.

Response: We have updated the funding numbers in the submission system and the manuscript.

- One callout is missing in the ms text for Fig. EV5, but a callout for Fig. EV8 is provided even though there are only 5 EV figures, please correct. Supplementary Table 1 and 2 have been called out but the files are missing. Also, "Supplementary" is not a correct callout and needs to be updated in all places to the correct nomenclature.

Response: We have corrected the callout for Fig. EV5 and updated Supplementary table to Table EV1.

- The Reagents & Tools TABLE needs to be removed from the ms and uploaded as a separate file.

Response: We have removed the Reagents & Tools Table from the manuscript and have uploaded it as a separate file, as requested.

- The Ethical Statement should be placed somewhere in the Methods.

Response: We have moved the Ethical Statement to the Methods section as suggested.

- Our routine image analysis detected a possible blot reuse between Figure 7F and EV4F that is not mentioned in the figure legends. Can you please clarify?

Response: Thank you for pointing out this issue. We have checked our raw data and confirmed that there was a reuse in the assembly of Figure 7F. The images in Figure 7F have now been corrected and are distinct from those in Figure EV4F. We apologize for this oversight and appreciate the opportunity to correct it.

* Figure Legends - Comments *

- Please note that the exact p values are not provided in the legends of figures 1G, I;2G, 3G, H; 4B, F, G; 5G, 6C, D, I, J; 7D, E, G, H; EV1 E, EV2 E, EV3 A, EV4 C, D, G. Please provide exact values as reasonable.

Response: We have now included the exact p values on the graphs as requested.

- Please indicate the statistical test used for data analysis in the legend of figure 4B

Response: Thank you for your comment. We have now indicated in the legend of Figure 4B that the Wilcoxon test was used for statistical analysis.

- Please note that the box plots need to be defined in terms of minima, maxima, centre, bounds of box and whiskers, and percentile in the legends of figures 4B, F.

Response: Thank you for this suggestion. We have now added detailed definitions of the box plots in the legends of Figures 4B and 4F. To be specific, from bottom to top: the lower whisker represents the 5th percentile, the lower box bound represents the 25th percentile, the centre line represents the 50th percentile (median), the upper box bound represents the 75th percentile, and the upper whisker represents the 95th percentile. The updated legends are highlighted in the revised manuscript.

- Please note that information related to n is missing in the legends of figures 4B, F.

Response: Thank you for your comment. We have now added this information to the legends of Figures 4B and 4F.

I would like to suggest some minor changes to the title and abstract. Please let me know whether you agree with the following:

roX1 and roX2 lncRNAs promote heterochromatinization in intestinal stem cells and impair longevity

Maintenance of the genome and epigenome stability is vital for animal longevity. The long noncoding RNAs roX1 and roX2 are known to be important in the male X chromosome dosage complex in Drosophila. However, their functions in Drosophila females have not been explored. Here we show that roX RNAs promote heterochromatin formation in intestinal stem cells (ISCs) and progenitor enteroblasts (EBs) of Drosophila females in response to pathogen infection or aging. Increased heterochromatin formation in ISCs/EBs is associated with decreased active epigenetic modifications and global gene repression. Elevation of roX RNAs in ISCs/EBs promotes heterochromatinization and represses gene expression by recruiting heterochromatin proteins such as HP1a and Su(var)3-9. Overexpression of roX RNAs promotes ISCs hyperplasia, while their inactivation mitigates ISCs dysplasia and extends female lifespan. Interestingly, Xist RNA, the functional analogue of roX RNAs in mammals, also promotes heterochromatin formation and ISCs hyperplasia in Drosophila, and increases in aged humans. Our findings reveal a role for roX RNAs in promoting heterochromatin expansion, controlling cell fate [is this shown?] and animal longevity.

Response: Thank you for your valuable suggestions on the title and abstract. We fully agree with your proposed changes and have revised the title and abstract. We see the hyperplasia of ISC under *roX* overexpression, but did not see the change of cell fate, so we deleted the statement.

Please carefully review the full ms text and remove all overstatements. The study does not show that rox RNAs impair longevity through heterochromatin formation, for example.

Response: Thank you for helpful suggestions. We have thoroughly revised the manuscript to remove overstatements, particularly about the relationship between *roX* RNAs, heterochromatin formation, and longevity. Line 44-45, 394-396, 489-490.

EMBO press papers are accompanied online by A) a short (1-2 sentences) summary of the findings and their significance, B) 2-3 bullet points highlighting key results and C) a synopsis image that is exactly 550 pixels wide and 200-600 pixels high (the height is variable). The synopsis image should provide a sketch of the major findings, like a graphical abstract. Please note that text needs to be readable at the final size. Please send us this information along with the final manuscript.

Response: Thank you for your instructions. We have prepared the required materials (short summary, key results bullet points, and synopsis image) for our manuscript and have uploaded them as separate files in the submission system.

I look forward to seeing a final version of your manuscript as soon as possible. Please use this link to submit your revision: https://embor.msubmit.net/cgi-bin/main.plex

Best regards,

Esther

Esther Schnapp, PhD

Senior Editor

EMBO reports

Referee #1:

The authors revisions have considerably improved the quality of this manuscript. They have addressed most of the outstanding problems adequately and I appreciate inclusion of summary figures. I suggest a few remaining edits to make the manuscript more clear and accessible.

Does line 172 refer only to female tissue?

Response: Yes, this refers to female tissue.

Replace roX1/2 by roX1 or roX2 (I believe the authors intend to communicate)

Response: Thank you for the suggestion. We have replaced *roX*1/2 with *roX*1 or *roX*2 throughout the manuscript.

In line 349 the authors state that HP1a expression is increased, but two lines later they state that heterochromatinization is not caused by increases in heterochromatin proteins. I urge the authors to go over the manuscript carefully to ensure accuracy.

Response: We thank the reviewer for pointing this out. We have revised the text to avoid any confusion between HP1a enrichment (detected by immunofluorescence) and its total protein expression (detected by Western blot). The changes are highlighted in the revised manuscript (line 350). We have carefully reviewed the manuscript and made necessary corrections to ensure accuracy.

Referee #2:

My concerns have been appropriately addressed, and I have no further comments.

Response: We thank the reviewer for the positive feedback.

Referee #3:

The authors have made substantial efforts to address the concerns raised in the initial review, and the manuscript has been significantly strengthened both experimentally and conceptually. In particular, the inclusion of rescue experiments, validation of the genetic manipulations, incorporation of ATAC-seq analyses, and the additional experiments in males have enhanced the overall rigor of the study. The revised schematic model (Figure 8) further improves the clarity and interpretability of the work.

Overall, this is an intriguing study identifying a stress-induced, female-specific role of roX RNAs in heterochromatin dynamics and intestinal stem cell regulation. The major concerns have been satisfactorily addressed. However, certain mechanistic interpretations remain stronger than the current evidence supports, and a few conceptual points would benefit from clarification. These are outlined below.

1. It is noteworthy that stress-induced heterochromatin expansion occurs in a sex-independent manner, whereas the increase in roX expression following PA14 infection is reported to be female-specific (Appendix Figure S5). This observation suggests that the mechanisms underlying heterochromatin expansion in response to infection stress may differ between males and females. While the authors discuss the sex-specific roles of roX RNAs in the context of dosage compensation in males and transcriptional repression in females (Lines 423-432), it would be important to explicitly acknowledge and discuss the apparent uncoupling between roX RNA upregulation and heterochromatinization in light of these sex-specific differences.

Response: We thank the reviewer for this comment. We agree that the apparent uncoupling between *roX* upregulation and heterochromatin expansion deserves explicit discussion. As suggested, we have added the following text to the Discussion section (lines 433-439):

"While stress-induced heterochromatin expansion occurs in both males and females, *roX* upregulation was observed only in females (Appendix Figure S6). In males, basal levels of *roX* may be sufficient to support heterochromatin expansion upon infection. Alternatively, other *roX*-independent pathways may exist that respond to stress and induce heterochromatin expansion. This uncoupling between *roX* RNA upregulation and heterochromatinization suggests that *roX* RNAs are not the only drivers of stress-induced heterochromatinization. Further investigation is necessary to elucidate the molecular mechanisms involved."

2. Figure 8 presents a model in which roX RNAs broadly mediate stress-induced heterochromatin expansion within the ISC/EB compartment. However, the data indicate that roX expression is preferentially restricted to a subset of Delta^{+} ISCs, with limited evidence for expression in EBs. Although the discussion considers potential indirect, lineage-based, or parallel stress-responsive mechanisms to account for heterochromatin expansion in EBs, the current schematic appears to

overstate the direct cellular scope of roX action. It would strengthen the model to more clearly distinguish roX-dependent effects in female ISCs from broader stress-induced chromatin remodeling processes that may operate in parallel or secondarily in other cell types.

Response: Thank you for your comment. We have revised Figure 8 to ensure the model accurately reflects the direct cellular scope of *roX* action. The updated figure now shows *roX*-dependent heterochromatin expansion occurring within the ISC compartment, and EBs have been removed from this pathway, while the potential indirect mechanisms affecting EBs remain in the discussion section.

3. In the response to reviewers, the authors show that increased roX expression is detected in only a subset of Delta^{+} ISCs (~15-20%) upon stress. This important quantitative detail is not clearly presented in the main manuscript. The authors should consider including this information in the results section and discussing how increased roX expression in a relatively small fraction of ISCs could account for the broader heterochromatin expansion observed across the ISC population under stress conditions.

Response: Thank you for this suggestion. We performed scRNA-seq analysis and noticed that only ~20% of ISCs showed significantly upregulated *roX* RNAs. In addition, we performed RT-qPCR using RNA from FACS-sorted cells and observed a significant increase in *roX* RNAs. As suggested, we have now added this information to the Results section (line 188-190). We have also included a discussion of how increased *roX* expression in a relatively small fraction of ISCs could account for the broader heterochromatin expansion observed across the ISC population. The following paragraph has been added to the Discussion section (lines 451-455):" The observation that increased *roX* expression is detected in only a subset of Delta^{+} ISCs (~15–20%) upon stress raises the question: increased *roX* expression in a relatively small fraction of ISCs could account for the broader heterochromatin expansion observed across the ISC population? One possibility is that the heterochromatin state established in *roX*-positive ISCs may be inherited by their progeny during cell division, leading to the spread of heterochromatinization across the ISC population."

4. The rescue experiments (Appendix Figure S10) are central to establishing the specificity of the roX2-dependent effects. The authors should consider integrating these key rescue data into main figure 5.

Response: We thank the reviewer for this suggestion. The key rescue results have been integrated into Figure 5A-C.

5. The FACS-based quantification of Esg^+ and $Delta^+$ populations presented in responsive figure 7 provides important quantitative validation of the ISC/EB expansion observed under aging and infection conditions. Given that these data directly support a central conclusion of the manuscript, they should be incorporated into the main or extended figures and discussed in the main text.

Response: We thank the reviewer for this suggestion. As recommended, we have now incorporated the FACS-based quantification data into Extended View Figure 1 (Fig. EV1D-F) and added a description of these results in the main text (line 126-128, 146-147).

6. The increase in HP1a and H3K9me3 levels, upon infection as shown in Appendix Figure S2, appears modest. Quantification of band intensities across biological replicates would strengthen the conclusion that these data support heterochromatin expansion.

Response: We thank the reviewer for this suggestion. Quantification of band intensities across biological replicates has now been performed and added to Appendix Figure S2. Statistical analysis confirmed a significant increase in both HP1a and H3K9me3 levels upon infection, further supporting heterochromatin expansion. The figure legend has been updated accordingly.

7. The manuscript would benefit from careful language editing to improve clarity and readability throughout.

Response: We thank the reviewer for this suggestion. We have carefully revised the manuscript to improve the language clarity and readability throughout. Changes are highlighted in the revised manuscript.

Dr. Jian-Quan Ni
Tsinghua University
School
China

Dear Dr. Ni,

I am very pleased to accept your manuscript for publication in the next available issue of EMBO reports. Thank you for your contribution to our journal.

Your manuscript will be processed for publication by EMBO Press. It will be copy edited and you will receive page proofs prior to publication. Please note that you will be contacted by Springer Nature Author Services to complete licensing and payment information.

You may qualify for financial assistance for your publication charges - either via a Springer Nature fully open access agreement or an EMBO initiative. Check your eligibility: https://link.springer.com/journal/44319/how-to-publish-with-us

Should you be planning a Press Release on your article, please get in contact with embo_production@springernature.com as early as possible in order to coordinate publication and release dates.

If you have any questions, please do not hesitate to contact the Editorial Office. Thank you for your contribution to EMBO Reports.

Best regards,
Esther

Esther Schnapp, PhD
Senior Editor
EMBO reports

>>> Please note that it is EMBO Reports policy for the transcript of the editorial process (containing referee reports and your response letter) to be published as an online supplement to each paper. If you do NOT want this, you will need to inform the Editorial Office via email immediately. More information is available here: https://link.springer.com/partners/embo-press/editorial-policies#Peer%20review

EMBO Press Author Checklist

Corresponding Author Name: jianquan Ni
Journal Submitted to: EMBO Reports
Manuscript Number: EMBOR-2025-62256V3

USEFUL LINKS FOR COMPLETING THIS FORM
The EMBO Journal - Author Guidelines
EMBO Reports - Author Guidelines
Molecular Systems Biology - Author Guidelines
EMBO Molecular Medicine - Author Guidelines

Reporting Checklist for Life Science Articles (updated January
This checklist is adapted from Materials Design Analysis Reporting (MDAR) Checklist for Authors. MDAR establishes a minimum set of requirements in transparent reporting in the life sciences (see Statement of Task: 10.31222/osf.io/9sm4x). Please follow the journal's guidelines in preparing your
Please note that a copy of this checklist will be published alongside your article.

Abridged guidelines for figures
1. Data
The data shown in figures should satisfy the following conditions:
- the data were obtained and processed according to the field's best practice and are presented to reflect the results of the experiments in an accurate and unbiased manner.
- ideally, figure panels should include only measurements that are directly comparable to each other and obtained with the same assay.
- plots include clearly labeled error bars for independent experiments and sample sizes. Unless justified, error bars should not be shown for technical
- if n<5, the individual data points from each experiment should be plotted. Any statistical test employed should be justified.
- Source Data should be included to report the data underlying figures according to the guidelines set out in the authorship guidelines on Data

2. Captions
Each figure caption should contain the following information, for each panel where they are relevant:
- a specification of the experimental system investigated (eg cell line, species name).
- the assay(s) and method(s) used to carry out the reported observations and measurements.
- an explicit mention of the biological and chemical entity(ies) that are being measured.
- an explicit mention of the biological and chemical entity(ies) that are altered/varied/perturbed in a controlled manner.
- the exact sample size (n) for each experimental group/condition, given as a number, not a range;
- a description of the sample collection allowing the reader to understand whether the samples represent technical or biological replicates (including how many animals, litters, cultures, etc.).
- a statement of how many times the experiment shown was independently replicated in the laboratory.
- definitions of statistical methods and measures:
  - common tests, such as t-test (please specify whether paired vs. unpaired), simple χ2 tests, Wilcoxon and Mann-Whitney tests, can be unambiguously identified by name only, but more complex techniques should be described in the methods section;
  - are tests one-sided or two-sided?
  - are there adjustments for multiple comparisons?
  - exact statistical test results, e.g., P values = x but not P values < x;
  - definition of 'center values' as median or average;
  - definition of error bars as s.d. or s.e.m.

Please complete ALL of the questions below.
Select "Not Applicable" only when the requested information is not relevant for your study.

Materials

Newly Created Materials	Information included in the manuscript?	In which section is the information available? (Reagents and Tools Table, Materials and Methods, Figures, Data Availability Section)
New materials and reagents need to be available; do any restrictions apply?	Yes	Reagents and Tools Table, Materials and Methods

Antibodies	Information included in the manuscript?	In which section is the information available? (Reagents and Tools Table, Materials and Methods, Figures, Data Availability Section)
For **antibodies** provide the following information:<br>- Commercial antibodies: RRID (if possible) or supplier name, catalogue number and or/clone number<br>- Non-commercial: RRID or citation	Yes	Reagents and Tools Table

DNA and RNA sequences	Information included in the manuscript?	In which section is the information available? (Reagents and Tools Table, Materials and Methods, Figures, Data Availability Section)
**Short novel DNA or RNA including primers, probes**: provide the sequences.	Yes	Table EV

Cell materials	Information included in the manuscript?	In which section is the information available? (Reagents and Tools Table, Materials and Methods, Figures, Data Availability Section)
**Cell lines:** Provide species information, strain. Provide accession number in repository **OR** supplier name, catalog number, clone number, and/**OR** RRID.	Yes	Reagents and Tools Table
**Primary cultures:** Provide species, strain, sex of origin, genetic modification status.	Not Applicable	
Report if the cell lines were recently **authenticated** (e.g., by STR profiling) and tested for mycoplasma contamination.	Not Applicable	

Experimental animals	Information included in the manuscript?	In which section is the information available? (Reagents and Tools Table, Materials and Methods, Figures, Data Availability Section)
**Laboratory animals or Model organisms:** Provide species, strain, sex, age, genetic modification status. Provide accession number in repository **OR** supplier name, catalog number, clone number, **OR** RRID.	Yes	Reagents and Tools Table
**Animal observed in or captured from the field:** Provide species, sex, and age where possible.	Yes	Materials and Methods
Please detail **housing and husbandry conditions**.	Yes	Materials and Methods

Plants and microbes	Information included in the manuscript?	In which section is the information available? (Reagents and Tools Table, Materials and Methods, Figures, Data Availability Section)
**Plants:** provide species and strain, ecotype and cultivar where relevant, unique accession number if available, and source (including location for collected wild specimens).	Not Applicable	
**Microbes:** provide species and strain, unique accession number if available, and source.	Not Applicable	

Human research participants	Information included in the manuscript?	In which section is the information available? (Reagents and Tools Table, Materials and Methods, Figures, Data Availability Section)
If collected and within the bounds of privacy constraints report on age, sex and gender or ethnicity for all study participants.	Yes	

Core facilities	Information included in the manuscript?	In which section is the information available? (Reagents and Tools Table, Materials and Methods, Figures, Data Availability Section)
If your work benefited from core facilities, was their service mentioned in the acknowledgments section?	Not Applicable	

Design

Study protocol	Information included in the manuscript?	In which section is the information available? (Reagents and Tools Table, Materials and Methods, Figures, Data Availability Section)
If study protocol has been **pre-registered, provide DOI in the manuscript**. For clinical trials, provide the trial registration number **OR** cite DOI.	Not Applicable	
Report the **clinical trial registration number** (at ClinicalTrials.gov or equivalent), where applicable.	Not Applicable	

Laboratory protocol	Information included in the manuscript?	In which section is the information available? (Reagents and Tools Table, Materials and Methods, Figures, Data Availability Section)
Provide DOI OR other citation details if **external detailed step-by-step protocols** are available.	Not Applicable	

Experimental study design and statistics	Information included in the manuscript?	In which section is the information available? (Reagents and Tools Table, Materials and Methods, Figures, Data Availability Section)
Include a statement about **sample size** estimate even if no statistical methods were used.	Yes	Figures
Were any steps taken to minimize the effects of subjective bias when allocating animals/samples to treatment (e.g. **randomization procedure**)? If yes, have they been described?	Not Applicable	
Include a statement about **blinding** even if no blinding was done.	Not Applicable	
Describe **inclusion/exclusion criteria** if samples or animals were excluded from the analysis. Were the criteria pre-established?<br><br>If sample or data points were omitted from analysis, report if this was due to attrition or intentional exclusion and provide justification.	Yes	Materials and Methods, Figures, main text
For every figure, are **statistical tests** justified as appropriate? Do the data meet the assumptions of the tests (e.g., normal distribution)? Describe any methods used to assess it. Is there an estimate of variation within each group of data? Is the variance similar between the groups that are being statistically compared?	Yes	Figures

Sample definition and in-laboratory replication	Information included in the manuscript?	In which section is the information available? (Reagents and Tools Table, Materials and Methods, Figures, Data Availability Section)
In the figure legends: state number of times the experiment was **replicated** in laboratory.	Yes	Figures
In the figure legends: define whether data describe **technical or biological replicates**.	Yes	Figures

Ethics

Ethics	Information included in the manuscript?	In which section is the information available? (Reagents and Tools Table, Materials and Methods, Figures, Data Availability Section)
Studies involving **human participants**: State details of **authority granting ethics approval** (IRB or equivalent committee(s), provide reference number for approval.	Yes	Ethical Statement
Studies involving **human participants**: Include a statement confirming that **informed consent** was obtained from all subjects and that the experiments conformed to the principles set out in the WMA Declaration of Helsinki and the Department of Health and Human Services Belmont Report.	Yes	Ethical Statement
Studies involving **human participants:** For publication of **patient photos**, include a statement confirming that consent to publish was obtained.	Not Applicable	
Studies involving experimental **animals**: State details of **authority granting ethics approval** (IRB or equivalent committee(s), provide reference number for approval. Include a statement of compliance with ethical regulations.	Not Applicable	
Studies involving **specimen and field samples:** State if relevant **permits** obtained, provide details of authority approving study; if none were required, explain why.	Not Applicable	

Dual Use Research of Concern (DURC)	Information included in the manuscript?	In which section is the information available? (Reagents and Tools Table, Materials and Methods, Figures, Data Availability Section)
Could your study fall under dual use research restrictions? Please check biosecurity documents and list of **select agents and toxins** (CDC): https://www.selectagents.gov/sat/list.htm	Not Applicable	
If you used a select agent, is the security level of the lab appropriate and reported in the manuscript?	Not Applicable	
If a study is subject to dual use research of concern regulations, is the name of the **authority granting approval and reference number** for the regulatory approval provided in the manuscript?	Not Applicable	

Reporting

The MDAR framework recommends adoption of discipline-specific guidelines, established and endorsed through community initiatives. Journals have their own policy about requiring specific guidelines and recommendations to complement MDAR.

Adherence to community standards	Information included in the manuscript?	In which section is the information available? (Reagents and Tools Table, Materials and Methods, Figures, Data Availability Section)
State if relevant guidelines or checklists (e.g., **ICMJE, MIBBI, ARRIVE, PRISMA**) have been followed or provided.	Not Applicable	
For **tumor marker prognostic studies,** we recommend that you follow the **REMARK** reporting guidelines (see link list at top right). See author guidelines, under 'Reporting Guidelines'. Please confirm you have followed these guidelines.	Not Applicable	
For **phase II and III randomized controlled trials**, please refer to the **CONSORT** flow diagram (see link list at top right) and submit the CONSORT checklist (see link list at top right) with your submission. See author guidelines, under 'Reporting Guidelines'. Please confirm you have submitted this list.	Not Applicable	

Data Availability

Data availability	Information included in the manuscript?	In which section is the information available? (Reagents and Tools Table, Materials and Methods, Figures, Data Availability Section)
Have **primary datasets** been deposited according to the journal's guidelines (see 'Data Deposition' section) and the respective accession numbers provided in the Data Availability Section?	Yes	Data Availabity
Were **human clinical and genomic datasets** deposited in a public access-controlled repository in accordance to ethical obligations to the patients and to the applicable consent agreement?	Not Applicable	
Are **computational models** that are central and integral to a study available without restrictions in a machine-readable form? Were the relevant accession numbers or links provided?	Yes	Data Availabity
If publicly available data were reused, provide the respective **data citations in the reference list**.	Yes	Reference list
